# Model Based Targeting of IL-6-Induced Inflammatory Responses in Cultured Primary Hepatocytes to Improve Application of the JAK Inhibitor Ruxolitinib

**DOI:** 10.3389/fphys.2017.00775

**Published:** 2017-10-09

**Authors:** Svantje Sobotta, Andreas Raue, Xiaoyun Huang, Joep Vanlier, Anja Jünger, Sebastian Bohl, Ute Albrecht, Maximilian J. Hahnel, Stephanie Wolf, Nikola S. Mueller, Lorenza A. D'Alessandro, Stephanie Mueller-Bohl, Martin E. Boehm, Philippe Lucarelli, Sandra Bonefas, Georg Damm, Daniel Seehofer, Wolf D. Lehmann, Stefan Rose-John, Frank van der Hoeven, Norbert Gretz, Fabian J. Theis, Christian Ehlting, Johannes G. Bode, Jens Timmer, Marcel Schilling, Ursula Klingmüller

**Affiliations:** ^1^Division Systems Biology of Signal Transduction, German Cancer Research Center, Heidelberg, Germany; ^2^Discovery Division, Merrimack Pharmaceuticals, Cambridge, MA, United States; ^3^Institute of Physics, Albert Ludwigs University of Freiburg, Freiburg, Germany; ^4^BIOSS Centre for Biological Signalling Studies, Albert Ludwigs University of Freiburg, Freiburg, Germany; ^5^Clinic of Gastroenterology, Hepatology and Infectious Diseases, University Hospital, Heinrich Heine University of Düsseldorf, Düsseldorf, Germany; ^6^Institute of Computational Biology, Helmholtz Zentrum München, Neuherberg, Germany; ^7^Department of Hepatobiliary Surgery and Visceral Transplantation, Leipzig University, Leipzig, Germany; ^8^Institute of Biochemistry, University of Kiel, Kiel, Germany; ^9^Transgenic Service, Center for Preclinical Research, German Cancer Research Center, Heidelberg, Germany; ^10^Medical Research Center, Medical Faculty Mannheim, University of Heidelberg, Mannheim, Germany; ^11^Department of Mathematics, Technical University of Munich, Garching, Germany

**Keywords:** IL-6, mathematical modeling, acute phase response, ruxolitinib, primary hepatocytes

## Abstract

IL-6 is a central mediator of the immediate induction of hepatic acute phase proteins (APP) in the liver during infection and after injury, but increased IL-6 activity has been associated with multiple pathological conditions. In hepatocytes, IL-6 activates JAK1-STAT3 signaling that induces the negative feedback regulator SOCS3 and expression of APPs. While different inhibitors of IL-6-induced JAK1-STAT3-signaling have been developed, understanding their precise impact on signaling dynamics requires a systems biology approach. Here we present a mathematical model of IL-6-induced JAK1-STAT3 signaling that quantitatively links physiological IL-6 concentrations to the dynamics of IL-6-induced signal transduction and expression of target genes in hepatocytes. The mathematical model consists of coupled ordinary differential equations (ODE) and the model parameters were estimated by a maximum likelihood approach, whereas identifiability of the dynamic model parameters was ensured by the Profile Likelihood. Using model simulations coupled with experimental validation we could optimize the long-term impact of the JAK-inhibitor Ruxolitinib, a therapeutic compound that is quickly metabolized. Model-predicted doses and timing of treatments helps to improve the reduction of inflammatory APP gene expression in primary mouse hepatocytes close to levels observed during regenerative conditions. The concept of improved efficacy of the inhibitor through multiple treatments at optimized time intervals was confirmed in primary human hepatocytes. Thus, combining quantitative data generation with mathematical modeling suggests that repetitive treatment with Ruxolitinib is required to effectively target excessive inflammatory responses without exceeding doses recommended by the clinical guidelines.

## Introduction

Increased activity of interleukin (IL)-6 has been associated with chronic inflammatory diseases including rheumatoid arthritis (Hirano et al., 1988), multiple sclerosis (Frei et al., 1991; Navikas et al., 1996), and Crohn's disease (Ito, 2003). High IL-6 levels are also frequently found and correlate with poor outcome in patients with sepsis, an acute systemic inflammatory response (Waage et al., 1989; Calandra et al., 1991; Damas et al., 1992; Norrby-Teglund et al., 1995). Accordingly, abrogation of glycoprotein 130 (gp130)-dependent signaling in hepatocytes was shown to prolong survival and to reduce liver damage in an *in vivo* sepsis model (Klein et al., 2007). Persistent inflammation can initiate or promote (Grivennikov and Karin, 2011) malignant progression and a pro-tumorigenic role of IL-6, which is elevated in many types of cancer, has been suggested (Heikkila et al., 2008). Thus, increased IL-6 levels can have detrimental effects. On the other hand, a certain amount of IL-6 is required for efficient immune defense (Kopf et al., 1994) and liver regeneration (Cressman et al., 1996; Sakamoto et al., 1999; Zimmers et al., 2003). Central target cells of IL-6 are hepatocytes, where IL-6 regulates the production of acute phase proteins (APPs) by first activating the IL-6 receptor complex with the signal-transducing subunit gp130. Signals are further transduced via janus kinase 1 (JAK1) and signal transducer and activator of transcription 3 (STAT3; Bode and Heinrich, 2001).

However, other cytokines such as Oncostatin-M (OSM), IL-11, IL-10, and IL-22, also induce STAT3 phosphorylation (see Nakamura et al., 2004; Sabat et al., 2010; Nishina et al., 2012; Rao et al., 2014) and therefore could contribute to the complex regenerative and inflammatory signaling in the liver. OSM is also able to induce IL-6 expression and therefore additionally feeds into JAK1/STAT3 signaling. However, OSM is primarily involved in developmental processes (Nakamura et al., 2004) and presumably only contributes to a lesser extent to the immediate activation of the acute phase response upon liver damage. IL-11 was shown to be mainly involved in hepatocellular responses upon oxidative stress and hepatotoxic drugs (Nishina et al., 2012). The anti-inflammatory cytokine IL-10 is an essential factor controlling inflammation (Murray, 2006). After partial hepatectomy Yin et al. observed after 1 h an increase in *Il10* mRNA expression, but the concentration of IL-10 protein was not examined (Yin et al., 2011). Distinct from IL-6, IL-10 apparently does not induce the expression of suppressor of cytokine signaling 3 (SOCS3), (Ichikawa et al., 2002) and in mice lacking a functional *Socs3* gene in macrophages or neutrophils no obvious alteration in IL-10 signal transduction is observed (Yasukawa et al., 2003). For IL-22 Rao et al. observed in hepatectomized mice in comparison to sham operated mice an increase of *Il22* mRNA after 1 h and a further increase after 3 h, whereas *Il6* mRNA was already maximally induced after 1 h (Rao et al., 2014). On the other hand Ren et al. did not detect a statistically significant increase of *Il22* mRNA in mice in response to hepatectomy, but rather reported a statistically significant increase of IL-22 protein in the serum starting at 6 h post hepatectomy with a peak at 12 h (Ren et al., 2010). Further, in response to LPS injection a very low level of induction of *Il22* mRNA was observed in the liver with a peak at 4 h post injection, whereas a much stronger activation of *Il22* mRNA with comparable kinetics was observed in the spleen (Wegenka et al., 2007). Likewise, Dumoutier et al. reported that IL-22 is primarily produced by innate spleen cells in mice. These studies showed a peak of *Il22* mRNA in the serum after 2–3 h post LPS injection and elevated serum levels of IL-22 at 4 h post treatment (Dumoutier et al., 2011). Analysis of IL-22 knockout mice revealed that in the absence of IL-22 hepatocellular proliferation at 48 h post hepatectomy is reduced (Kudira et al., 2016). Further, 6 h post LPS injection a very heterogeneous decrease in STAT3 phosphorylation is observed in IL-22 knockout mice compared to wild type mice (Wallace and Subramaniam, 2015) and the authors concluded that the IL-22 knockout mice display appropriate inflammatory responses to LPS in the liver. Together these studies suggest that IL-22 is a mediator of the cross-talk between immune cells and hepatocytes and contributes to efficient liver regeneration but potentially distinct from IL-6 primarily contributes to long-term recovery.

IL-6/STAT3-dependent target genes encode the APPs fibrinogen-γ (*Fgg*), serum amyloid P (*Apcs*), haptoglobin (*Hp*), hemopexin (*Hpx*; Alonzi et al., 2001), hepcidin (*Hamp*; Wrighting and Andrews, 2006; Pietrangelo et al., 2007), as well as *Socs3*, the negative feedback regulator of IL-6 signaling (Starr et al., 1997; Croker et al., 2003). Although, APPs fulfill beneficial roles in host defense and tissue repair (Bode et al., 2012), several adverse effects have been reported for different APPs. Hepcidin, for instance, a crucial regulator of iron homeostasis (Sakamori et al., 2010; Ganz and Nemeth, 2012), contributes to the development of anemia under inflammatory conditions (Weinstein et al., 2002; Kemna et al., 2005). Elevated expression of fibrinogen was related to formation and progression of atherosclerotic plaques (Levenson et al., 1995) and serum amyloid P, the major APP in mice, was suggested to contribute to the persistence of amyloid deposits (Tennent et al., 1995). Dysregulated APP production may thus foster pathologic changes during uncontrolled inflammatory responses.

Triggered by its involvement in several pathologies, therapeutic targeting of IL-6 signaling is a focus of ongoing basic and clinical investigations. The JAK1/2 inhibitor Ruxolitinib/INCB018424 (Jakavi/Jakafi, Incyte Pharmaceuticals, Novartis; Lin et al., 2009; Quintas-Cardama et al., 2010) has been internationally approved for the therapy of myelofibrosis (Mesa et al., 2012; Verstovsek et al., 2012) and polycythemia vera (Vannucchi et al., 2015), which are frequently caused by the V617F gain-of-function mutation within JAK2 (Kralovics et al., 2005). According to the guidelines, the recommendation for Jakavi is a repetitive, constant dose of 10 mg twice daily (q12h) for polycythemia vera or 20 mg twice daily (q12h) for myelofibrosis (Rote, 2016). Ruxolitinib affects the hematological status of patients and therefore the platelet count should be assessed before the start of a therapy. Since low neutrophil counts have been observed in 66% of healthy volunteers treated with 100 mg Ruxolitinib daily (q24h; Shi et al., 2011), it is of great importance not to exceed the recommended daily doses. Ruxolitinib is also tested for the treatment of other malignancies as well as chronic inflammatory diseases such as rheumatoid arthritis (Williams et al., 2008; Quintas-Cardama et al., 2011).

The STAT3 inhibitor Stattic, which is not approved for clinical applications, targets the STAT3 SH2 domain, thus blocking receptor association and dimerization. Stattic treatment inhibited IL-6-induced STAT3 phosphorylation and nuclear translocation in hepatocytes (Schust et al., 2006). Moreover, increased apoptosis was observed in STAT3-dependent cancer cell lines upon Stattic treatment (Schust et al., 2006). Although the molecular mechanisms of Ruxolitinib and Stattic are well-established, their impact on the dynamics of signal transduction, expression of target genes, and cellular response is, due to the non-linear reactions, not intuitive.

Mathematical models based on ordinary differential equations (ODEs) are well-suited to study the dynamics of signal transduction and have enabled the identification of therapeutic targets within signaling networks (Schoeberl et al., 2009; Raia et al., 2011). ODEs describe concentration changes of species over time. The law of mass-action kinetics defines a reaction rate to be proportional to the concentrations of reacting biomolecules thus facilitating the translation of a pathway map into a set of ODEs. In the model, species concentrations are the state variables, while rate constants, initial conditions, or other proportionality factors are termed parameters. Although, some parameter values such as initial protein concentrations may be accessible by measurements, most parameter values remain unknown and have to be estimated based on experimental data (Aldridge et al., 2006; Chen et al., 2010). This process is called model calibration and requires highly quantitative and reproducible experimental data, as well as a sufficient number of data points and measured species (Bachmann et al., 2012).

Formulating biological hypotheses in terms of mathematical models allows to quantitatively test such hypotheses by challenging model predictions with additional experimental data. For example Swameye et al. established a dynamic pathway model for the JAK2-STAT5 signaling pathway and tested conflicting hypothesis on signal transduction from the cell surface receptor to the nucleus (Swameye et al., 2003). The mathematical model revealed that STAT5 acts as a remote sensor for receptor activation and that repeated nucleocytoplasmic cycling of STAT5 is required for effective target gene activation in the nucleus. Furthermore, by a mathematical modeling approach Sasagawa et al. showed that the transient activation of ERK depends on rapid increases in the amount of epidermal growth factor and nerve growth factor (NGF), while sustained ERK activation depends on the final NGF concentration (Sasagawa et al., 2005). Nelson et al. revealed that oscillations observed in TNFalpha induced activation of NF-kB control the dynamics of gene expression. The mathematical modeling approach revealed that two molecular species were strongly coupled to the oscillation dynamics (Nelson et al., 2004). By iteratively combining mathematical modeling with model-guided experiments, these and other studies (Alon et al., 1999; Sick et al., 2006; Borisov et al., 2009; Becker et al., 2010; Bachmann et al., 2011) demonstrated that it is possible to capture biological behavior, reject hypotheses which fail to describe data and make non-trivial predictions for validation experiments. Moreover, uncertainty analysis can give insight into how well a model is constrained and what kind of predictive power one can expect when predicting similar experiments (Kreutz et al., 2012; Vanlier et al., 2013). Additionally, validation experiments guided by well-constrained predictions can be performed to improve the confidence in the model (Steiert et al., 2012).

Although several ODE-based mathematical models of IL-6 signaling have been reported to date (Singh et al., 2006; Moya et al., 2011; Dittrich et al., 2012), only with a recently described mathematical model (Xu et al., 2015) potential effects of targeting selected pathway components on APP expression were tested *in silico*. These studies predicted that IL-6 signaling could be best targeted at the receptor level, and that reduced inhibitor dose may be achievable by applying possible inhibitor combinations (Xu et al., 2015). However, the model-based predictions reported by Xu et al. were not experimentally validated, thus limiting applicability to targeting IL-6 signaling in human disease.

Here we present an ODE model of IL-6-induced JAK1-STAT3 signaling in primary mouse hepatocytes. Based on extensive experimental data, the mathematical model describes pathway activation and key target gene induction during regenerative and inflammatory conditions, as well as the impact of the pathway inhibitors Ruxolitinib and Stattic. We combined model predictions with experimental validation to optimize the long-term Ruxolitinib-mediated reduction of APP gene expression, while maintaining gene expression levels that are present during regenerative conditions without employing excessive inhibitor concentrations. The presented approach represents a starting point for systematic clinical intervention in inflammatory or malignant diseases.

## Results

### Physiological IL-6 concentrations during liver regeneration and inflammation

**Figure 1:**
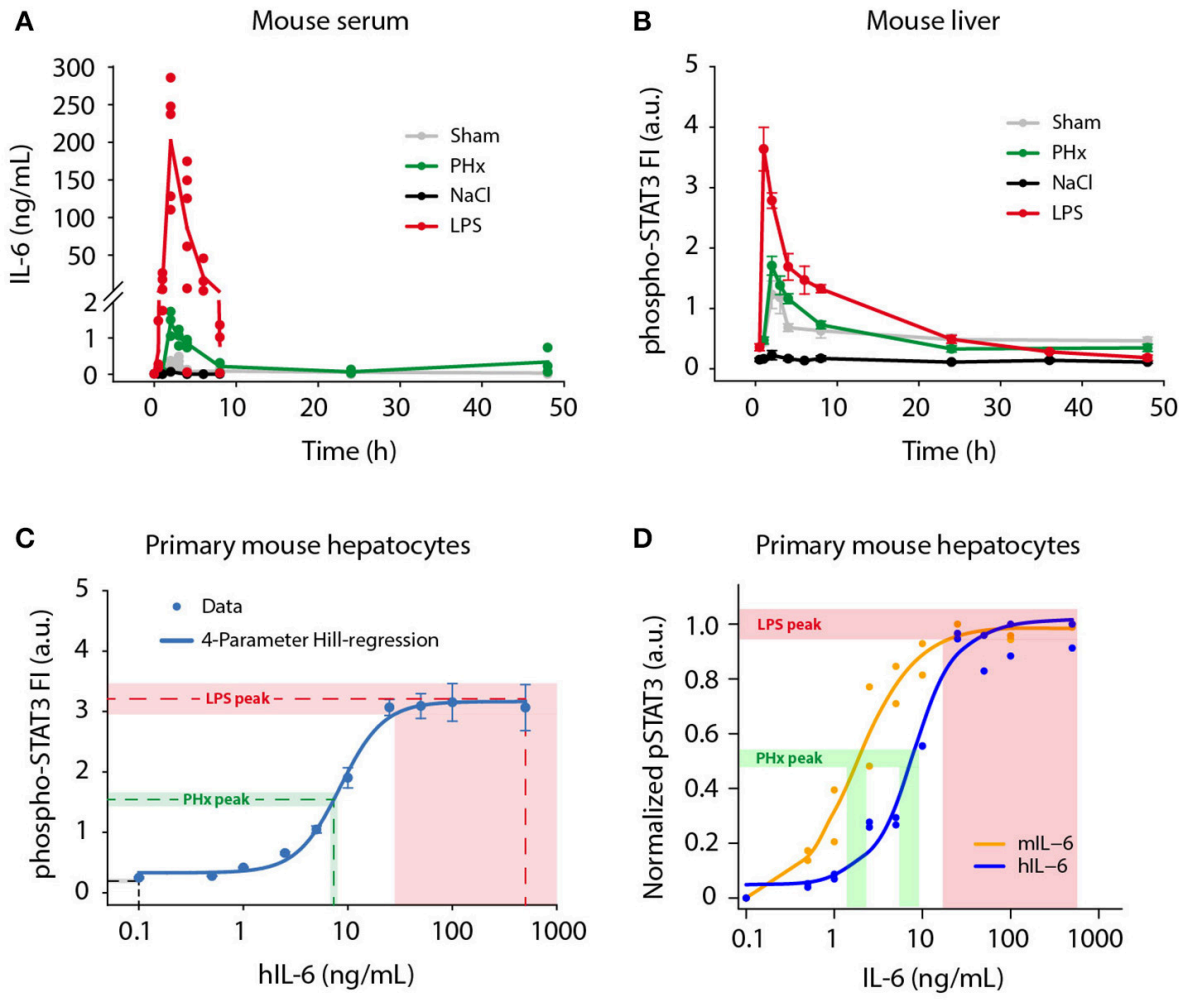
Physiological IL-6 concentrations during liver regeneration and inflammation. Mice were subjected to control (Sham) surgery or partial hepatectomy (PHx), and control (NaCl) or lipopolysaccharide (LPS, 1 μg/g body weight) treatment. Serum and livers were collected at indicated time points. **(A)** Absolute serum IL-6 levels were measured by a bead-based immunoassay. Filled circles represent data from individual mice, solid lines connect average values of biological replicates. Sham, PHx, and LPS-treatment: *n* = 3–6 per time point; NaCl: *n* = 1 per time point. **(B)** STAT3 phosphorylation at Tyr-705 was quantified by multiplexed bead-based arrays in lysates of frozen mouse liver samples. Per time point or IL-6 dose, 3–6 biological replicates were measured and scaled by normalizing to their average signal. Filled circles represent average ±standard error of the mean (SEM) of biological replicates; solid lines are shown for visual guidance in the mouse liver data set. **(C)** Primary mouse hepatocytes were stimulated with 0.1–500 ng/mL of recombinant human IL-6, and lysed after 20 min. The 4-parameter Hill regression function **(C)** was generated using SigmaPlot software, and served to convert phospho-STAT3 signals to IL-6 concentrations. Dashed lines represent average phospho-STAT3 signals of the 1 and 2 h time points (NaCl, LPS), or 2 and 3 h time points (PHx), and corresponding derived IL-6 concentrations. Shaded areas represent standard error of the mean. **(D)** Primary mouse hepatocytes were stimulated with mouse IL-6 or human IL-6 to derive the doses of human IL-6 mimicking regenerative and inflammatory conditions, indicating that 1.8 ng of mouse IL-6 is equally potent to 7.5 ng of human IL-6 on mouse hepatocytes. FI, fluorescence intensity; a.u., arbitrary units.

A broad range of circulating IL-6 concentrations has been reported during liver regeneration (Slotwinski et al., 2002; Nechemia-Arbely et al., 2011; Yin et al., 2011) and inflammation (Waage et al., 1989; Damas et al., 1992; Piao et al., 2013), but a direct comparison of regenerative and inflammatory conditions has not been performed yet. To provide a basis for our *ex vivo* experiments and to enable model predictions of physiological relevance, we determined physiological IL-6 concentrations in mice following partial hepatectomy (PHx; Mitchell and Willenbring, 2008) and lipopolysaccharide (LPS) injection (Fattori et al., 1994; Copeland et al., 2005), which trigger liver regeneration and acute inflammation, respectively. Serum IL-6 levels were measured using a bead-based immunoassay. We observed rapid but transient induction of serum IL-6 in response to PHx and LPS treatment (Figure 1A). Peak IL-6 levels were detected 2 h post PHx with 1.4 ng/mL (±0.3 ng/mL *SD*; *n* = 3). Similarly, IL-6 amounts in response to LPS injection peaked at 2 h, but reached considerably higher concentrations of 201.8 ng/mL (±77.8 ng/mL *SD*; *n* = 5). Following the peak, IL-6 levels dropped quickly and returned to baseline levels at 8–24 h. For comparison, sham surgery and NaCl injection as control treatments for PHx and LPS, respectively, caused serum IL-6 levels to increase only slightly. Less than 0.4 ng/mL IL-6 after sham surgery and <0.1 ng/mL IL-6 after NaCl injection were measured. The background IL-6 concentration in untreated mice was <10 pg/mL, which corresponds to previously reported values (Huang et al., 2003). To conclude, IL-6 levels in response to PHx and LPS treatment increased in a fast but transient manner. Similar dynamics were observed for PHx and LPS treatment. However, LPS caused 100-fold higher peak IL-6 levels, compared to PHx.

In addition to serum IL-6 levels, we developed a strategy to determine the IL-6 concentrations present in the hepatocytes' microenvironment. We analyzed STAT3 activation (Tyr-705 phosphorylation) as read-out in livers from PHx and LPS-treated mice. Phospho-STAT3 levels were measured using multiplexed bead-based arrays (Figure 1B), revealing rapid and transient induction of STAT3 activation after PHx and LPS treatment. Peak levels were reached at 2 h following PHx and even earlier, after 1 h following LPS injection. Thus, the conversion of IL-6 signal to STAT3 activation is very efficient. The amplitude of STAT3 activation after LPS treatment was more than twice as high as in the case of PHx. After peaking, the phospho-STAT3 signal decreased likewise in PHx and LPS treated livers, and returned to levels close to baseline at 24 h. STAT3 activation was also detectable in animals treated with sham surgery, which has been reported previously (Cressman et al., 1996; Heim et al., 1997), and which is likely due to stress caused by the surgical procedure.

To compare the contribution of IL-6 and of the other STAT3 activating cytokines IL-22, OSM, or IL-11, on STAT3 phosphorylation in the liver, we performed additional time-resolved qRT-PCR measurements of liver lysates from LPS or PHx treated mice. In comparison to the induction of IL-6 protein expression (Figure 1A) and *Il6* gene expression (Appendix Figure S14) no major induction of OSM, IL-22, or IL-11 was elicited by partial hepatectomy or by LPS (Appendix Figure S14).

In parallel, phospho-STAT3 levels were determined in primary mouse hepatocytes stimulated with 0.1–500 ng/mL recombinant human IL-6 (hIL-6) for 20 min to capture the maximal phospho-STAT3 signal (Figure 1C). When the work of the presented study was initiated, recombinant murine IL-6 was not yet commercially available. Therefore, human IL-6, produced as described in Vandam et al. (1993), was utilized and was kept for the entire study to ensure consistency.

Samples from mouse livers (Figure 1B) and from primary mouse hepatocytes (Figure 1C) were analyzed simultaneously in a 96-well plate format using equal lysis conditions for direct comparability of the measured phospho-STAT3 signal. Dose-dependent STAT3 activation in primary mouse hepatocytes followed a sigmoidal behavior in response to IL-6. It was detectable from 2.5 ng/mL hIL-6 on, then steeply increased and quickly reached saturation at 25–50 ng/mL (Figure 1C). Approximation of the IL-6/phospho-STAT3 dose-response curve from primary mouse hepatocytes by a 4-parameter Hill regression function enabled to correlate phospho-STAT3 signal intensities in livers from PHx or LPS-treated mice to IL-6 concentrations that elicited the observed STAT3 response. The peak phospho-STAT3 signal (average ± SEM of 2 and 3 h time points) after PHx approximately corresponded to an IL-6 concentration equivalent to 6.8–7.9 ng/mL hIL-6. In the case of LPS, the peak phospho-STAT3 signal (average ± SEM of 1 and 2 h time points) approximately corresponded to a signal obtained with 28.1–500 ng/mL hIL-6. The STAT3 signal detected after NaCl injection was out of range of our reference curve and corresponded to <0.1 ng/mL hIL-6 (Figure 1C).

To convert the responses elicited by hIL-6 to the concentrations relevant in the mouse, we performed dose response experiments comparing the potency of increasing doses of human and murine IL-6 in stimulating STAT3 phosphorylation in primary mouse hepatocytes. As shown in Figure 1D, this revealed that murine IL-6 is more potent to elicit STAT3 phosphorylation in murine hepatocytes compared to hIL-6 with an overall shift of the dose-response curve to lower IL-6 concentrations. Collectively, STAT3 was activated rapidly, efficiently and transiently in mouse livers after PHx and LPS treatment. In line with IL-6 serum concentrations (Figure 1A), peak phospho-STAT3 signals corresponded to human IL-6 concentration ranges for PHx (6.8–7.9 ng/mL; average: 7.4 ng/mL) and LPS (28.1–500 ng/mL; average: 264.1 ng/mL). This corresponds to a mouse IL-6 concentration of 1.8 and 50 ng/mL, respectively (Figure 1D).

### Time-resolved characterization of key IL-6 target genes

**Figure 2:**
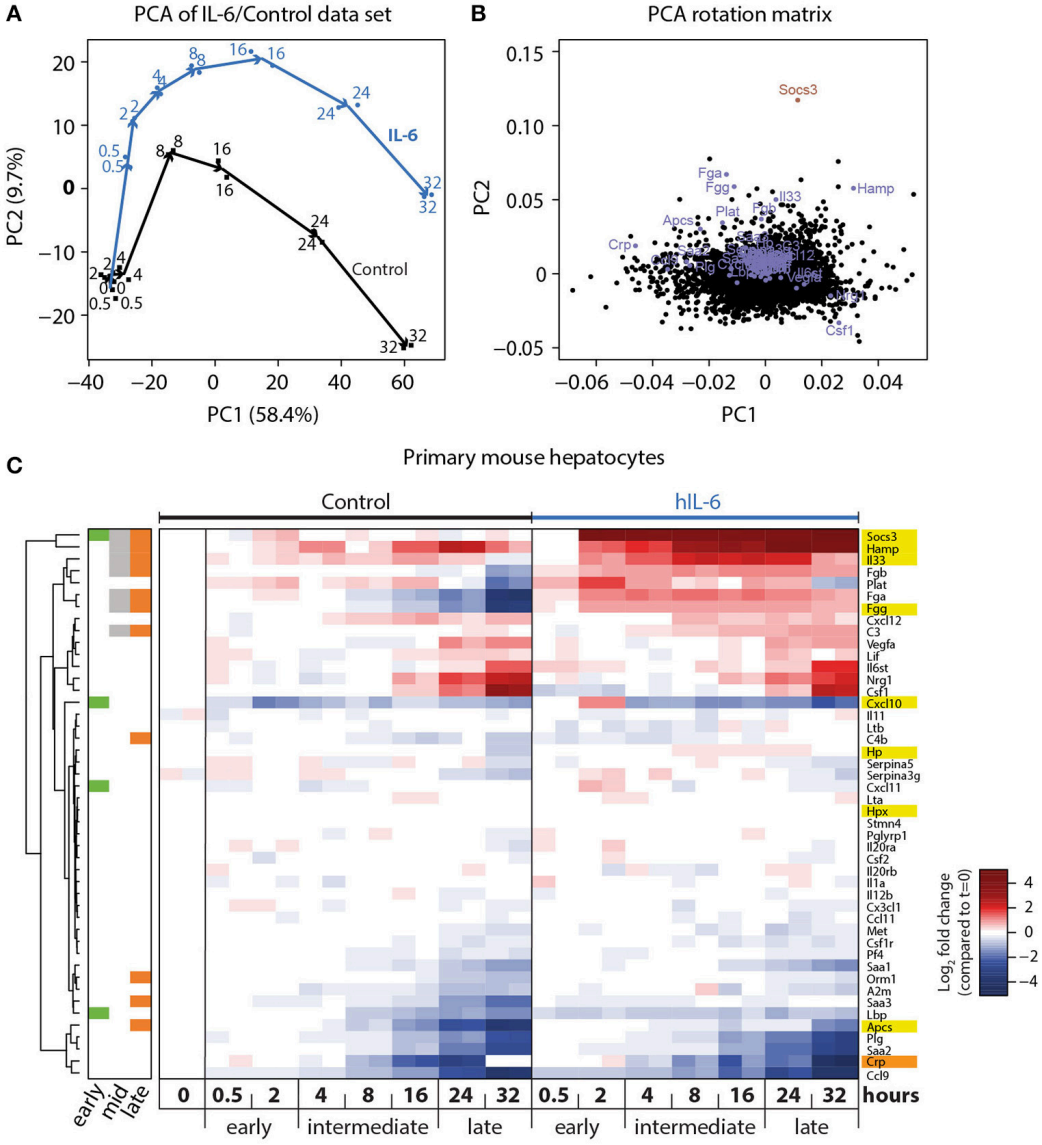
Genome-wide analysis of IL-6-induced transcriptional responses. **(A)** Primary mouse hepatocytes were stimulated with 40 ng/mL hIL-6 or left untreated (Control), and RNA was isolated at indicated time points. Transcriptome profiling was performed using GeneChip Mouse Genome 430 2.0 Arrays (Affymetrix). Samples were projected onto the first two principal components (PC) of the data set, and values in PC1/2 are plotted. Arrows connect subsequent time points for Control and IL-6 condition, and are shown for visual guidance. **(B)** Contributions of individual genes to the respective PCs are shown. Genes of interest are labeled and highlighted in blue (APP), or dark red (*Socs3*). **(C)** Hierarchical clustering of expression patterns of APP genes of interest. Expression values are centered to mean expression of *t* = 0 Control samples. Sidebar color-codes whether genes were significantly regulated at early, intermediate, or late time points. APPs that are most relevant in the murine context are highlighted in yellow. CRP, an important APP in the human context, is highlighted in orange.

To investigate the APP gene signature induced by hIL-6 in primary mouse hepatocytes, and to establish the time-dependent regulation of respective genes, we performed microarray analysis of primary mouse hepatocytes stimulated with hIL-6 (40 ng/mL) for up to 32 h. Global analysis of the genome-wide transcriptome profiling was performed using principal component (PC) analysis (PCA). In the two most relevant PCs the samples were separated by time and by condition (control vs. hIL-6) and biological duplicates were clustered (Figure 2A) indicating high reproducibility. The individual contributions of genes to the two PCs are shown in the respective rotation space for PC1/2 (Figure 2B). We found well-established IL-6 targets, such as *Socs3* and the APP genes *Apcs, Fgg*, and *Hamp* to be major contributors to both, stimulus-specific and time-dependent, regulation (Figure 2B).

Differential gene expression analysis of the microarray data set was performed using a linear regression model with gene-wise Bayesian variance estimation (Ritchie et al., 2015). We classified the IL-6-regulated genes as early (0.5–2 h), intermediate (4–16 h), and late (24–32 h) response genes to establish optimized time frames for the measurements (Figure 2C). In total 1,728 genes were significantly regulated upon hIL-6 stimulation (Appendix Figure S1A), while 723, 779, and 694 genes were IL-6-regulated at early, intermediate, and late time points, respectively. Intermediate and late IL-6-response genes showed more than 40% overlap. Enrichment analysis of respective gene lists showed that the late IL-6 response was enriched for genes relevant in the acute phase response (Appendix Figure S1B). Significantly regulated genes included *Socs3*, which was induced at early, intermediate, and late time points. Another early-induced gene was *Cxcl10*. We found the APP genes *Fgg, Hamp*, and *Il33* to be induced at intermediate and late time points, whereas compared with control *Apcs* was increased only at late time points. Interestingly, the gene encoding the C reactive protein (CRP), displayed a similar expression pattern as *Apcs*. Hierarchical clustering of significantly regulated genes and further APP genes of interest (Heinrich et al., 1990) revealed that especially late APP genes clustered and were mostly induced upon hIL-6 treatment (Figure 2C).

Taken together, we present a comprehensive list of IL-6 target genes that are expressed in response to hIL-6 stimulation in primary mouse hepatocytes. Of these we obtained detailed time-resolved expression profiles of previously known (*Apcs, Fgg, Hamp, Hp, Hpx, Socs3*) and less well-established (*Cxcl10, Il33*) IL-6 target genes. These selected genes served as read-out for the IL-6-induced hepatic acute phase response in the following experiments, and the recorded temporal dynamics enabled the choice of optimal time points for dose-dependent analysis.

### Dynamic mathematical model of IL-6 signaling capturing inhibitor effects

**Figure 3:**
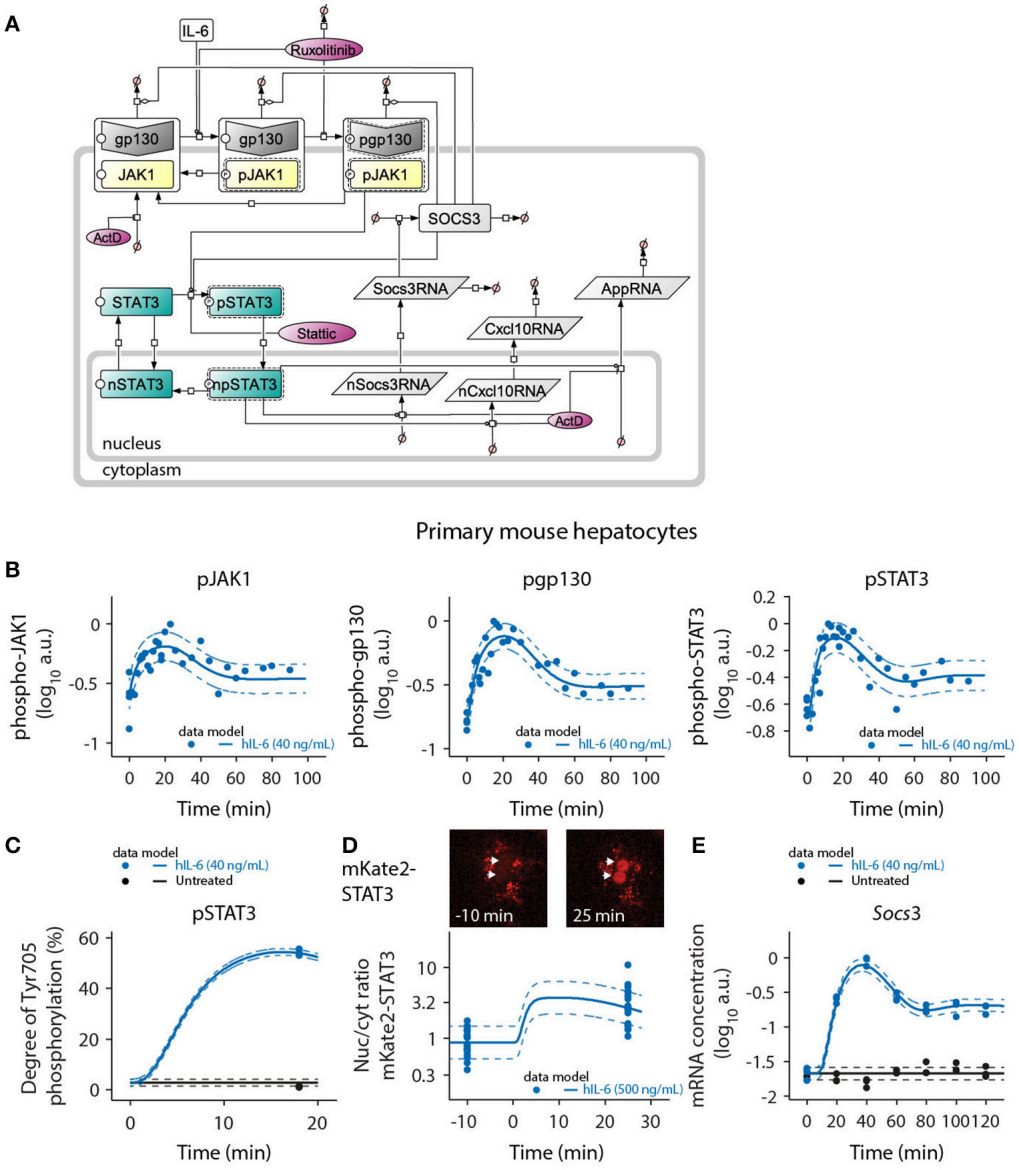
Mathematical model of IL-6-induced JAK1-STAT3 signaling and model calibration with time-resolved signaling data. **(A)** The ODE-based model is represented as process diagram (Kitano et al., 2005). Individual reactions of species (arrows) can be induced (circle-headed lines) or inhibited (bar-headed lines). Dashed line borders highlight active species. AppRNA is representative for the different intermediate/late APP mRNAs *Fgg, Hamp, Il33, Apcs, Hp*, and *Hpx*. The production of cytoplasmic *Socs3* and *APP* mRNAs was modeled using a delay (τ), corresponding to five additional processing steps of intermediate nuclear RNA species. Inhibitors are shown in red color. ActD, actinomycin D; Prefix p, phosphorylated species; Prefix n, nuclear species. **(B)** Primary mouse hepatocytes were treated with 40 ng/mL of hIL-6 and lysed for protein or RNA isolation at indicated time points. Phosphorylated JAK1, gp130, and STAT3 were measured using quantitative immunoblotting preceded by immunoprecipitation to enrich for the target proteins. Recombinant calibrator proteins were used for normalization. **(C)** Primary mouse hepatocytes were treated with 40 ng/mL of hIL-6 for 18 min, lysed and subject to immunoprecipitation. Enriched proteins were separated by SDS-PAGE, in-gel digested, and analyzed by mass spectrometry to determine the degree of Tyr-705 phosphorylation of STAT3. **(D)** Example widefield fluorescent microscopic images of primary hepatocytes from mKate2-STAT3 mice unstimulated (left panel) or stimulated with 500 ng/mL hIL-6 for 25 min (right panel). White arrows indicate positions of nuclei. The ratio of nuclear to cytoplasmic mKate2-STAT3 was determined by live-cell imaging in 20 hepatocytes isolated from mKate2-STAT3 heterozygous mice 10 min prior to and 25 min after stimulation with hIL-6 (500 ng/mL). **(E)** Primary mouse hepatocytes were treated with 40 ng/mL of hIL-6 and lysed for RNA isolation at indicated time points. *Socs3* mRNA was quantified by qRT-PCR (*n* = 3). Filled circles: experimental data; solid lines: model trajectories. Dashed lines indicate the measurement noise as estimated by the error model. a.u., arbitrary units. For additional experimental data used for model calibration see Appendix Figures S26–S81. In total, the model was calibrated with 3090 data points.

To link the observed physiological IL-6 concentrations to activation of signal transduction and induction of target genes and to quantitatively predict the impact of the pathway inhibitors Ruxolitinib and Stattic, we generated a mathematical model of IL-6-induced JAK1-STAT3 signaling in primary mouse hepatocytes (Figure 3A). Assuming the law of mass-action kinetics, we translated the previously established molecular interactions (Heinrich et al., 2003) in response to IL-6 into a set of ODEs. Two compartments were modeled to describe the shuttling of STAT3 between cytoplasm and nucleus, as well as the nuclear export of newly synthesized mRNAs. The model contained four input variables: IL-6, Ruxolitinib, Stattic, and actinomycin D (ActD). Based on immunoassays of human IL-6 in mouse hepatocyte supernatants (Appendix Figure S2), constant IL-6 concentrations and complete removal of ligand after stimulation pulses were assumed. Because Ruxolitinib was reported to have a plasma half-life of ~3 h in humans (Shi et al., 2011), Ruxolitinib degradation was included in the model. Considering that inhibition by Stattic is irreversible, its concentrations were modeled as constant. The temporal evolution of the dynamic variables was described by 25 ODEs, the detailed steps were as follows (Figure 3A): gp130 and JAK1 were described to be pre-associated (Behrmann et al., 2004) and were modeled as one complex JAK1_gp130 with different activation states, as described previously for the interaction of JAK2 with the erythropoietin receptor (Bachmann et al., 2011). The alpha receptor subunit IL-6R was not considered in the model, because it is not directly involved in the dynamic phosphorylation events that initiate IL-6 signaling (Taga et al., 1989). IL-6 promotes activation and phosphorylation of JAK1, causing generation of the species pJAK1_gp130. Additionally, there is also a low level of basal activation. Subsequently, also gp130 is phosphorylated by JAK1 to create the fully activated receptor complex pJAK1_pgp130. Stimulus-independent negative regulatory mechanisms at the receptor level, as reported for JAK1 (Simoncic et al., 2002; Lehmann et al., 2003), were taken into account by including two deactivating steps. Both partly active pJAK1_gp130 and fully active pJAK1_pgp130 are directly converted to inactive JAK1_gp130. This simplification is based on the assumption that dephosphorylation of gp130 and JAK1 is coupled. STAT3 is activated by JAK1 only after docking to phosphorylated gp130 (Lutticken et al., 1994; Stahl et al., 1995; Yamanaka et al., 1996). Thus, double-phosphorylated pJAK1_pgp130 mediates STAT3 activation. The species pSTAT3 represents phosphorylated, active, and dimeric STAT3. A separate dimerization step was neglected, because the oligomerization state of STAT3 was not assessed by experiments. Active, dimeric pSTAT3 subsequently translocates to the nuclear compartment, and npSTAT3 promotes transcription of *Socs3* and APP genes. The generation of cytoplasmic RNA was modeled including a delay (τ; MacDonald, 1976; Bachmann et al., 2011) for both *Socs3* and APP genes to account for processing and nuclear export of these early-induced transcripts. Based on repeated profile likelihood analysis, we concluded that *Socs3, Cxcl10, Fgg, Il33, Hp*, and *Hpx* required an explicit delay in the model to describe the available data, while *Apcs* and *Hamp* did not. *Socs3* was expressed earlier than any of the APP genes. For the APP genes, *Cxcl10* had the shortest delay, followed by *Fgg*. The other APP genes exhibited slower dynamics (see Appendix Figure S24). Cytoplasmic Socs3RNA promotes synthesis of SOCS3. SOCS3 inhibits the signaling pathway by increasing degradation of the receptor complex as well as inhibiting phosphorylation of STAT3 by the fully activated receptor complex (Starr et al., 1997; Babon et al., 2012; Kershaw et al., 2013). Therefore, SOCS3 enhances degradation of all receptor states and inhibits the STAT3-activating reaction converting STAT3 to pSTAT3. Production of APP proteins was not assessed experimentally and was thus not considered in the model. The target RNA and protein species Socs3RNA, SOCS3, and Cxcl10/AppRNA are furthermore subject to degradation.

Deactivation of nuclear STAT3 was suggested to be mediated by phosphatases (Yamamoto et al., 2002). A combined dephosphorylation and dissociation step was therefore modeled in the nuclear compartment, converting dimeric, active npSTAT3 to monomeric, inactive nSTAT3. Based on model identifiability analysis, it was concluded that dephosphorylation and dissociation of STAT3 in the nucleus is very fast. Therefore, npSTAT3 was not considered as a state variable, but modeled proportional to the cytoplasmic concentration of pSTAT3 (see Appendix for more information). STAT3 was shown to continuously shuttle between cytoplasm and nucleus independent of its activation state (Liu et al., 2005; Reich and Liu, 2006). Accordingly, we allowed nuclear import for both, inactive STAT3 and active pSTAT3, while only inactive nSTAT3 can be exported back to the cytoplasm. The previously determined (Mueller et al., 2015) ratio of cytoplasmic to nuclear volume of primary mouse hepatocytes (12.67/0.5 pL, for frequently binucleated hepatocytes) facilitated modeling of concentration changes due to inter-compartmental transport processes.

The pathway inhibitors Ruxolitinib and Stattic were incorporated into the model according to their published molecular modes of action. The JAK inhibitor Ruxolitinib (Lin et al., 2009; Quintas-Cardama et al., 2010) negatively influences JAK1-dependent reactions in the model, specifically the generation of pJAK1_gp130 and pJAK1_pgp130. Stattic blocks activation and dimerization of STAT3 (Schust et al., 2006) and therefore in our model inhibits the respective conversion of STAT3 to pSTAT3. All transcriptional processes are furthermore blocked by ActD.

The protein abundances of the pathway components gp130, JAK1, STAT3, and SOCS3 were determined by quantitative immunoblotting (Schilling et al., 2005a) according to standard curves of recombinant calibrator proteins. The determined number of molecules per cell (Appendix Figure S3) provided the absolute scale for model predictions of those specific states. Remaining unknown model parameters were estimated based on time- and dose-dependent experimental data, as described in the following sections.

### Model calibration with time-resolved signaling and gene expression data

The mathematical model depicted in Figure 3A was calibrated with quantitative experimental data describing the time-resolved dynamics of IL-6-induced JAK1-STAT3 signaling in primary mouse hepatocytes. Cells were treated with hIL-6 for up to 120 min in a continuous or pulsed manner. The levels of phosphorylated and total protein species were measured by quantitative immunoblotting (Schilling et al., 2005a), including randomized sample loading and normalization to suitable housekeeping proteins or, in case proteins were enriched by immunoprecipitation, to recombinant calibrator proteins. hIL-6-induced phosphorylation of gp130, JAK1, and STAT3 was transient displaying a peak at around 20 min. Phospho-gp130, -JAK1, and -STAT3 subsequently declined, but did not reach basal levels within the observed time frame (Figure 3B). Further, SOCS3 protein expression was determined upon treatment with different concentrations of IL-6 (Appendix Figures S43–S45) and we observed a transient protein expression dynamic that resembles the mRNA expression profiles. Using quantitative mass spectrometry (Hahn et al., 2011), we determined the site-specific degree of Tyr-705 phosphorylation of STAT3 at the time point of maximal activation (54.4% at 18 min, 40 ng/mL hIL-6; Figure 3C). We also quantified the nuclear translocation of STAT3 by live-cell imaging of hepatocytes isolated from heterozygous mKate2-STAT3 knock-in mice (Figure 3D and Appendix Figure S11). In mKate2-STAT3 mice, the wild type STAT3 locus is replaced by an mKate2-STAT3 knock-in reporter gene. The expression and phosphorylation of the fusion protein was validated by immunoblotting in primary mouse hepatocytes that were isolated from the knock-in reporter mice and wild-type animals (Appendix Figure S13). Cells were treated with hIL-6 and the phosphorylation of endogenous and tagged-STAT3 was compared. These studies showed that mKate2-STAT3 is expressed at a slightly lower level than the endogenous protein but the phosphorylation dynamics correlated with the phosphorylation dynamics of the endogenous STAT3. The mKate2-STAT3 reporter mice so far have only been obtained as heterozygous mice. For the generation of the mKate2-STAT3 reporter mice we identified seven positive ES clones and out of these two generated germline transmission. The crossing of the heterozygous animals resulted in 66.8% WT and 33.2% heterozygous animals (*n* = 232) and these heterozygous mice showed no phenotype, also concerning viability in comparison to WT animals. Due to the strong autofluorescence in the cytoplasmic compartment of primary mouse hepatocytes (see dot-like structures in Appendix Figure S13C), we focused on the quantification of the IL-6-induced translocation of mKate2-STAT3 to the nucleus. In unstimulated cells, mKate2-STAT3 was equally distributed between cytoplasm and nucleus (STAT3 nuc/cyt ratio of 1), in accordance with the previously reported continuous shuttling of STAT3 independent of its activation state (Liu et al., 2005; Reich and Liu, 2006). We tested continuous shuttling of STAT3 between nucleus and cytoplasm, independent of its activation state in our initial mathematical model. However, based on identifiability analysis (see Appendix Figure S15), we found that export of phosphorylated STAT3 could be made arbitrarily small and was therefore omitted from the final model. Following hIL-6 stimulation, mKate2-STAT3 quickly accumulated in the nucleus. At 25 min (500 ng/mL hIL-6), the nuclear mKate2-STAT3 concentration exceeded cytoplasmic mKate2-STAT3 by a factor of 3 (STAT3 nuc/cyt ratio of ≈3; Figure 3D). *Socs3* mRNA was measured by qRT-PCR, revealing rapid induction after IL-6 stimulation with a peak time of 40 min. Afterwards, *Socs3* mRNA levels declined, but stayed elevated throughout the observed time frame. Background *Socs3* mRNA expression in unstimulated hepatocytes did not change over time, indicating a specific response (Figure 3E). The trajectories of the calibrated model accurately represented the experimental data describing multiple levels of IL-6-induced signaling (solid lines in Figures 3B–E and Appendix Figure S9).

**Figure 4:**
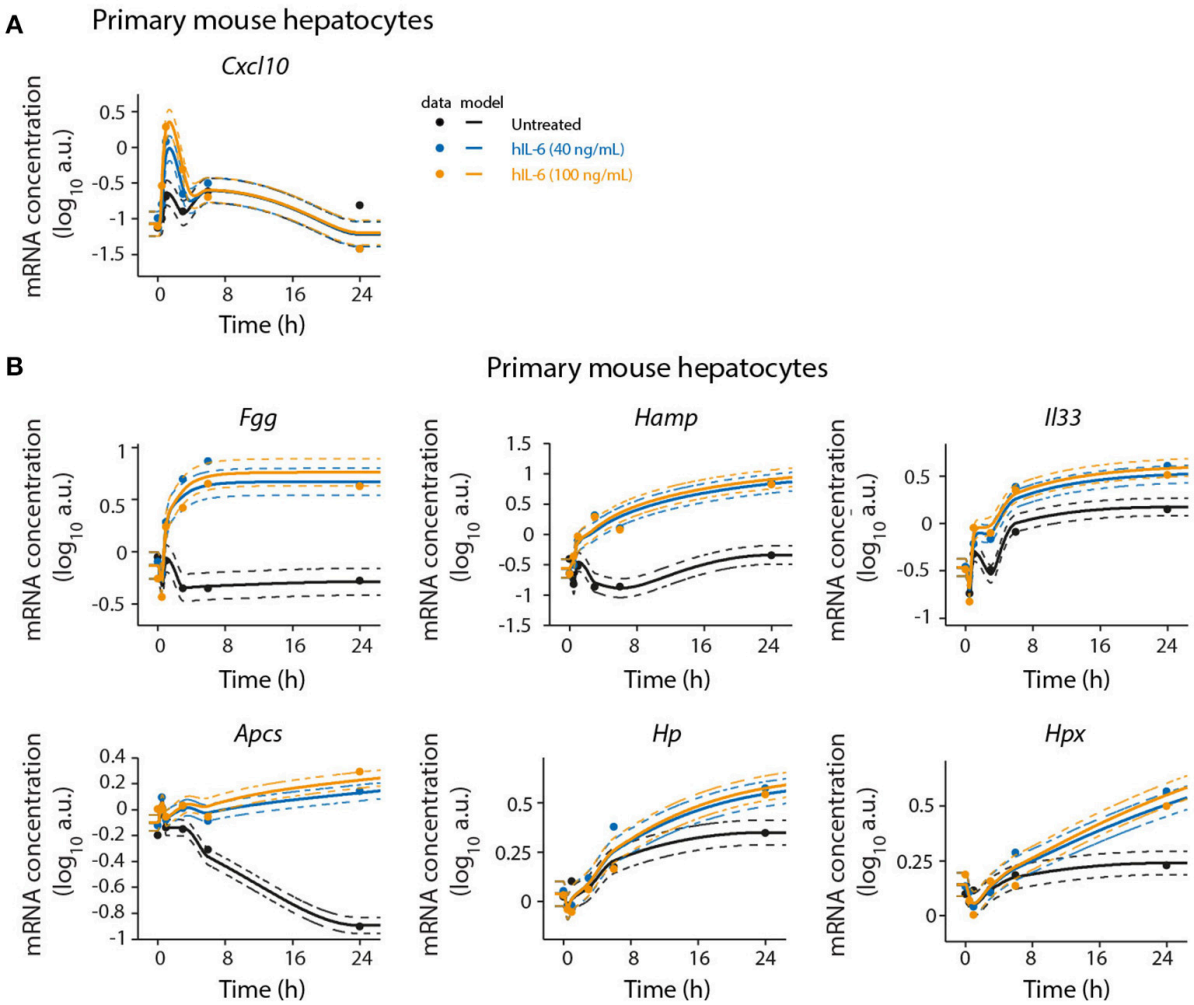
Detailed time-resolved analysis of selected IL-6 target genes. Primary mouse hepatocytes were stimulated with hIL-6 or left untreated and RNA was isolated at indicated time points. Target mRNA expression was analyzed by qRT-PCR (qPCR) (**A** *Cxcl10*; **B** *Fgg, Hamp, Il33, Apcs, Hp*, and *Hpx*). Data were normalized to the geometric mean (Vandesompele et al., 2002) of *Hprt* and *Tbp* expression. Filled circles represent individual replicates (*n* = 2); solid lines are model fits; dashed lines indicate errors estimated from the model. The expression level in the control is modeled using a monotonic spline. Additional replicate is displayed in Appendix Figure S10.

To validate the microarray analysis and to obtain detailed time-resolved expression profiles, we analyzed selected significantly regulated genes by quantitative real-time quantitative PCR (qRT-PCR) in a time-resolved manner. In agreement with the microarray analysis, we found *Cxcl10* and *Socs3* to be early-response genes. *Cxcl10* was transiently induced by hIL-6. After an initial peak at 1 h *Cxcl10* expression levels decreased below those observed in untreated cells. *Socs3* expression showed a sharp peak with high amplitude at 1 h of hIL-6 treatment. Subsequently, its levels declined but stayed elevated up to 24 h. This is consistent with our microarray analysis, which identified *Socs3* to critically contribute to overall regulation (Figure 2B) and to be significantly IL-6-induced at all time points (Figure 2C). The genes *Fgg, Hamp*, and *Il33* were induced by IL-6 and were clearly detectable from 3 to 6 h on, as shown by qRT-PCR analysis. All three genes showed sustained activation with high expression levels up to 24 h of IL-6 treatment, thus validating our microarray analysis which identified *Fgg, Hamp*, and *Il33* to be significantly regulated at intermediate and late time points. *Apcs* was found to be a late-regulated gene. qPCR-based validation revealed a steady decrease of *Apcs* expression in untreated cells. IL-6 treatment rescued this decrease and caused elevated *Apcs* expression at 24 h relative to untreated cells (Figure 4 and Appendix Figure S10). The APP genes *Hp* and *Hpx* were not significantly regulated in our microarray analysis (Figure 2C), but have previously been reported to be IL-6 responsive and STAT3-dependent (Alonzi et al., 2001). qPCR analysis identified *Hp* and *Hpx* to be late-response genes with increased expression at 24 and 48 h of IL-6 treatment (Figure 4 and Appendix Figure S10).

### Model calibration with dose-dependent target gene expression data from normal and perturbed conditions

**Figure 5:**
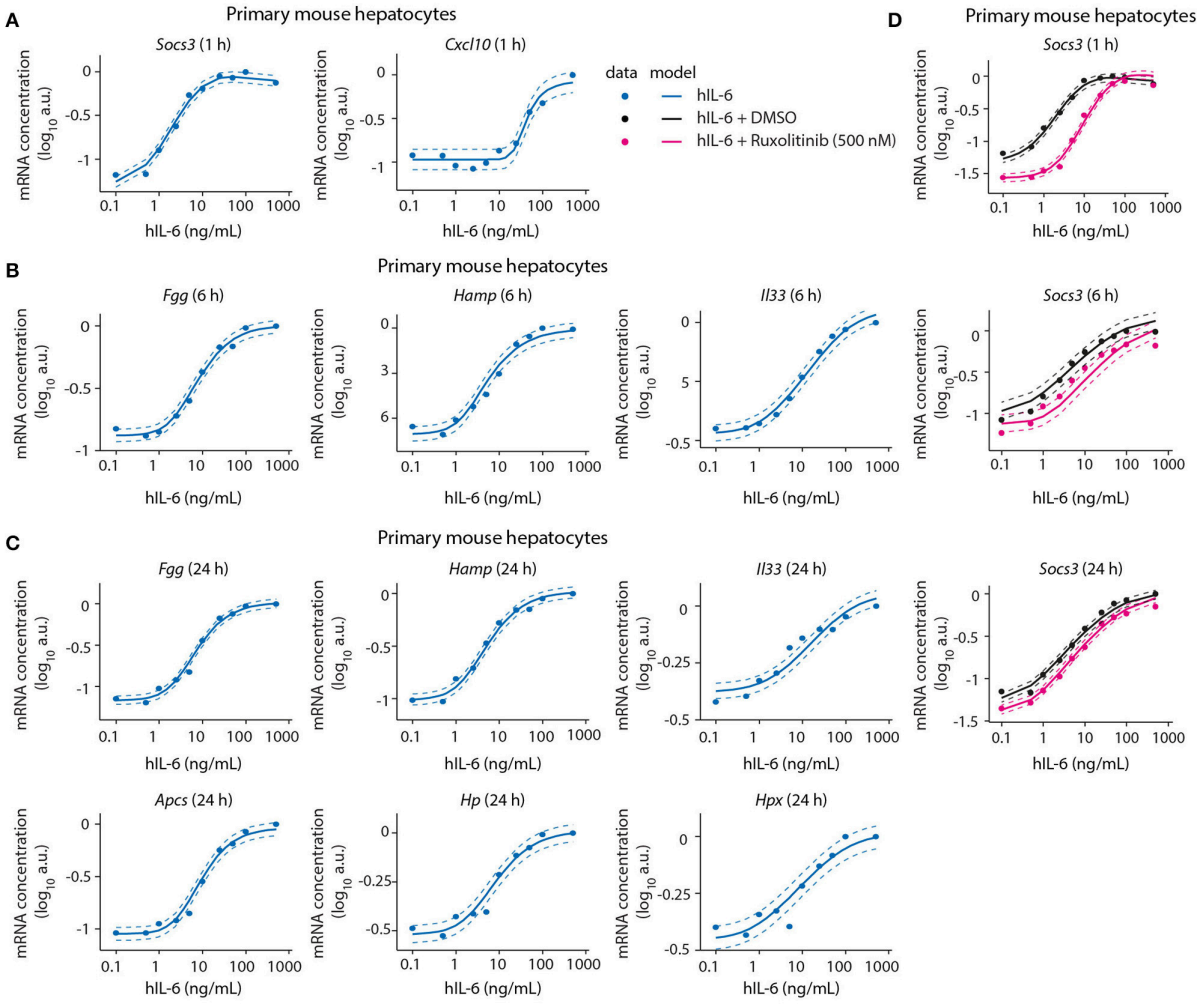
Model calibration with dose-dependent target gene expression data from normal and perturbed conditions. Primary mouse hepatocytes were pre-treated with Ruxolitinib or DMSO control for 1 h prior to hIL-6 stimulation, or left untreated. Dose-dependent analysis of target gene expression was performed by applying 0.1–500 ng/mL hIL-6. At indicated time points (1, 6, and 24 h), target mRNAs were quantified by qPCR (**A** *Socs3* and *Cxcl10*; **B** *Fgg, Hamp*, and *Il33*; **C** *Fgg, Hamp, Il33, Apcs, Hp*, and *Hpx*; **D** *Socs3*). Filled circles: log-transformed experimental data; solid lines: model trajectories. Dashed lines indicate the measurement noise as estimated by the error model. a.u., arbitrary units. For additional replicates used for model calibration see Appendix Figure S4.

In addition to time-resolved data, we calibrated the mathematical model with dose-dependent expression data for the IL-6 target genes shown in Figure 5 and Appendix Figures S4, S5. Primary mouse hepatocytes were treated with a wide range of hIL-6 concentrations covering basal, regenerative, and inflammatory physiological levels (Figure 1) for 1, 6, or 24 h to capture strong expression of early, intermediate, and late responsive genes, respectively. Based on the experimental data, we identified the nuclear dephosphorylation rate to be high. Since nuclear STAT3 dephosphorylation is so rapid that the level of nuclear phosphorylated STAT3 exactly follows the cytoplasmic concentration of phospho-STAT3, the model was reduced by one equation.

We further identified *Socs3* mRNA to respond to IL-6 treatment in a highly sensitive manner (Figure 5A). The other early-induced gene *Cxcl10* responded at 25–50 ng/mL hIL-6, and did not reach saturation within the observed hIL-6 range. Compared with *Socs3*, it thus showed lower sensitivity toward hIL-6 (Figure 5A). In contrast, the sensitivities of intermediate and late APP genes were similar to that of *Socs3*—*Fgg, Hamp, Il33, Apcs, Hp*, and *Hpx* mRNAs were induced from 1 to 10 ng/mL hIL-6. All showed sigmoidal dose response curves and saturation at high IL-6 concentrations (100–500 ng/mL). The dose-dependent behavior of all target genes was accurately described by the model (Figures 5A–C and Appendix Figure S4).

We also calibrated our model with experimental data describing the impact of the two pathway inhibitors Ruxolitinib and Stattic on dose/time-dependent *Socs3* mRNA induction and STAT3 phosphorylation dynamics in primary mouse hepatocytes (Figure 5D). The inhibitor Stattic shows toxic effects and is not used in the clinic. In our experiments we applied Stattic for a maximum of 2 h to primary mouse hepatocytes to avoid general toxicity. Though Stattic was used to calibrate the model, experimental results with this inhibitor are only shown in the Appendix (Appendix Figures S9, S46, S52–S53, S62–S69, S83–S84, S89).

Inhibitor pre-treatment for 1 h caused a reduced basal level of *Socs3* mRNA and a reduced sensitivity and peak magnitude of the dose-dependent *Socs3* response upon IL-6 stimulation (Figure 5). *Socs3* expression at 1, 6, and 24 h was detectable from 1 ng/mL hIL-6, steadily increased, and reached saturation at 50 ng/mL hIL-6. In line with the previous observation in a clinical trial (Shi et al., 2011), the efficacy of Ruxolitinib decreased with increasing incubation time. The experimental data of *Socs3* expression in response to hIL-6 alone or hIL-6 and Ruxolitinib was described by the model trajectories (Figure 5D).

To summarize, the model was calibrated in two stages. First the upstream model of IL-6 signaling was developed and calibrated. The upstream model also termed “core model” consists of the receptor level, the STAT3 pools and SOCS3 and is calibrated on both wild type as well as inhibitor data. The downstream model, which consists of the APP genes, was included in a second step. Since none of the APP genes feed back into the system, these were parameterized separately to keep the analyses computationally tractable. The downstream model was parameterized while keeping the upstream model parameters fixed. Parameter profile likelihood curves for all APP genes are presented in the supplement (Appendix Figures S15–S22).

### Designing improved ruxolitinib treatment schedules

**Figure 6:**
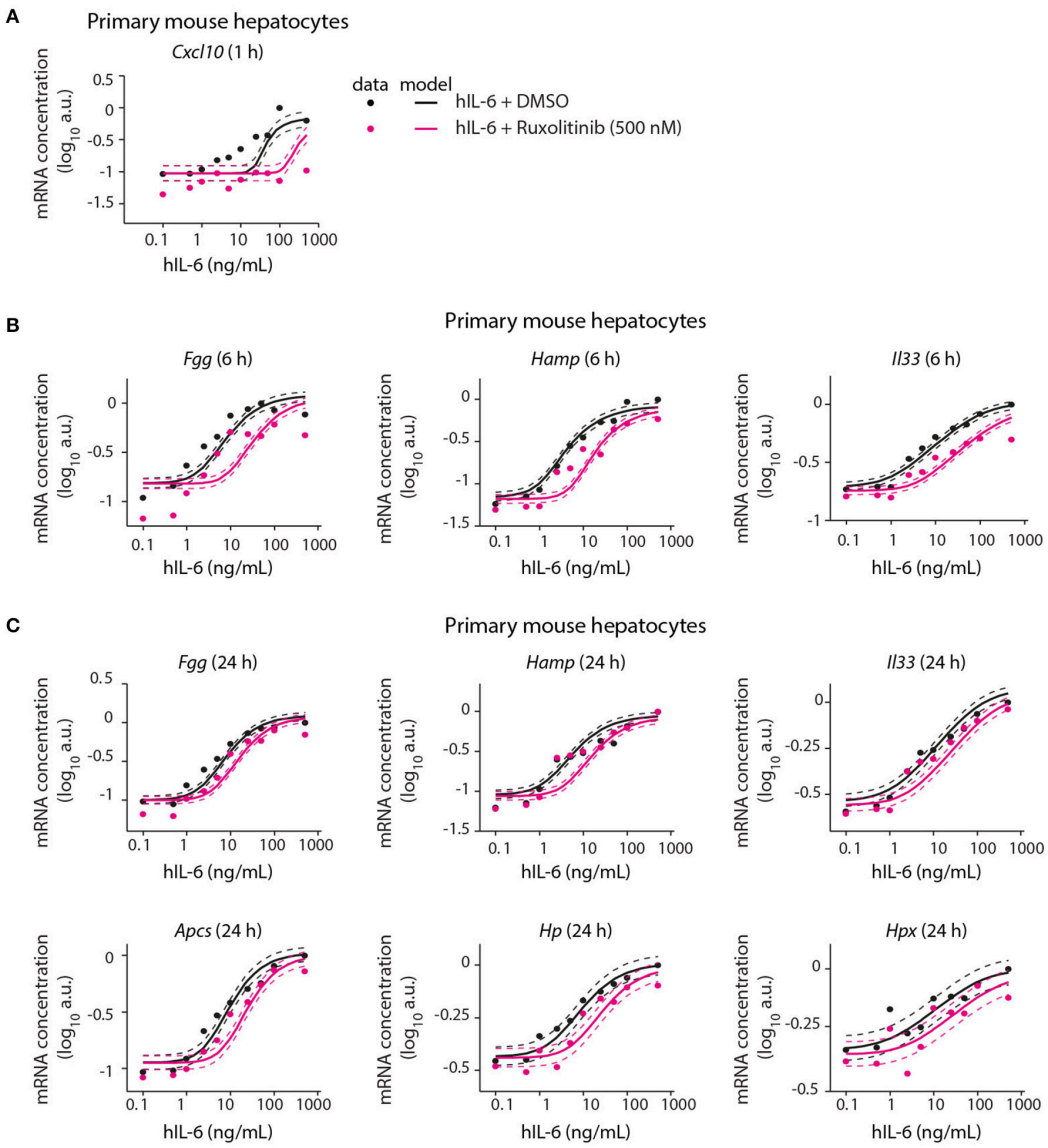
Model prediction and experimental validation of the inhibitor impact on dose-dependent APP gene expression. Solid lines represent model predictions for dose-dependent APP gene expression with or without Ruxolitinib pre-treatment. For experimental validation (filled circles), primary mouse hepatocytes were pre-treated with Ruxolitinib or DMSO control for 1 h prior to hIL-6 (0.1–500 ng/mL) stimulation. APP mRNA expression was quantified at indicated time points by qPCR (**A** *Cxcl10*; **B** *Fgg, Hamp*, and *Il33*; **C** *Fgg, Hamp, Il33, Apcs, Hp*, and *Hpx*). Dashed lines indicate the measurement noise as estimated by the error model. a.u, arbitrary units. For additional replicates see Appendix Figure S5. For additional experimental data used for model validation see Appendix Figures S82–S88.

Following calibration with quantitative experimental data, our mathematical model was able to describe IL-6-induced signaling responses at multiple levels, including the impact of pathway inhibitors on STAT3 (Appendix Figure S9A) and *Socs3* activation (Figure 5). We next employed the model to predict the inhibitor impact on hIL-6 dose-dependent APP gene expression in murine hepatocytes. In analogy to the inhibition of *Socs3* mRNA induction (Figure 5D), the model predicted that Ruxolitinb treatment reduces sensitivity of the response for most APP genes (Figure 6). Importantly, subsequent experimental analysis validated the model-predicted effects of Ruxolitinib treatment on all analyzed APP genes (Figure 6 and Appendix Figure S5). As observed previously in the case of *Socs3* mRNA expression at 1, 6, and 24 h (Figure 5D), the long-term efficacy of Ruxolitinb in primary mouse hepatocytes was reduced in the case of intermediate/late APP genes (Figures 6B,C and Appendix Figures S5B,C).

**Figure 7:**
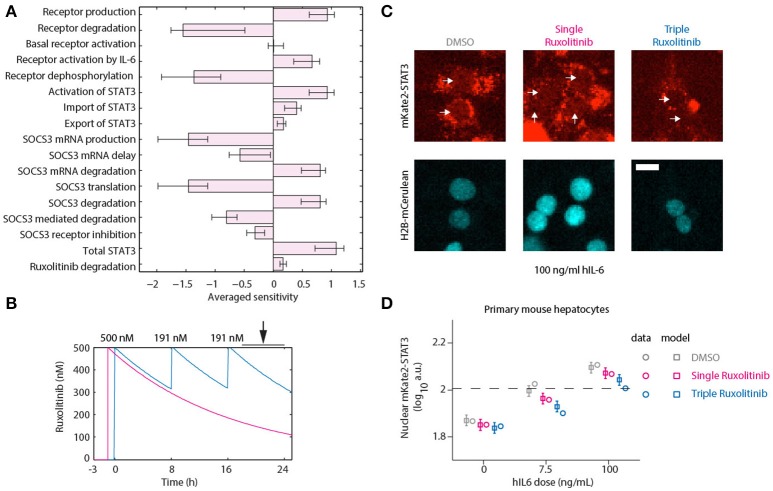
Optimized inhibition of STAT3 nuclear translocation. **(A)** Averaged Local Parameter Sensitivity Analysis for the integrated APP response. Bars indicate model sensitivities to a 50% perturbation in model parameter. Error bars indicate maximum and minimum sensitivity along parameter likelihood profiles. Large perturbations were performed since we are interested in parameters that would have a substantial impact on dynamics. **(B)** Model predictions for Ruxolitinib concentrations over time after single treatment with 500 nM at *t* = –1 h (Single), or using the optimized triple treatment scenario, including Ruxolitinib treatments at *t* = 0 h (500 nM), 8 h (191 nM), and 16 h (191 nM) (Triple). Arrow indicates the time window of nuclear STAT3 profiling. **(C)** Example widefield fluorescent microscopic images of primary hepatocytes from mKate2-STAT3 mice during the long-term quantification of IL-6-induced mKate2-STAT3 translocation in presence or absence of Ruxolitinib. Cells were stimulated with 100 ng/ml hIL-6 for 24 h, either treated with solvent control (DMSO), pre-treated for 1 h with 500 nM Ruxolitinib (Single Ruxolitinib) or co-treated with 500 nM Ruxolitinib and re-treated with 191 nM at 8 and 16 h (Triple Ruxolitinib). Inhibitor treatment was performed as suggested by the model **(B)**. Image quantification of nuclear mKate2-STAT3 was conducted from 20 to 24 h after hIL-6 stimulation. White arrows indicate positions of nuclei. H2B-mCerulean was used to indicate the positions of nuclei. Scale bar: 20 μm. **(D)** Squares represent model predictions for nuclear STAT3 after treatment with the indicated hIL-6 concentrations in combination with DMSO control, single or triple Ruxolitinib treatment. For experimental validation, primary mouse hepatocytes from mKate2-STAT3 mice were treated accordingly with DMSO or Ruxolitinib and hIL-6. Circles represent average nuclear STAT3 measured with time-lapse microscopy 20–24 h after hIL-6 stimulation. Error bars indicate the measurement noise as estimated by the error model. a.u., arbitrary units. Data presented corresponds to the average of at least 45 imaging fields per condition. For additional replicates see Appendix Figure S6. For additional experimental data used for model validation see Appendix Figures S93–S98.

To assess the suitability of different targets in reducing the APP response, we performed a sensitivity analysis. If one considers the APPs to be very stable, then the protein levels will approximately be proportional to the integral of the expression of the APP genes. We performed a Local Parameter Sensitivity Analysis with respect to the model parameters, which is shown in Figure 7A. Here we can observe that inhibiting production and activation of the receptor, inhibiting the activation of STAT3 and reducing the degradation of *Socs3* (mRNA) are all predicted to lead to additional attenuation of the APP response. To further inhibit STAT3 activation, we decided to apply additional doses of Ruxolitinib.

**Figure 8:**
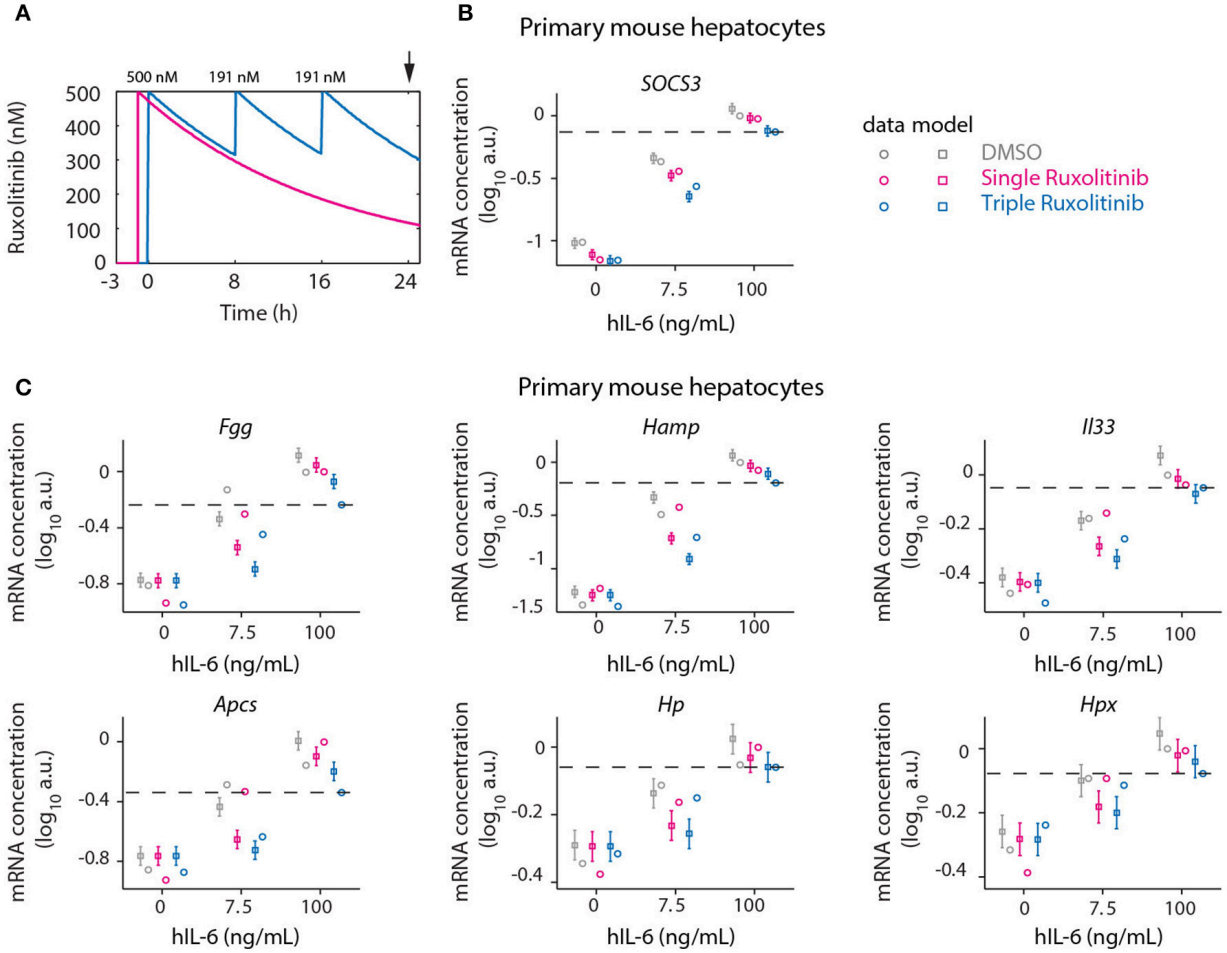
Optimized inhibition of APP gene expression in primary mouse hepatocytes. **(A)** Model predictions for Ruxolitinib concentrations over time after single treatment with 500 nM at *t* = –1 h (Single), or using the optimized triple treatment scenario, including Ruxolitinib treatments at *t* = 0 h (500 nM), 8 h (191 nM), and 16 h (191 nM) (Triple). Arrow indicates the time point of gene expression analysis. **(B,C)** Expression of *Socs3* **(B)** and APP genes **(C)** at 24 h after inhibitor treatments as described in **(A)**. Squares represent model predictions and circles represents experimental data, while error bars indicate the measurement noise estimated by the error model. a.u., arbitrary units. Dashed lines indicate the level of gene expression after triple inhibitor dosing of the cells treated with inflammatory dose of hIL-6 (100 ng/mL). Displayed are results of one biological replicate, while two more replicates are shown in Appendix Figure S7. For additional experimental data used for model validation see Appendix Figures S89–S91.

Continuous suppression of elevated IL-6-induced APP gene expression would be required to counteract inappropriate inflammatory responses, but Ruxolitinib-mediated reduction of hIL-6 target gene expression in murine hepatocytes was less effective at advanced time points (Figures 5, 6). The model predicted that higher single doses of Ruxolitinib would lead to larger suppression of the APP genes. However, since higher doses of the inhibitor could have detrimental side-effects, we employed our mathematical model to design treatment schedules for Ruxolitinib where the concentration of Ruxolitinib in the system does not exceed a maximal dose. The aim was to continuously suppress elevated IL-6-induced APP gene expression, while not exceeding a maximal level of 500 ng/mL Ruxolitinib. As objective, the integral up to 24 h of the APP mRNA levels in response to 100 ng/mL hIL-6 was used as a proxy for APP expression during inflammation. In this way, inappropriate inflammatory responses could be counteracted without completely abrogating APP gene expression. Ideally, continuous administration of Ruxolitinib would be preferred. However, due to practical considerations, we restricted the search to a maximum of three injections. The model predicted which three Ruxolitinib doses in even time intervals would effectively counteract loss of the inhibitor due to degradation (Figures 7B, 8A).

Therefore, in the presented study, the objective was to minimize the integral of APP mRNA levels. However, we did not use an optimization procedure as a means of determining the treatment schedule, because it is not clear how to prioritize the different APP genes. Instead we made response curves for each of the APP genes and determined the ideal point via visual inspection. Depending on which APP gene is considered therapeutically most important, deviations from this design may be more optimal (see section 3.7 of the Appendix). Predictions for the integrated target gene expression at time point 24 h furthermore revealed that applying the first treatment at *t* = 0 h, simultaneously with the start of IL-6 treatment, would be superior to the previously applied pre-treatment with Ruxolitinib at 1 h before IL-6 stimulation (Appendix Figure S8). Thus, an optimized Ruxolitinib treatment would include three subsequent treatments at *t* = 0, 8, and 16 h. Given that 500 nM Ruxolitinib would be applied as first bolus at *t* = 0 h, the model predicted that 191 nM Ruxolitinib would be required to replenish the full inhibitor potential at each, 8 h, and 16 h. The initial dose of 500 nM Ruxolitinib was selected based on dose response experiments (Appendix Figures S70, S71) in primary mouse hepatocytes and closely relates to the determined IC_50_.

Using two experimental readouts, namely nuclear translocation of STAT3 as an indicator of activated STAT3, and IL-6 target gene expression, we validated the predicted advantage of Ruxolitinib triple treatment over the previously applied single pre-treatment (Figures 7, 8). Compared to the single treatment (q24h), the triple treatment (q8h) induced a more sustained inhibitory effect, utilizing considerably lower doses for the repetitive treatment after the initial bolus. Primary mouse hepatocytes were either treated with a single dose (500 nM) of Ruxolitinib at *t* = –1 h (Single), or with three doses at time points *t* = 0 h (500 nM), 8 h (191 nM), and 16 h (191 nM; Triple). Cells were stimulated with hIL-6 concentrations resembling basal (0 ng/mL), regenerative (7.5 ng/mL) or inflammatory (100 ng/mL) physiological levels (Figure 1). In hepatocytes derived from mKate2-STAT3 mice, we analyzed the nuclear mKate2-STAT3 concentration within the time frame 20–24 h (after start of IL-6 treatment) by live-cell imaging (Figure 7C). Comparing model predictions and experimental data for the different Ruxolitinib treatment regimens and IL-6 concentrations revealed good agreement between model and experiment (Figure 7D and Appendix Figure S6): Ruxolitinib-mediated suppression of mKate2-STAT3 nuclear translocation was improved when the triple treatment regime was applied, compared with single treatment. In wild type hepatocytes we measured APP gene expression at 24 h. Triple Ruxolitinib treatment lead to improved suppression of most genes, compared with single Ruxolitinib treatment (Figure 8 and Appendix Figure S7). The effect was most obvious for *Socs3*, and also recognizable for all other genes, although error bars were partly overlapping for single and triple treatment. Importantly, triple Ruxolitinib treatment reduced gene expression observed at inflammatory IL-6 concentrations (100 ng/mL) to levels more closely resembling regenerative conditions (7.5 ng/mL, DMSO control) for all genes (Figure 8 and Appendix Figure S7). To conclude, our mathematical model and experimental validation suggested that a triple treatment with Ruxolitinib and not a single dose is required, when an effective attenuation of IL-6-dependent responses in hepatocytes is desired.

To provide a proof-of-concept that these insights, obtained with our model based approach for primary mouse hepatocytes, are applicable to the human system, we employed primary human hepatocytes to compare a single bolus treatment with the model-suggested triple dosing strategy. To mimic the regenerative and inflammatory situation in the human system, 1.8 and 50 ng/mL hIL-6 were chosen to stimulate human hepatocytes, assuming that the potency of human IL-6 on human hepatocytes is comparable to that of mouse IL-6 on primary mouse hepatocytes and by utilizing the dose response curves shown in Figure 1D.

**Figure 9:**
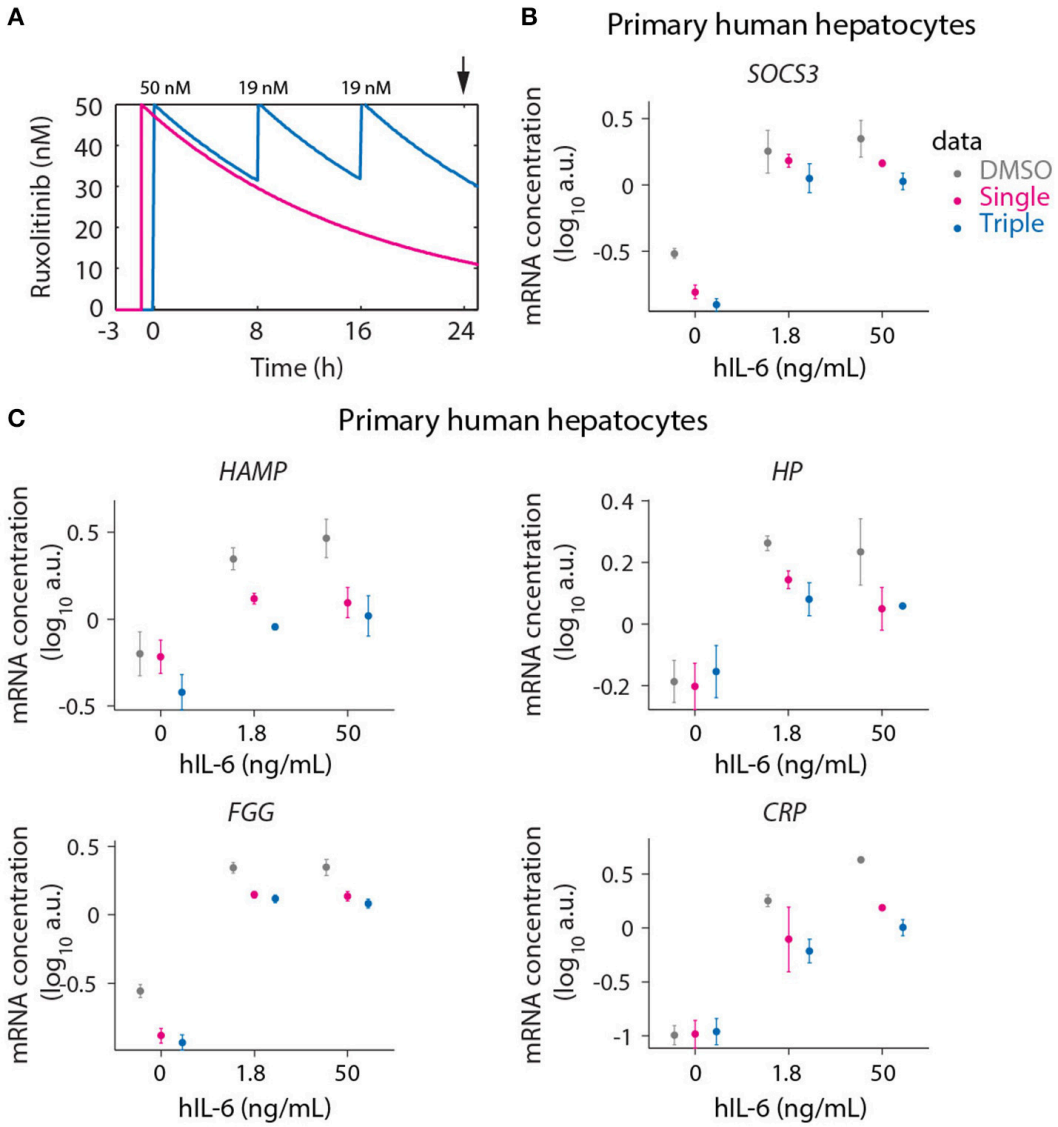
Optimized inhibition of APP gene expression in primary human hepatocytes. **(A)** Model predictions for Ruxolitinib concentrations over time after single treatment with 50 nM at *t* = –1 h (Single), or using the optimized triple treatment scenario, including Ruxolitinib treatments at *t* = 0 h (50 nM), 8 h (19 nM), and 16 h (19 nM) (Triple). Arrow indicates the time point of gene expression analysis. **(B,C)** Expression of *SOCS3* **(B)** and APP genes **(C)** at 24 h after inhibitor treatments as described in **(A)**. Data from primary human hepatocytes of three different donors are shown as mean ± SEM.

The maximal tolerable dose of Ruxolitinib in healthy volunteers was described to be 100 mg once daily, already inducing severe side effects as neutropenia (Shi et al., 2011). This amount corresponds to a concentration of 65 nM assuming an average blood volume of 5 L per human body. The dose-dependent effect of Ruxolitinib on STAT3 phosphorylation in primary human hepatocytes revealed that 50 nM of the inhibitor is in the range of the IC_50_ determined in cells co-treated with hIL-6 (1 or 10 ng/mL) and increasing doses of Ruxolitinib (up to 5,000 nM; Appendix Figure 12). Hence, primary human hepatocytes show an increased sensitivity toward the treatment with Ruxolitinib in comparison to primary mouse hepatocytes. Therefore, we reduced the initial dose of Ruxolitinib from 500 nM as applied in primary mouse hepatocytes to 50 nM for primary human hepatocytes (Figure 9A) and the subsequent second and third dosing of 191 nM Ruxolitinib was reduced to 19 nM accordingly.

We measured the expression of the four previously analyzed genes *SOCS3* (Figure 9B), *HAMP, HP*, and *FGG* (Figure 9C), which are established as IL-6 responsive genes both in mouse and human. Additionally the IL-6 induced expression of *CRP* (Figure 9C) was examined in primary human hepatocytes due to its routine clinical determination as an indicator of inflammatory responses. For several of the genes of interest almost maximal expression was already achieved with the lower hIL-6 concentration applied suggesting that their expression saturated at lower IL-6 doses in human hepatocytes compared to murine hepatocytes. In line with the model-based insights, the triple Ruxolitinib treatment at equivalent time intervals was again more effective compared to the single treatment to suppress IL-6 induced *SOCS3* and APP gene expression in primary human hepatocytes, confirming our concept.

## Discussion

While IL-6 has repeatedly been suggested to contribute to inflammatory or malignant diseases, targeting this central mediator needs to be carefully evaluated to maintain its beneficial regenerative functions (Hunter and Jones, 2015). Here we developed a mathematical model of IL-6-induced JAK1-STAT3 signaling in primary mouse hepatocytes, which adequately predicted how inflammatory gene expression could be reduced to regenerative levels by optimized Ruxolitinib treatment.

Determination of *in vivo* circulating and the IL-6 concentrations during liver regeneration and inflammation enabled to study IL-6 signaling pathway activation within relevant IL-6 concentration ranges. Determined serum levels agree with previously reported values after PHx (1–2 ng/mL; Nechemia-Arbely et al., 2011; Yin et al., 2011) and LPS treatment (175 ng/mL; Piao et al., 2013) of mice. Importantly, simultaneous analysis of samples from PHx- and LPS-treated mice, as performed here, enabled direct comparison of the regenerative and inflammatory scenarios. Thus, we established distinct IL-6 concentration ranges during liver regeneration and inflammation. By quantifying the hepatic IL-6 concentrations, we provide evidence that IL-6 accumulates in the hepatocyte microenvironment after PHx (serum: 1.4 ng/mL, local: 6.8–7.9 ng/mL). This is likely a result of an increased IL-6 secretion by Kupffer cells (Aldeguer et al., 2002) to promote an efficient regenerative response. In contrast, IL-6 levels were similar in serum and hepatocyte microenvironment after LPS injection (serum: 201.8 ng/mL, local: 28.1–500 ng/mL). Local hepatic IL-6 levels following PHx in liver tissues at mRNA and protein level were reported previously (Yin et al., 2011). However, the published IL-6 levels represent both, extra- and intracellular IL-6, and are thus not directly equivalent to IL-6 levels that actively stimulate hepatocytes. Here we inferred the IL-6 concentrations from STAT3 phosphorylation levels in whole liver lysates. Because IL-6 appears to be the main inducer of STAT3 activation in hepatocytes (Cressman et al., 1996), the major hepatic cell type, this approach provides a good estimate of IL-6 concentrations in the hepatocytes' microenvironment.

To exclude the contribution of other cytokines to the activation of STAT3, we performed a qPCR analysis of *Il11, Osm*, and *Il22* mRNA expression in liver lysates from LPS or PHx mice (Appendix Figure S14). In these experiments we did not observe an elevation of the mRNAs encoding these cytokines within the time frame of maximal STAT3 phosphorylation detected in the liver lysates from the corresponding mice, at 1 h in LPS treated mice and at 2 h in hepatectomized mice, respectively (Figure 1B). On the contrary a very rapid induction of *Il6* mRNA was detected particularly in response to LPS injection that was already maximal after 1 h of LPS injection (Appendix Figure S14) and coincided with maximal STAT3 phosphorylation observed at 1 h post treatment (Figure 1B). These results are in line with the studies of Ren et al. reporting that there is no statistically significant increase of *Il22* mRNA in response to hepatectomy, but rather an increase of IL-22 protein is observed in the serum at late time points post hepatectomy starting at 6 h post treatment with a peak at 12 h (Ren et al., 2010). Others observed a peak of *Il22* mRNA induction in the liver at ~3 h post hepatectomy (Rao et al., 2014) or 4 h after LPS injection (Wegenka et al., 2007) and thus much later than the rapid maximal phosphorylation of STAT3 we observed in our study in the liver of hepatectomized or LPS treated mice. Furthermore, 4 h post LPS injection peak levels of IL-22 were detected by ELISA measurements corresponding to ~600 pg/mL (Dumoutier et al., 2011) whereas, in agreement with the study by Wegenka et al., we observed already at 2 h post LPS injection a peak concentration of IL-6 in the serum of 201.8 ng/mL suggesting that the induction of IL-6 in response to LPS is more rapid and more than two orders of magnitude higher compared to IL-22. Further, a major contribution of IL-10 to the early activation of STAT3 and the induction of the acute phase response in hepatocytes appears unlikely. Although it has been observed that the expression of *Il10* mRNA can be induced by PHx (Yin et al., 2011), the ability of IL-10 to induce signaling via JAK1/STAT3 appears primarily restricted to cells of the immune system, such as macrophages and dendritic cells, due to the expression of the IL-10 receptor that is most prominent in these cell types (Murray, 2006; Sabat et al., 2010). In light of these observations we propose that IL-6 is the mediator of immediate early responses in hepatocytes involving STAT3 phosphorylation during liver regeneration whereas other cytokines such as IL-22 or IL-10 may contribute to STAT3 phosphorylation at later time points or in other cell types than hepatocytes.

Because the hepatic acute phase response is largely regulated at the transcriptional level (Andus et al., 1988; Heinrich et al., 1990), we studied IL-6-induced mRNA expression changes in primary mouse hepatocytes. We established the time-dependent regulation of previously known IL-6 targets including *Socs3* (Starr et al., 1997), *Hamp* (Wrighting and Andrews, 2006; Pietrangelo et al., 2007), *Fgg, Apcs, Hp, Hpx* (Alonzi et al., 2001) as well as of two less well-established targets, *Cxcl10* and *Il33*. IL-6-induced genes were grouped into early, intermediate, and late responsive genes, according to their expression levels at 1, 6, and 24 h after IL-6 stimulation. CXCL10 was described previously to be secreted by macrophages in an IL-6/STAT3-dependent manner (Xu et al., 2012, 2015). In the context of hepatitis C virus infection, CXCL10 was suggested to contribute to persistent liver inflammation and fibrosis (Zeremski et al., 2008, 2009; Brownell and Polyak, 2013). Here, we provide evidence that IL-6 stimulated hepatocytes might be a crucial source for CXCL10. In line with the reports by Zeremski et al. (2008, 2009) and Brownell and Polyak (2013), linking CXCL10 and inflammation, *Cxcl10* mRNA was selectively induced by inflammatory IL-6 concentrations, while all other analyzed target genes responded to both, regenerative and inflammatory IL-6 stimuli (Figure 5). In addition to *Cxcl10*, we identified *Il33* to be expressed in response to IL-6 in primary mouse hepatocytes. This IL-1-like cytokine (Schmitz et al., 2005) was described to be induced in fibrotic livers, and hepatic stellate cells were suggested as major IL-33 producer in this context (Marvie et al., 2010). Our results indicate that also hepatocytes might produce IL-33. We observed strong and sustained induction of *Il33* mRNA (Figure 4), which together with its suggested role of IL-33 as a general alarm signal (Miller, 2011), highlight this gene as an interesting IL-6 target and APP gene in hepatocytes. Both, CXCL10 and IL-33 secretion by hepatocytes should be investigated in the future to better understand the potential link between IL-6 and chronic inflammation. By acting on T-cells and innate immune cells (Miller, 2011; Brownell and Polyak, 2013), CXCL10 and IL-33 might contribute to the amplification of inflammatory responses.

Signal processing in hepatocytes translates extracellular IL-6 levels to JAK1-STAT3 signaling dynamics and to changes in gene expression. We could quantitatively link these different levels by implementing a mathematical model, which not only described experimentally observed signaling dynamics, but also the impact of the pathway inhibitors Ruxolitinib and Stattic on STAT3 and its target gene activation. While previous mathematical models of IL-6 signaling incorporated IL-6-induced JAK-STAT as well as mitogen-activated protein kinase (MAPK) cascades (Singh et al., 2006; Moya et al., 2011; Xu et al., 2015), we focused our model scope on the JAK-STAT pathway, which according to Dierssen et al. is relevant for IL-6-induced APP expression whereas MAPK activation is dispensable (Dierssen et al., 2008). To obtain a predictive mathematical model, the relation of considered species and experimentally measured components as well as the amount of experimental data needs to be appropriate (Aldridge et al., 2006; Bachmann et al., 2012). Compared to the earlier approaches (Singh et al., 2006; Moya et al., 2011; Xu et al., 2015) that partly relied on literature derived parameter values obtained from different cell types and stimulating agents, the amount of experimental data used for calibration and validation of the model presented here is more extensive. As an example, we assessed the protein concentration of key players of the signal transduction pathway in primary mouse hepatocytes. The obtained value for STAT3 is in good agreement with recently published data from a mass spectrometry approach to determine molecules per cell in primary human hepatocytes (Wisniewski et al., 2016). For the presented study an extensive amount of experimental data was generated using different technologies ranging from quantitative immunoblotting, multiplexed bead-based arrays and quantitative mass spectrometry to qRT- PCR and microarray analysis in order to assess the dynamics of signal transduction and target gene expression. Whereas quantitative immunoblotting, multiplexed bead-based arrays, and qRT-PCR permit very detailed time-resolved analysis, omics technologies will be increasingly employed for quantitative analysis and to facilitate the link to primary patient material (Iwamoto et al., 2016; Adlung et al., 2017).

The nuclear translocation of STAT3 is a crucial aspect of signal transduction in response to IL-6 stimulation. Therefore the determination of the spatial dynamics of STAT3 and inclusion in the mathematical model are of importance. To quantitatively assess this behavior in the context of primary hepatocytes expressing endogenous amounts of fluorescently labeled STAT3, we generated the mKate2-STAT3 reporter mouse line. Since the fluorescently labeled STAT3 was created as knock-in into the endogenous STAT3 locus, its expression should mirror the expression of STAT3 in different organs. Therefore, the reporter mouse model offers a wide range of possible applications to study STAT3 in multiple organs and could especially be useful to track the dynamics of STAT3 at the single cell level.

We established a mathematical representation of the signaling network and could confirm its high predictive power by experimentally validating previously untested scenarios. Specifically, a model-predicted treatment with a high initial bolus and two following lower doses is necessary for a long-term effect of the clinically applied inhibitor Ruxolitinib, thus counteracting its rapid metabolism (Shilling et al., 2010) resulting in its short half-life (Shi et al., 2011).

Multiple-dose Ruxolitinib treatment with equal doses was shown earlier to have a more sustained effect on STAT3 signaling, compared with single treatment (Shi et al., 2011). Importantly, Shi et al. found negligible accumulation of Ruxolitinib after multiple doses, thus minimizing the risk of potential side effects. A twice-daily dosing regimen was furthermore successfully applied in the treatment of myelofibrosis patients (Verstovsek et al., 2012). Notably, previous treatment planning was based on preclinical data and empirical results from clinical trials (Quintas-Cardama et al., 2010; Shi et al., 2011).

While the single treatment with Ruxolitinib did reduce the APP response after treatment with IL-6, more sustained inhibition may still be of importance, but an increase in dose is not desirable due to harmful side-effects such as neutropenia (Shi et al., 2011). If we consider the parameter sensitivity analysis (see Figure 7A), we can observe that additional alternatives exist to further suppress the APP response. One option would be to selectively reduce the *Socs3* mRNA degradation rate to benefit from synergistic effects between the pathway's natural inhibitor SOCS3 and the drug Ruxolitinib. However, since selective inhibition of mRNA degradation for one specific mRNA species may not be feasible, a more practical option could be to reduce receptor accessibility. This may be achieved by additional application of a therapeutic antibody against the IL-6 receptor.

*In silico* analyses, based on mathematical models represent promising approaches to optimally exploit an inhibitor's potential and pre-assess drug safety, prior to testing the drug in patients or healthy individuals. Our established mathematical model represents a starting point for further adaptation to the human system, and could facilitate *in silico* drug treatment planning in the future.

## Materials and methods

### Chemicals

Chemicals were purchased from Sigma-Aldrich, if not specified otherwise.

### Partial hepatectomy (PHx) and LPS treatments of mice

C57BL/6J mice (Janvier) were housed in the animal facility of the Heinrich-Heine-University of Düsseldorf under a constant light/dark cycle, maintained on a standard mouse diet, and allowed *ad libitum* access to food and water. Male, 8–12 week old mice were used for PHx and LPS experiments. Procedures were approved by the North Rhine-Westphalia State Agency for Nature, Environment and Consumer Protection (reference number 87-51.04.2010.A279 for PHx experiments; reference number 84-02.04.2011.A096 for LPS injections). PHx was performed based on the standardized procedure described by Mitchell and Willenbring (2008). Mice were anesthetized using isoflurane (Abott) and received 5 mg/kg body weight carprofen (Pfizer) subcutaneously for analgesia during surgery and the three following days. During the operation, mice were placed on a warming pad. The abdominal cavity was opened applying a 3 cm incision. The left lateral liver lobe was removed by applying a ligature (time point 0 h) close to the base of the lobe followed by cutting the tied lobe just above the suture. A second ligature was placed around the median lobe above the gall bladder but with at least 2 mm distance to the suprahepatic vena cava. The tied median lobe including gall bladder was then resected by cutting just above the suture. Ringer's lactate solution (B.Braun) was applied to detect possible abdominal bleeding which, if present, was stopped before closing peritoneum and skin by over-and-over sutures. The weight of the resected left lateral and median liver lobes was determined. Mice were monitored during awakening and the following days including daily determination of body weight. Sham surgeries were performed analogous to PHx, but without placing ligatures and liver lobe removal. Liver lobes were moved as during PHx operations (time point 0 h).

For LPS-injections, LPS (*Escherichia coli* 0111:B4) was dissolved in 0.9% NaCl (Baxter) and injected intraperitoneally at a concentration of 1 μg/g body weight. At indicated time points after Sham/PHx surgery or LPS-injection mice were anesthetized as outlined above and blood was collected from the vena cava. After clotting, blood serum was obtained by two centrifugation steps at 10,000 × g for 10 min. Livers were perfused in an antegrade direction with cold PBS (Biochrom) supplemented with 0.1 mM Na_3_VO_4_ until perfusate was clear. Livers were extracted from mice, flash frozen in liquid nitrogen, and stored at –80°C.

### Isolation of primary mouse hepatocytes

Primary mouse hepatocyte isolation was performed in a standardized way according to Klingmuller et al. (2006) or according to the refined protocol described by Huard et al. (2012). C57BL/6N mice (Charles River) were housed at the DKFZ animal facility under a constant light/dark cycle, maintained on a standard mouse diet, and allowed *ad libitum* access to food and water. Hepatocyte isolation from mice was approved by the governmental review committee on animal care of the state Baden-Württemberg, Germany (reference number A24/10). For standard time course and dose response experiments, 2 × 10^6^ cells were seeded in 6 cm collagen I-coated tissue culture plates (BD Biosciences) in 2 mL of adhesion medium [phenol red-free Williams E medium (Biochrom) containing 10% (v/v) fetal bovine serum (Life Technologies), 0.1 μM dexamethasone, 10 μg/mL insulin, 2 mM l-glutamine and 1% (v/v) penicillin/streptomycin 100 × (both Life Technologies)]. Cells were maintained at 37°C, 5% CO_2_, and 95% relative humidity. After 4 h of adhesion, unattached hepatocytes were removed by washing 3 × with DPBS (PAN Biotech) followed by over-night cultivation (14–16 h) in pre-starvation medium [phenol red-free Williams E medium containing 0.1 μM dexamethasone, 2 mM l-glutamine, and 1% (v/v) penicillin/streptomycin 100 × ]. The next day cells were washed 3 × with DPBS and cultured for 5 h in starvation medium [phenol red-free Williams E medium supplemented with 2 mM l-glutamine, 1% (v/v) penicillin/streptomycin 100 × and 25 mM HEPES] prior to inhibitor/IL-6 treatment. Hepatocytes were always isolated as described and cultivated on collagen I-coated tissue culture plates (BD Biosciences). Differing cell numbers and plate formats or extended pre-starvation periods are indicated in respective methods sections.

### Isolation of primary human hepatocytes

The isolation of primary human hepatocytes was performed as described in Iwamoto et al. (2016). For the isolation of the primary human hepatocytes macroscopically healthy tissue was used that originated from resected tumor-free tissue from human livers of three patients (Donor 1: age 65, gender male, disease hepatocellular carcinoma with cirrhosis and diabetes; Donor 2: age 68, gender male, disease hepatocellular carcinoma with nutritive-toxic liver cirrhosis, diabetes, arterial hypertonia; Donor 3: age 78, gender female, disease Klatskin tumor with Steatosis hepatis grade 2, diabetes, arterial hypertonia). Informed consent of the patients was obtained according to the ethical guidelines of the Medical Faculty of the University of Leipzig. Primary human hepatocytes were shipped as cell suspension in ChillProtec Plus (Biochrom) on ice overnight to DKFZ Heidelberg. Primary human hepatocytes were serum- and dexamethasone-depleted and cultivated using the protocol described above for primary mouse hepatocytes, with an adhesion time of 6 h.

### Inhibitor and IL-6 treatments

The STAT3 inhibitor Stattic (Merck Millipore) and the JAK inhibitor Ruxolitinib (Cayman Chemical) were reconstituted in DMSO and primary mouse hepatocytes were pre-treated with the indicated concentrations of inhibitors or DMSO control for 1 h prior to IL-6 stimulation. Actinomycin D was dissolved in DMSO and cells were pre-treated with 1 μg/mL actinomycin D or DMSO control for 10 min prior to addition of IL-6. Human recombinant hIL-6 was manufactured as described in Vandam et al. (1993). Mouse IL-6 was purchased from R & D (406-ML-005). IL-6 stock solutions were diluted in starvation medium and cells were stimulated with the indicated IL-6 concentrations and time spans. Pulsed stimulation was achieved by carefully washing the cells 3 × with starvation medium to remove unbound IL-6 ligand at indicated time points. For treatment durations of up to 2 h, cells were kept at 37°C in a bench-top incubator after inhibitor and/or IL-6 stimulation. During long-term experiments cells were incubated at 37°C, 5% CO_2_, and 95% relative humidity.

### Immunoassays for the quantification of IL-6 levels in serum and hepatocyte supernatants

IL-6 concentrations in mouse serum were quantified using the MILLIPLEX mouse cytokine/chemokine magnetic bead panel (EMD Millipore) according to the manufacturer's instructions. Samples were incubated with antibody-coupled beads overnight. Washing procedures were performed using the ELx405 wash station (BioTek) and fluorescence intensity was detected by a Luminex 200 System in combination with xPONENT Software version 3.1 (Millipore). IL-6 concentrations in hepatocyte supernatants were measured using the Bio-Plex Pro human IL-6 assay in combination with the Bio-Plex Pro reagent kit (both Bio-Rad) according to the manufacturer's instructions. A dilution series of the recombinant human IL-6 used for stimulation was used as standard curve. Washing was performed using the Bio-Plex Pro II wash station (Bio-Rad) and fluorescence intensity was acquired using the Bio-Plex 200 system and Bio-Plex Manager software version 6.1 (both Bio-Rad). Alternatively, IL-6 concentrations in hepatocyte supernatants were determined using the Quantikine Human IL-6 Immunoassay (R&D Systems). Stabilization of ligand in medium samples was achieved by supplementing 450 μL conditioned medium with 50 μL of 40 mM HCl and 10 mg/mL BSA.

### Quantitative immunoblotting

At precise time points cells were lysed in 1% Nonidet P-40 lysis buffer [1% (v/v) Nonidet P-40 (Roche Applied Sciences), 150 mM NaCl, 20 mM Tris pH 7.4, 10 mM NaF, 1 mM EDTA (Applichem) pH 8.0, 1 mM ZnCl_2_ pH 4.0, 1 mM MgCl_2_, 1 mM Na_3_VO_4_, 10% glycerol; freshly supplemented with 2 μg/mL aprotinin and 200 μg/mL 4-(2-aminoethyl)benzenesulfonylfluorid] and cleared lysates were either directly subjected to SDS-PAGE or used for immunoprecipitations. For cellular fractionation, cytosolic extracts were prepared as described above using Nonidet P-40 lysis buffer. Pelleted nuclei were washed once with Nonidet P-40 lysis buffer and then resuspended in nuclear lysis buffer [420 mM NaCl, 20 mM HEPES pH 7.9, 10 mM KCl, 1 mM EDTA pH 8.0, 1 mM Na_3_VO_4_, 10% (v/v) glycerol) supplemented with 2 μg/mL aprotinin, 200 μg/mL 4-(2-aminoethyl)benzenesulfonylfluorid and 1 mM DTT]. Nuclei were lysed by pulsed sonication and cleared nuclear lysates were subjected to SDS-PAGE or used for immunoprecipitations. Quality of fractionation was checked by correct subcellular localization of marker proteins Sp1 (nuclear; Santa Cruz Biotechnology, #sc-59) and Eps15 (cytosolic; Santa Cruz Biotechnology, #sc-534).

Protein concentrations of lysates were quantified by BCA assay (Pierce, Thermo Scientific). To immunoprecipitate target proteins, lysates were incubated with anti-gp130 (C20, Santa Cruz Biotechnology, #sc-655), anti-JAK1 serum (Upstate/Merck Millipore, #06-272), anti-STAT3 (Cell Signaling Technology, #9132) or anti-SOCS3 (clone 1B2, Invitrogen, #37-7200) antibodies, protein-A sepharose (GE Healthcare), and recombinant calibrator proteins. For immunoprecipitation experiments the following recombinant proteins were added as calibrator proteins directly to the cell lysates to enable normalization of immunoblot data: Glutathione S-transferase (GST)-tagged gp130ΔN (cytoplasmic domain); GST-STAT3 (full length protein) and Streptavidin binding protein (SBP)-tagged SOCS3 (full length protein). Precipitated proteins and cytoplasmic or nuclear lysates (40–50 μg) were resolved by 10% SDS-polyacrylamide gel electrophoresis and transferred to nitrocellulose membranes according to previously described recommendations for quantitative immunoblotting (Schilling et al., 2005a). Membranes were incubated with anti-phosphotyrosine antibody (4G10, Upstate/Merck Millipore, #05 321) to detect the phosphorylated forms of gp130 and JAK1, anti-gp130 (C20, Santa Cruz Biotechnology, #sc-655), anti-JAK1 (Cell Signaling Technologies, #3332), anti-phospho-STAT3, anti-STAT3 (both Cell Signaling Technologies, 3E2 #9138, #9132) and anti-SOCS3 (Abcam, #ab16030) antibodies. For normalization in cell lysate samples, anti-calnexin and anti-Hsc70 (both Stressgen, #ADI-SPA-860, #SPA-816) antibodies were applied. Nuclear marker proteins were detected by anti-Sp1 and anti-Eps15 antibodies (both Santa Cruz, #sc-59, #sc-534). Horseradish peroxidase coupled secondary antibodies (anti-mouse, anti-rabbit, protein A) were derived from GE Healthcare. Antibodies were removed by β-mercaptoethanol/SDS-treatment prior to re-probing for a different protein. Phosphorylated species were detected first, followed by total proteins and normalizers. Proteins were visualized using enhanced chemiluminescence substrate (GE Healthcare) and signals were detected using a CCD camera (LumiImager F1, Roche; or ImagequantLAS4000, GE Healthcare). For band quantification, LumiAnalyst 3.1 (Roche) or ImagequantTL (GE Healthcare) software was used. Quantitative immunoblotting data were either processed using GelInspector software (Schilling et al., 2005b) or directly used for mathematical modeling.

### Bead-based immunoassays for the analysis of STAT3 activation

IL-6 concentrations in the liver were determined by measuring STAT3 activation as read-out. Livers from Sham/PHx or NaCl/LPS-treated mice as well as primary mouse hepatocytes were lysed in total cell lysis buffer [136 mM NaCl, 20 mM Tris-HCl, 10% glycerol, 2 mM EDTA, 50 mM β-glycerophosphate, 20 mM sodium pyrophosphate, 1 mM Na_3_VO_4_, 1% Triton X-100, 0.2% SDS, 1 tablet/10 mL complete Mini EDTA-free protease inhibitors (Roche), pH 7.4]. For other experiments, hepatocytes were lysed using Nonidet P-40 lysis buffer as described above. Livers were homogenized using a microcentrifuge tube-pestle followed by passing through QIAshredder (Qiagen) columns. Cleared liver and hepatocyte lysates were subjected to BCA assay (Pierce, Thermo Scientific) to determine protein concentrations. Relative phospho-STAT3 levels were quantified using the bead-based Bio-Plex phospho-STAT3 (Tyr-705) assay in combination with the Bio-Plex phosphoprotein detection reagent kit, or using the magnetic bead-based Bio-Plex Pro phospho-STAT3 (Tyr-705) set (all Bio-Rad) according to the manufacturer's instructions. Equal amounts of protein (16.67 μg/well or 10 μg/well in a 96-well plate format) were incubated with antibody-coupled beads overnight. For washing steps, the Bio-Plex Pro II wash station (Bio-Rad) was used. The fluorescence intensity corresponding to relative phospho-STAT3 levels was acquired using the Bio-Plex 200 system and Bio-Plex Manager software version 6.1 (both Bio-Rad).

### Quantification of target gene expression by quantitative real-time PCR (qRT-PCR)

Cells were collected in RLT Plus lysis buffer and lysates were homogenized using QIAshredder spin columns (both Qiagen). Homogenized lysates were immediately placed on dry-ice and stored at –80°C until RNA isolation. RNA was extracted using the RNeasy Plus Mini Kit (Qiagen) according to the manufacturer's instructions. Reverse transcription was performed using either the High Capacity cDNA Reverse Transcription Kit (Applied Biosystems) or the QuantiTect Reverse Transcription Kit (Qiagen). Diluted cDNA was analyzed applying the Universal ProbeLibrary System on a LightCycler 480 (both Roche), cycling conditions can be found in Appendix Table S2. Relative mRNA concentrations were calculated according to a cDNA dilution series with the Absolute Quantification Second Derivative Maximum method of the LightCycler 480 Basic Software (Roche). Target mRNA concentrations were normalized to the geometric mean of *Hprt*/*Tbp* concentrations or to *Hprt* concentrations. Primer/probe combinations were designed using the Universal ProbeLibrary Assay Design Center (Roche) and are listed in Appendix Table S3.

### Microarray experiment

Primary mouse hepatocytes (2 × 10^6^ cells per 10 cm dish) were cultivated in pre-starvation medium for 24 h before ligand treatment. hIL-6 was added directly to cells in pre-starvation medium at 40 ng/mL and untreated or IL-6-treated samples were collected at time points 0 (4 replicates), 0.5, 2, 4, 8, 16, 24, 32 h (2 replicates each). Results are shown in duplicates, for time point 0 the first two replicates were utilized. RNA was isolated as described above (RNeasy, Qiagen) and gene expression was analyzed on GeneChip® Mouse Genome 430 2.0 Arrays (Affymetrix). The microarray data is accessible via the following URL: https://www.ncbi.nlm.nih.gov/geo/query/acc.cgi?acc=GSE69939.

### Microarray analysis

Principal component analysis was performed on the global transcriptional profiles. The first two principal components explained most of the variance in the data set (PC1: 58.4%, PC2: 9.7%, 68.1% in total).

To analyze significant gene regulation, we applied a linear regression model with the Limma package (Ritchie et al., 2015). Gene expression values *y*_*i*_ were modeled to be explained by time frames *t* (early, intermediate and late) and condition *c* (IL-6 and control). The significance threshold of Benjamini–Hochberg adjusted *p* < 0.01 was implemented. For example “early IL-6 response” was extracted from the global linear model by performing contrast analysis of early IL-6 vs. respective (unpaired) early control samples.

All analyses were performed in R Statistical software (www.r-project.org).

Ontology analysis of the three response lists was performed using a model-based ontology analysis (*http://nar.oxfordjournals.org/content/early/2010/02/19/nar.gkq045.full*) using Wiki Pathways and implementing an enrichment threshold using pathways with a probability of being regulated > 0.5.

### Mass spectrometric analysis

Primary mouse hepatocytes (5 × 10^6^ cells per 10 cm dish) were cultivated for 40 h in serum-depleted medium. Cells were then serum- and dexamethasone-depleted for 5 h in starvation medium, stimulated with 40 ng/mL hIL-6 for 18 min, and lysed in Nonidet P-40 lysis buffer. STAT3 immunoprecipitations were subjected to 10% SDS-PAGE and proteins were stained with SimplyBlue SafeStain (Life Technologies). STAT3-α bands were excised, cut into small pieces (~1 mm^3^) and destained with 0.07 M NH_4_HCO_3_ buffer/30% acetonitrile. Gel pieces were dehydrated in 0.1% trifluoroacetic acid/50% acetonitrile, followed by protein in-gel reduction with 10 mM dithiothreitol (45 min at 56°C) and alkylation with 55 mM iodoacetamide for 30 min in the dark. Digestions were performed with AspN + LysC in 0.05 M NH_4_HCO_3_ buffer at 37°C overnight. Following incubation, internal peptide-/phospho-peptide one-source ratio standards for quantification of STAT3 Tyr-705 phosphorylation were added. The standard consists of the isotope labeled [^13^C_5_,^15^N] peptides DPGSAAP-**pY**-[L+6Da]-K and DPGSAAP-**Y**-[L+6Da]-K at an exact molar ratio of 1:1. Following standard addition to the gel pieces and 15 min of shaking the supernatant of each sample was collected. Peptide extraction was finished by sequentially adding appropriate volumes of eluents to the gel pieces, shaking them and combining all the supernatants for each sample. The eluents were (i) acetonitrile, (ii) 5% formic acid, and (iii) acetonitrile. The collected sample volumes were reduced by speedvac and purified by applying the ZipTip method (Millipore) according to the manufacturer's recommendations. Final sample volumes of 5 μl were injected into an ultra-performance liquid chromatography (nanoUPLC, nanoAcquity, Waters) online coupled to a Q Exactive Plus-Orbitrap mass spectrometer (Thermo). For details about preparation and application of peptide-/phosphopeptide one-source ratio standards see Hahn et al. (2011) and Boehm et al. (2014).

### Generation of mKate2-STAT3 knock-in mouse

To generate the mKate2-STAT3 reporter gene, we based the fusion construct on earlier studies (see Herrmann et al., 2007; Samsonov et al., 2013) and inserted the mKate2-coding sequence in front of the first exon of STAT3 by BAC recombineering. An insert harboring mKate2 and part of STAT3 as well as the Neomycin selection cassette flanked by homologous arms was retrieved into PL253 vector (NCI Frederick; Liu et al., 2003) to obtain the gene targeting construct. Gene targeting was performed in the mouse embryonic stem (ES) cell line JM8A3 (Pettitt et al., 2009) by electroporation of the linearized gene targeting construct followed by selection with G418 (Life Technologies) and Ganciclovir. Correctly targeted ES cell clones were identified by long-range PCR and confirmed with southern blot. Chimera were generated by blastocyst injection of correctly targeted ES clones. Male chimera were bred with female wild type C57BL/6N mice to promote germline transmission of the reporter gene. Germline transmission was identified by genotyping PCR. The selection cassette was removed by subsequently crossing heterozygous mice with Cre expressing mice (Schwenk et al., 1995). Only heterozygous mice were used for the experiments, because it was so far not possible to obtain homozygous mKate2-STAT3 reporter mouse offsprings. See Appendix for more information about gene targeting and genotyping.

### Live-cell imaging

Primary hepatocytes (15,000 cells per well, 96-well plate format) derived from mKate2-STAT3 heterozygous knock-in mice (Appendix Supplementary Experimental Procedures and Table S1) were infected with adeno-associated viruses encoding mCerulean-labeled histone-2B during adhesion. Cells were cultivated as described above, stimulated with inhibitor/ligand, and imaged using a Nikon Eclipse Ti Fluorescence microscope in combination with NIS-Elements software. Temperature (37°C), CO_2_ (5%), and humidity were held constant through an incubation chamber enclosing the microscope. Three channels were acquired for each position: bright-field channel, STAT3 channel (mKate2), and nuclear channel (CFP). Image analysis was performed using Fiji software (Schindelin et al., 2012), and data were processed using R software (The R Foundation). The ratio of nuclear to cytoplasmic (nuc/cyt) mKate2-STAT3 was determined in 20 cells, facilitated by manual segmentation of nuclei (histone-2B-mCerulean signal) and whole cells (bright-field channel). The mean concentration of cytoplasmic STAT3 was derived assuming constant overall mKate2-STAT3. mKate2 background was determined in wild-type nuclei and cytoplasm and subtracted accordingly.

### Mathematical modeling

A mathematical multi-compartment model describing IL-6 signaling in primary mouse hepatocytes was developed. The model is described by a set of coupled non-linear differential equations implemented using the Data2Dynamics software package (Raue et al., 2015) In each simulated experiment, the model is equilibrated to steady state prior to treatment with inhibitors or stimulation. Considering the size and complexity of the model and experimental data, model calibration was performed in two separate stages. The core model describes receptor production, degradation and phosphorylation as well as activation and translocation of STAT3, negative feedback by SOCS3 and the effect of inhibitors, while the downstream model describes the transcription of the various APP genes. Parameters for the upstream and downstream model were estimated separately. All model parameters were estimated directly from the experimental data using Maximum Likelihood Estimation. Several experiments required the use of scaling, offset and error model parameters that were estimated simultaneously with the dynamic parameters. For the core model, 270 parameters (of which 22 dynamic parameters) were estimated on a total of 2220 data points. For the downstream components, we estimated 471 additional parameters (of which 29 dynamic parameters) on a total of 2,288 data points.

To evaluate that parameters are identifiable (Maiwald et al., 2016), profile likelihood calculation followed by either model reduction or additional data acquisition were iteratively applied. For a full mathematical description of the model, including a detailed description of the iterative model building and reduction steps see Appendix section 3.5.

Local Parameter Sensitivity Analysis (LPSA) was performed with respect to model parameters. The local parameter sensitivity for a single APP gene / parameter pair is defined as:

(1) $$\begin{array}{l} S_{app}=\frac{\left(y-y_{ref}\right)/y_{ref}}{\left(p-p_{ref}\right)/p_{ref}} \end{array}$$

Here *y* refers to the model output at the perturbed parameter, while *y*_*ref*_ indicates the reference output. As model output, we selected the integral of the mRNA levels. Analogously, *p* and *p*_*ref*_ refer to the parameter value in the perturbed and reference state. These sensitivities are then computed for each of the APP genes and averaged. To assess how much uncertainty there is in these sensitivities, we computed an LPSA for each parameter set in our parameter profile likelihoods and reported the maximum and minimum value encountered within the confidence intervals of all parameters.

The mathematical model is available to the community at the biomodels database as well as on www.data2dynamics.org.

## Author contributions

SS, AR, XH, JV, SeB, JT, MS, UK were responsible for study conception and design. SS, AR, XH, AJ, SeB, UA, MH, SW, LAD, SM, MB, PL, SaB, WL, JB acquired the data. GD, DS provided primary human hepatocytes. SR provided recombinant human IL-6. NG conducted the microarray experiments. XH and FvdH generated the recombinant mouse line. SS, AR, XH, JV, SaB, UA, MH, SW, NM, LAD, SM, MB, PL, WL, FT, JT, MS, UK performed the analysis and interpretation of data. SS, AR, XH, JV, AJ, JT, MS, UK drafted the manuscript and all authors critically revised the manuscript.

### Conflict of interest statement

The authors declare that the research was conducted in the absence of any commercial or financial relationships that could be construed as a potential conflict of interest.
