# Supplementary material for: Model Based Targeting of IL-6-Induced Inflammatory Responses in Cultured Primary Hepatocytes to Improve Application of the JAK Inhibitor Ruxolitinib
Source: Front Physiol. 2017 Oct 9;8:775. doi: 10.3389/fphys.2017.00775 (PMC5640784; doi:10.3389/fphys.2017.00775)
Supplement: Supplementary file 1 [file DataSheet1.PDF]

Appendix: Model based Targeting of IL-6-induced Inflammatory Responses in Cultured Primary Hepatocytes to Improve Application of the JAK Inhibitor Ruxolitinib

Svantje Sobotta, Andreas Raue, Xiaoyun Huang, Joep Vanlier, Anja Jünger, Sebastian Bohl, Ute Albrecht, Maximilian J. Hahnel, Stephanie Wolf, Nikola S. Mueller, Lorenza A. D'Alessandro, Stephanie Mueller-Bohl, Martin E. Boehm, Philippe Lucarelli, Sandra Bonefas, Georg Damm, Daniel Seehofer, Wolf D. Lehmann, Stefan Rose-John, Frank van der Hoeven, Norbert Gretz, Fabian J. Theis, Christian Ehlting, Johannes G. Bode, Jens Timmer, Marcel Schilling, Ursula Klingmüller

Contents

1 Supplementary Experimental Procedures **8**
1.1 Generation of mKate2-Stat3 Knock-in Mice . . . 8
1.1.1 ESC Gene Targeting . . . 8
1.1.2 Blastocyst Injection and Embryo Transfer . . . 8
1.2 Genotyping PCR . . . 9
1.3 Southern Blotting . . . 9
1.4 AAV Vector Production . . . 9
1.5 Live Cell Imaging of mKate2-STAT3 . . . 10
1.6 Generation of Recombinant Calibrator Proteins . . . 10
1.7 Preparation of total cell lysate from primary human hepatocytes . . . 10
1.8 Quantitative real-time PCR . . . 10

2 Supplementary Figures **12**
2.1 Figure S1 . . . 12
2.2 Figure S2 . . . 13
2.3 Figure S3 . . . 14
2.4 Figure S4 . . . 15
2.5 Figure S5 . . . 16
2.6 Figure S6 . . . 17
2.7 Figure S7 . . . 18
2.8 Figure S8 . . . 19
2.9 Figure S9 . . . 20
2.10 Figure S10 . . . 21
2.11 Figure S11 . . . 22
2.12 Figure S12 . . . 23
2.13 Figure S13 . . . 24
2.14 Figure S14 . . . 25

3 Mathematical Modeling **26**
3.1 Model Description . . . 26
3.2 Observables . . . 31
3.3 Default Parameter Transformations . . . 31

3.4 Dynamic Parameters . . . 32
3.5 Model Development . . . 34
3.6 Sensitivity Analysis . . . 43
3.7 Experiment Design . . . 45

4 Experiments **48**
4.1 Experiment: bohl hep 2005 02 03 Cont40ng T90min . . . 49
4.1.1 Comments . . . 49
4.1.2 Model fit and plots . . . 49
4.2 Experiment: bohl hep 2004 10 26 Cont 30min . . . 50
4.2.1 Comments . . . 50
4.2.2 Model fit and plots . . . 50
4.3 Experiment: bohl hep 2005 02 14 DoseResponse T15min . . . 51
4.3.1 Comments . . . 51
4.3.2 Model fit and plots . . . 51
4.3.3 Condition dependent parameter changes . . . 51
4.4 Experiment: bohl hep 2005 03 01 Pulse20min . . . 52
4.4.1 Comments . . . 52
4.4.2 Model fit and plots . . . 52
4.5 Experiment: bohl hep 2005 03 16 Cont40ng 23min . . . 54
4.5.1 Comments . . . 54
4.5.2 Model fit and plots . . . 54
4.6 Experiment: bohl hep 2005 07 26 Pulse5min . . . 55
4.6.1 Comments . . . 55
4.6.2 Model fit and plots . . . 55
4.7 Experiment: bohl hep 2005 11 22 DoseResponseUntil120ng . . . 57
4.7.1 Comments . . . 57
4.7.2 Model fit and plots . . . 57
4.7.3 Condition dependent parameter changes . . . 57
4.8 Experiment: bohl hep 2009 10 14 qRTPCR . . . 58
4.8.1 Comments . . . 58
4.8.2 Model fit and plots . . . 58
4.9 Experiment: bohl mlc per cell log . . . 59
4.9.1 Comments . . . 59
4.9.2 Model fit and plots . . . 59
4.10 Experiment: bohl hep 2007 07 10 ActD . . . 60
4.10.1 Comments . . . 60
4.10.2 Model fit and plots . . . 60
4.10.3 Condition dependent parameter changes . . . 61
4.11 Experiment: bohl hep 2006 06 07 ActD . . . 62
4.11.1 Comments . . . 62
4.11.2 Model fit and plots . . . 62
4.11.3 Condition dependent parameter changes . . . 63
4.12 Experiment: bohl hep 2006 02 09 FourPulses ReceptorNucleus . . . 64
4.12.1 Comments . . . 64
4.12.2 Model fit and plots . . . 64
4.12.3 Condition dependent parameter changes . . . 64
4.13 Experiment: bohl hep 2006 04 20 ThreePulses ThreeDoses . . . 66
4.13.1 Comments . . . 66
4.13.2 Model fit and plots . . . 66
4.13.3 Condition dependent parameter changes . . . 67
4.14 Experiment: bohl hep 2006 05 16 ThreeDoses . . . 68

4.14.1 Comments . . . 68
4.14.2 Model fit and plots . . . 68
4.14.3 Condition dependent parameter changes . . . 69
4.15 Experiment: bohl hep 2006 11 15 FourPulses 120min . . . 70
4.15.1 Comments . . . 70
4.15.2 Model fit and plots . . . 70
4.15.3 Condition dependent parameter changes . . . 71
4.16 Experiment: bohl hep 2007 04 04 DoseResponse 3TP . . . 72
4.16.1 Comments . . . 72
4.16.2 Model fit and plots . . . 72
4.16.3 Condition dependent parameter changes . . . 73
4.17 Experiment: bohl hep 2007 07 17 DRTC . . . 74
4.17.1 Comments . . . 74
4.17.2 Model fit and plots . . . 74
4.17.3 Condition dependent parameter changes . . . 75
4.18 Experiment: bohl hep 2009 06 23 DRTC . . . 76
4.18.1 Comments . . . 76
4.18.2 Model fit and plots . . . 76
4.18.3 Condition dependent parameter changes . . . 77
4.19 Experiment: bohl hep 2009 07 07 DRTC . . . 78
4.19.1 Comments . . . 78
4.19.2 Model fit and plots . . . 78
4.19.3 Condition dependent parameter changes . . . 79
4.20 Experiment: braun hep 2013 11 04 Stattic Ruxolitinib Inhibitor . . . 80
4.20.1 Comments . . . 80
4.20.2 Model fit and plots . . . 80
4.20.3 Condition dependent parameter changes . . . 80
4.21 Experiment: braun hep 2013 11 12 Stattic Ruxolitinib Inhibitor . . . 81
4.21.1 Comments . . . 81
4.21.2 Model fit and plots . . . 81
4.21.3 Condition dependent parameter changes . . . 81
4.22 Experiment: braun app hep 2014 04 28 IL6DR Rux 1h SOCS3 . . . 82
4.22.1 Model fit and plots . . . 82
4.22.2 Condition dependent parameter changes . . . 82
4.23 Experiment: braun app hep 2011 06 06 qPCR 140109 IL6DR 1h . . . 83
4.23.1 Model fit and plots . . . 83
4.23.2 Condition dependent parameter changes . . . 83
4.24 Experiment: braun app hep 2013 10 14 qPCR 140109 IL6DR 1h . . . 84
4.24.1 Model fit and plots . . . 84
4.24.2 Condition dependent parameter changes . . . 84
4.25 Experiment: braun app hep 2013 10 21 qPCR 140109 IL6DR 1h . . . 85
4.25.1 Model fit and plots . . . 85
4.25.2 Condition dependent parameter changes . . . 85
4.26 Experiment: braun app hep 2012 02 14 qPCR 140224 IL6DR Inh 1h Socs3 . . . 86
4.26.1 Model fit and plots . . . 86
4.26.2 Condition dependent parameter changes . . . 86
4.27 Experiment: braun app hep 2012 04 10 qPCR 140224 IL6DR Inh 1h Socs3 . . . 87
4.27.1 Model fit and plots . . . 87
4.27.2 Condition dependent parameter changes . . . 87
4.28 Experiment: braun app hep 2013 10 14 qPCR 140224 IL6DR Inh 1h Socs3 . . . 88
4.28.1 Model fit and plots . . . 88
4.28.2 Condition dependent parameter changes . . . 88

4.29 Experiment: braun app hep 2014 04 22 qPCR 140526 IL6DR Rux 6h SOCS3 89
4.29.1 Model fit and plots . . . 89
4.29.2 Condition dependent parameter changes . . . 89
4.30 Experiment: braun app hep 2014 05 19 qPCR 140526 IL6DR Rux 6h SOCS3 90
4.30.1 Model fit and plots . . . 90
4.30.2 Condition dependent parameter changes . . . 90
4.31 Experiment: braun app hep 2014 04 22 qPCR 140604 IL6DR Rux 24h SOCS3 91
4.31.1 Model fit and plots . . . 91
4.31.2 Condition dependent parameter changes . . . 91
4.32 Experiment: braun app hep 2014 05 19 qPCR 140604 IL6DR Rux 24h SOCS3 92
4.32.1 Model fit and plots . . . 92
4.32.2 Condition dependent parameter changes . . . 92
4.33 Experiment: braun hep 2011 09 06 DRTC . . . 93
4.33.1 Comments . . . 93
4.33.2 Model fit and plots . . . 93
4.33.3 Condition dependent parameter changes . . . 93
4.34 Experiment: braun hep 2011 04 04 STAT3 pY degree replicates . . . 94
4.34.1 Comments . . . 94
4.34.2 Model fit and plots . . . 94
4.34.3 Observables . . . 94
4.34.4 Condition dependent parameter changes . . . 95
4.35 Experiment: braun hep 2013 07 08 qPCR Socs3 Stat3 . . . 96
4.35.1 Comments . . . 96
4.35.2 Model fit and plots . . . 96
4.35.3 Condition dependent parameter changes . . . 96
4.36 Experiment: braun hep 2011 12 12 Stattic Stat3 Inhibitor DR . . . 97
4.36.1 Comments . . . 97
4.36.2 Model fit and plots . . . 97
4.36.3 Condition dependent parameter changes . . . 97
4.37 Experiment: braun hep 2012 01 10 Stattic Stat3 Inhibitor DR . . . 98
4.37.1 Comments . . . 98
4.37.2 Model fit and plots . . . 98
4.37.3 Condition dependent parameter changes . . . 98
4.38 Experiment: braun hep 2012 01 23 Stattic Stat3 Inhibitor TC . . . 99
4.38.1 Comments . . . 99
4.38.2 Model fit and plots . . . 99
4.38.3 Condition dependent parameter changes . . . 99
4.39 Experiment: braun hep 2012 01 23 Stattic Stat3 Inhibitor . . . 100
4.39.1 Comments . . . 100
4.39.2 Model fit and plots . . . 100
4.39.3 Condition dependent parameter changes . . . 100
4.40 Experiment: braun hep 2012 02 14 Stattic Stat3 Inhibitor TC . . . 101
4.40.1 Comments . . . 101
4.40.2 Model fit and plots . . . 101
4.40.3 Condition dependent parameter changes . . . 101
4.41 Experiment: braun hep 2012 04 10 Stattic Stat3 Inhibitor TC . . . 102
4.41.1 Comments . . . 102
4.41.2 Model fit and plots . . . 102
4.41.3 Condition dependent parameter changes . . . 102
4.42 Experiment: braun hep 2012 05 02 Stattic Stat3 Inhibitor 40 . . . 103
4.42.1 Comments . . . 103
4.42.2 Model fit and plots . . . 103

4.42.3 Condition dependent parameter changes . . . 103
4.43 Experiment: braun hep 2012 05 02 Stattic Stat3 Inhibitor 60 . . . 104
4.43.1 Comments . . . 104
4.43.2 Model fit and plots . . . 104
4.43.3 Condition dependent parameter changes . . . 104
4.44 Experiment: braun hep 2013 06 03 Ruxolitinib . . . 105
4.44.1 Comments . . . 105
4.44.2 Model fit and plots . . . 105
4.44.3 Condition dependent parameter changes . . . 105
4.45 Experiment: braun hep 2013 06 17 Ruxolitinib . . . 106
4.45.1 Comments . . . 106
4.45.2 Model fit and plots . . . 106
4.45.3 Condition dependent parameter changes . . . 106
4.46 Experiment: braun hep 2013 09 30 Ruxolitinib Inhibitor TC . . . 107
4.46.1 Comments . . . 107
4.46.2 Model fit and plots . . . 107
4.46.3 Condition dependent parameter changes . . . 107
4.47 Experiment: xiaoyun nucSTAT3 Ratio . . . 108
4.47.1 Model fit and plots . . . 108
4.47.2 Condition dependent parameter changes . . . 108
4.48 Experiment: APP calibration 1h . . . 109
4.48.1 Model fit and plots . . . 109
4.48.2 Condition dependent parameter changes . . . 109
4.49 Experiment: braun calibration hep 2014 01 13 IL6DR 6h Replicate1 . . . 110
4.49.1 Model fit and plots . . . 110
4.49.2 Condition dependent parameter changes . . . 110
4.50 Experiment: braun calibration hep 2014 01 13 IL6DR 6h Replicate2 . . . 111
4.50.1 Model fit and plots . . . 111
4.50.2 Condition dependent parameter changes . . . 111
4.51 Experiment: braun calibration hep 2014 01 13 IL6DR 6h Replicate3 . . . 112
4.51.1 Model fit and plots . . . 112
4.51.2 Condition dependent parameter changes . . . 112
4.52 Experiment: braun calibration hep 2014 01 27 IL6DR 24h Replicate1 . . . 113
4.52.1 Model fit and plots . . . 113
4.52.2 Condition dependent parameter changes . . . 113
4.53 Experiment: braun calibration hep 2014 01 27 IL6DR 24h Replicate2 . . . 114
4.53.1 Model fit and plots . . . 114
4.53.2 Condition dependent parameter changes . . . 114
4.54 Experiment: braun calibration hep 2014 01 27 IL6DR 24h Replicate3 . . . 115
4.54.1 Model fit and plots . . . 115
4.54.2 Condition dependent parameter changes . . . 115
4.55 Experiment: xiaoyun calibration APP tc replicate1 . . . 116
4.55.1 Comments . . . 116
4.55.2 Model fit and plots . . . 116
4.55.3 Observables . . . 117
4.55.4 Condition dependent parameter changes . . . 117
4.56 Experiment: xiaoyun calibration APP tc replicate2 . . . 118
4.56.1 Comments . . . 118
4.56.2 Model fit and plots . . . 118
4.56.3 Observables . . . 119
4.56.4 Condition dependent parameter changes . . . 119

4.57 Experiment: braun validation Ruxolitinib 1h hep 2013 10 14 qPCR 140224 IL6DR Inh 1h Cxcl10 . . . 120
4.57.1 Model fit and plots . . . 120
4.57.2 Condition dependent parameter changes . . . 120
4.58 Experiment: braun validation Ruxolitinib 1h hep 2014 04 28 IL6DR Rux 1h CXCL10 . . . 121
4.58.1 Model fit and plots . . . 121
4.58.2 Condition dependent parameter changes . . . 121
4.59 Experiment: braun validation Ruxolitinib 6h hep 2014 04 22 qPCR 140526 IL6DR Rux 6h APP . . . 122
4.59.1 Model fit and plots . . . 122
4.59.2 Condition dependent parameter changes . . . 122
4.60 Experiment: braun validation Ruxolitinib 6h hep 2014 05 19 qPCR 140526 IL6DR Rux 6h APP . . . 123
4.60.1 Model fit and plots . . . 123
4.60.2 Condition dependent parameter changes . . . 123
4.61 Experiment: braun validation Ruxolitinib 24h hep 2014 04 22 qPCR 140604 IL6DR Rux 24h APP . . . 124
4.61.1 Model fit and plots . . . 124
4.61.2 Condition dependent parameter changes . . . 124
4.62 Experiment: braun validation Ruxolitinib 24h hep 2014 05 19 qPCR 140604 IL6DR Rux 24h APP . . . 125
4.62.1 Model fit and plots . . . 125
4.62.2 Condition dependent parameter changes . . . 125
4.63 Experiment: APP validation 1h . . . 126
4.63.1 Model fit and plots . . . 126
4.63.2 Condition dependent parameter changes . . . 126
4.64 Experiment: braun validation Ruxolitinib triple 2015 04 13 triple treatment replicate1 . . . 127
4.64.1 Model fit and plots . . . 127
4.64.2 Condition dependent parameter changes . . . 128
4.65 Experiment: braun validation Ruxolitinib triple 2015 04 13 triple treatment replicate2 . . . 129
4.65.1 Model fit and plots . . . 129
4.65.2 Condition dependent parameter changes . . . 130
4.66 Experiment: braun validation Ruxolitinib triple 2015 04 13 triple treatment replicate3 . . . 131
4.66.1 Model fit and plots . . . 131
4.66.2 Condition dependent parameter changes . . . 132
4.67 Experiment: xiaoyun validation nucSTAT3 validation 20150428 nExpID1 . . . 133
4.67.1 Model fit and plots . . . 133
4.67.2 Observables . . . 133
4.67.3 Condition dependent parameter changes . . . 133
4.68 Experiment: xiaoyun validation nucSTAT3 validation 20150428 nExpID2 . . . 134
4.68.1 Model fit and plots . . . 134
4.68.2 Observables . . . 134
4.68.3 Condition dependent parameter changes . . . 134
4.69 Experiment: xiaoyun validation nucSTAT3 validation 20150508 nExpID1 . . . 135
4.69.1 Model fit and plots . . . 135
4.69.2 Observables . . . 135
4.69.3 Condition dependent parameter changes . . . 135
4.70 Experiment: xiaoyun validation nucSTAT3 validation 20150508 nExpID2 . . . 136
4.70.1 Model fit and plots . . . 136
4.70.2 Observables . . . 136
4.70.3 Condition dependent parameter changes . . . 136
4.71 Experiment: xiaoyun validation nucSTAT3 validation 20150528 nExpID1 . . . 137

4.71.1 Model fit and plots . . . 137
4.71.2 Observables . . . 137
4.71.3 Condition dependent parameter changes . . . 137
4.72 Experiment: xiaoyun validation nucSTAT3 validation 20150528 nExpID2 . . . 138
4.72.1 Model fit and plots . . . 138
4.72.2 Observables . . . 138
4.72.3 Condition dependent parameter changes . . . 138

5 Estimated model parameters **139**

Supplementary References **155**

1 Supplementary Experimental Procedures

1.1 Generation of mKate2-Stat3 Knock-in Mice

1.1.1 ESC Gene Targeting

40 µg gene targeting constructs were linearized with NotI in a total reaction volume of 200 µl. The successful linearization of gene targeting constructs was validated by gel electrophoresis with 0.5 % agarose gel at 30 V for 10 h at 4 °C. Successfully linearized gene targeting constructs were stored at −20 °C. ESC line JM8A3 was cultivated on neomycin resistant mouse embryonic fibroblasts that served as feeder cells. Linearized gene targeting constructs were thawed and precipitated with NaAc and ethanol one day before use for electroporation into ESC. Precipitated DNA was washed with 70 % ethanol three times and stored in last wash solution overnight at 4 °C. The next day ESC were washed with PBS two times and trypsinized with 1 ml trypsin (6-cm dish) for 2 min 37 °C. To obtain a single cell suspension, ESC were pippeted up and down gently and spinned down at 1000 ×g for 5 min.

At the day of electroporation, ESC cultivated in 10-cm dishes were washed for two times with PBS and then trypsinized with 2.5 ml trypsin for 5 min. 10 ml ESC medium was used to terminate trypsinization and obtain a single cell suspension by centrifugation at 1000 ×g for 5 min. The ESC pellet was resuspended with 0.5 to 1 ml PBS. The linearized DNA pellet was resuspended with 250 µl of PBS and mixed with 500 µl ESC in PBS in an electroporation cuvette. Electroporaton was performed with a gene pulser cuvette 0.4 cm electrode gap, 50 Biorad Electroporator 240 V, 500 µF, Time constant 5 to 7 s. The ESC were kept at room temperature for 20 min and diluted with ESC medium. Diluted cells were plated on 8 × 10-cm dishes with feeder cells. Selection with G418 and Ganciclovir was initiated 24 h after electroporation: 4 plates with 300 µg/µl G418 only and 4 plates with 300 µg/µl G418 and 255 µg/µl Ganciclovir. Media were refreshed every second day.

12 × 48-well plates with neomycin resistant feeder cells (500 µl/well) were prepared for later use. A total number of 288 clones were isolated with a 200 µl tip and transferred to one well of 48-well plate. All clones were kept in selective medium with G418. Around 12 days later, the clones were washed once with 500 µl PBS and trypsinized with 100 µl trypsin for 1.5 min at 37 °C. The clones were dissociated by pipetting up and down gently for 12 times with 200 µl tip. 800 µl ESC medium were used to terminate trypsinzation and cells were transferred to a new 48-well plate with MEFs as feeders. Media were refreshed every day (500 µl/well).

24-well plates with gelatin coating were prepared for all clones with 1 ml ESC medium. 48-well plates of ESC clones were processed when they were confluent. 1/3 of the ESC were transferred to one well of 24-well plate with gelatin coating and 2/3 of the ESC were frozen down. Each clone was trypsinized with 100 µl trypsin and resuspended with 200 µl Solution A (70 % ESC medium and 30 %). 100 µl of the total 300 µl cell suspension were transferred to one well of 24-well plate for DNA isolation. All 24-well plates were incubated at 37 °C with 5 % CO_2. 48-well plates with 2/3 of ESC were incubated on ice for several minutes and frozen down by adding 200 µl of Solution B (50 % ESC medium, 30 % FCS and 20 % DMSO). All 48-well plates with 2/3 of each clone were sealed with tape and wrapped in plastic. ESC clones were frozen at −80 °C.

ESC in 24-well plate were washed once with PBS and lysed with 500 µl lysis buffer. Cells were incubated with lysis buffer in the incubator overnight. 500 µl lysate was transferred with a 1 ml tip to 1.5 ml Eppendorf tube and mixed with 250 µl NaCl solution (5 mol/l) by shaking vigorously for around 1 min. Cellular debris was removed by centrifugation at 20 000 ×g. 550 µl supernatant was mixed with 450 µl isopropanol to precipitate DNA. Precipitated DNA was washed with 500 µl 70 % ethanol. The pellet was dried on the bench for 2 to 3 min min and dissolved in 200 µl TE.

1.1.2 Blastocyst Injection and Embryo Transfer

For each positive ESC clone, around 45 blastocysts were used for injection. For each blastocyst, around 10 to 15 ESC were injected. Those 45 injected blastocysts were transferred to three foster mothers.

1.2 Genotyping PCR

Genotyping PCR for mKate2-Stat3 was performed with a 3-primer strategy (B7, F5 and D2), using one forward primer located on the wild type genomic DNA, one reverse primer located on the wild type genomic DNA and an additional forward primer located within the mKate2 coding sequence. Genotyping PCR for removal of selection cassette was performed with 2-primer PCR (G2 and B8). Example PCR results can be seen in Figure S11.

Table S1: Genotyping primers

Name	Sequence
B7-forward	TCTGTTCTCCCAATCCTTCC
F5-forward	GCGGGTTTCTTGGATCTGTA
D2-reverse	taccgGAATTCGTTTGATCCCCAGCATCA
G2-forward	CTCTCTCCCATGTGAAGCCC
B8-reverse	GGCTAGCCTGAGACCCTGTT

1.3 Southern Blotting

Southern blotting was performed with a non-radioactive method using probes directly labeled with Alk-phosphatase. Genomic DNA was extracted using standard procedure and digested with restriction enzymes overnight. DNA was digested with EcoR V and Kpn I and separated by electrophoresis with 0.6 % agarose gel and transferred to a positively charged nitrocellulose membrane. Specific probes were labeled with Alk-phosphotase (RPN3680, GE Healthcare) and used for hybridization. CDP-STAR (RPN3682, GE Healthcare) was used for development and ImageQuant LAS 4000 (GE Healthcare) used to acquire luminescent signal after development.

1.4 AAV Vector Production

AAV vectors were produced using a standard triple transfection protocol as described previously [1]. Briefly, an AAV helper plasmid encoding AAV rep and cap genes, an AAV vector plasmid carrying the transgene as well as an adenoviral helper plasmid were transfected into HEK293T cells. The cap gene in the AAV helper was derived from wild type AAV serotype 9 through insertion of a short DNA oligonucleotide encoding a heptamer peptide. Human Histone2B was kindly provided by Jörg Langowski and mCerulean was kindly provided by David W. Piston. The hH2B-mCerulean cassette was amplified by PCR, digested with Age I and Avr II restriction enzymes and ligated into the AAV vector plasmid. The AAV vector plasmid expressing a fusion of H2B and mCerulean under the control of a cytomegalovirus (CMV) promoter was based on pSSV9, a plasmid that is routinely used for generation of single-stranded AAV vectors. For the triple transfection, ten 15 cm^2-dishes with 4×10^6 HEK293T cells per dish were seeded two days prior to transfection. Using polyethylenimine (PEI) as transfection reagent, the cells were then triple-transfected with 14.6 µg of each plasmid per dish. 43.8 µg total DNA were diluted in DMEM without any supplements, mixed with 140 ul of PEI (1 mg/ml in H_2O), and incubated for at least 30 min at room temperature. This mixture was next added dropwise to the cells and incubated at 37 °C for three days, before the cells were harvested into the medium and pelleted at 1200 rpm for 15 min. After one wash step with PBS, the pellet was resuspended in 6 ml lysis buffer (50 mmol/l Tris-HCl pH 8.5, 50 mmol/l $NaHCO_3$) and subjected to five rounds of freezing and thawing (−80/37 °C). Following digestion of the cell lysate with 50 U/l benzonase for 1 h at 37 °C, cell debris was removed by centrifugation at 3750 rpm for 20 min. For purification, the virus-containing lysate was added to a preformed gradient of 15 %, 25 %, 40 % and 60 % iodixanol (OptiPrep in PBS-MK; PBS with 1 mmoll $MgCl_2$, 2.5 mmoll KCl) and centrifuged in a 70.1 Ti rotor (Beckman Coulter) at 50 000 rpm and 4 °C for 2 h. Using needle and syringe, purified viral particles were retrieved from the 40 % phase and stored in 50 µl aliquots at −80 °C. A vector titer of 9.17×10^{10} genome copies per ml was determined using standard RT-PCR.

1.5 Live Cell Imaging of mKate2-STAT3

Primary hepatocytes were seeded at 15000 cells/well into 96-well plate pre-coated with collagen I in adhesion medium. 4 hours after seeding, media were changed to pre-starvation medium and hepatocytes were pre-starved overnight (15 to 16 hours). On the next day, hepatocytes were starved for 5 hours before pre-treatment with DMSO or Ruxolitinib. Serially diluted doses of IL-6 were applied after 1 hour of pre-treatment with DMSO or Ruxolitinib. STAT3 dynamics were monitored before inhibitor pre-treatment, after inhibitor pre-treatment and after IL-6 stimulation.

Hepatocytes were imaged with Nikon Eclipse Ti Fluorescence microscope controlled with NIS-Elements software. Temperature (37 °C), CO_2 (5 %) and humidity were held constant through an incubation chamber enclosing the microscope and a 96-well plate stage insert. Three channels were acquired for each position: brightfield channel, STAT3 channel (mKate2) and nuclear channel (CFP channel).

Time-lapse live cell microscopy was performed with Nikon Ti-HCS screening microscope. Nikon Ti-HCS is a Nikon ECLIPSE Ti microscope that was equipped with okolab incubation chamber and fully automated xyz stage controller. Image acquisition was done with Andor Clara camera (model DR-328-C01-SIL, OXFORD INSTRUMENTS), which is a high-sensitive interline-CCD-camera with 1392 × 1040 pixel resolution. Each pixel has a size of 6.45 × 6.45 µm. 20× air objective (NA = 0.75) was used.

1.6 Generation of Recombinant Calibrator Proteins

Glutathione S-transferase (GST) or streptavidin binding peptide (SBP)-tagged murine proteins were used for normalization of immunoblot data and as reference for the determination of molecules per cell. gp130 (cytoplasmic domain) and STAT3 were cloned into pGEX-2T (GE Healthcare) and SOCS3 was cloned into pGEX-2T derived vector pSBPEX. The resulting GST-gp130, GST-STAT3 and SBP-SOCS3 were expressed in *Escherichia coli* BL21(DE3) CodonPlusRIL (Stratagene) and extracted by lysozyme lysis and sonication. Glutathione sepharose beads or streptavidin sepharose beads were added to lysates and bound recombinant proteins were eluted by addition of reduced glutathione or biotin for GST- or SBP-tagged proteins, respectively. For quantification of purified proteins, dilution series of purified bovine serum albumin and recombinant proteins were separated by 10 % SDS-polyacrylamide gel electrophoresis and stained with Coomassie Brilliant Blue. The gel was documented using the trans-illumination mode of LumiImager F1 and band quantification was achieved with LumiAnalyst software (both Roche).

1.7 Preparation of total cell lysate from primary human hepatocytes

After appropriate treatment times 2×10^6 primary human hepatocytes were lysed in 300 µl RIPA lysis buffer (50 mM Tris pH 7.2, 250 mM NaCl, 2 % (v/v) NP40, 2.5 mM EDTA, 0.1 % (w/v) SDS, 0.5 % (w/v) sodium deoxycholate (Serva), 0.1 mg/ml AEBSF, 1 mg/ml aprotinin, 1 % (v/v) 1 M Na_3VO_4, 2 % (v/v) 0.5 M NaF) and sonicated on ice (30 s, 75 % amplitude, 0.1 s on, 0.5 s off). Subsequently, samples were centrifuged 10 min at 14 000 rpm at 4 °C and supernatants were transferred into a fresh tube.

1.8 Quantitative real-time PCR

Reverse transcribed cDNA was analyzed using the Universal Probe Library System on a LightCycler 480 with the cycling conditions as shown in Table S2. Primer design was performed using the Universal Probe Library Assay Design Center. Primers and corresponding Universal Probe Library (UPL) probes are listed in Table S3.

Table S2: Cycling conditions for quantitative real-time PCR

Program Name	Pre-Incubation		
Cycles	1	Analysis Mode	None
Target (°C)	Acquisition Mode	Hold (hh:mm:ss)	Ramp Rate (°C/s)
95	None	00:05:00	4.40
Program Name	Amplification		
Cycles	50	Analysis Mode	Quantification
Target (°C)	Acquisition Mode	Hold (hh:mm:ss)	Ramp Rate (°C/s)
95	None	00:00:10	4.40
60	None	00:00:30	2.20
72	Single	00:00:01	4.40
Program Name	Cooling		
Cycles	1	Analysis Mode	None
Target (°C)	Acquisition Mode	Hold (hh:mm:ss)	Ramp Rate (°C/s)
40	None	00:02:00	1.50

Table S3: Primers and probes used for quantitative real-time PCR

**Gene**	**Species**	**UPL Probe**	**Forward primer (5'-3')**	**Reverse primer (5'-3')**
Apcs	mouse	15	AGCCCCACCCAGTATAGTCC	CTTTGAAACCCTCCTCCGTA
Cxcl10	mouse	3	GCTGCCGTCATTTTCTGC	TCTCACTGGCCCGTCATC
Fgg	mouse	56	TGAAGGAAACCACCATGAAGA	GCAAACCAGCTTTGAAATGTT
Hamp	mouse	63	GATGGCACTCAGCACTCG	GCTGCAGCTCTGTAGTCTGTCT
Hp	mouse	15	GGCAAGAGAGGTCCACGAT	CCACAGCAAAAAGCTGACC
Hpx	mouse	109	GTGCCACCTATGCCTTCAC	CCAGCTATGCCATCCATCA
Il33	mouse	71	GGTGAACATGAGTCCCATCA	CGTCACCCCTTTGAAGCTC
Socs3	mouse	25	GCTGGTACTGAGCCGACCT	TTCCGACAAAGATGCTGGA
Hprt	mouse	95	TCCTCCTCAGACCGCTTTT	CCTGGTTCATCATCGCTAATC
Tbp	mouse	97	GGGAGAATCATGGACCAGAA	GATGGGAATTCCAGGAGTCA
CRP	human	18	GAATTCAGGCCCTTGTATCACT	ACACAAAAGCCTTCCTCGAC
FGG	human	19	GAAGGACAGCAACACCACCT	GTAAATCTCTTTTGAAACGGTCTTTTA
SOCS3	human	36	AGACTTCGATTCGGGACCA	AACTTGCTGTGGGTGACCA
HAMP	human	36	GACCAGTGGCTCTGTTTTCC	TCTGGAACATGGGCATCC
HP	human	86	TGGAGTGTACACCTTAAACAATGAG	TTCTTGGGCTTCCCACATAC
HPRT	human	73	TGACCTTGATTTATTTTGCATACC	CGAGCAAGACGTTCAGTCCT
TBP	human	3	CGGCTGTTTAACTTCGCTTC	CACACGCCAAGAAACAGTGA
IL-11	human		CGCTGGGACATTGGGATCTTT	CTTTAGGGAAGGACCACCTGC
IL-22	human		TCCAGCAGCCATACATCGTC	CCTCGGAACAGTTTCTCCCC
IL-6	human		GTTCTCTGGGAAATCGTGGA	GATTGTTTTCTGCAAGTGCATC
OSM	human		TACCTGAGCCCACACAGACA	CGATGGTATCCCCAGAGAAA

2 Supplementary Figures

2.1 Figure S1

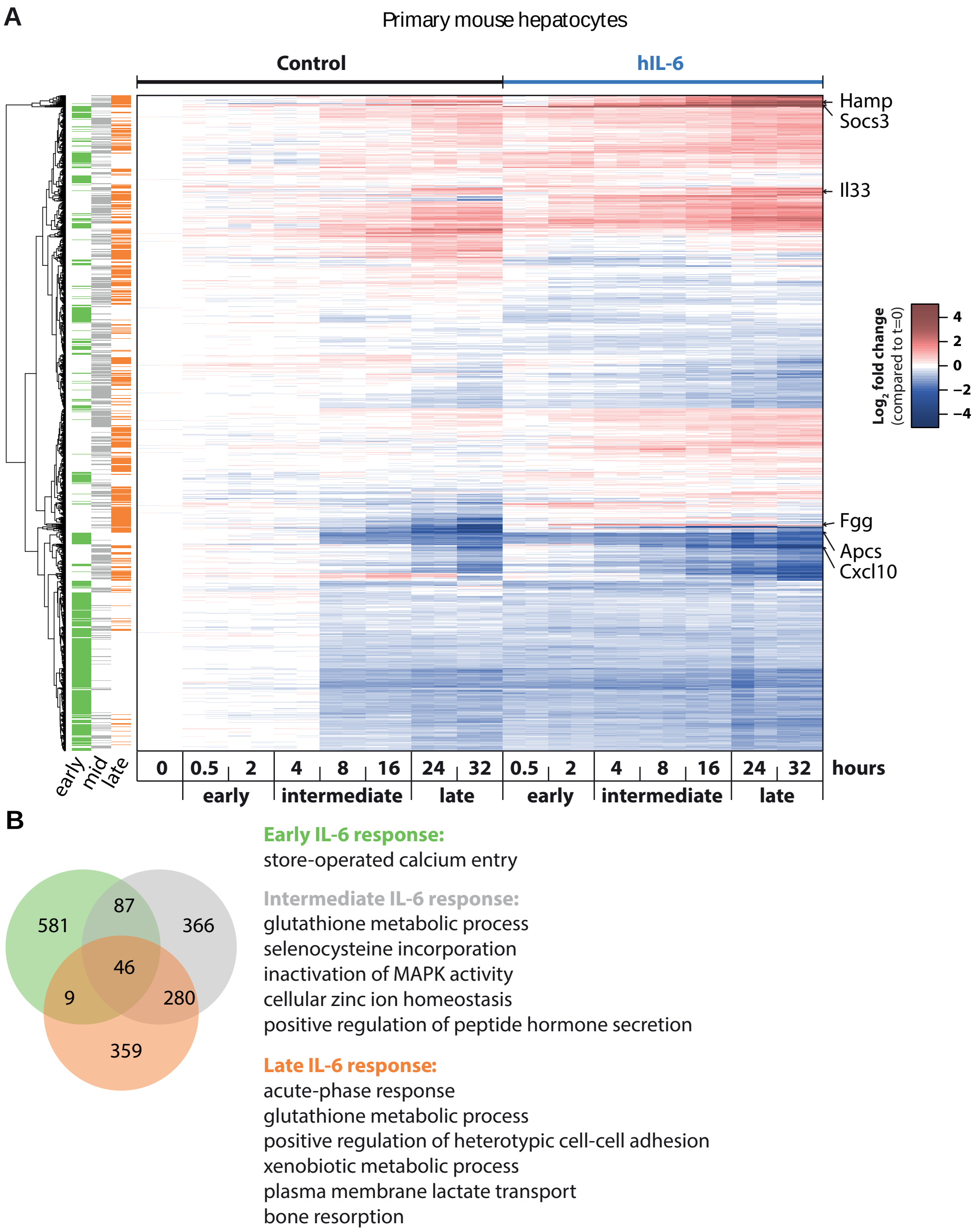

Figure S1: (**A**) Genome-wide analysis of IL-6-induced transcriptional responses. Hierarchical clustering of expression profiles of significantly IL-6-regulated genes. Primary mouse hepatocytes were stimulated with 40 ng/ml hIL-6 or left untreated (Control), and RNA was isolated at indicated time points. Transcriptome profiling was performed as described in Figure 2. Expression values are centered to mean expression of t=0 Control samples. (**B**) Venn diagram shows numbers of genes overlapping in their IL-6-temporal regulation. Results of ontology analysis are listed for each response class.

2.2 Figure S2

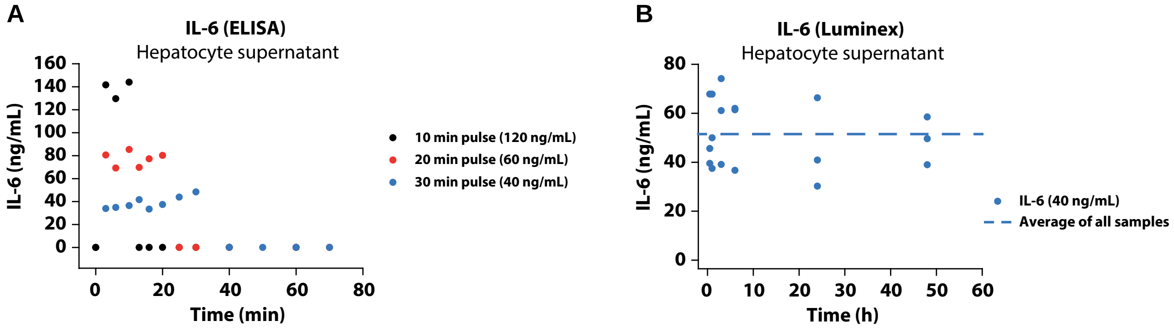

Figure S2: (**A**) Primary mouse hepatocytes were pulse-stimulated with indicated concentrations of hIL-6. At respective time points, IL-6 concentrations in hepatocyte medium were measured by ELISA. (**B**) Primary mouse hepatocytes were stimulated with 40 ng/ml of hIL-6 (continuous). At indicated time points up to 48 h, IL-6 concentrations in medium were analyzed by a bead-based immunoassay. The experiment was performed in three biological replicates, the average of all samples is indicated (dashed line).

2.3 Figure S3

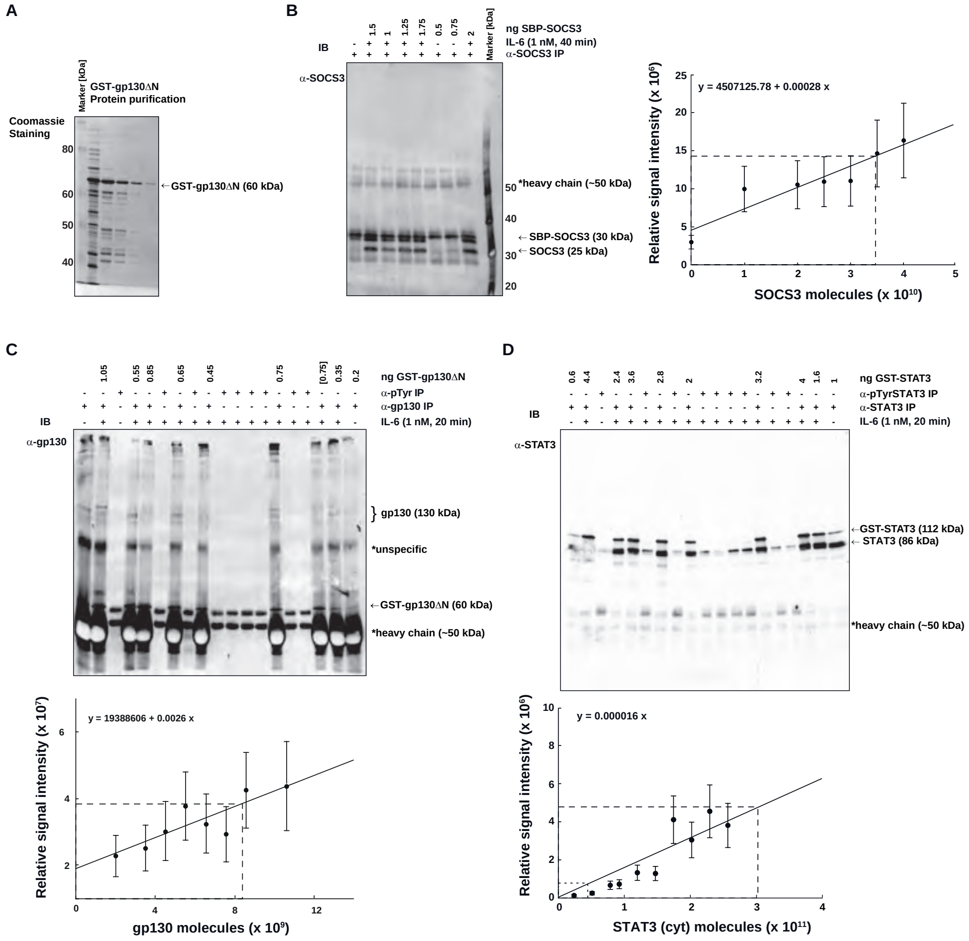

E

	Molecules per cell	Concentration (nM)
gp130	4680 +/- 2908	0.61 +/- 0.38
STAT3	215370 +/- 32393	28.23 +/- 4.25
SOCS3	22630 +/- 7897	2.97 +/- 1.04

Figure S3: (**A**)Coomassie-stained gel of protein fractions after purification and size determination of GST-gp130 ΔN. As described in the Supplementary Materials section 1.6, gp130 was N-terminally truncated. Unpurified bacterial lysate (left) followed by sequential protein fractions from the purification process. Position of the molecular weight markers are indicated in kDa (**B-D**)Cytoplasmic lysates of $2{\times}10^6$ primary mouse hepatocytes were spiked with indicated amounts of recombinant GST- or SBP-tagged calibrator proteins to determine absolute protein amounts of SOCS3 (**B**), gp130 (**C**) and STAT3 (**D**). Protein levels were determined by immunoprecipitation (IP) and subsequent quantitative immunoblotting (IB). Fusion proteins were added at the indicated quantities to the cellular lysates. Anti-phosphoTyr immunoprecipitation was attempted to determine the position of tyrosine phosphorylated gp130 in the blot, but failed since no band was detected by the anti-gp130. Marked by an asterisks is a prominent unspecific band that appeared in all anti-gp130 immunoprecipitation reactions and was not changed by IL-6 stimulation. Signals of the recombinant calibrator proteins were fitted by a linear regression function (solid lines). The amount of calibrator protein added is indicated and samples that were not utilized for the generation of the standard curve are indicated by squared brackets. Error bars of calibrator signals represent 20 % relative error. Absolute protein amounts (dashed lines) were calculated by means of the respective regression functions. Quantification results are summarized in (**E**).

2.4 Figure S4

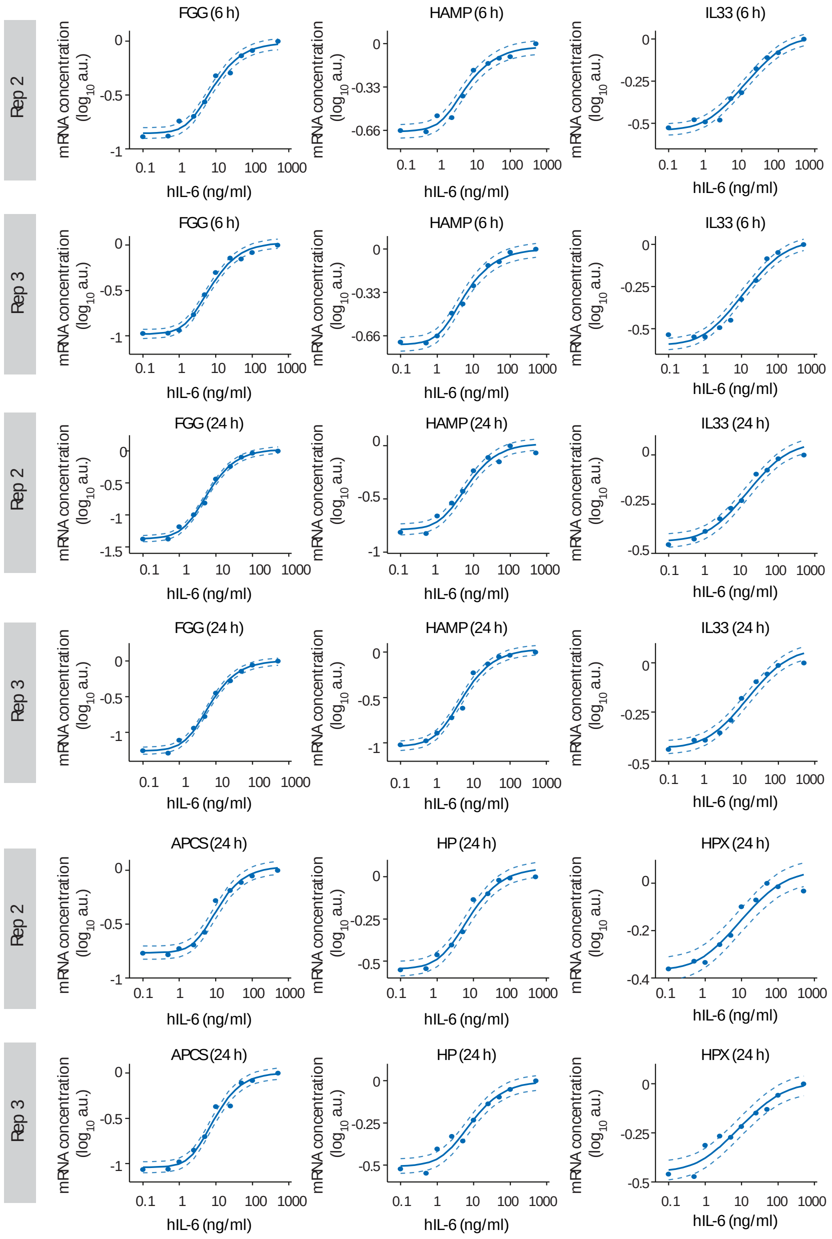

Figure S4: Additional replicates related to Figure 5. Primary mouse hepatocytes were treated with hIL-6 stimulation or were left untreated. Dose-dependent analysis of target gene expression was performed applying hIL-6 concentrations ranging from 0.1 to 500 ng/ml. At indicated time points, target mRNAs were quantified by qPCR. Filled circles: experimental data; solid lines: model trajectories. Dashed lines indicate the measurement noise as estimated by the error model. a.u.: arbitrary units.

2.5 Figure S5

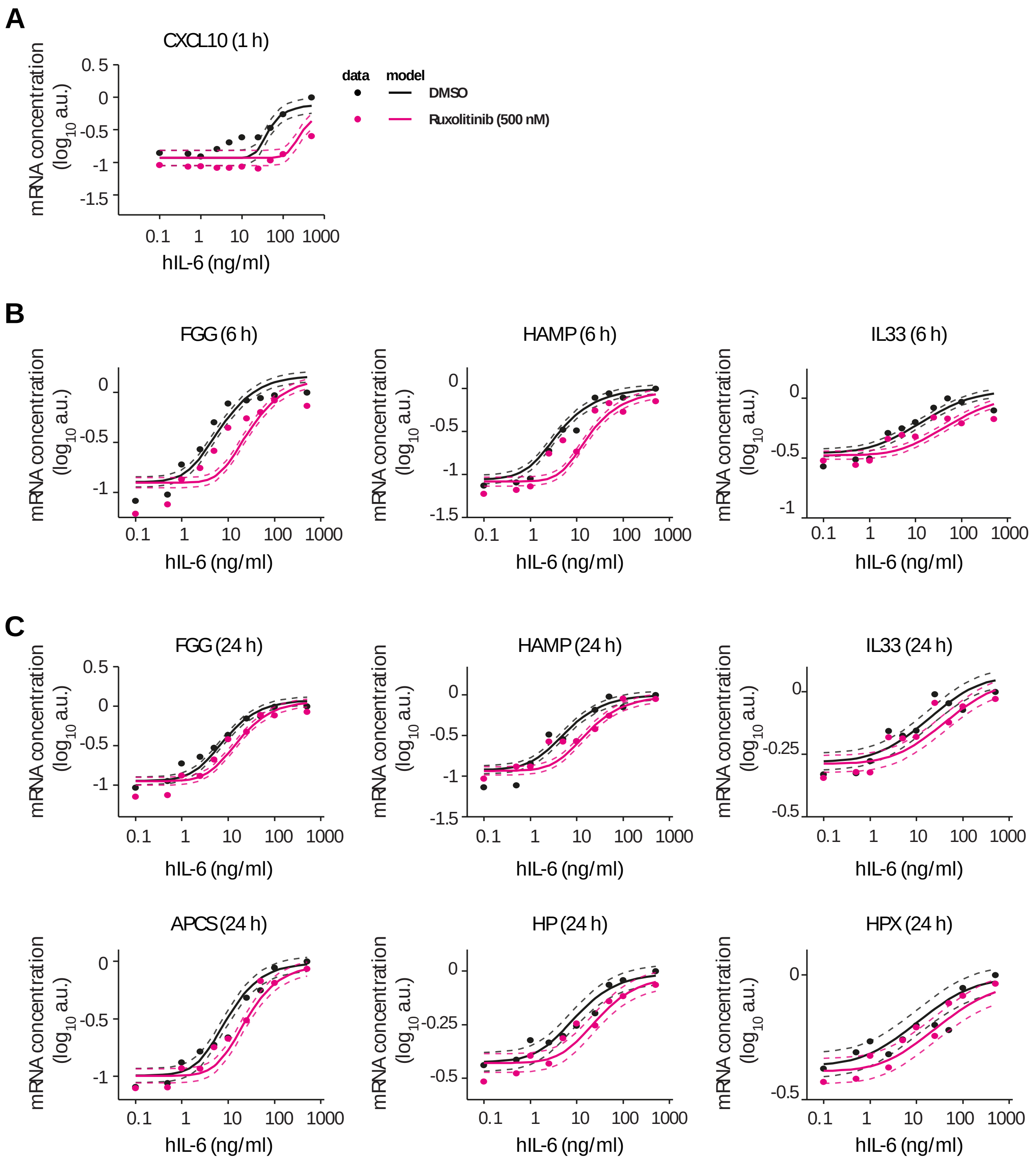

Figure S5: An individual replicate related to Figure 6. Solid lines represent model predictions for dose-dependent APP gene expression with or without Ruxolitinib pre-treatment. For experimental validation (filled circles), primary mouse hepatocytes were pre-treated with Ruxolitinib or DMSO control for 1 h prior to hIL-6 (ranging from 0.1 to 500 ng/ml) stimulation. APP mRNA expression was quantified at indicated time points by qPCR. Dashed lines indicate the measurement noise as estimated by the error model. a.u.: arbitrary units.

2.6 Figure S6

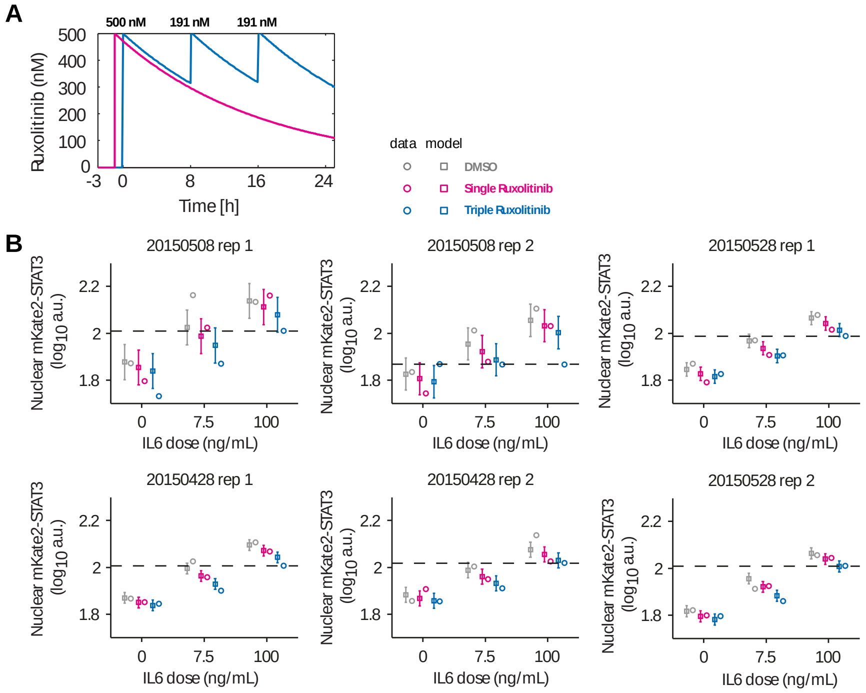

Figure S6: All replicates related to Figure 7 (n=6). Primary mouse hepatocytes were used for DMSO, single or triple Ruxolitinib dosing followed by stimulation of indicated doses of hIL-6 and time-lapse microscopy was employed to measure nuclear STAT3. Data and model predictions are shown as circles and squares, respectively.

2.7 Figure S7

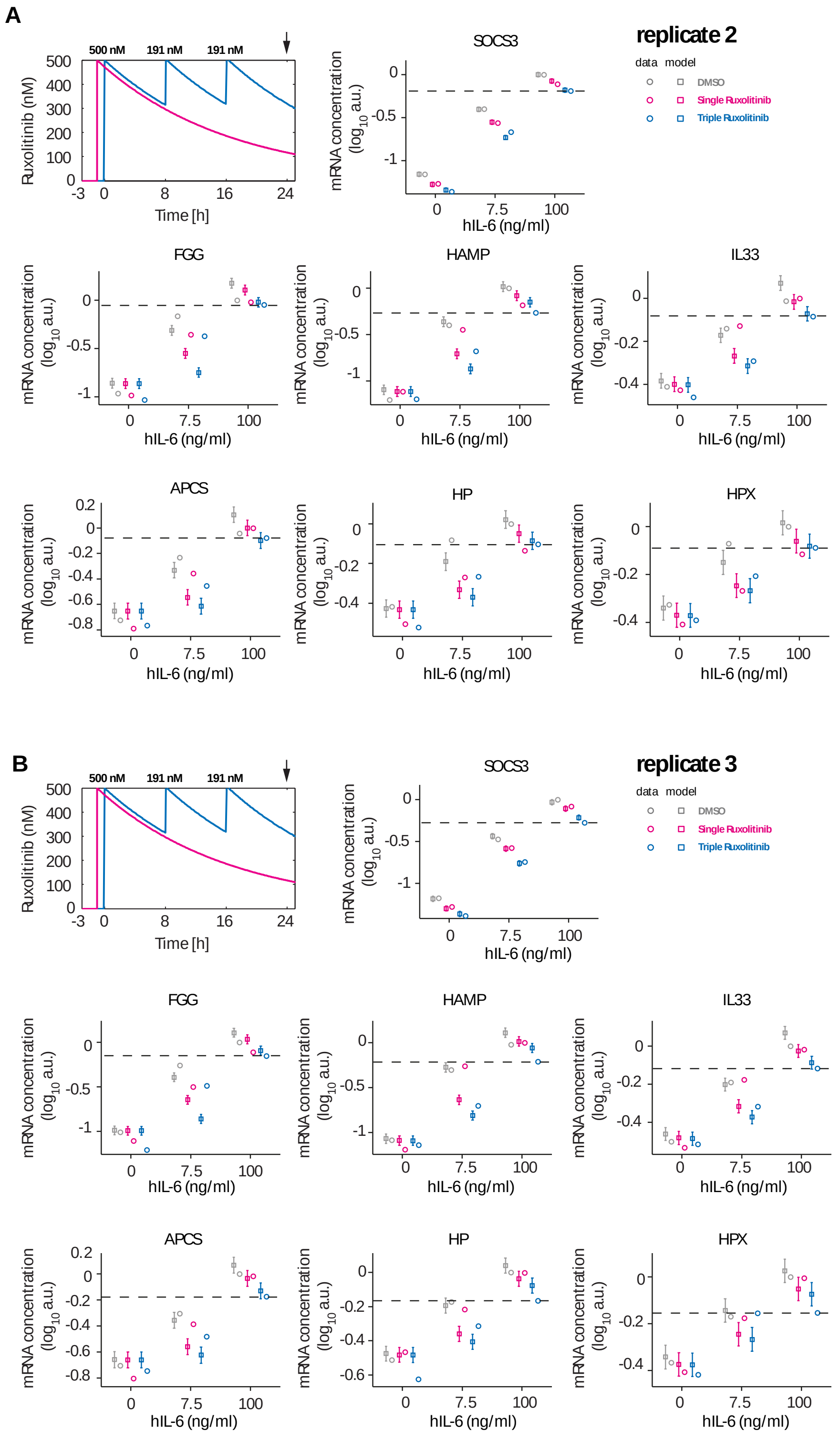

Figure S7: Two individual replicates for Figure 8. Primary mouse hepatocytes were used for DMSO, single or triple Ruxolitinib dosing and qRT-PCR was employed to measure gene expression after 24 h. Data and model predictions are shown as circles and bars, respectively.

2.8 Figure S8

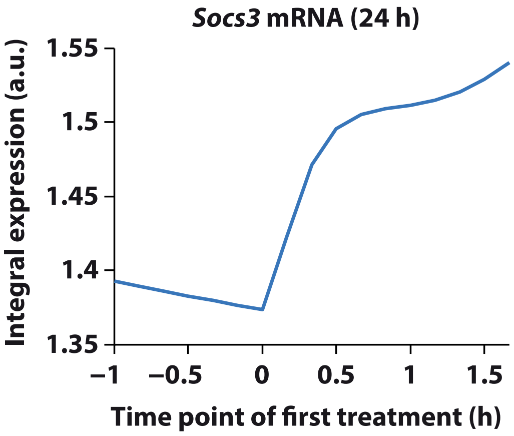

Figure S8: The solid line indicates the model-predicted integral *Socs3* mRNA expression at time point 24 h depending on the time point of first Ruxolitinib (500 nmol/l) treatment. Time points of the second and third Ruxolitinib treatment (191 nmol/l each, to replenish 500 nmol/l) were fixed to achieve optimal repression (8 h and 16 h).

2.9 Figure S9

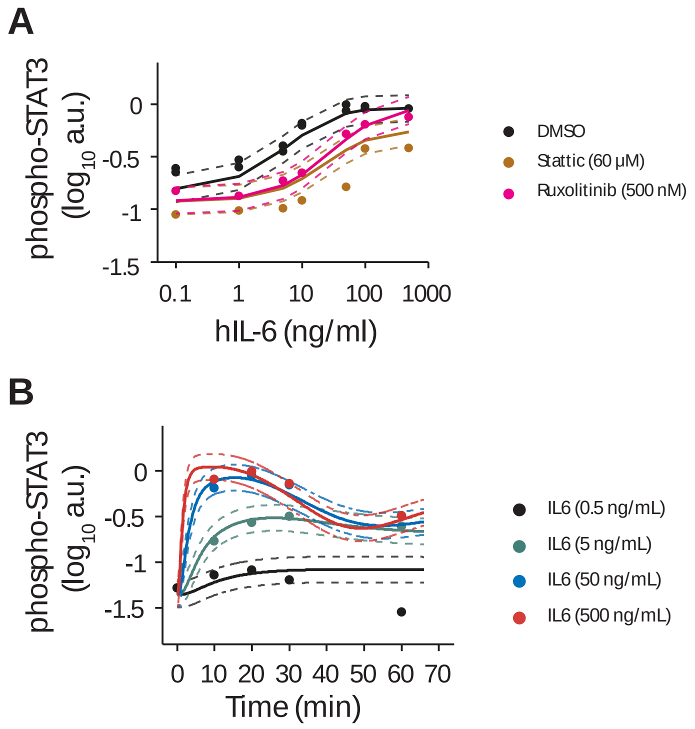

Figure S9: (**A**) Dose response of hIL-6 induced STAT3 phosphorylation at t= 20 min treatment with IL-6 and 1 h pre-treatment with the indicated inhibitor in primary murine hepatocytes and (**B**) time course of hIL-6 induced STAT3 phosphorylation in primary murine hepatocytes.

2.10 Figure S10

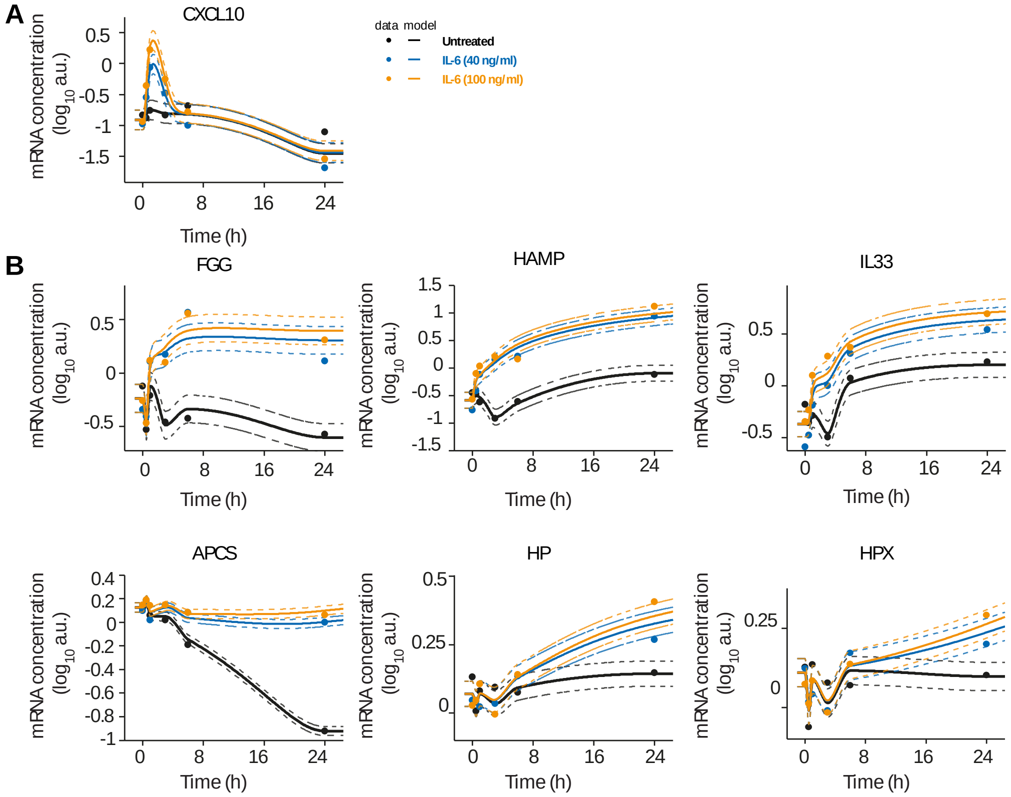

Figure S10: An individual replicate for Figure 4. Primary mouse hepatocytes were stimulated with hIL-6 or left untreated and RNA was isolated at indicated time points. Target mRNA expression was analyzed by quantitative real-time PCR (qPCR) and data were normalized to the geometric mean [2] of *Hprt* and *Tbp* expression. Filled circles represent data points; solid lines connect average values; dashed lines indicate measurement noise as estimated by the model.

2.11 Figure S11

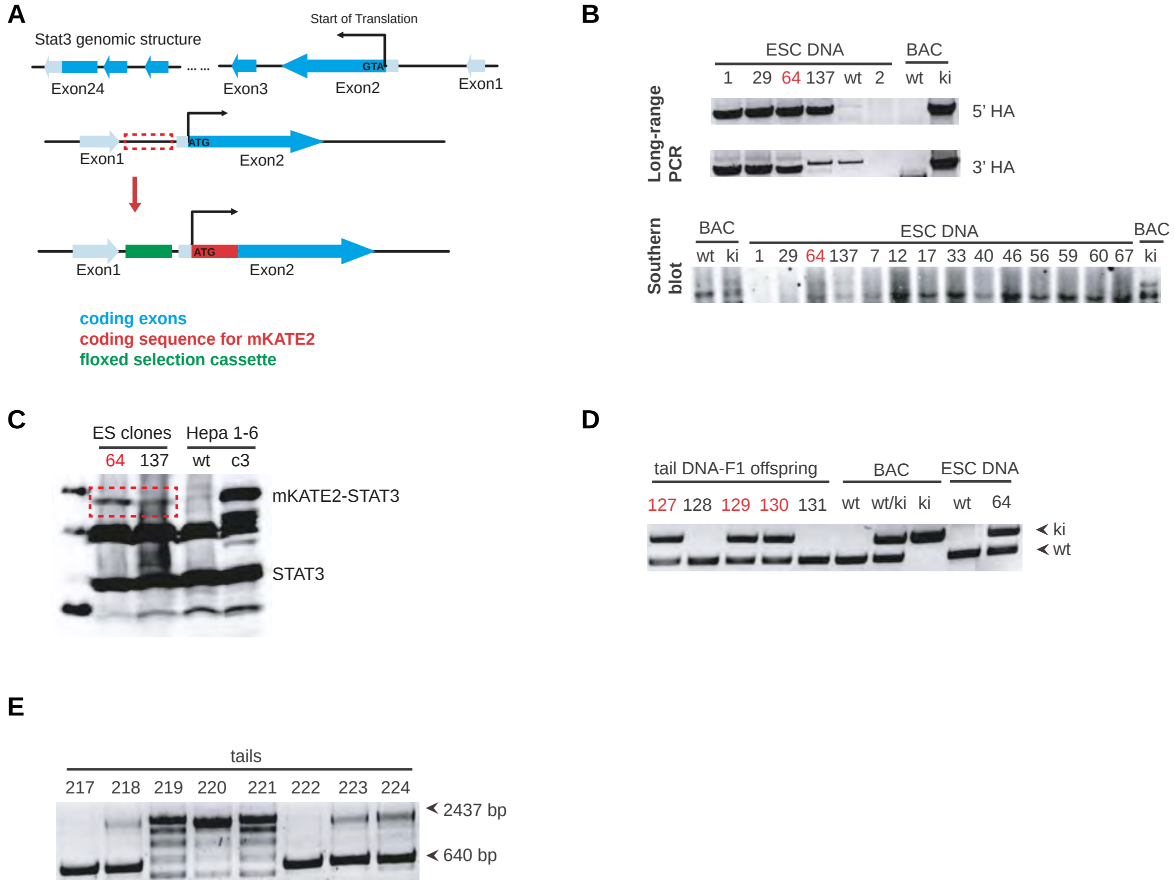

Figure S11: Generation of mKate2-Stat3 knock-in reporter mice. (**A**) Schematic depiction of the gene targeting strategy. Dark blue boxes indicate the coding exons of *Stat3*, light blue boxes indicate the untranslated exons, red box indicates the coding sequence for mKate2, and green box indicates the neomycin selection cassette (Neo) floxed by loxP sites. mKate2 coding sequence is integrated right before the first coding exon of *Stat3* to enable the expression of N-terminally tagged STAT3 fusion protein. Dashed rectangle box indicates the targeting locus. (**B**) ESC clones were screened with long-range PCR. PCR products from long-range PCR were subjected to agarose gel electrophoresis using 1% agarose gel that was stained with midori green DNA dye. The gel was documented with GelDoc using UV illumination. For Southern blot analysis for each sample 10 μg DNA was digested using EcoRV and KpnI and separated with 0.6 % agarose gel. Southern blot detection was performed with a non-radioactive techinique using Alk-phosphatase labeled probes. Chemiluminescent signals were detected with ImageQuant. HA: homologous arm; ki: knock-in; wt: wild type. (**C**) Gene targeted ESC clones 64 and 137 were lysed with RIPA total cell lysis buffer and cell lysates were seprated by 10 % SDS-PAGE. Immunoblotting was performed with anti-STAT3 antibody. Chemiluminescent signals were detected with ImageQuant. Total cell lysates from wild type and *mKate2-Stat3* BAC transgenic Hepa1-6 clone 3 (C3) were included as negative and positive control. The red rectangle marks the knock-in allele. Clone 64 indicated by red was chosen for further experiments. (**D**) Germline transmission of gene targeted ESC clone 64 identified by PCR-based genotyping. PCR products were separated by a 1 % agarose gel that was stained with midori green DNA dye. The gel was documented with GelDoc using UV illumination. DNA from bacterial artifical chromosome (BAC) and embryonic stem cells (ESC) were used as controls. Offsprings harboring the knock-in allele are indicated by red color. The image represents one litter having both agouti and black offsprings. (**E**) The Neomycin selection cassette was removed by breeding with Cre deleter mouse, and offsprings were screened with PCR-based genotyping. PCR products were separated by agarose gel electrophoresis and stained with midori green DNA dye.

2.12 Figure S12

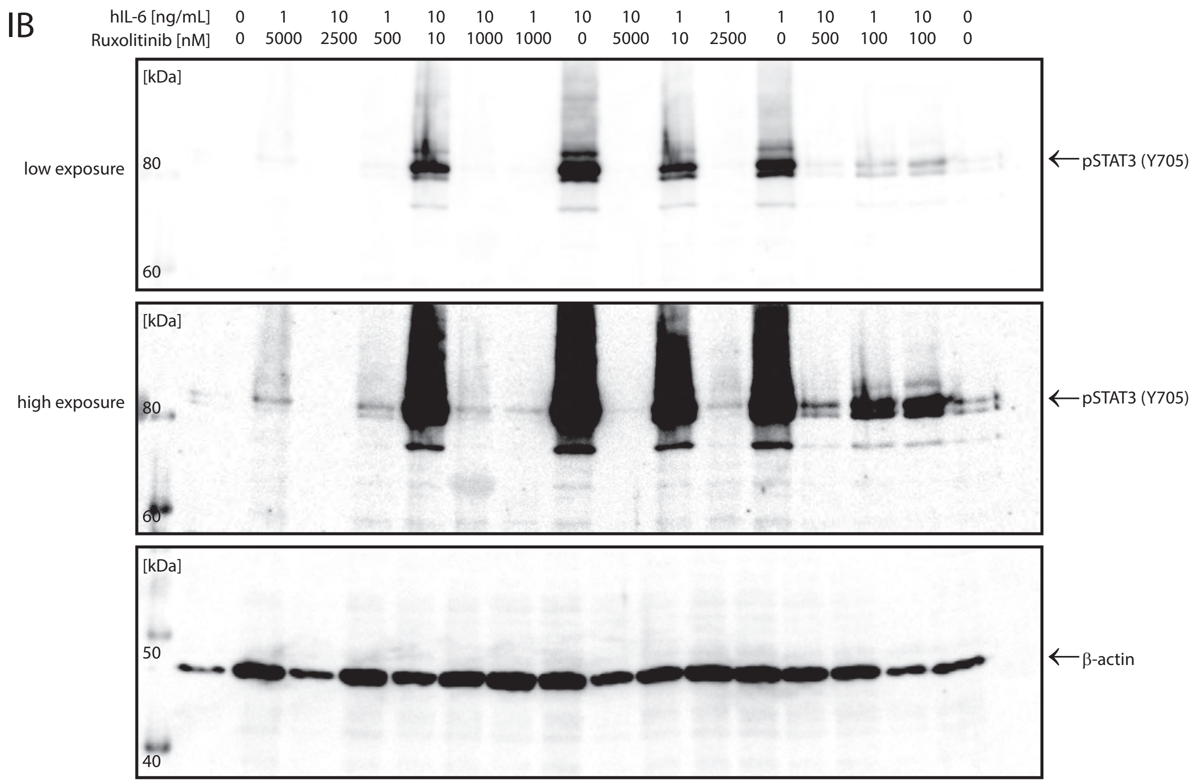

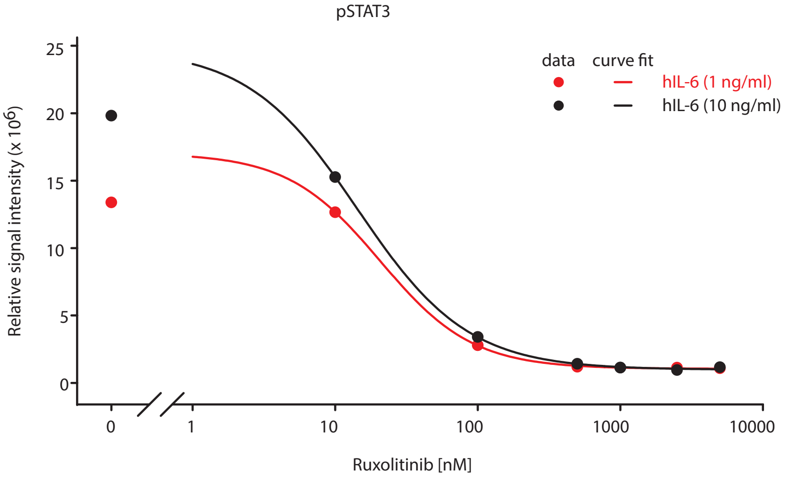

Figure S12: 2×10^6 primary human hepatocytes were co-treated with varying doses of Ruxolitinib and either 1 or 10 ng/ml hIL-6. Total cell lysates were prepared after 20 min total treatment time and subjected to quantitative immunoblotting to detect phosphorylated STAT3 (Y705) and β-actin. Four parameter logistic curve fit was conducted with SigmaPlot 13.0 (Systat Software, Inc).

2.13 Figure S13

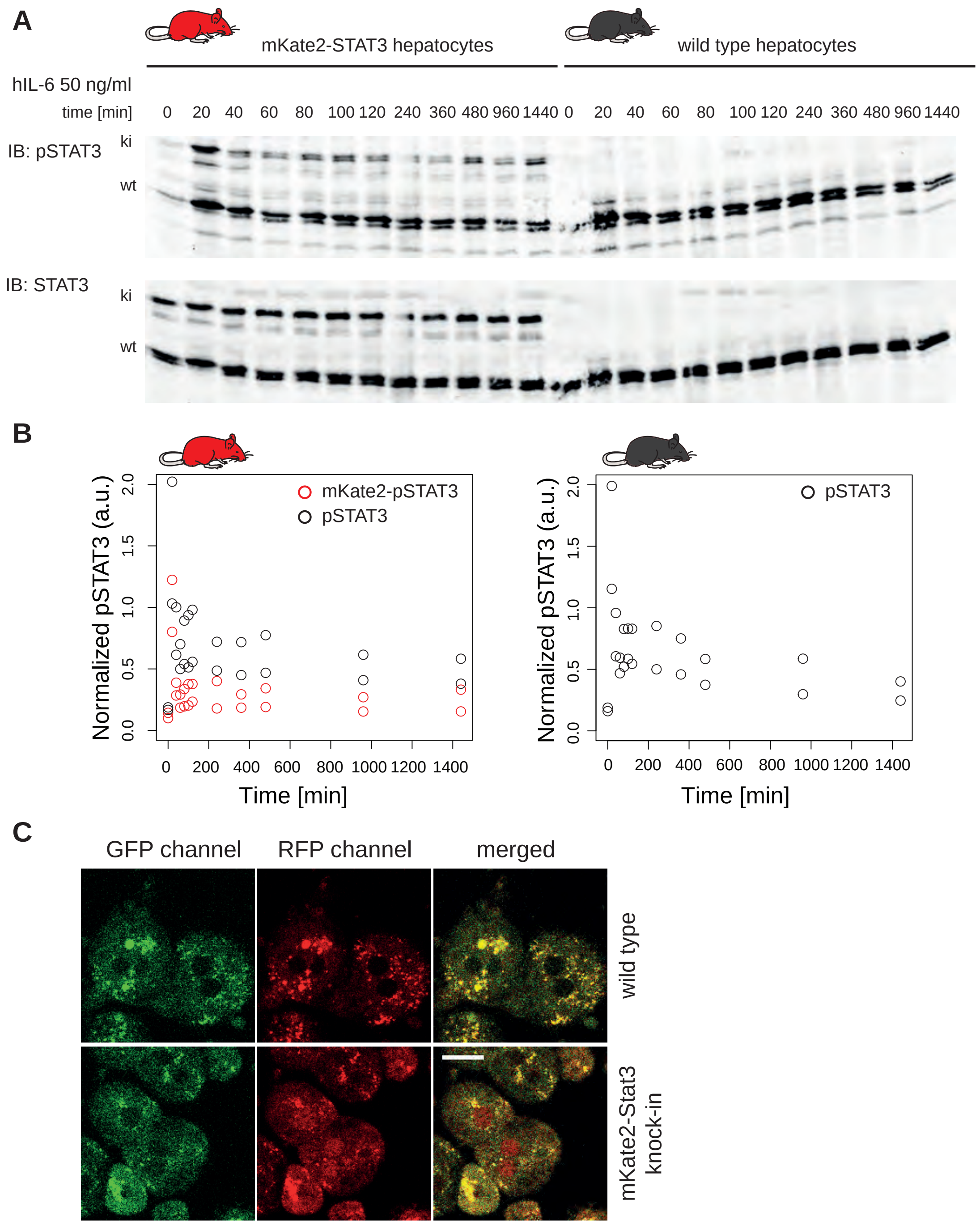

Figure S13: (**A**) Primary mouse hepatocytes were isolated from both wild type and Stat3 knock-in mice and 2×10^6 cells were seeded per 6-cm dish, were permitted to adhere for four hours, and were serumdepleted overnight. Six hours after serum and dexamethasone depletion, cells were stimulated with 50 ng/ml hIL-6 and lysed with RIPA total cell lysis buffer at indicated time points. Protein lysates were separated by SDS-PAGE and immunoblotting was performed with anti-phosphorylated STAT3 (Y705) and anti-STAT3 antibody. Fluorescent dye-conjugated secondary antibodies were used to amplify the signal and the Odyssey system was employed to detect fluorescent signals. (**B**) Quantification of Y705 phosphorylation signals of STAT3 shown in A. Phosphorylated STAT3 signals were normalized to total STAT3 signals. Normalized pSTAT3 data were plotted (n=2). (**C**) Primary mouse hepatocytes were isolated from both wild type and mKate2-Stat3 knock-in mice and seeded in Collagen IV-coated 8-well ibidi plates (2×10^4 cells/well). Four hours after adhesion, the hepatocytes were subjected to confocal microscopy to measure the expression and localization of mKate2-STAT3. Shown are representative images of hepatocytes with mKate2-STAT3 localized to the nuclei from two experiments performed independently (n=2). The scale bar is 50 µm.

2.14 Figure S14

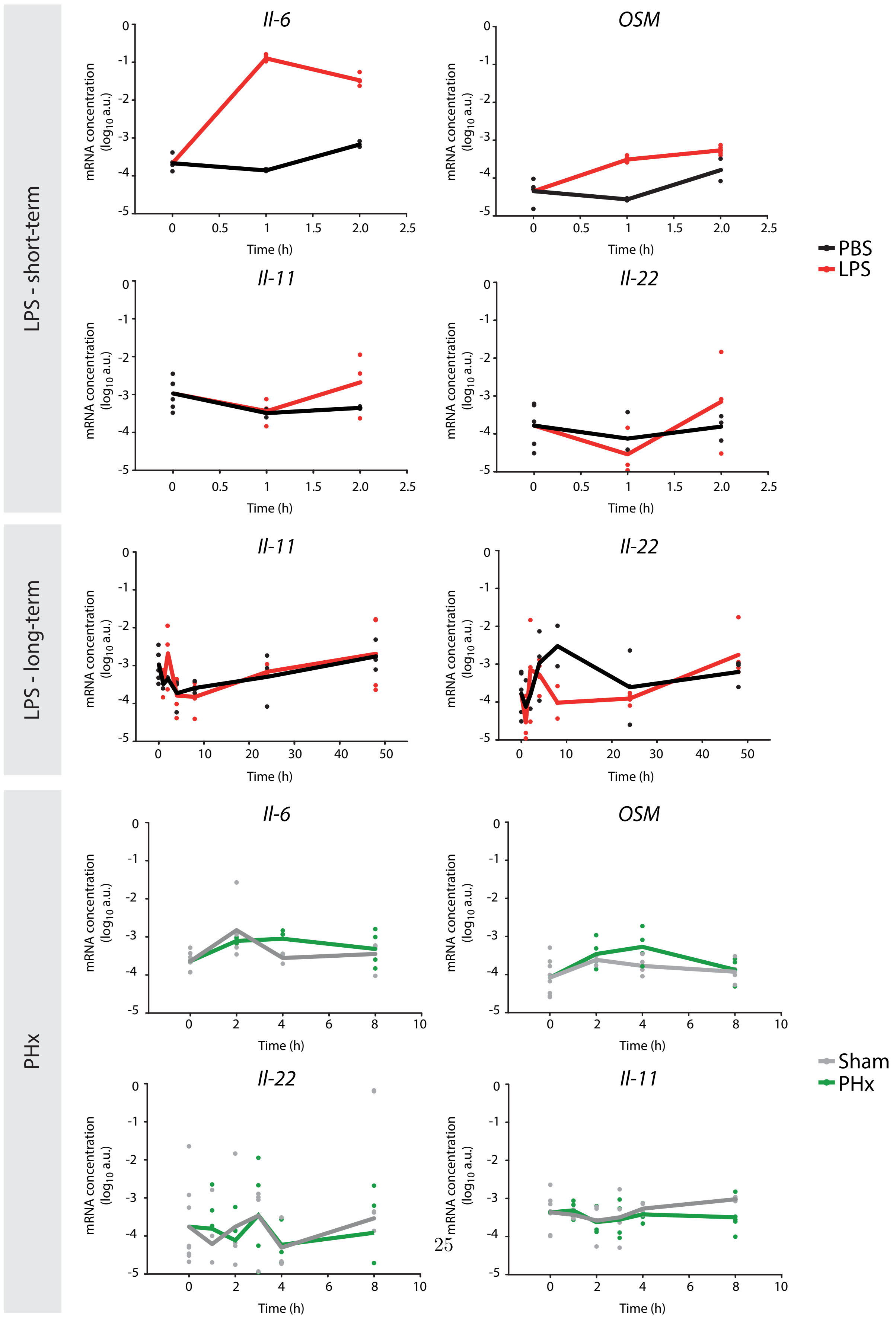

Figure S14: Mice were subjected to control (Sham) surgery or partial hepatectomy (PHx), and control (PBS) or lipopolysaccharide (LPS, 1 µg/g body weight) treatment. Liver lysates were collected at indicated time points subjected to qPCR analysis of indicated cytokines.

3 Mathematical Modeling

3.1 Model Description

The model used in this study is based on a system of Ordinary Differential Equations (ODE). These ordinary differential equations are derived by means of the law of mass-action. The time evolution of the biochemical compounds is computed by numerically integrating these differential equations. The model contains parameters which are estimated by calibrating the model to data using a Maximum-Likelihood estimation approach. Prior to stimulation, the cells are assumed to be in a resting (i.e. unstimulated) state. To achieve such an initial steady state, dynamic variables in the model (see Table S4) are pre-equilibrated prior to each simulation experiment. Model pre-equilibration was performed by simulating until the right hand side of the differential equations dropped below the equilibration threshold (1×10^{-10}). All analyses were performed using the *Data 2 Dynamics* software package [3], which is available from http://data2dynamics.org. The model considers two compartments namely cytoplasm (volume = 12.67 pl) and nucleus (volume = 0.5 pl) of primary mouse hepatocytes [1], which are taken into account by considering the relevant compartment volumes in the flux expressions. The 35 dynamic variables used in the model are summarized in Table S4, while the 4 external inputs variables are summarized in Table S5.

The model consists of 35 differential equations, which are given by the following equations:

$$\mathrm{d[JAK1\_gp130]/dt} = v_1 - v_2 - v_5 + v_7 + v_8 - v_9 \quad (1)$$

$$\mathrm{d[pJAK1\_gp130]/dt} = -v_4 + v_5 - v_6 - v_7 - v_{10} \quad (2)$$

$$\mathrm{d[pJAK1\_pgp130]/dt} = -v_3 + v_6 - v_8 - v_{11} \quad (3)$$

$$\mathrm{d[STAT3]/dt} = -v_{12} + v_{13} \cdot \frac{\mathrm{V_{nuc}}}{\mathrm{V_{cyt}}} - v_{14} \quad (4)$$

$$\mathrm{d[tpSTAT3]/dt} = v_{14} - v_{15} \quad (5)$$

$$\mathrm{d[nSTAT3]/dt} = v_{12} \cdot \frac{\mathrm{V_{cyt}}}{\mathrm{V_{nuc}}} - v_{13} + v_{15} \cdot \frac{\mathrm{V_{cyt}}}{\mathrm{V_{nuc}}} \quad (6)$$

$$\mathrm{d[nSOCS3RNA1]/dt} = v_{16} - v_{17} \quad (7)$$

$$\mathrm{d[nSOCS3RNA2]/dt} = v_{17} - v_{18} \quad (8)$$

$$\mathrm{d[nSOCS3RNA3]/dt} = v_{18} - v_{19} \quad (9)$$

$$\mathrm{d[nSOCS3RNA4]/dt} = v_{19} - v_{20} \quad (10)$$

$$\mathrm{d[nSOCS3RNA5]/dt} = v_{20} - v_{21} \quad (11)$$

$$\mathrm{d[SOCS3RNA]/dt} = v_{21} \cdot \frac{\mathrm{V_{nuc}}}{\mathrm{V_{cyt}}} - v_{22} \quad (12)$$

$$\mathrm{d[SOCS3]/dt} = -v_9 - v_{10} - v_{11} + v_{23} - v_{24} \quad (13)$$

$$\mathrm{d[nIL33RNA1]/dt} = v_{41} - v_{42} \quad (14)$$

$$\mathrm{d[nIL33RNA2]/dt} = v_{42} - v_{43} \quad (15)$$

$$\mathrm{d[nIL33RNA3]/dt} = v_{43} - v_{44} \quad (16)$$

$$\mathrm{d[nIL33RNA4]/dt} = v_{44} - v_{45} \quad (17)$$

$$\mathrm{d[nIL33RNA5]/dt} = v_{45} - v_{46} \quad (18)$$

$$\mathrm{d[nCXCL10RNA1]/dt} = v_{25} - v_{31} \quad (19)$$

$$\mathrm{d[nCXCL10RNA2]/dt} = v_{31} - v_{32} \quad (20)$$

$$\mathrm{d[nCXCL10RNA3]/dt} = v_{32} - v_{33} \quad (21)$$

$$\mathrm{d[nFGGRNA1]/dt} = v_{26} - v_{36} \quad (22)$$

$$\mathrm{d[nFGGRNA2]/dt} = v_{36} - v_{37} \quad (23)$$

$$\mathrm{d[nFGGRNA3]/dt} = v_{37} - v_{38} \quad (24)$$

$$\mathrm{d[nFGGRNA4]/dt} = v_{38} - v_{39} \quad (25)$$

$$\mathrm{d[nFGGRNA5]/dt} = v_{39} - v_{40} \quad (26)$$

$$\mathrm{d[nHPRNA1]/dt} = v_{29} - v_{35} \quad (27)$$

$$\mathrm{d[nHPXRNA1]/dt} = v_{30} - v_{34} \quad (28)$$

$$\mathrm{d[CXCL10RNA]/dt} = v_{33} \cdot \frac{\mathrm{V_{nuc}}}{\mathrm{V_{cyt}}} - v_{47} \quad (29)$$

$$\mathrm{d[FGGRNA]/dt} = v_{40} \cdot \frac{\mathrm{V_{nuc}}}{\mathrm{V_{cyt}}} - v_{48} \quad (30)$$

$$\mathrm{d[HAMPRNA]/dt} = v_{27} - v_{49} \quad (31)$$

$$\mathrm{d[IL33RNA]/dt} = v_{46} \cdot \frac{\mathrm{V_{nuc}}}{\mathrm{V_{cyt}}} - v_{50} \quad (32)$$

$$\mathrm{d[APCSRNA]/dt} = v_{28} - v_{51} \quad (33)$$

$$\mathrm{d[HPRNA]/dt} = v_{35} \cdot \frac{\mathrm{V_{nuc}}}{\mathrm{V_{cyt}}} - v_{52} \quad (34)$$

$$\mathrm{d[HPXRNA]/dt} = v_{34} \cdot \frac{\mathrm{V_{nuc}}}{\mathrm{V_{cyt}}} - v_{53} \quad (35)$$

Based on numerical integration of these equations, 10 derived quantities are computed:

$$\mathrm{[tgp130](t)} = \mathrm{[JAK1\_gp130]} + \mathrm{[pJAK1\_gp130]} + \mathrm{[pJAK1\_pgp130]} \quad (36)$$

$$[\mathrm{pJAK1}](t) = [\mathrm{pJAK1\_gp130}] + [\mathrm{pJAK1\_pgp130}] \tag{37}$$

$$[\mathrm{pgp130}](t) = [\mathrm{pJAK1\_pgp130}] \tag{38}$$

$$[\mathrm{tSOCS3}](t) = [\mathrm{SOCS3}] \tag{39}$$

$$[\mathrm{npSTAT3}](t) = \frac{\mathrm{stat3\_imp} \cdot [\mathrm{tpSTAT3}] \cdot \mathrm{V_{cyt}} \cdot (\mathrm{f\_pstat3\_imp} + 1)}{\mathrm{V_{nuc}}} \tag{40}$$

$$[\mathrm{pSTAT3}](t) = [\mathrm{tpSTAT3}] \tag{41}$$

$$[\mathrm{pSTAT3rel}](t) = \frac{[\mathrm{pSTAT3}]}{[\mathrm{STAT3}] + [\mathrm{pSTAT3}]} \tag{42}$$

$$[\mathrm{tSTAT3}](t) = \frac{[\mathrm{STAT3}] \cdot \mathrm{V_{cyt}} + [\mathrm{nSTAT3}] \cdot \mathrm{V_{nuc}} + [\mathrm{pSTAT3}] \cdot \mathrm{V_{cyt}}}{\mathrm{V_{cyt}} + \mathrm{V_{nuc}}} \tag{43}$$

$$[\mathrm{ctSTAT3}](t) = [\mathrm{STAT3}] + [\mathrm{pSTAT3}] \tag{44}$$

$$[\mathrm{ntSTAT3}](t) = [\mathrm{nSTAT3}] \tag{45}$$

$$\tag{46}$$

The flux expressions corresponding to these equations are provided in Table S6. Most of these equations were based on mass action kinetics. Additional information is provided for the following equations: Equation 5 combines a basal activation term with an active activation term that depends on the concentration of the ligand IL-6. This activation is allosterically inhibited by Ruxolitinib whose inhibitory effect is assumed to be due to blockage of the JAK1 kinase domain (binding is assumed to be in rapid equilibrium). Equation 6 represents further activation of the receptor; which is also inhibited (analogously to 5) by Ruxolitinib. Equation 14 represents a phenomenological description of the phosphorylation and dimerization of STAT3. Here SOCS3 inhibits the activated receptor by reducing its accessibility (lower apparent concentration), while STAT3 is inhibited by the drug Stattic. Equation 25 to 29 represent a phenomenological description of mRNA transcription initiation in the form of Hill kinetics, while equation 16, 30 and 41 represent a reduced form of this equation. Hill kinetics are often used to describe such processes [4]. ActD is assumed to fully inhibit all transcriptional activity (hence the (1-ActD) terms in all equations reflecting transcriptional activity).

The system of ODEs was integrated using the CVODES algorithm from the SUNDIALS suite of solvers [5]. First order derivatives were computed using the sensitivity equations and used for numerical optimization. Relative and absolute tolerances were both set to 1×10^{-8}.

Table S4: Dynamic variables used in the model. Note that all dynamic variables are simulated to steady state prior to perturbation in all of the simulation experiments.

Variable	Unit	Compartment	Initial Condition	Description
JAK1_gp130	conc. [nmol/l]	cyt	gp130_pro/gp130_deg	Unphosphorylated receptor
pJAK1_gp130	conc. [au]	cyt	0	Phosphorylated JAK1 receptor
pJAK1_pgp130	conc. [au]	cyt	0	Active receptor
STAT3	conc. [nmol/l]	cyt	0	Inactive cytoplasmic STAT3
tpSTAT3	conc. [nmol/l]	cyt	0	Active STAT3
nSTAT3	conc. [nmol/l]	nuc	total_STAT3	Inactive nuclear STAT3
nSOCS3RNA1	conc. [au]	nuc	0	SOCS3 transcriptional delay
nSOCS3RNA2	conc. [au]	nuc	0	SOCS3 transcriptional delay
nSOCS3RNA3	conc. [au]	nuc	0	SOCS3 transcriptional delay
nSOCS3RNA4	conc. [au]	nuc	0	SOCS3 transcriptional delay
nSOCS3RNA5	conc. [au]	nuc	0	SOCS3 transcriptional delay
SOCS3RNA	conc. [au]	cyt	0	SOCS3 mRNA
SOCS3	conc. [nmol/l]	cyt	0	SOCS3 protein
nIL33RNA1	conc. [au]	nuc	0	IL33 transcriptional delay
nIL33RNA2	conc. [au]	nuc	0	IL33 transcriptional delay
nIL33RNA3	conc. [au]	nuc	0	IL33 transcriptional delay
nIL33RNA4	conc. [au]	nuc	0	IL33 transcriptional delay
nIL33RNA5	conc. [au]	nuc	0	IL33 transcriptional delay
nCXCL10RNA1	conc. [au]	nuc	0	CXCL10 transcriptional delay
nCXCL10RNA2	conc. [au]	nuc	0	CXCL10 transcriptional delay
nCXCL10RNA3	conc. [au]	nuc	0	CXCL10 transcriptional delay
nFGGRNA1	conc. [au]	nuc	0	FGG transcriptional delay
nFGGRNA2	conc. [au]	nuc	0	FGG transcriptional delay
nFGGRNA3	conc. [au]	nuc	0	FGG transcriptional delay
nFGGRNA4	conc. [au]	nuc	0	FGG transcriptional delay
nFGGRNA5	conc. [au]	nuc	0	FGG transcriptional delay
nHPRNA1	conc. [au]	nuc	0	HP transcriptional delay
nHPXRNA1	conc. [au]	nuc	0	HPX transcriptional delay
CXCL10RNA	conc. [au]	cyt	0	CXCL10 mRNA
FGGRNA	conc. [au]	cyt	0	FGG mRNA
HAMPRNA	conc. [au]	cyt	0	HAMP mRNA
IL33RNA	conc. [au]	cyt	0	IL33 mRNA
APCSRNA	conc. [au]	cyt	0	APCS mRNA
HPRNA	conc. [au]	cyt	0	HP mRNA
HPXRNA	conc. [au]	cyt	0	HPX mRNA

Table S5: Inputs used in the model

Input	Unit	Default equation	Description
IL6	conc. [ng/ml]	$\mathrm{input\_il6} \cdot \mathrm{heaviside(t)}$	Interleukin 6
ActD	boolean [on/off]	$\mathrm{input\_actd} \cdot \mathrm{heaviside}(t + 10)$	Actinomycin D
Stattic	conc. [mumol/l]	$\mathrm{input\_stattic} \cdot \mathrm{heaviside}(t + 60)$	Stattic
Ruxolitinib	conc. [nmol/l]	$\mathrm{input\_ruxolitinib} \cdot \mathrm{heaviside}(t + 60) \cdot e^{-\mathrm{rux\_degrade} \cdot (t+60)}$	Ruxolitinib

Table S6: Model flux expressions

Flux	Equation	Description
v_1	$-\text{gp130\_pro} \cdot ([\text{ActD}] - 1)$	Receptor production
v_2	$[\text{JAK1\_gp130}] \cdot \text{gp130\_deg}$	Receptor degradation
v_3	$\text{gp130\_deg} \cdot [\text{pJAK1\_pgp130}]$	Receptor degradation
v_4	$\text{gp130\_deg} \cdot [\text{pJAK1\_gp130}]$	Receptor degradation
v_5	$\frac{[\text{JAK1\_gp130}]\cdot(\text{jak1\_act\_basal}+[\text{IL6}]\cdot\text{jak1\_act\_il6})}{[\text{Ruxolitinib}]\cdot\text{jak1\_inh\_ruxolitinib}+1}$	Activation of the JAK1 receptor
v_6	$\frac{\text{gp130\_act\_jak1}\cdot[\text{pJAK1\_gp130}]}{[\text{Ruxolitinib}]\cdot\text{jak1\_inh\_ruxolitinib}+1}$	Activation of the gp130 subunit
v_7	$\text{jak1\_gp130\_dea} \cdot [\text{pJAK1\_gp130}]$	Receptor dephosphorylation
v_8	$\text{jak1\_gp130\_dea} \cdot [\text{pJAK1\_pgp130}]$	Receptor dephosphorylation
v_9	$[\text{JAK1\_gp130}] \cdot [\text{SOCS3}] \cdot \text{socs3\_assoc}$	SOCS3 mediated degradation
v_{10}	$[\text{SOCS3}] \cdot [\text{pJAK1\_gp130}] \cdot \text{socs3\_assoc}$	SOCS3 mediated degradation
v_{11}	$[\text{SOCS3}] \cdot [\text{pJAK1\_pgp130}] \cdot \text{socs3\_assoc}$	SOCS3 mediated degradation
v_{12}	$[\text{STAT3}] \cdot \text{stat3\_imp}$	Import of unphosphorylated STAT3
v_{13}	$[\text{nSTAT3}] \cdot \text{stat3\_exp}$	Export of unphosphorylated STAT3
v_{14}	$\frac{[\text{STAT3}]\cdot[\text{pJAK1\_pgp130}]\cdot\text{stat3\_act\_gp130}}{([\text{SOCS3}]\cdot\text{stat3\_inh\_socs3}+1)\cdot([\text{Stattic}]\cdot\text{stat3\_inh\_stattic}+1)}$	STAT3 phosphorylation and dimerization
v_{15}	$\text{stat3\_imp} \cdot [\text{tpSTAT3}] \cdot (\text{f\_pstat3\_imp} + 1)$	STAT3 import and deactivation
v_{16}	$-\text{socs3rna\_pro} \cdot ([\text{ActD}] - 1) \cdot \frac{\left(\text{stat3\_imp}\cdot[\text{tpSTAT3}]\cdot V_{cyt}\cdot(\text{f\_pstat3\_imp}+1)\right)^{\text{socs3rna\_hill}}}{V_{nuc}}$	Initiation of SOCS3 mRNA transcription
v_{17}	$[\text{nSOCS3RNA1}] \cdot \text{socs3rna\_delay}$	Transcriptional delay
v_{18}	$[\text{nSOCS3RNA2}] \cdot \text{socs3rna\_delay}$	Transcriptional delay
v_{19}	$[\text{nSOCS3RNA3}] \cdot \text{socs3rna\_delay}$	Transcriptional delay
v_{20}	$[\text{nSOCS3RNA4}] \cdot \text{socs3rna\_delay}$	Transcriptional delay
v_{21}	$[\text{nSOCS3RNA5}] \cdot \text{socs3rna\_delay}$	Transcriptional delay
v_{22}	$[\text{SOCS3RNA}] \cdot \text{socs3rna\_des}$	SOCS3 mRNA degradation
v_{23}	$[\text{SOCS3RNA}] \cdot \text{socs3\_pro}$	SOCS3 mRNA translation
v_{24}	$[\text{SOCS3}] \cdot \text{socs3\_des}$	SOCS3 degradation
v_{25}	$-\frac{\text{cxcl10rna\_pro}\cdot([\text{ActD}]-1)}{{\frac{(\text{cxcl10rna\_ka}\cdot V_{nuc})}{(\text{stat3\_imp}\cdot[\text{tpSTAT3}]\cdot V_{cyt}\cdot(\text{f\_pstat3\_imp}+1))}}^{\text{cxcl10rna\_hill}}+1}$	Initiation of CXCL10 mRNA transcription
v_{26}	$-\frac{\text{fggrna\_pro}\cdot([\text{ActD}]-1)}{{\frac{(\text{fggrna\_ka}\cdot V_{nuc})}{(\text{stat3\_imp}\cdot[\text{tpSTAT3}]\cdot V_{cyt}\cdot(\text{f\_pstat3\_imp}+1))}}^{\text{fggrna\_hill}}+1}$	Initiation of FGG mRNA transcription
v_{27}	$-\frac{\text{hamprna\_pro}\cdot([\text{ActD}]-1)}{{\frac{(\text{hamprna\_ka}\cdot V_{nuc})}{(\text{stat3\_imp}\cdot[\text{tpSTAT3}]\cdot V_{cyt}\cdot(\text{f\_pstat3\_imp}+1))}}^{\text{hamprna\_hill}}+1}$	Initiation of HAMP mRNA transcription
v_{28}	$-\frac{\text{apcsrna\_pro}\cdot([\text{ActD}]-1)}{{\frac{(\text{apcsrna\_ka}\cdot V_{nuc})}{(\text{stat3\_imp}\cdot[\text{tpSTAT3}]\cdot V_{cyt}\cdot(\text{f\_pstat3\_imp}+1))}}^{\text{apcsrna\_hill}}+1}$	Initiation of APCS mRNA transcription
v_{29}	$-\frac{\text{hprna\_pro}\cdot([\text{ActD}]-1)}{{\frac{(\text{hprna\_ka}\cdot V_{nuc})}{(\text{stat3\_imp}\cdot[\text{tpSTAT3}]\cdot V_{cyt}\cdot(\text{f\_pstat3\_imp}+1))}}^{\text{hprna\_hill}}+1}$	Initiation of HP mRNA transcription
v_{30}	$-\text{hpxrna\_pro} \cdot ([\text{ActD}] - 1) \cdot \frac{\left(\text{stat3\_imp}\cdot[\text{tpSTAT3}]\cdot V_{cyt}\cdot(\text{f\_pstat3\_imp}+1)\right)^{\text{hpxrna\_hill}}}{V_{nuc}}$	Initiation of HPX mRNA transcription
v_{31}	$\text{cxcl10rna\_delay} \cdot [\text{nCXCL10RNA1}]$	Transcriptional delay
v_{32}	$\text{cxcl10rna\_delay} \cdot [\text{nCXCL10RNA2}]$	Transcriptional delay
v_{33}	$\text{cxcl10rna\_delay} \cdot [\text{nCXCL10RNA3}]$	Transcriptional delay
v_{34}	$\text{hpxrna\_delay} \cdot [\text{nHPXRNA1}]$	Transcriptional delay
v_{35}	$\text{hprna\_delay} \cdot [\text{nHPRNA1}]$	Transcriptional delay
v_{36}	$\text{fggrna\_delay} \cdot [\text{nFGGRNA1}]$	Transcriptional delay
v_{37}	$\text{fggrna\_delay} \cdot [\text{nFGGRNA2}]$	Transcriptional delay
v_{38}	$\text{fggrna\_delay} \cdot [\text{nFGGRNA3}]$	Transcriptional delay
v_{39}	$\text{fggrna\_delay} \cdot [\text{nFGGRNA4}]$	Transcriptional delay
v_{40}	$\text{fggrna\_delay} \cdot [\text{nFGGRNA5}]$	Transcriptional delay
v_{41}	$-\text{il33rna\_pro} \cdot ([\text{ActD}] - 1) \cdot \frac{\left(\text{stat3\_imp}\cdot[\text{tpSTAT3}]\cdot V_{cyt}\cdot(\text{f\_pstat3\_imp}+1)\right)^{\text{il33rna\_hill}}}{V_{nuc}}$	Initiation of IL33 mRNA transcription
v_{42}	$\text{il33rna\_delay} \cdot [\text{nIL33RNA1}]$	Transcriptional delay
v_{43}	$\text{il33rna\_delay} \cdot [\text{nIL33RNA2}]$	Transcriptional delay
v_{44}	$\text{il33rna\_delay} \cdot [\text{nIL33RNA3}]$	Transcriptional delay
v_{45}	$\text{il33rna\_delay} \cdot [\text{nIL33RNA4}]$	Transcriptional delay
v_{46}	$\text{il33rna\_delay} \cdot [\text{nIL33RNA5}]$	Transcriptional delay
v_{47}	$[\text{CXCL10RNA}] \cdot \text{cxcl10rna\_des}$	CXCL10 degradation
v_{48}	$[\text{FGGRNA}] \cdot \text{fggrna\_des}$	FGG degradation
v_{49}	$[\text{HAMPRNA}] \cdot \text{hamprna\_des}$	HAMP degradation
v_{50}	$[\text{IL33RNA}] \cdot \text{il33rna\_des}$	IL33 degradation
v_{51}	$[\text{APCSRNA}] \cdot \text{apcsrna\_des}$	APCS degradation
v_{52}	$[\text{HPRNA}] \cdot \text{hprna\_des}$	HP degradation
v_{53}	$[\text{HPXRNA}] \cdot \text{hpxrna\_des}$	Transcriptional delay

3.2 Observables

The model contains 18 standard observables listed in table S7. Certain experiments may contain experiment specific observation functions, which are detailed in the experiment section (see 4).

Table S7: Model observables and error models

Observable		Equations
pJAK1 Western blot [au]	y	$\log_{10}(\text{offset\_pjak1\_wb\_filename} + [\text{pJAK1}] \cdot \text{scale\_pjak1\_wb\_filename})$
	σ	sd_pjak1_wb_filename
pgp130 Western blot [au]	y	$\log_{10}(\text{offset\_pgp130\_wb\_filename} + [\text{pgp130}] \cdot \text{scale\_pgp130\_wb\_filename})$
	σ	sd_pgp130_wb_filename
pSTAT3 Western blot [au]	y	$\log_{10}(\text{offset\_pstat3\_wb\_filename} + [\text{pSTAT3}] \cdot \text{scale\_pstat3\_wb\_filename})$
	σ	sd_pstat3_wb_filename
pSTAT3 [au]	y	$\log_{10}(\text{offset\_pstat3\_lumi\_filename} + [\text{pSTAT3}] \cdot \text{scale\_pstat3\_lumi\_filename})$
	σ	sd_pstat3_lumi_filename
pSTAT3 Western blot [au]	y	$\log_{10}(\text{offset\_npstat3\_wb\_filename} + [\text{npSTAT3}] \cdot \text{scale\_npstat3\_wb\_filename})$
	σ	sd_npstat3_wb_filename
SOCS3 Western blot [au]	y	$\log_{10}(\text{offset\_socs3\_wb\_filename} + \text{scale\_socs3\_wb\_filename} \cdot [\text{tSOCS3}])$
	σ	sd_socs3_wb_filename
SOCS3 qPCR [au]	y	$\log_{10}(\text{offset\_socs3\_qpcr\_filename} + [\text{SOCS3RNA}] \cdot \text{scale\_socs3\_qpcr\_filename})$
	σ	sd_socs3_qpcr_filename
Total nuclear STAT3 [au]	y	$\log_{10}(\frac{[\text{ntSTAT3}]}{[\text{STAT3}]+[\text{pSTAT3}]})$
	σ	sd_ntstat3_ratio_filename
JAK1_gp130 complex molecules per cell [nmol/l]	y	$\log_{10}([\text{tgp130}])$
	σ	sd_JAK1_gp130_abs
Cytoplasmic STAT3 molecules per cell [nmol/l]	y	$\log_{10}([\text{ctSTAT3}])$
	σ	sd_ctSTAT3_abs
SOCS3 molecules per cell [nmol/l]	y	$\log_{10}([\text{tSOCS3}])$
	σ	sd_SOCS3_abs
CXCL10 qPCR [au]	y	$\log_{10}(\text{offset\_cxcl10\_qpcr\_filename} + [\text{CXCL10RNA}] \cdot \text{scale\_cxcl10\_qpcr\_filename})$
	σ	sd_cxcl10_qpcr
FGG qPCR [au]	y	$\log_{10}(\text{offset\_fgg\_qpcr\_filename} + [\text{FGGRNA}] \cdot \text{scale\_fgg\_qpcr\_filename})$
	σ	sd_fgg_qpcr
HAMP qPCR [au]	y	$\log_{10}(\text{offset\_hamp\_qpcr\_filename} + [\text{HAMPRNA}] \cdot \text{scale\_hamp\_qpcr\_filename})$
	σ	sd_hamp_qpcr
IL33 qPCR [au]	y	$\log_{10}(\text{offset\_il33\_qpcr\_filename} + [\text{IL33RNA}] \cdot \text{scale\_il33\_qpcr\_filename})$
	σ	sd_il33_qpcr
APCS qPCR [au]	y	$\log_{10}(\text{offset\_apcs\_qpcr\_filename} + [\text{APCSRNA}] \cdot \text{scale\_apcs\_qpcr\_filename})$
	σ	sd_apcs_qpcr
HP qPCR [au]	y	$\log_{10}(\text{offset\_hp\_qpcr\_filename} + [\text{HPRNA}] \cdot \text{scale\_hp\_qpcr\_filename})$
	σ	sd_hp_qpcr
HPX qPCR [au]	y	$\log_{10}(\text{offset\_hpx\_qpcr\_filename} + [\text{HPXRNA}] \cdot \text{scale\_hpx\_qpcr\_filename})$
	σ	sd_hpx_qpcr

3.3 Default Parameter Transformations

Some of the bare model parameters are transformed before each simulation experiment. This is done both to facilitate fitting (e.g. decoupling scale from dynamics), but also to allow condition specific parameter changes. The ODE system is modified by the following parameter transformations:

$$\text{apcsrna\_pro} \to \frac{\text{apcsrna\_pro}}{\text{scale\_apcs\_qpcr\_37}} \tag{47}$$

$$\text{cxcl10rna\_pro} \to \frac{\text{cxcl10rna\_pro}}{\text{scale\_cxcl10\_qpcr\_31}} \tag{48}$$

$$\text{fggrna\_pro} \to \frac{\text{fggrna\_pro}}{\text{scale\_fgg\_qpcr\_34}} \tag{49}$$

$$\text{hamprna\_pro} \to \frac{\text{hamprna\_pro}}{\text{scale\_hamp\_qpcr\_34}} \tag{50}$$

$$\text{hprna\_pro} \to \frac{\text{hprna\_pro}}{\text{scale\_hp\_qpcr\_37}} \tag{51}$$

$$\text{hpxrna\_pro} \to \frac{\text{hpxrna\_pro}}{\text{scale\_hpx\_qpcr\_37}} \tag{52}$$

$$\text{il33rna\_pro} \rightarrow \frac{\text{il33rna\_pro}}{\text{scale\_il33\_qpcr\_34}} \tag{53}$$

$$\text{init\_JAK1\_gp130} \rightarrow \frac{\text{gp130\_pro}}{\text{gp130\_deg}} \tag{54}$$

$$\text{init\_nSTAT3} \rightarrow \text{total\_STAT3} \tag{55}$$

$$\text{input\_actd} \rightarrow 0 \tag{56}$$

$$\text{input\_il6} \rightarrow 40 \tag{57}$$

$$\text{input\_ruxolitinib} \rightarrow 0 \tag{58}$$

$$\text{input\_stattic} \rightarrow 0 \tag{59}$$

$$\text{jak1\_act\_basal} \rightarrow \frac{\text{jak1\_act\_basal}}{\text{scale\_pgp130\_wb\_6}} \tag{60}$$

$$\text{jak1\_act\_il6} \rightarrow \frac{\text{jak1\_act\_il6}}{\text{scale\_pgp130\_wb\_6}} \tag{61}$$

$$\text{scale\_npstat3\_wb\_filename} \rightarrow \frac{\text{scale\_npstat3\_wb\_filename}}{\text{total\_STAT3}} \tag{62}$$

$$\text{scale\_pstat3\_lumi\_filename} \rightarrow \frac{\text{scale\_pstat3\_lumi\_filename}}{\text{total\_STAT3}} \tag{63}$$

$$\text{scale\_pstat3\_wb\_filename} \rightarrow \frac{\text{scale\_pstat3\_wb\_filename}}{\text{total\_STAT3}} \tag{64}$$

$$\text{socs3\_pro} \rightarrow \text{scale\_socs3\_qpcr\_52} \cdot \text{socs3\_pro} \tag{65}$$

$$\text{socs3rna\_pro} \rightarrow \frac{\text{socs3rna\_pro}}{\text{scale\_socs3\_qpcr\_52}} \tag{66}$$

$$\text{stat3\_act\_gp130} \rightarrow \text{scale\_pgp130\_wb\_6} \cdot \text{stat3\_act\_gp130} \tag{67}$$

$$\text{V}_{\text{cyt}} \rightarrow 12.67 \tag{68}$$

$$\text{V}_{\text{nuc}} \rightarrow 0.5 \tag{69}$$

$$\tag{70}$$

3.4 Dynamic Parameters

In total 656 parameters are estimated from the experimental data based on a total of 4508 data points. The model parameters were estimated by maximum likelihood estimation applying the MATLAB lsqnonlin algorithm. The model parameters which influence system dynamics are listed in Table S8. Parameters highlighted in red color indicate parameter values close to their bounds. An extensive list of all the estimated parameters (including all observational and error model parameters) is given in section 5. These can be found in Table S102 – S116.

	name	θ_{min}	$\hat{\theta}$	θ_{max}	log	non-log $\hat{\theta}$	fitted
141	apcsrna_des	-5	-2.9749	+5	1	$+1.06 \cdot 10^{-03}$	1
142	apcsrna_hill	-5	+0.6266	+5	1	$+4.23 \cdot 10^{+00}$	1
143	apcsrna_ka	-5	+1.6998	+5	1	$+5.01 \cdot 10^{+01}$	1
144	apcsrna_pro	-5	-2.8555	+5	1	$+1.39 \cdot 10^{-03}$	1
145	cxcl10rna_delay	-5	-1.3446	+5	1	$+4.52 \cdot 10^{-02}$	1
146	cxcl10rna_des	-5	-1.3448	+5	1	$+4.52 \cdot 10^{-02}$	1
147	cxcl10rna_hill	-5	+0.9890	+5	1	$+9.75 \cdot 10^{+00}$	1
148	cxcl10rna_ka	-5	+2.1546	+5	1	$+1.43 \cdot 10^{+02}$	1
149	cxcl10rna_pro	-5	+0.7548	+5	1	$+5.69 \cdot 10^{+00}$	1
150	f_pstat3_imp	+0	+4.9712	+100	0	$+4.97 \cdot 10^{+00}$	1
151	fggrna_delay	-5	-0.9180	+5	1	$+1.21 \cdot 10^{-01}$	1
152	fggrna_des	-5	-2.1131	+5	1	$+7.71 \cdot 10^{-03}$	1
153	fggrna_hill	-5	+0.5952	+5	1	$+3.94 \cdot 10^{+00}$	1
154	fggrna_ka	-5	+1.6916	+5	1	$+4.92 \cdot 10^{+01}$	1
155	fggrna_pro	-5	-0.6278	+5	1	$+2.36 \cdot 10^{-01}$	1
156	gp130_act_jak1	-15	-0.2700	+6	1	$+5.37 \cdot 10^{-01}$	1
157	gp130_deg	-5	-2.4193	+3	1	$+3.81 \cdot 10^{-03}$	1
158	gp130_pro	-5	-2.6189	+3	1	$+2.40 \cdot 10^{-03}$	1
159	hamprna_des	-8	-3.6729	+8	1	$+2.12 \cdot 10^{-04}$	1
160	hamprna_hill	-8	+0.5128	+8	1	$+3.26 \cdot 10^{+00}$	1
161	hamprna_ka	-8	+1.6750	+8	1	$+4.73 \cdot 10^{+01}$	1
162	hamprna_pro	-8	-2.5187	+8	1	$+3.03 \cdot 10^{-03}$	1
163	hprna_delay	-5	-2.9264	+5	1	$+1.18 \cdot 10^{-03}$	1
164	hprna_des	-5	-2.9214	+5	1	$+1.20 \cdot 10^{-03}$	1
165	hprna_hill	-5	+0.4497	+5	1	$+2.82 \cdot 10^{+00}$	1
166	hprna_ka	-5	+1.7030	+5	1	$+5.05 \cdot 10^{+01}$	1
167	hprna_pro	-5	-1.2230	+5	1	$+5.98 \cdot 10^{-02}$	1
168	hpxrna_delay	-5	-3.4287	+5	1	$+3.73 \cdot 10^{-04}$	1
169	hpxrna_des	-5	-3.4283	+5	1	$+3.73 \cdot 10^{-04}$	1
170	hpxrna_hill	-5	+0.1176	+5	1	$+1.31 \cdot 10^{+00}$	1
171	hpxrna_pro	-5	-3.6257	+5	1	$+2.37 \cdot 10^{-04}$	1
172	il33rna_delay	-8	-0.8718	+8	1	$+1.34 \cdot 10^{-01}$	1
173	il33rna_des	-8	-2.9602	+8	1	$+1.10 \cdot 10^{-03}$	1
174	il33rna_hill	-8	+0.2458	+8	1	$+1.76 \cdot 10^{+00}$	1
175	il33rna_pro	-8	-4.4645	+8	1	$+3.43 \cdot 10^{-05}$	1
176	jak1_act_basal	-15	-2.3652	+6	1	$+4.31 \cdot 10^{-03}$	1
177	jak1_act_il6	-15	-2.0421	+6	1	$+9.08 \cdot 10^{-03}$	1
178	jak1_gp130_dea	-15	-1.1070	+6	1	$+7.82 \cdot 10^{-02}$	1
179	jak1_inh_ruxolitinib	-15	-2.2202	+6	1	$+6.02 \cdot 10^{-03}$	1
357	rux_degrade	-15	-3.0129	+6	1	$+9.71 \cdot 10^{-04}$	1
358	scale_apcs_qpcr_37	-15	+2.6611	+6	1	$+4.58 \cdot 10^{+02}$	1
369	scale_cxcl10_qpcr_31	-15	+2.4397	+6	1	$+2.75 \cdot 10^{+02}$	1
382	scale_fgg_qpcr_34	-15	+1.3594	+6	1	$+2.29 \cdot 10^{+01}$	1
398	scale_hamp_qpcr_34	-15	+0.5955	+6	1	$+3.94 \cdot 10^{+00}$	1
414	scale_hp_qpcr_37	-15	+1.2459	+6	1	$+1.76 \cdot 10^{+01}$	1
425	scale_hpx_qpcr_37	-15	+0.0982	+6	1	$+1.25 \cdot 10^{+00}$	1
436	scale_il33_qpcr_34	-15	+0.1663	+6	1	$+1.47 \cdot 10^{+00}$	1
471	scale_pgp130_wb_6	-5	+0.8987	+5	1	$+7.92 \cdot 10^{+00}$	1
533	scale_socs3_qpcr_52	-15	+1.7928	+6	1	$+6.21 \cdot 10^{+01}$	1
658	socs3_assoc	-5	-1.7135	+3	1	$+1.93 \cdot 10^{-02}$	1
659	socs3_des	-15	-0.3481	+6	1	$+4.49 \cdot 10^{-01}$	1
660	socs3_pro	-15	+0.2299	+6	1	$+1.70 \cdot 10^{+00}$	1
661	socs3rna_delay	-15	-0.5172	+6	1	$+3.04 \cdot 10^{-01}$	1
662	socs3rna_des	-15	-0.5173	+6	1	$+3.04 \cdot 10^{-01}$	1
663	socs3rna_hill	-10	+0.2338	+4	1	$+1.71 \cdot 10^{+00}$	1
664	socs3rna_pro	-15	-2.7695	+10	1	$+1.70 \cdot 10^{-03}$	1
665	stat3_act_gp130	-15	-0.3856	+6	1	$+4.12 \cdot 10^{-01}$	1
666	stat3_exp	-15	+0.2946	+6	1	$+1.97 \cdot 10^{+00}$	1
667	stat3_imp	-15	-1.2265	+6	1	$+5.94 \cdot 10^{-02}$	1
668	stat3_inh_socs3	-5	-0.2084	+3	1	$+6.19 \cdot 10^{-01}$	1
669	stat3_inh_stattic	-15	-0.9984	+6	1	$+1.00 \cdot 10^{-01}$	1
670	total_STAT3	-15	+2.8921	+6	1	$+7.80 \cdot 10^{+02}$	1

Table S8: Estimated dynamic parameter values
$\hat{\theta}$ indicates the estimated value of the parameters. θ_{min} and θ_{max} indicate the upper and lower bounds for the parameters. The log-column indicates if the value of a parameter was log-transformed. If log = 1 the non-log-column indicates the non-logarithmic value of the estimate. The fitted-column indicates if the parameter value was estimated (1), was temporarily fixed (0) or if its value was fixed to a constant value (2).

3.5 Model Development

Models correspond to simplifications of reality which aid in understanding the phenomena they were developed to describe. It is important to balance model complexity with experimental observations, such that only those aspects of reality on which the observed data has any meaningful information are included in the model. By restricting the model to a minimal number of effects, one can ameliorate predictive uncertainties. The more precise a prediction, the easier it will be to falsify, but also the more we learn when the prediction is not falsified. Models that predict accurately, while still resisting falsification are the most useful. Model development was performed by iteratively performing parameter and uncertainty estimation followed by either model reduction or data acquisition. An important concept here is parameter identifiability. When constructing a model from first principles, it is typically the case that several parameters are non-identifiable, which means they do not have finite confidence intervals. In turn, this leads to large uncertainties in the predictions. It is therefore important to assess the identifiability of the various model parameters and resolve them when they have direct consequences on predictions of interest.

The model was tailored to the data in such a way that the dynamic parameters were identifiable. To assess identifiability, we computed likelihood profiles, which systematically trace an optimal path over the likelihood in order to determine parameter confidence bounds. The algorithm works as follows: Every profile is initiated at the best fit parameters. One parameter is subsequently selected and incrementally sampled over a range while optimizing for all the other parameters. If the parameter is identifiable, the negative log-likelihood will reach a statistical threshold in both directions. If the negative log-likelihood does not reach this bound on either side, then it is not possible to determine a range for the parameter and the parameter is classified as non-identifiable.

In this process of iterative model refinement a few data-based model reduction steps are worth mentioning:

1. Pre-stimulation steady states are determined implicitly, by simulating the system to convergence. As such, the system has no initial condition parameters for moieties which are not conserved and one parameter per conserved moiety to estimate its total pool.

2. Only relative mRNA levels were quantified within this study. Therefore the absolute scale of SOCS3 mRNA is unknown. By non-dimensionalizing those parts of the system, the dynamic parameters were decoupled from the scale of these components. A non-identifiability in the scaling parameters can cause problems when computing profile likelihoods (since extremely low or high values result in numerical difficulties). For this reason, one of the mRNA scaling factors was set to unity, providing a fixed scale for the mRNA levels.

3. The original model contained Hill kinetics for SOCS3 activation. However, profile likelihood analysis revealed that even after fixing the scale of SOCS3 mRNA, the mRNA production rate and microscopic dissociation constant (socs3_ka) were non-identifiable. This was the result of a high microscopic dissociation constant (open ended profile towards high rates). For this reason, this part of the rate equation was reformulated as shown in Equation 71, without loss of fidelity to the data.

$$\frac{\text{socs3rna\_pro}^{old}}{1+\left(\frac{\text{socs3rna\_ka}}{[\text{npSTAT3}]}\right)^{\text{socs3\_hill}}} \rightarrow \frac{\text{socs3rna\_pro}^{\text{old}}}{\text{socs3}_{\text{r}}\text{na\_ka}^{\text{socs3\_hill}}}[\text{npSTAT3}]^{\text{socs3\_hill}} \rightarrow \text{socs3rna\_pro}[\text{npSTAT3}]^{\text{socs3\_hill}} \tag{71}$$

4. Considering that import and export of both phosphorylated and unphosphorylated STAT3 have been suggested to occur in literature [6, 7], the initial model contained import and export parameters for both

phosphorylated and unphosphorylated STAT3. Profile likelihood analysis indicated that nuclear export could be omitted from the model as its contribution was non-observably small. This parameter was removed from the system without incurring any penalty on the data fit.

5. Based on the data, the nuclear dephosphorylation rate was very high (open-ended profile likelihood towards high values). This indicated that no dynamical difference between nuclear pSTAT3 and cytoplasmic pSTAT3 could be observed, despite measurements on both. Considering that nuclear STAT3 dephosphorylation is so rapid that the level of nuclear phosphorylated STAT3 follows the cytoplasmic concentration exactly, the dynamic part of the model was reduced by one equation. The speed at which dephosphorylation occured in the nucleus also meant that phosphorylated STAT3 in the nucleus contributed a negligible amount to the total of STAT3. Therefore, despite measurements of the absolute number of STAT3 molecules per cell, the absolute scale of nuclear pSTAT3 is unknown. More concretely; the nuclear phosphorylated STAT3 was modelled as the cytoplasmic phosphorylated STAT3 level multiplied by an estimated scaling factor. Aside from resolving non-identifiabilities, step 4 and 5 also reduced the stiffness of the system of ordinary differential equations and resulted in faster simulation times.

6. Based on subsequent profile likelihood analysis, we determined that the import and export rates of unphosphorylated STAT3 were also not fully identifiable. The resulting parameterization contained at least two qualitatively distinct optima. In the first optimum, the import and export of unphosphorylated STAT3 was rapid and the import rate of STAT3 was faster than that of pSTAT3. In the second optimum, the import and export rates of unphosphorylated STAT3 were relatively low (but observably nonzero). We decided to reparameterize the model in such a way that the import rate of the phosphorylated form is always bigger than the unphosphorylated form pstat3_imp = stat3_imp(1 + f_pstat3_imp). This parameter transformation resolved the non-identifiability.

7. Upstream in the model, only a very small fraction of the receptors were phosphorylated. Similarly to nuclear pSTAT3, this meant that the scale of the activated receptors was unknown despite measurements on the total (there was only a meaningful upper bound). As such, the basal and induced activation parameters were practically non-identifiable (no lower bound), and correlated. Again, we verified this by applying a parameter transformation based on non-dimensionalization (dividing the dynamic parameter by the scale of one of the data sets used for parameter estimation). This rendered the dynamical parameter identifiable and the scale non-identifiable.

8. Initially two inhibition mechanisms for Ruxolitinib were included in order to avoid biasing our decision as to where the activity of Ruxolitinib was most pronounced. One was at the receptor level, while the other inhibited the activation of STAT3 directly. While the former parameter was clearly identifiable from the data, the latter mechanism of inhibition was non-identifiable (no lower bound) and could safely be removed without appreciable loss of fit accuracy.

9. The original model contained a SOCS3 inhibition effect on the phosphorylation steps in the model. This caused a fairly strong transient in the concentration of SOCS3 post-peak due to lack of damping. By allowing receptor production and degradation, and explicit SOCS3 binding to the receptor (with its own degradation rate), we obtained a model with a comparable fit but a less extreme post-peak oscillatory transient. This was in better agreement with the data.

10. Profiling the new receptor model showed that the modification at the receptor level introduced a new non-identifiability in the degradation rate of the SOCS3 bound receptor (open ended profiles to positive infinity). Considering that the speed of degradation was not constrained by the available data, this allowed for a model reduction involving the removal of all SOCS3-bound receptor states. In the new model, SOCS3 binding constitutes an additional degradation mechanism for the receptor to be degraded.

11. The initial APP model looked very similar to the model for SOCS3. Delays were implemented using the linear chain trick in which a delay is distributed over multiple steps with identical rates. Increasing

the number of steps in such a linear chain results in a sharper delay, while fewer steps result in smearing of the input signal. An APP model with too many delay steps was characterized by an open ended profile towards positive infinity for the degradation rate of the mRNA (meaning that the mRNA follows the last delayed intermediate exactly). The models for the APP genes were iteratively reduced by reducing the number of delay states until each APP model was identifiable. In the finalized model SOCS3, CXCL10, FGG, IL33, HP and HPX required a delay. Whereas SOCS3, FGG and IL33 required five intermediate delay steps to adequately describe the data, CXCL10 required only three and HP and HPX only a single delay intermediate. HAMP and APCS were sufficiently described without any delay. Similarly to SOCS3, the ka's of HPX and IL-33 were non-identifiable and could be removed from the model.

Concluding, the result from these model simplifications is a model where all the dynamical parameters are identifiable. The profile likelihoods corresponding to the dynamical parameters of the core model are shown in Figure S15. The profile likelihoods for the APP genes can be found in S16 to S22. To keep computational time reasonable, and since the APP genes do not feed back into the core model, the APP likelihood profiles were computed keeping the core model (SOCS3/STAT3 axis) parameters fixed. The final estimated parameter values were then incorporated into the full model.

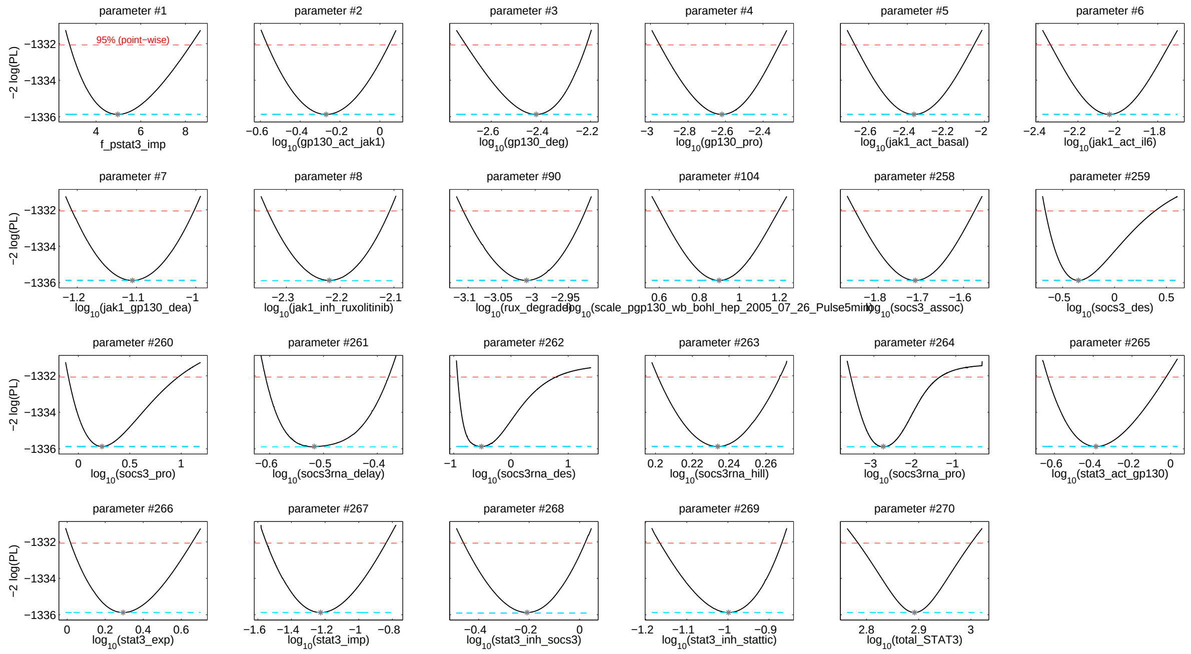

Figure S15: Overview of the profile likelihood of the core model parameters. The solid lines indicate the profile likelihood. The dashed lines indicate the threshold to assess confidence intervals. The asterisks indicate the optimal parameter values.

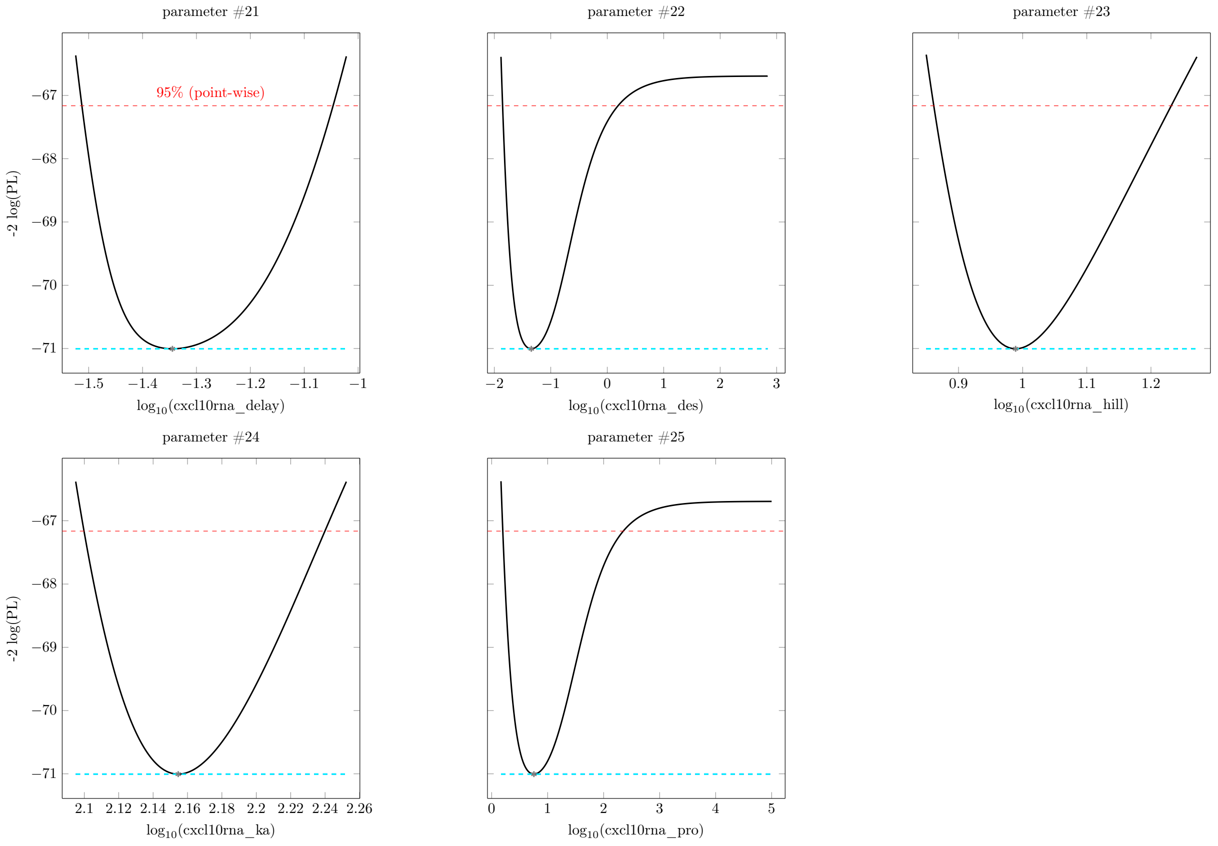

Figure S16: Overview of the profile likelihood of the CXCL10 model parameters. The solid lines indicate the profile likelihood. The dashed lines indicate the threshold to assess confidence intervals. The asterisks indicate the optimal parameter values.

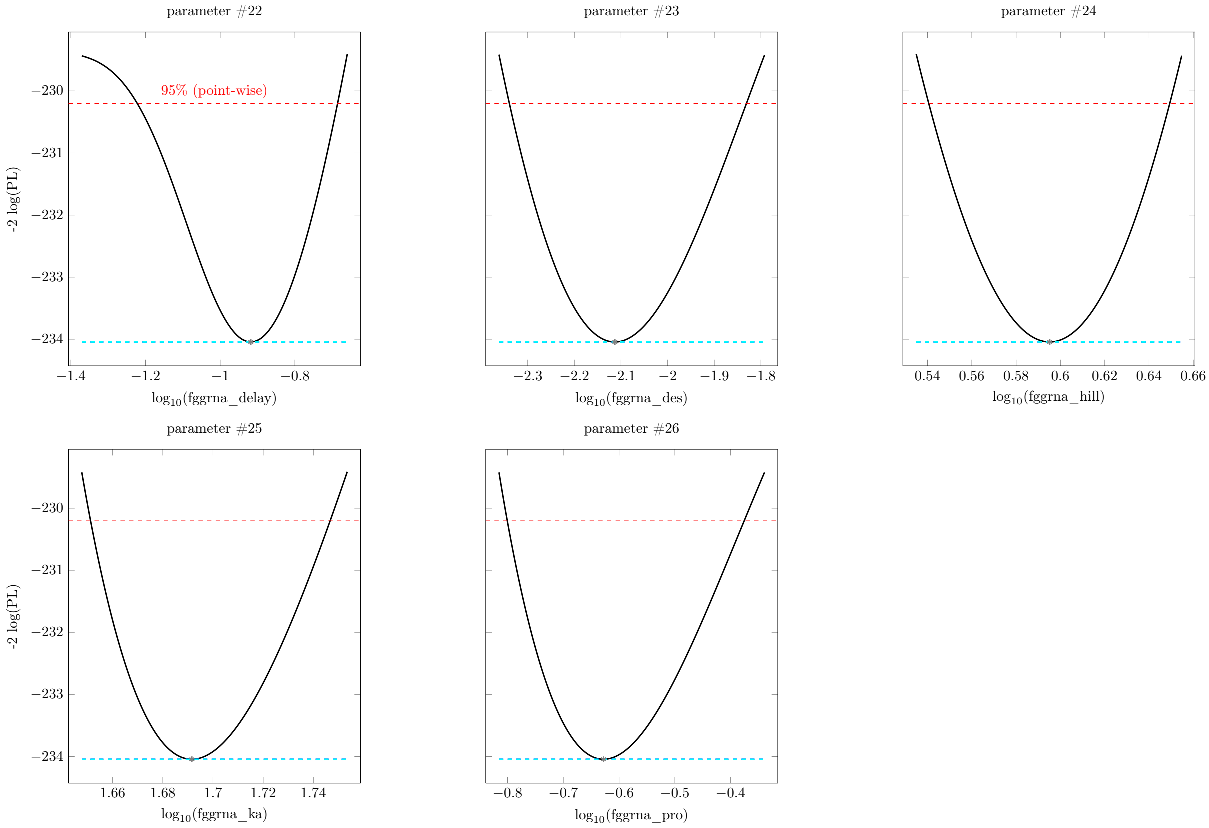

Figure S17: Overview of the profile likelihood of the FGG model parameters. The solid lines indicate the profile likelihood. The dashed lines indicate the threshold to assess confidence intervals. The asterisks indicate the optimal parameter values.

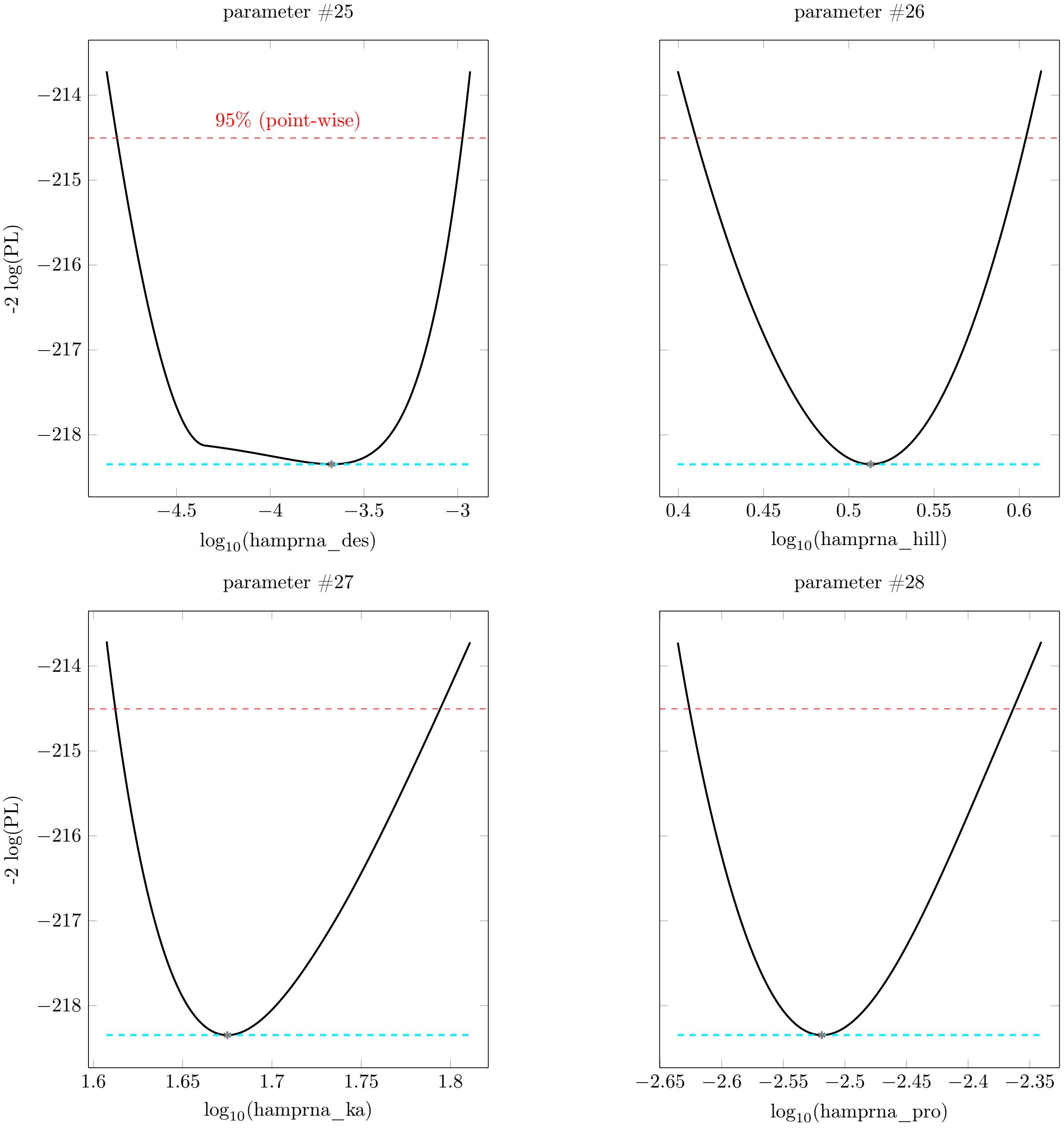

Figure S18: Overview of the profile likelihood of the HAMP model parameters. The solid lines indicate the profile likelihood. The dashed lines indicate the threshold to assess confidence intervals. The asterisks indicate the optimal parameter values.

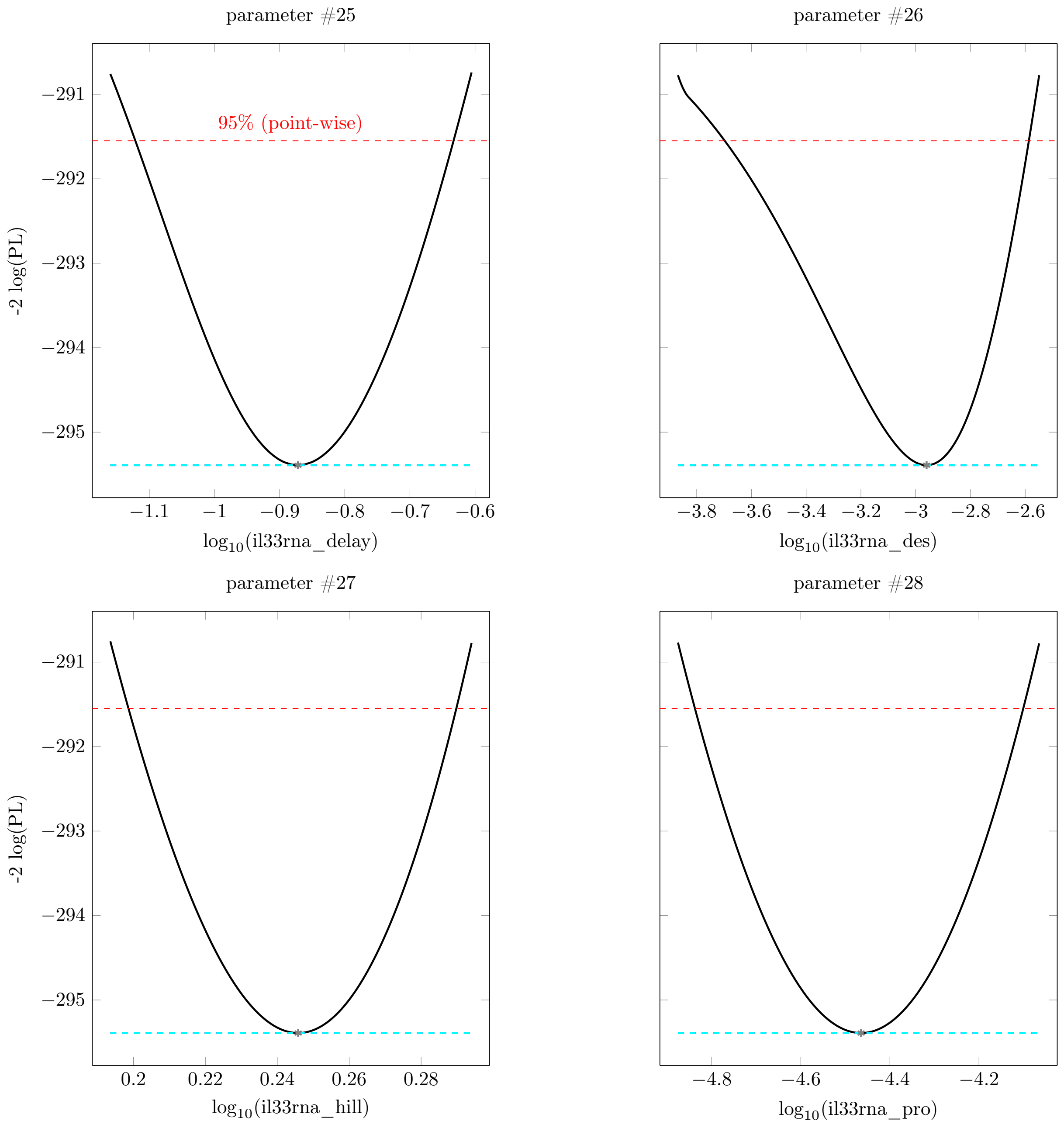

Figure S19: Overview of the profile likelihood of the IL33 model parameters. The solid lines indicate the profile likelihood. The dashed lines indicate the threshold to assess confidence intervals. The asterisks indicate the optimal parameter values.

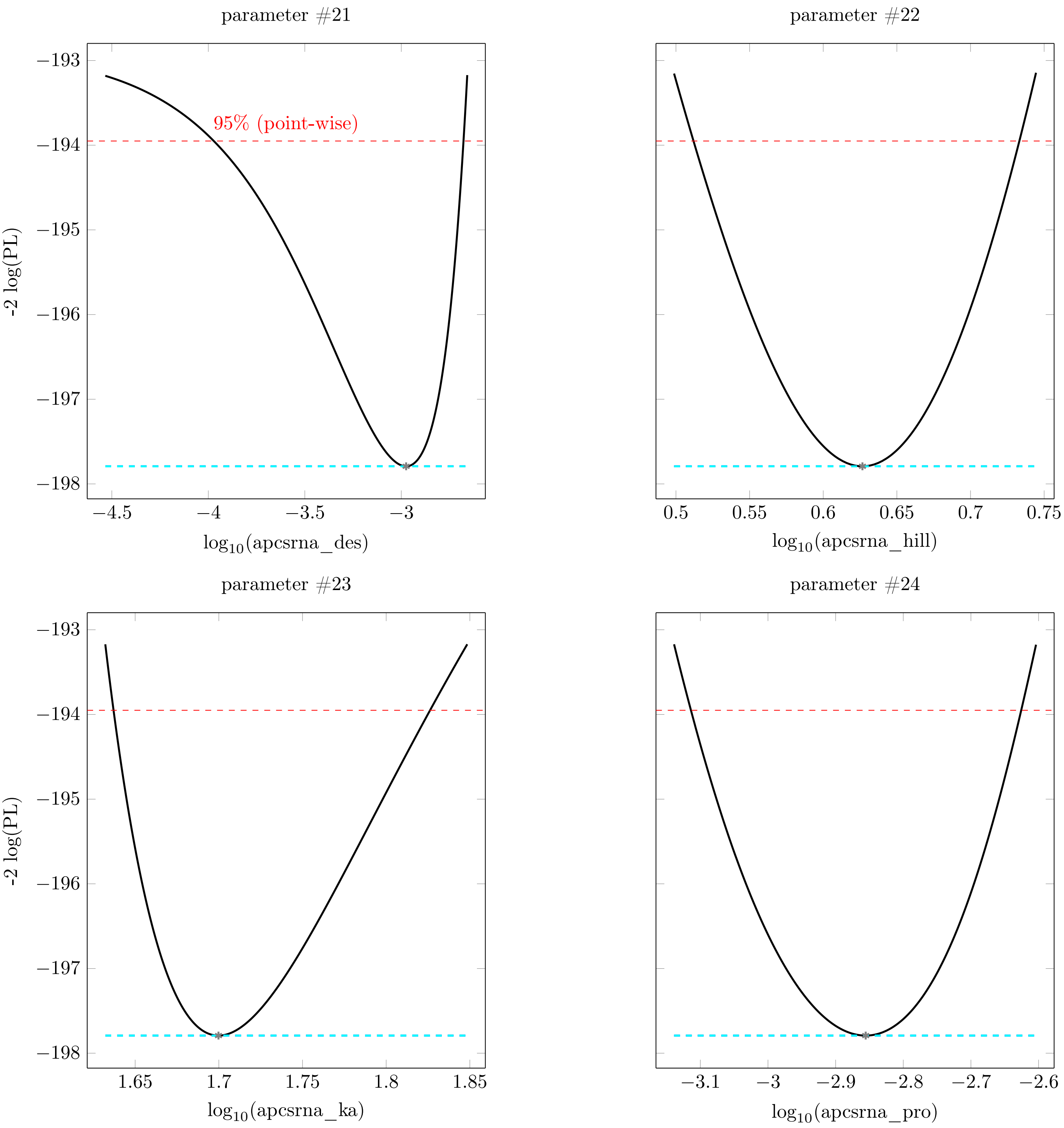

Figure S20: Overview of the profile likelihood of the APCS model parameters. The solid lines indicate the profile likelihood. The dashed lines indicate the threshold to assess confidence intervals. The asterisks indicate the optimal parameter values.

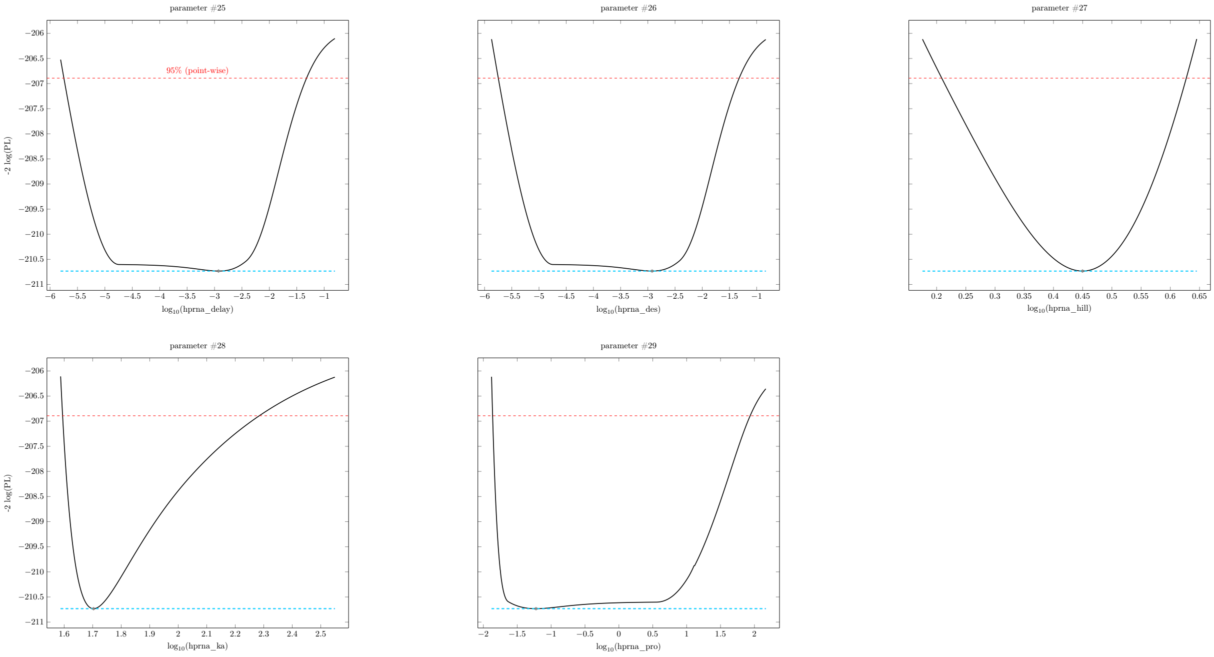

Figure S21: Overview of the profile likelihood of the HP model parameters. The solid lines indicate the profile likelihood. The dashed lines indicate the threshold to assess confidence intervals. The asterisks indicate the optimal parameter values.

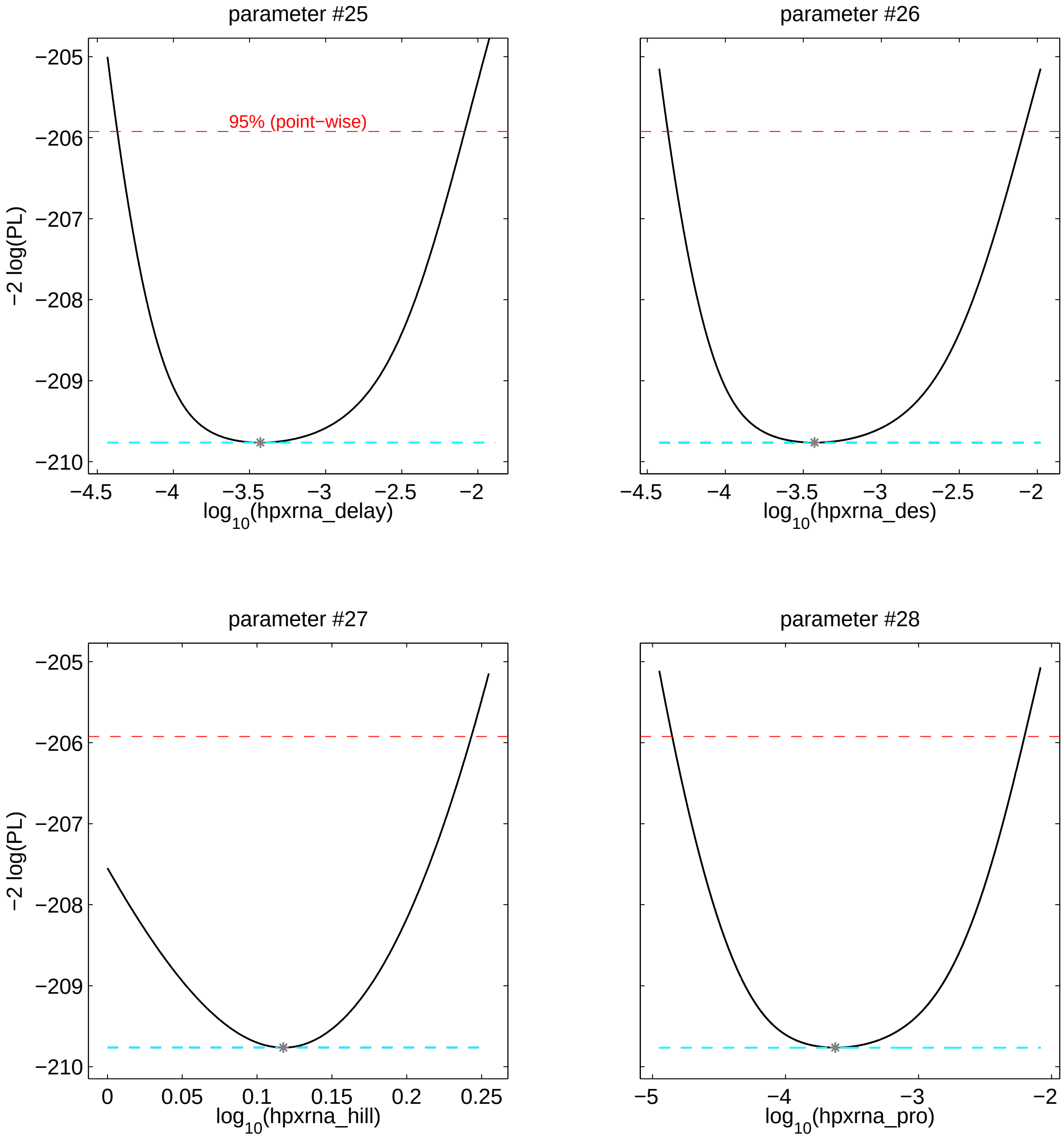

Figure S22: Overview of the profile likelihood of the HPX model parameters. The solid lines indicate the profile likelihood. The dashed lines indicate the threshold to assess confidence intervals. The asterisks indicate the optimal parameter values. For this APP gene model integration fails when the hill coefficient is chosen lower than 1.

3.6 Sensitivity Analysis

If one considers the acute phase proteins to be very stable, then the protein levels will represent the integral of APP expression. In order to assess how to optimally reduce the APP response induced by IL-6, we performed a sensitivity analysis on our model. In Figure S23 we see the average local parameter sensitivity of the integrated expression of APP genes with respect to different model parameters as well

as the parameter sensitivity of SOCS3. This local parameter sensitivity is defined as (72), where y^* refers to the model output at the perturbed parameter ($\theta^* = (\theta_1, \theta_2, ..., \theta_i \Delta, ..., \theta_n)$), while y indicates the reference output. Analogously, θ_i^* refers to the value of the parameter of interest in the perturbed condition, while θ_i indicates the reference parameter value.

The perturbation tested here is a 50% inhibition of the parameter. Positive values indicate that APP gene expression increases with the parameter value, while negative values indicate the opposite. This means that in order to obtain reduced APP gene expression, we should aim to either inhibit those parameters that exhibit positive sensitivity, while attempting to increase those that show a negative sensitivity.

To assess the uncertainty in the model sensitivities, we made use of the parameter sets obtained using the profile likelihood. For each profile likelihood we computed sensitivities for the parameter sets at the confidence interval. These sensitivities are denoted by circles.

$$S_i = \frac{(y^* - y)/y}{(\theta_i^* - \theta_i)} \tag{72}$$

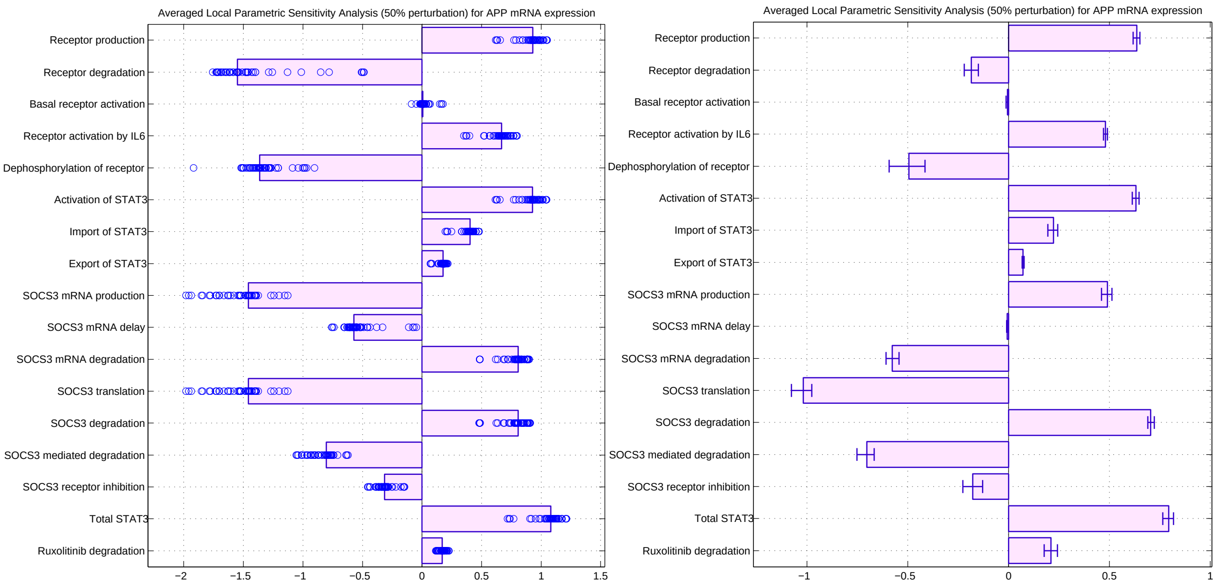

Figure S23: Parameter sensitivity of the integrated APP and SOCS3 mRNA response. Circles denote sensitivities computed using profile likelihood parameter sets corresponding at the confidence interval.

Based on sensitivity analysis, we find that additional options exist to further suppress the APP response. One such alternative would be to reduce the mRNA degradation rate of SOCS3 in order to benefit from synergistic effects between the pathway's natural inhibitor SOCS3 and the drug Ruxolitinib.

Shown in Figure S24 are the final APP dynamics. Note how the states which do not require a delay exhibit smoother dynamics and do not require preservation of the input function (the nuclear phospho-STAT3 dynamics).

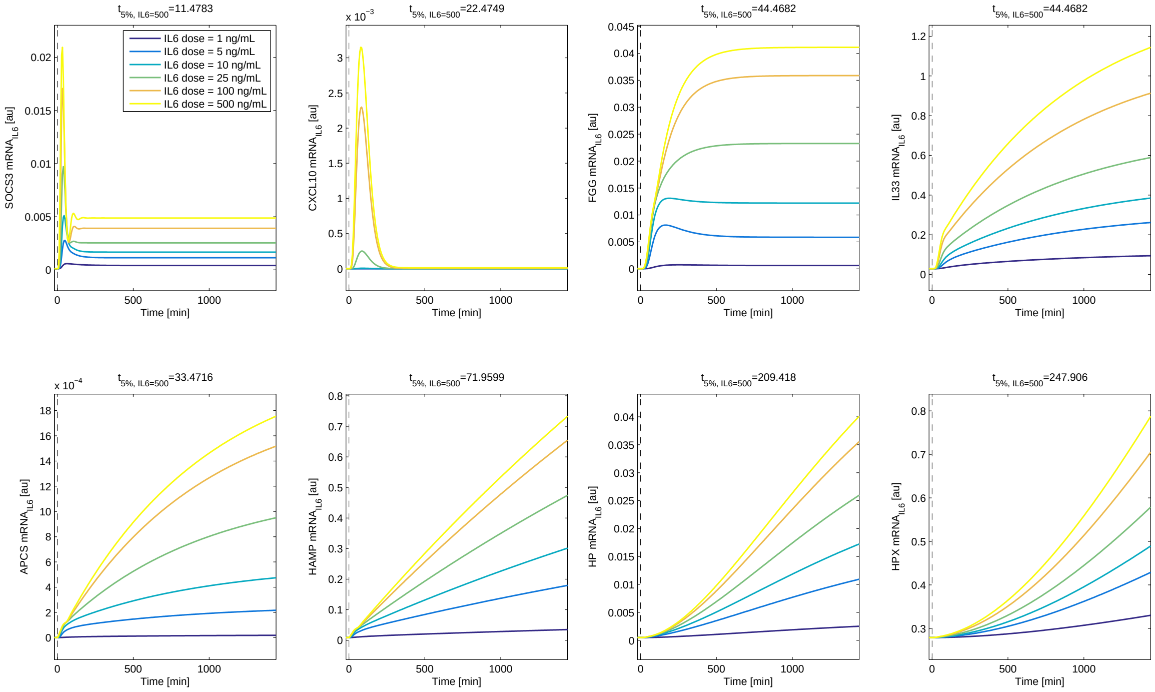

Figure S24: Dynamics of the APP genes in the dynamic model. The time indicated is the time to reach 5% of the final value at t = 24 hours. Most APP genes have not reached their steady state level at this point however.

3.7 Experiment Design

The developed model was used to perform experiment design, the aim of which is continuous suppression of elevated IL-6-induced APP gene expression. As an objective criterion, the integrals of the APP mRNA levels in response to 100 ng/ml IL-6 were used as a proxy for APP expression during inflammation. Based on the dose responses to Ruxolitinib, it could be concluded that the efficacy of inhibition depended monotonically on the Ruxolitinib dose. Hence, maximization of the dose of Ruxolitinib at every time point will lead to maximal inhibition. Since larger doses are expected to lead to more adverse effects, it was decided to spread the administration of Ruxolitinib over several doses. Since Ruxolitinib degradation is modelled by first order metabolization, the dose at each time point can be computed from a mono-exponential. Hence, for treatment planning, we are left with three degrees of freedom i.e. the different time points at which doses of Ruxolitinib are administered. We evaluated whether pre-treatment or treatment at the same time as IL-6 was more beneficial. In all cases, pretreatment led to longer high levels of APP expression, due to the fact that the Ruxolitinib had already partially degraded.

To determine the optimal dosage schedule, we discretized time and sampled the 3D volume representing the different dosage times. Figure S25, shows the integrated expression of the various APP genes with respect to choosing the initial dose at this time point, whilst choosing time point two and three most optimally. Hence, it shows the lowest attainable integrated APP expression when administering the first dose at this time point. Figure S26 shows the other two time points, given that the first time point is fixed at its most optimal point. Depending on which APP gene one would like to suppress, different dosage times may be optimal. Considering the sharp increase after t=0 for CXCL10, we decided to choose the time point for Ruxolitinib administration the same as that for IL-6. We decided to equally space the remaining two doses. However, as shown in Figure S25 and S26, when one is interested in targetting one APP gene specifically, different time points may be more optimal.

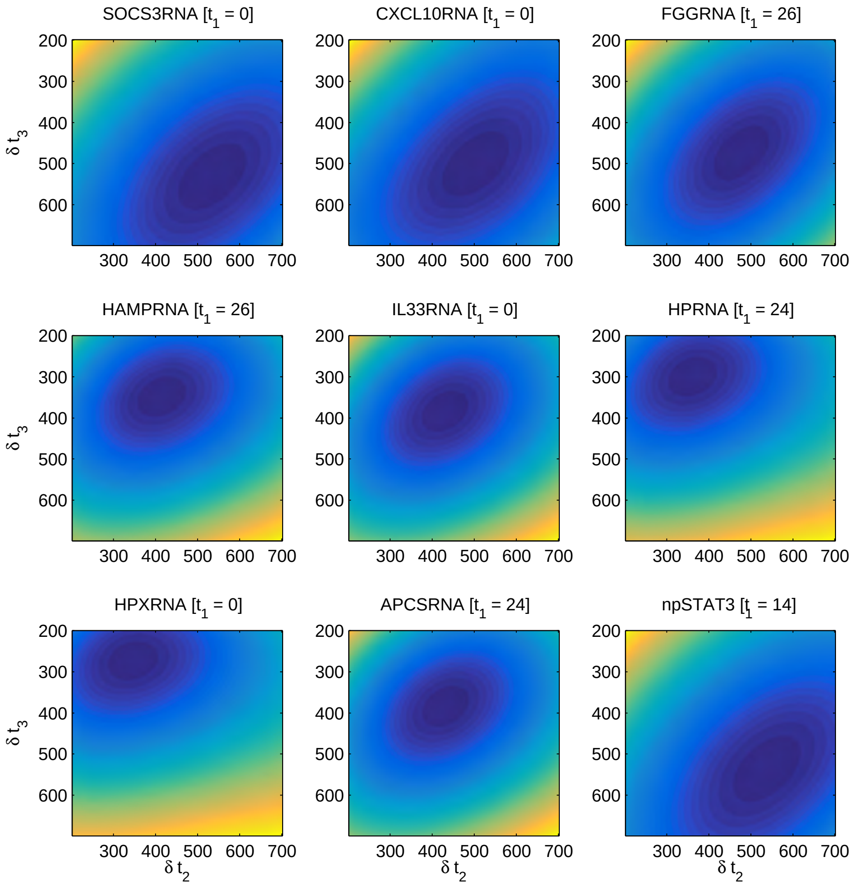

Figure S25: Dependency of integrated APP responses on initial treatment time. Shown in the graph is the lowest integrated signal attainable using triple dosing when the initial dose is given at the time point indicated on the axis.

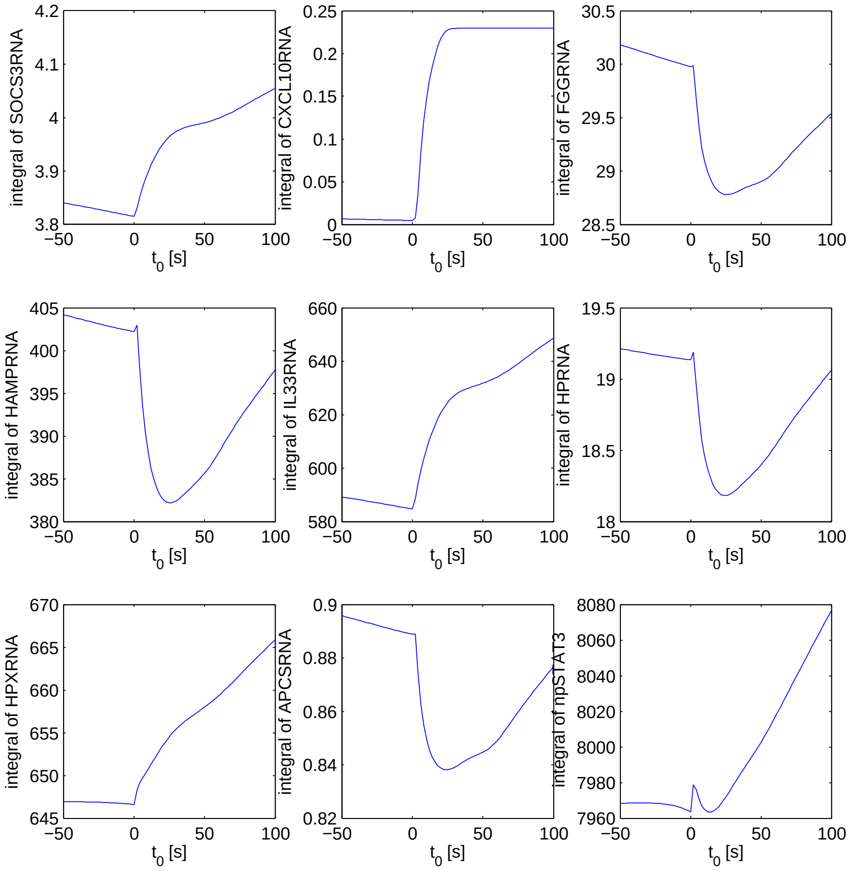

Figure S26: Dependency of the integrated APP responses on the time intervals between the three doses. The colors are proportional to the integrated APP response for the different APP genes with respect to the dosing times indicated on the axes. Deep blue indicates a low response, while yellow indicates a high (unfavorable) response. For each plot, the first dose is chosen at its most optimal value.

As an extra control, an optimization procedure was performed to optimally divide the 882 nM of Ruxolitinib (amount used in the triple treatment) over three arbitrarily placed time points with no constraint on how the doses are divided over the time interval (with the dosing time points constrained between -200 and 1440). The only constraint used was that the total may not exceed 882 nM (total amount used in the triple treatment). The objective function was the sum of the integral of all APP genes, where each gene integral was normalized to its expression integral without Ruxolitinib administration. To avoid getting trapped in local minima, we initialized this optimization over 10000 randomly chosen initial guess parameters uniformly dispersed over the design parameter ranges. As expected, the resulting optimal time schedule to achieve maximal APP mRNA suppresion with Ruxolitinib would be one single bolus. However, a single bolus is expected to lead to more adverse effects. This is why we decided to limit the maximum instantaneous dose, rather than the total dose.

4 Experiments

The following pages detail the different simulation experiments that the model was calibrated and/or validated with. Each page contains a brief description of the experiment, a plot with the model simulation, the raw data and additional information pertaining to the simulation protocol. Different simulation protocols are encoded by applying condition specific parameter and input transformations, which are listed in each of the experiment descriptions. Unlisted parameters either default to the transformation listed in the main model description 3.3, or to the raw parameter value as specified in the full table of model parameters (see 5). To get a global overview of the data involved in model parameterization, please refer to Table S9, for a description of which experiments refer to which states.

Table S9: Global overview of the used measurement data. Each line corresponds to a list of experiments that a particular model variable was observed in.

Variable	Datasets
JAK1_gp130	4.9
pJAK1_gp130	4.1 4.2 4.4 4.5 4.6 4.9 4.16 4.18 4.19
pJAK1_pgp130	4.1 4.2 4.4 4.5 4.6 4.9 4.10 4.11 4.12 4.13 4.14 4.15 4.16 4.17 4.18 4.19
STAT3	4.9 4.34 4.47
tpSTAT3	4.1 4.2 4.3 4.4 4.5 4.6 4.7 4.10 4.11 4.12 4.13 4.14 4.15 4.16 4.17 4.18 4.19 4.20 4.21 4.33 4.36 4.37 4.38 4.40 4.41 4.42 4.43 4.44 4.45 4.47
nSTAT3	4.47 4.67 4.68 4.69 4.70 4.71 4.72
SOCS3RNA	4.8 4.22 4.23 4.24 4.25 4.26 4.27 4.28 4.29 4.30 4.31 4.32 4.35 4.39 4.46 4.64 4.65 4.66
SOCS3	4.9 4.10 4.17 4.18 4.19
CXCL10RNA	4.48 4.55 4.56 4.57 4.58 4.63 4.64 4.65 4.66
FGGRNA	4.49 4.50 4.51 4.52 4.53 4.54 4.55 4.56 4.59 4.60 4.61 4.62 4.64 4.65 4.66
HAMPRNA	4.49 4.50 4.51 4.52 4.53 4.54 4.55 4.56 4.59 4.60 4.61 4.62 4.64 4.65 4.66
IL33RNA	4.49 4.50 4.51 4.52 4.53 4.54 4.55 4.56 4.59 4.60 4.61 4.62 4.64 4.65 4.66
APCSRNA	4.52 4.53 4.54 4.55 4.56 4.61 4.62 4.64 4.65 4.66
HPRNA	4.52 4.53 4.54 4.55 4.56 4.61 4.62 4.64 4.65 4.66
HPXRNA	4.52 4.53 4.54 4.55 4.56 4.61 4.62 4.64 4.65 4.66

4.1 Experiment: bohl hep 2005 02 03 Cont40ng T90min

4.1.1 Comments

Experimenter: Sebastian Bohl
Date: 2005-02-03
Cells: primary mouse hepatocytes
Treatment: IL-6 40 ng/ml constant

4.1.2 Model fit and plots

The model observables and the experimental data is shown in Figure S27.

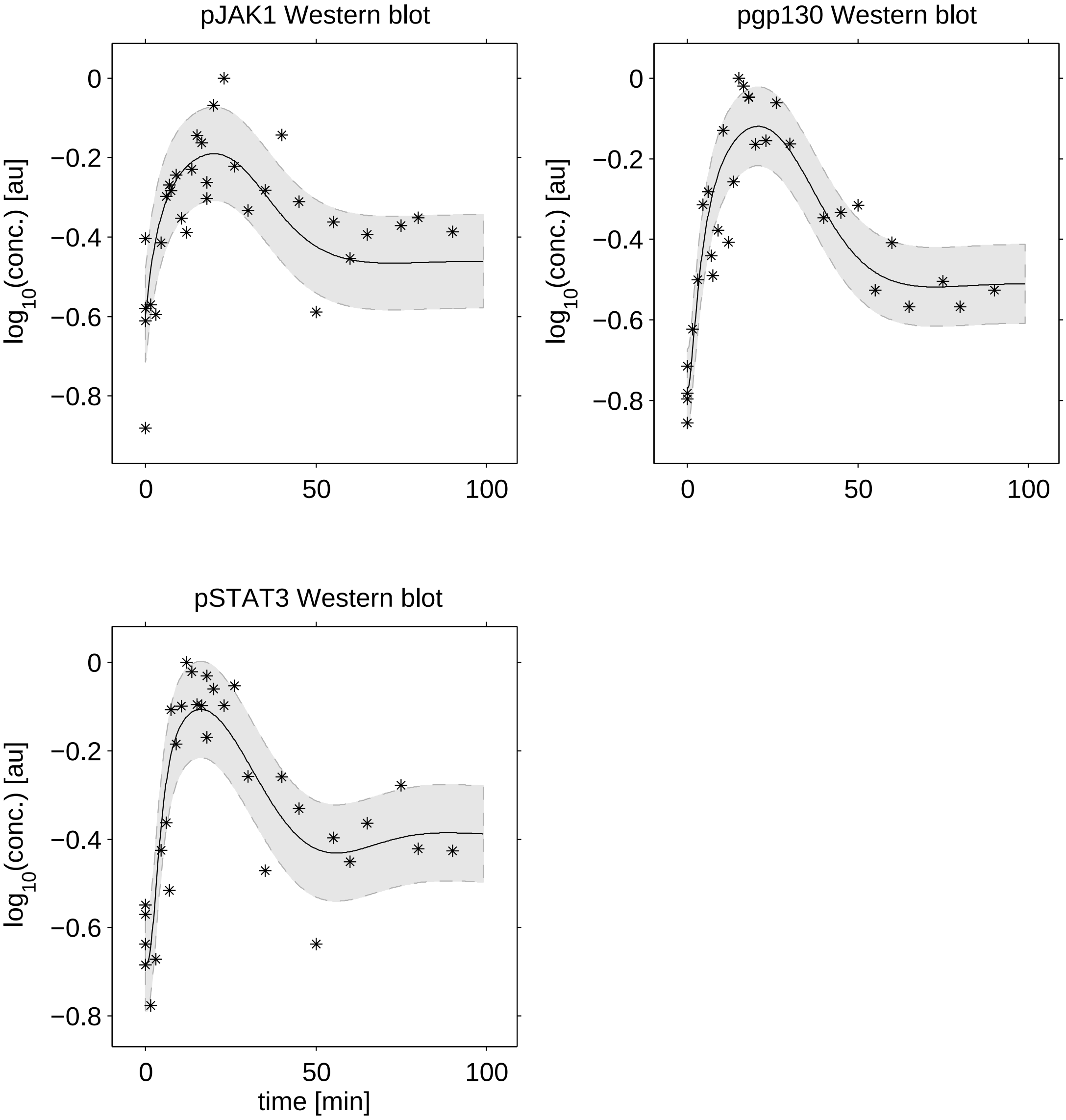

Figure S27: bohl_hep_2005_02_03_Cont40ng_T90min observables and experimental data for the experiment.

4.2 Experiment: bohl hep 2004 10 26 Cont 30min

4.2.1 Comments

Experimenter: Sebastian Bohl
Date: 2004-10-26
Cells: primary mouse hepatocytes
Treatment: IL-6 40 ng/ml constant

4.2.2 Model fit and plots

The model observables and the experimental data is shown in Figure S28.

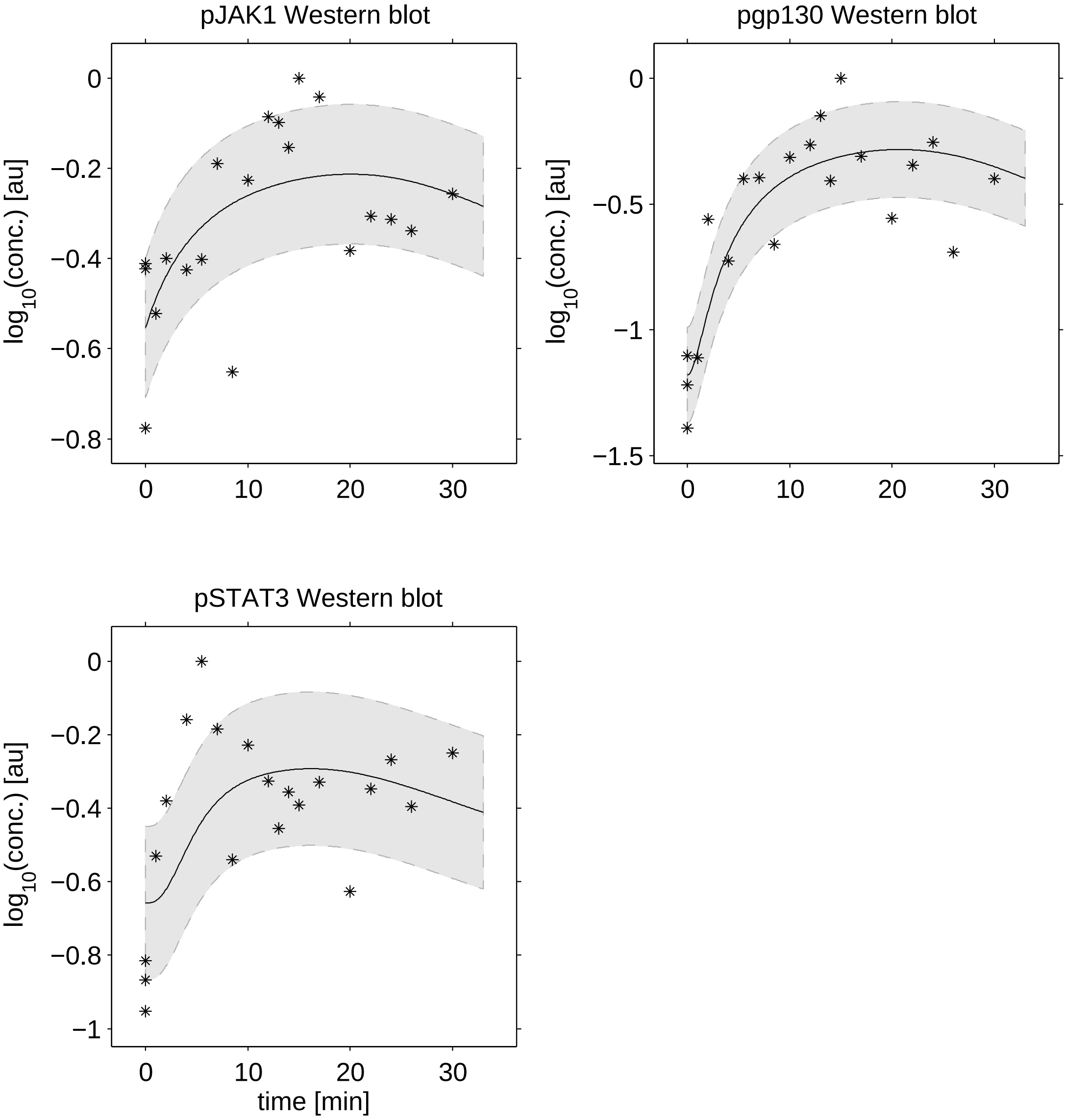

Figure S28: bohl_hep_2004_10_26_Cont_30min observables and experimental data for the experiment.

4.3 Experiment: bohl hep 2005 02 14 DoseResponse T15min

4.3.1 Comments

Experimenter: Sebastian Bohl
Date: 2005-02-14
Cells: primary mouse hepatocytes
Treatment: IL-6 dose response

4.3.2 Model fit and plots

The model observables and the experimental data is shown in Figure S29. The necessary parameter transformation is listed in Table S10.

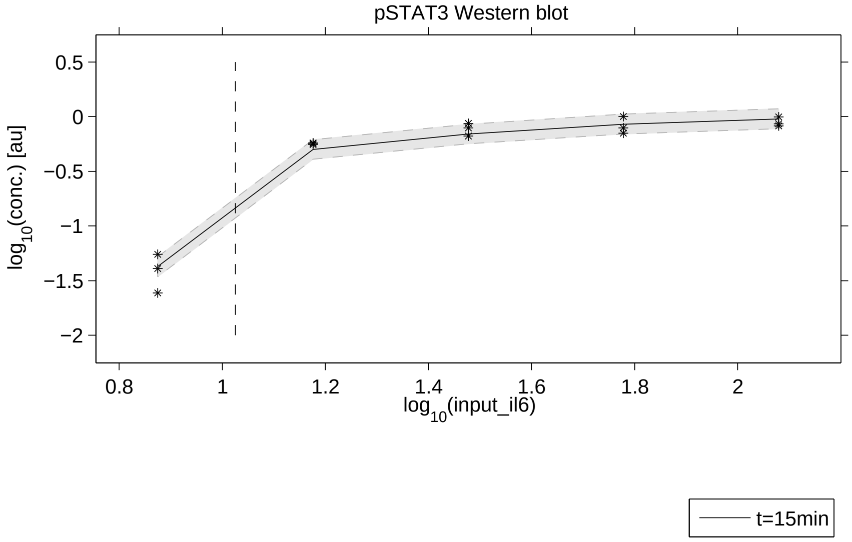

Figure S29: bohl_hep_2005_02_14_DoseResponse_T15min observables and experimental data for the experiment.

4.3.3 Condition dependent parameter changes

The following model parameters were changed to simulate these experimental conditions:

Parameter	Condition values				
input_il6	0	120	15	30	60

Table S10: Model parameters modified for experiment bohl hep 2005 02 14 DoseResponse T15min. Different columns indicate different conditions.

4.4 Experiment: bohl hep 2005 03 01 Pulse20min

4.4.1 Comments

Experimenter: Sebastian Bohl
Date: 2005-03-01
Cells: primary mouse hepatocytes
Treatment: IL-6 40 ng/ml 20 min pulse

4.4.2 Model fit and plots

The model observables and the experimental data is shown in Figure S30. This experiment requires a custom input function which is defined in Table S11.

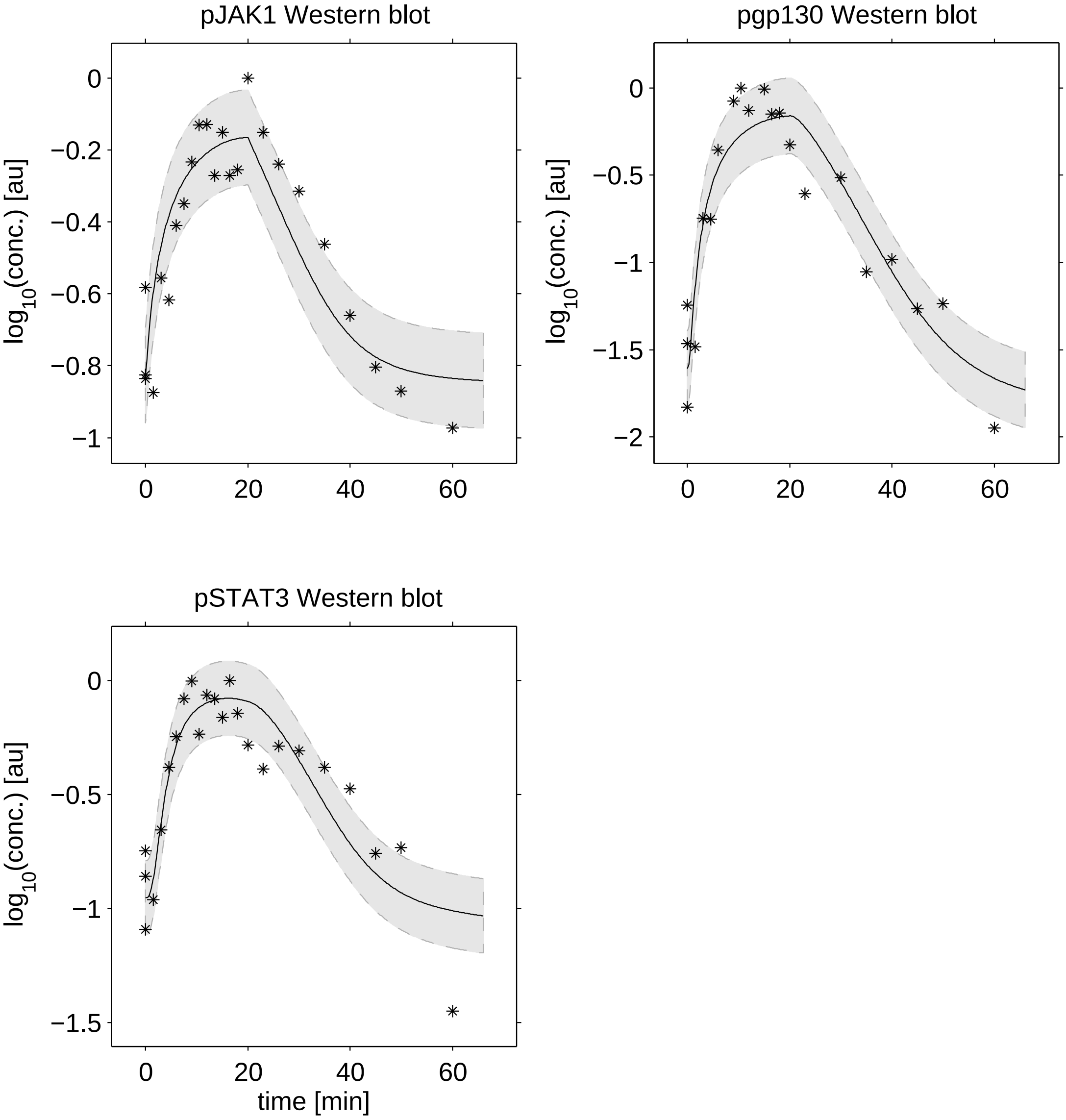

Figure S30: bohl_hep_2005_03_01_Pulse20min observables and experimental data for the experiment.

Input	Unit	Modified equation
IL6	conc. [ng/ml]	$40 - 40 \cdot \text{heaviside}(t - 20)$

Table S11: Inputs modified for experiment bohl_hep_2005_03_01_Pulse20min

4.5 Experiment: bohl hep 2005 03 16 Cont40ng 23min

4.5.1 Comments

Experimenter: Sebastian Bohl
Date: 2005-03-16
Cells: primary mouse hepatocytes
Treatment: IL-6 40 ng/ml constant

4.5.2 Model fit and plots

The model observables and the experimental data is shown in Figure S31.

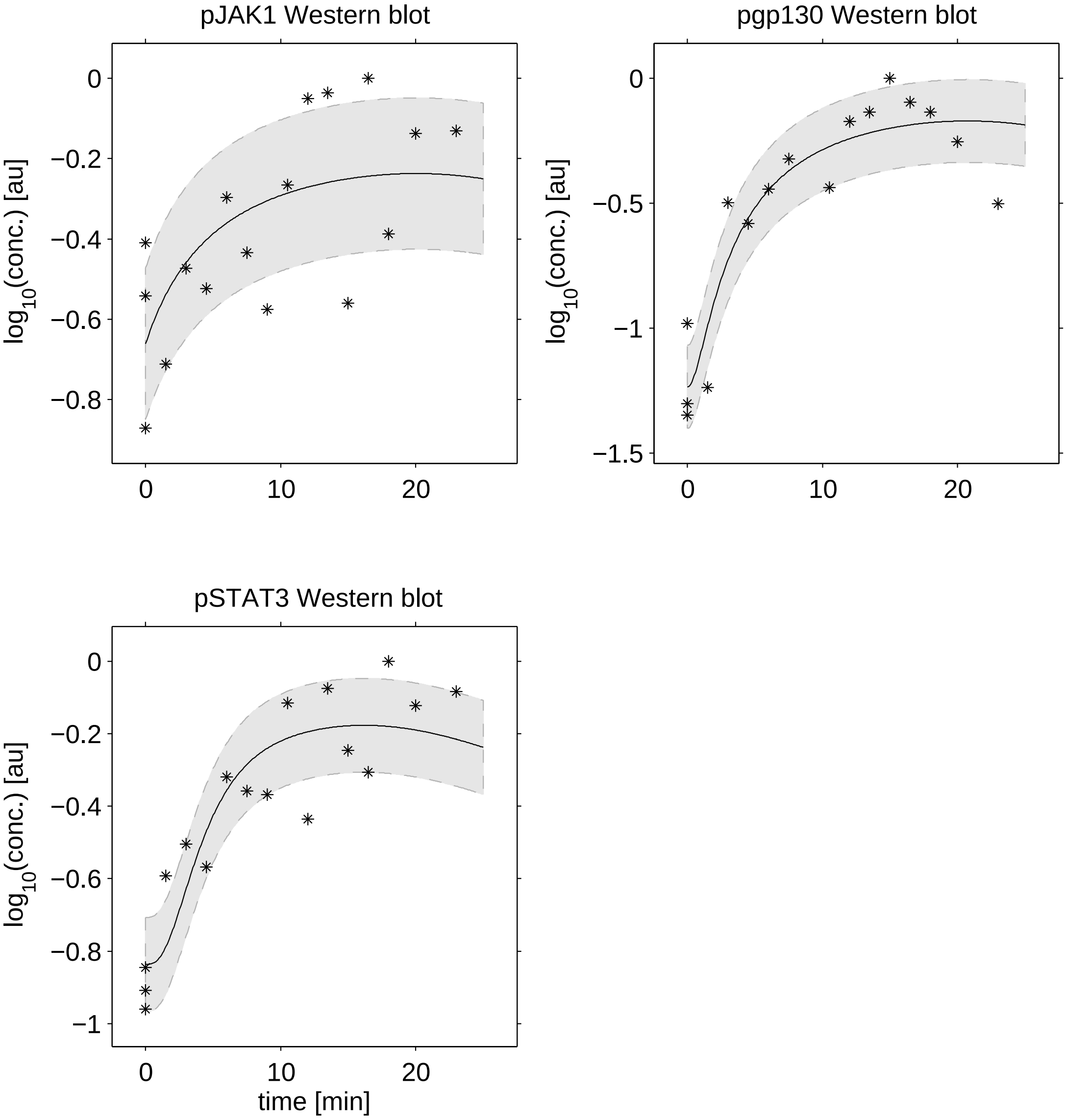

Figure S31: bohl_hep_2005_03_16_Cont40ng_23min observables and experimental data for the experiment.

4.6 Experiment: bohl hep 2005 07 26 Pulse5min

4.6.1 Comments

Experimenter: Sebastian Bohl
Date: 2005-07-26
Cells: primary mouse hepatocytes
Treatment: IL-6 40 ng/ml 5 min pulse

4.6.2 Model fit and plots

The model observables and the experimental data is shown in Figure S32. This experiment requires a custom input function which is defined in Table S12.

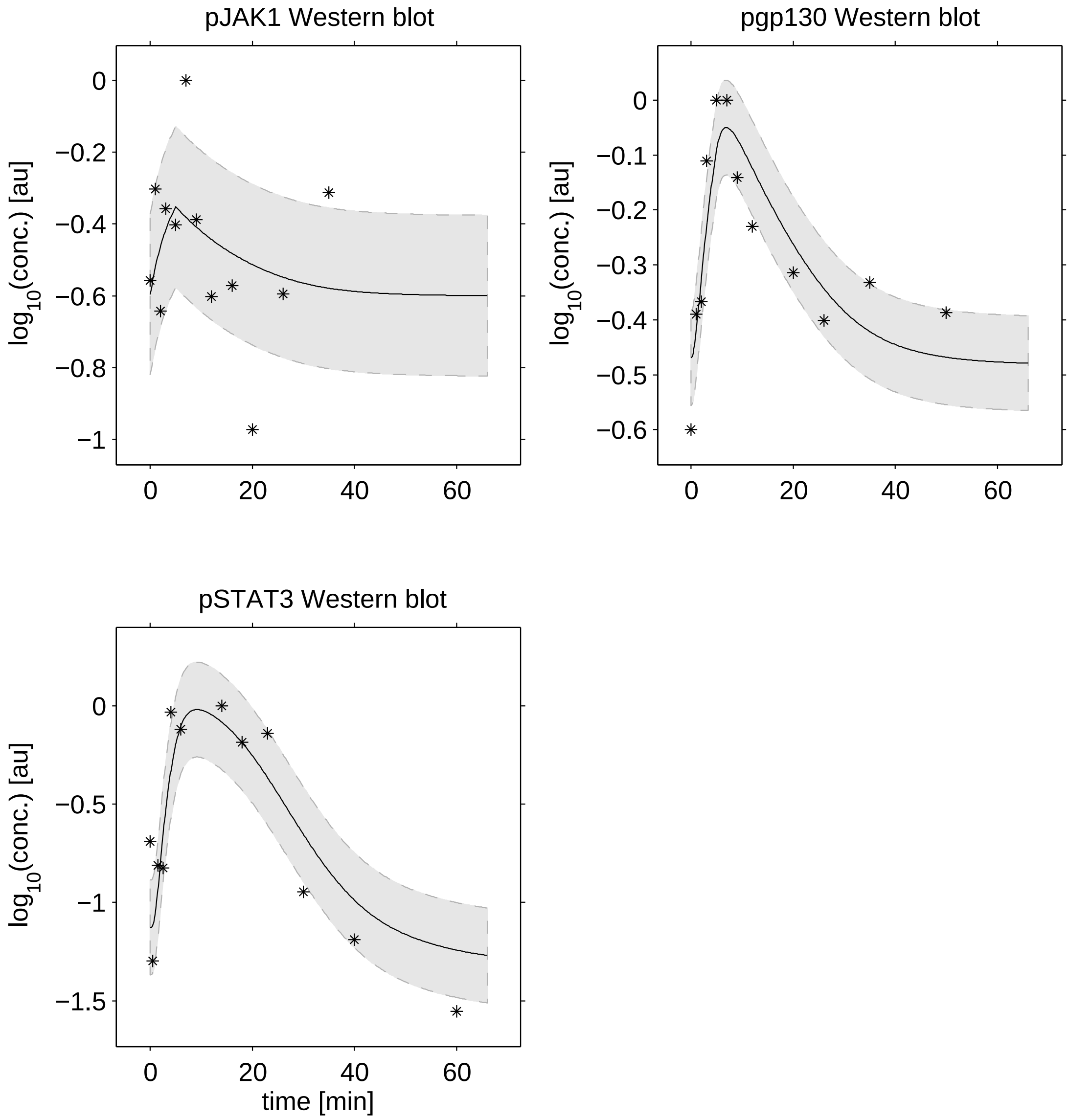

Figure S32: bohl_hep_2005_07_26_Pulse5min observables and experimental data for the experiment.

Input	Unit	Modified equation
IL6	conc. [ng/ml]	$40 - 40 \cdot \text{heaviside}(t - 5)$

Table S12: Inputs modified for experiment bohl_hep_2005_07_26_Pulse5min

4.7 Experiment: bohl hep 2005 11 22 DoseResponseUntil120ng

4.7.1 Comments

Experimenter: Sebastian Bohl
Date: 2005-11-22
Cells: primary mouse hepatocytes
Treatment: IL-6 dose response

4.7.2 Model fit and plots

The model observables and the experimental data is shown in Figure S33. The necessary parameter transformation is listed in Table S13.

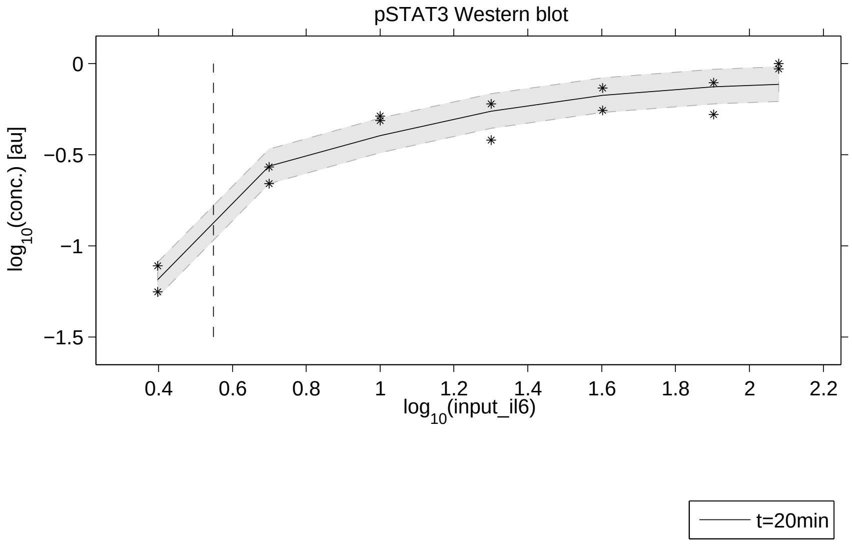

Figure S33: bohl_hep_2005_11_22_DoseResponseUntil120ng observables and experimental data for the experiment.

4.7.3 Condition dependent parameter changes

The following model parameters were changed to simulate these experimental conditions:

Parameter	Condition values						
input_il6	0	10	120	20	40	5	80

Table S13: Model parameters modified for experiment bohl hep 2005 11 22 DoseResponseUntil120ng. Different columns indicate different conditions.

4.8 Experiment: bohl hep 2009 10 14 qRTPCR

4.8.1 Comments

Experimenter: Sebastian Bohl
Date: 2009-10-14
Cells: primary mouse hepatocytes
Treatment: IL-6 40 ng/ml = 1 nmol/l
values normalized by HPRT
biological replicates

4.8.2 Model fit and plots

The model observables and the experimental data is shown in Figure S34.

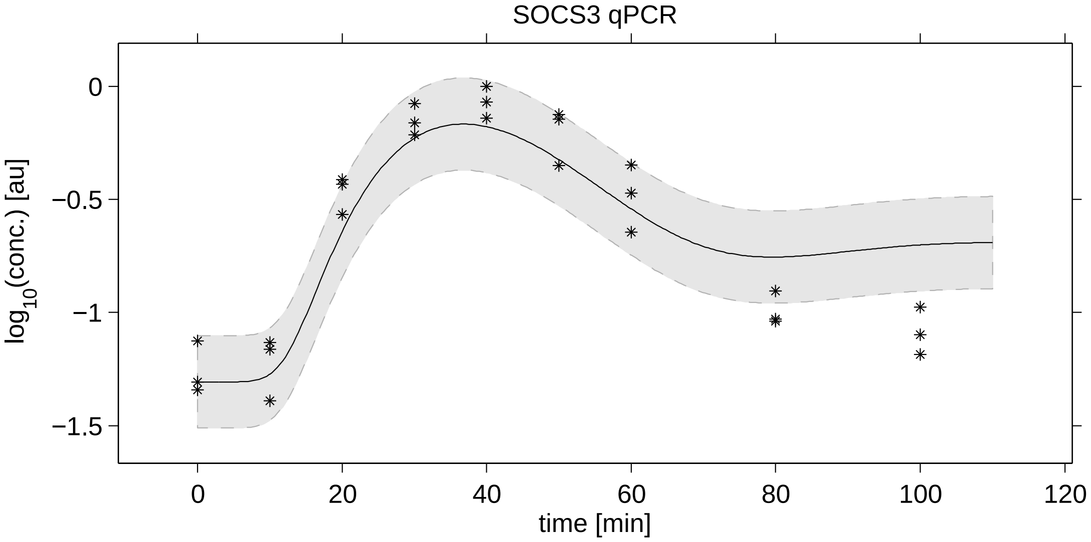

Figure S34: bohl_hep_2009_10_14_qRTPCR observables and experimental data for the experiment.

4.9 Experiment: bohl mlc per cell log

4.9.1 Comments

Experimenter: Sebastian Bohl
Date: 2007-09-05 (summary)
Cells: primary mouse hepatocytes
Treatment: IL-6 40 to 625 ng/ml constant

4.9.2 Model fit and plots

The model observables and the experimental data is shown in Figure S35.

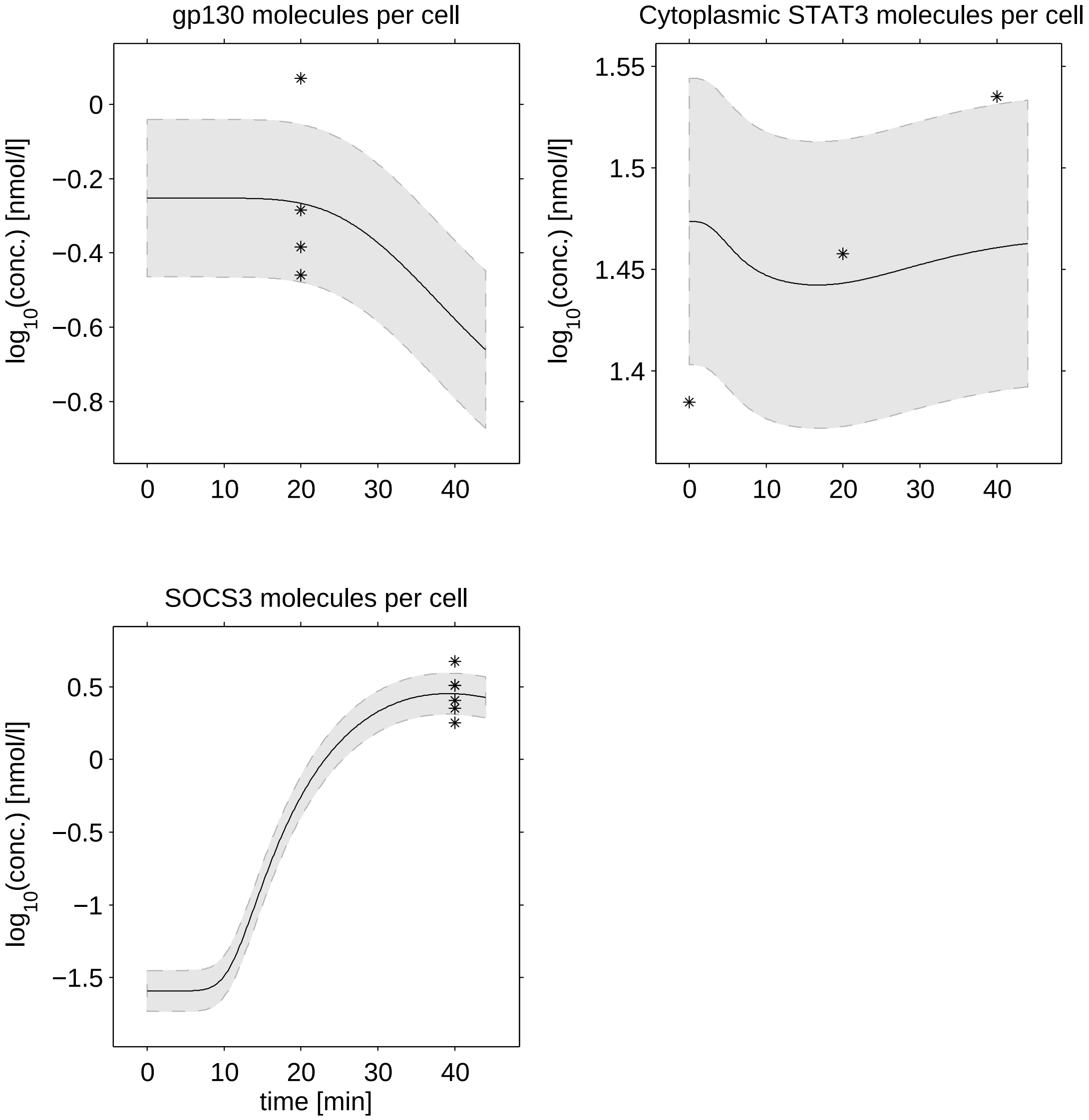

Figure S35: bohl_mlc_per_cell_log observables and experimental data for the experiment.

4.10 Experiment: bohl hep 2007 07 10 ActD

4.10.1 Comments

Experimenter: Sebastian Bohl
Date: 2007-07-10
Cells: primary mouse hepatocytes
Treatment: IL-6 80 ng/ml constant + Act-D treatment

4.10.2 Model fit and plots

The model observables and the experimental data is shown in Figure S36. The 2 necessary parameter transformations are listed in Table S14. Note that transformations with only one entry are the same for all experimental conditions corresponding to this experiment.

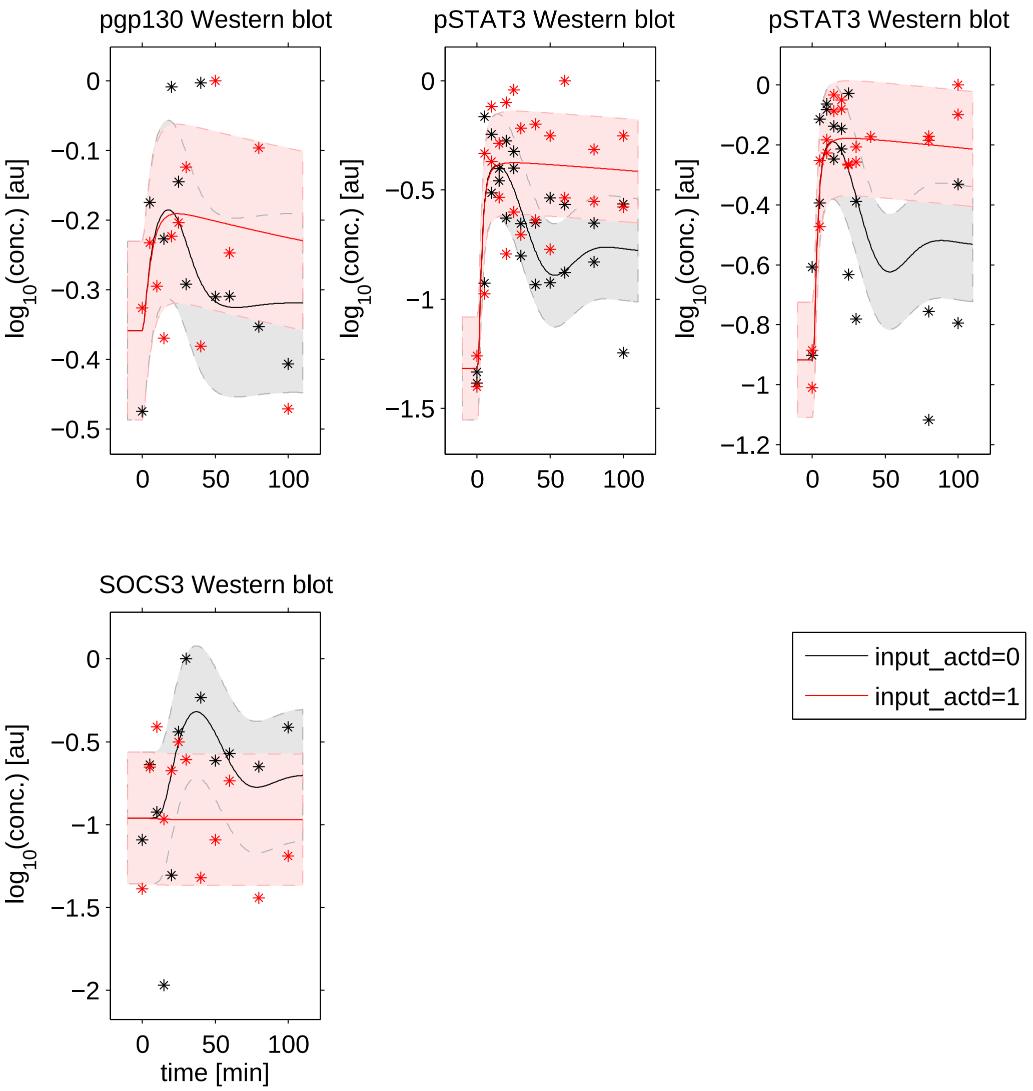

Figure S36: bohl_hep_2007_07_10_ActD observables and experimental data for the experiment.

4.10.3 Condition dependent parameter changes

The following model parameters were changed to simulate these experimental conditions:

Parameter	Condition values	
input_il6	80	
input_actd	0	1

Table S14: Model parameters modified for experiment bohl hep 2007 07 10 ActD. Different columns indicate different conditions.

4.11 Experiment: bohl hep 2006 06 07 ActD

4.11.1 Comments

Experimenter: Sebastian Bohl
Date: 2006-06-07
Cells: primary mouse hepatocytes
Treatment: IL-6 80 ng/ml constant + 3 different Act-D treatment

4.11.2 Model fit and plots

The model observables and the experimental data is shown in Figure S37. This experiment requires a custom input function which is defined in Table S15. The 3 necessary parameter transformations are listed in Table S16. Note that transformations with only one entry are the same for all experimental conditions corresponding to this experiment.

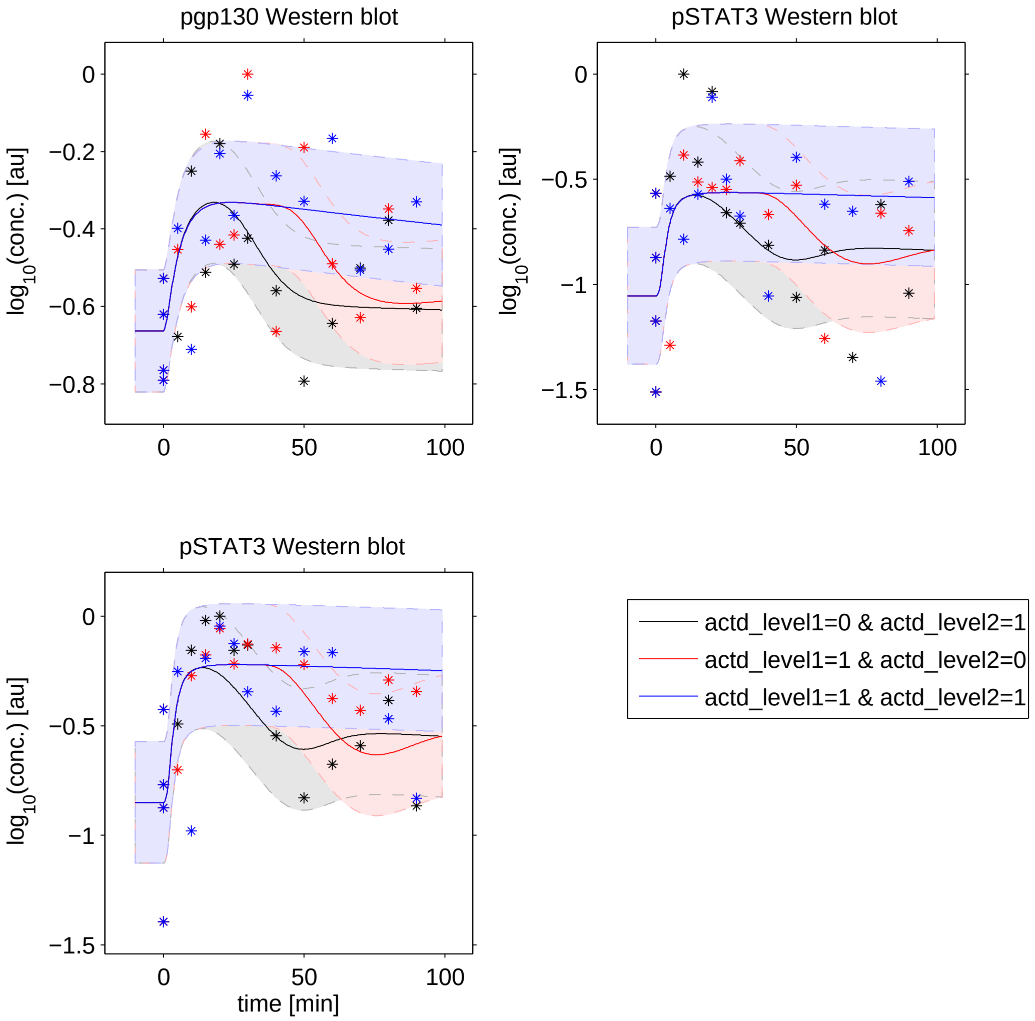

Figure S37: bohl_hep_2006_06_07_ActD observables and experimental data for the experiment.

Input	Unit	Modified equation
ActD	boolean [on/off]	actd_level1 · heaviside(t) − heaviside(t − 30) · (actd_level1 − actd_level2)

Table S15: Inputs modified for experiment bohl_hep_2006_06_07_ActD

4.11.3 Condition dependent parameter changes

The following model parameters were changed to simulate these experimental conditions:

Parameter	Condition values		
input_il6	80		
actd_level1	0	1	1
actd_level2	1	0	1

Table S16: Model parameters modified for experiment bohl hep 2006 06 07 ActD. Different columns indicate different conditions.

4.12 Experiment: bohl hep 2006 02 09 FourPulses ReceptorNucleus

4.12.1 Comments

Experimenter: Sebastian Bohl
Date: 2006-02-09
Cells: primary mouse hepatocytes
Treatment: IL-6 80 ng/ml 6-12-24-90 min pulses

4.12.2 Model fit and plots

The model observables and the experimental data is shown in Figure S38. This experiment requires a custom input function which is defined in Table S17. The necessary parameter transformation is listed in Table S18.

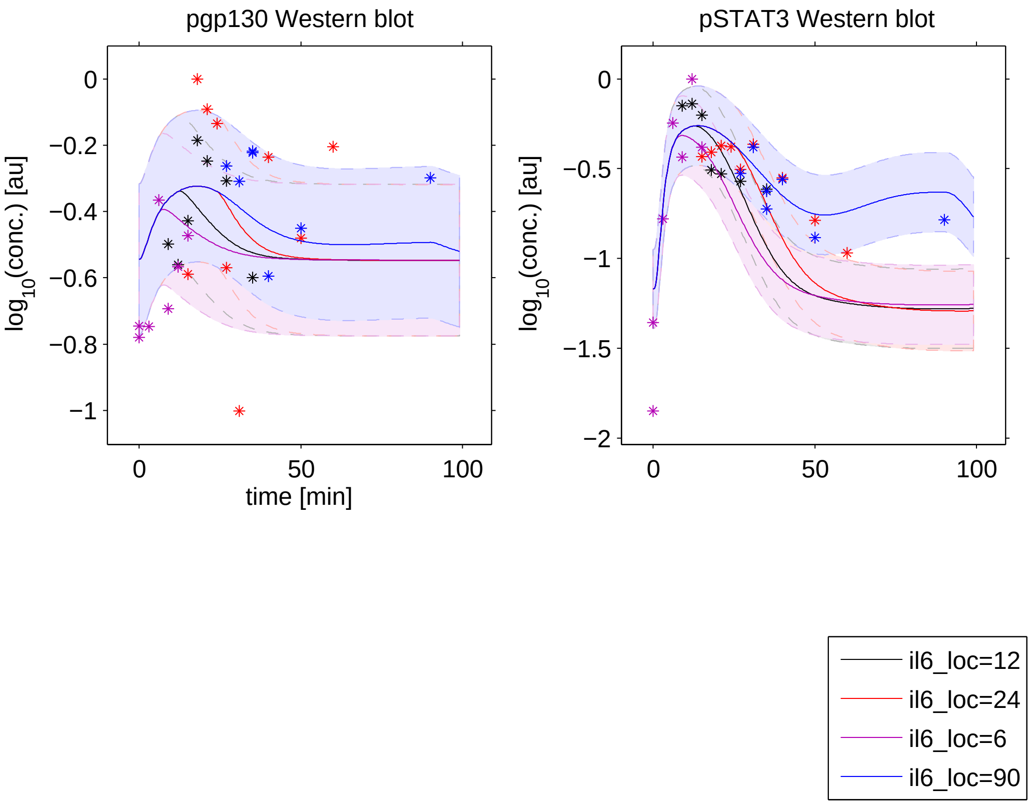

Figure S38: bohl_hep_2006_02_09_FourPulses_ReceptorNucleus observables and experimental data for the experiment.

Input	Unit	Modified equation
IL6	conc. [ng/ml]	$80 - 80 \cdot \text{heaviside}(t - \text{il6\_loc})$

Table S17: Inputs modified for experiment bohl_hep_2006_02_09_FourPulses_ReceptorNucleus

4.12.3 Condition dependent parameter changes

The following model parameters were changed to simulate these experimental conditions:

Parameter	Condition values			
il6_loc	12	24	6	90

Table S18: Model parameters modified for experiment bohl hep 2006 02 09 FourPulses ReceptorNucleus. Different columns indicate different conditions.

4.13 Experiment: bohl hep 2006 04 20 ThreePulses ThreeDoses

4.13.1 Comments

Experimenter: Sebastian Bohl
Date: 2006-04-20
Cells: primary mouse hepatocytes
Treatment: IL-6 30-60-120 ng/ml 10-20-40 min pulses

4.13.2 Model fit and plots

The model observables and the experimental data is shown in Figure S39. This experiment requires a custom input function which is defined in Table S19. The 2 necessary parameter transformations are listed in Table S20. Note that transformations with only one entry are the same for all experimental conditions corresponding to this experiment.

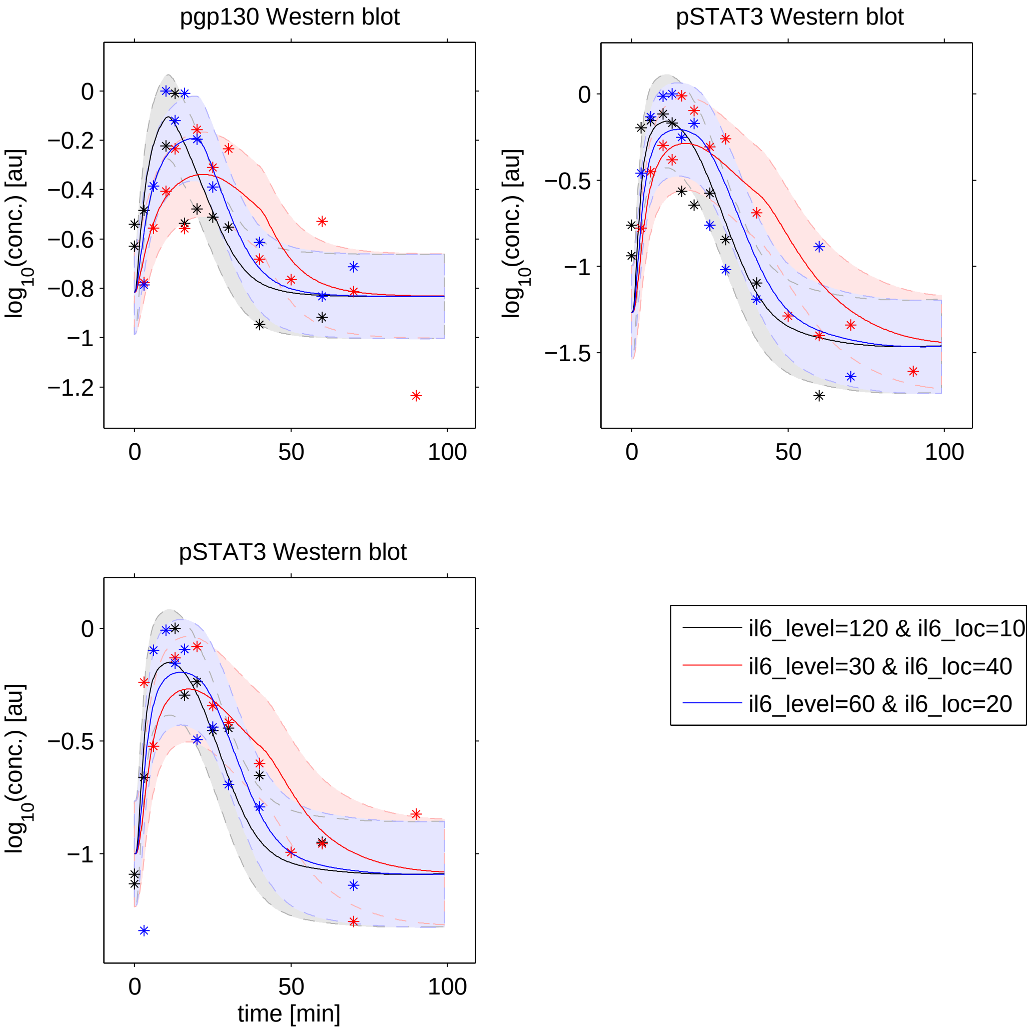

Figure S39: bohl_hep_2006_04_20_ThreePulses_ThreeDoses observables and experimental data for the experiment.

Input	Unit	Modified equation
IL6	conc. [ng/ml]	il6_level − il6_level · heaviside(t − il6_loc)

Table S19: Inputs modified for experiment bohl_hep_2006_04_20_ThreePulses_ThreeDoses

4.13.3 Condition dependent parameter changes

The following model parameters were changed to simulate these experimental conditions:

Parameter	Condition values		
il6_level	120	30	60
il6_loc	10	40	20

Table S20: Model parameters modified for experiment bohl hep 2006 04 20 ThreePulses ThreeDoses. Different columns indicate different conditions.

4.14 Experiment: bohl hep 2006 05 16 ThreeDoses

4.14.1 Comments

Experimenter: Sebastian Bohl
Date: 2006-05-16
Cells: primary mouse hepatocytes
Treatment: IL-6 20-40-80 ng/ml 20 min pulses

4.14.2 Model fit and plots

The model observables and the experimental data is shown in Figure S40. This experiment requires a custom input function which is defined in Table S21. The necessary parameter transformation is listed in Table S22.

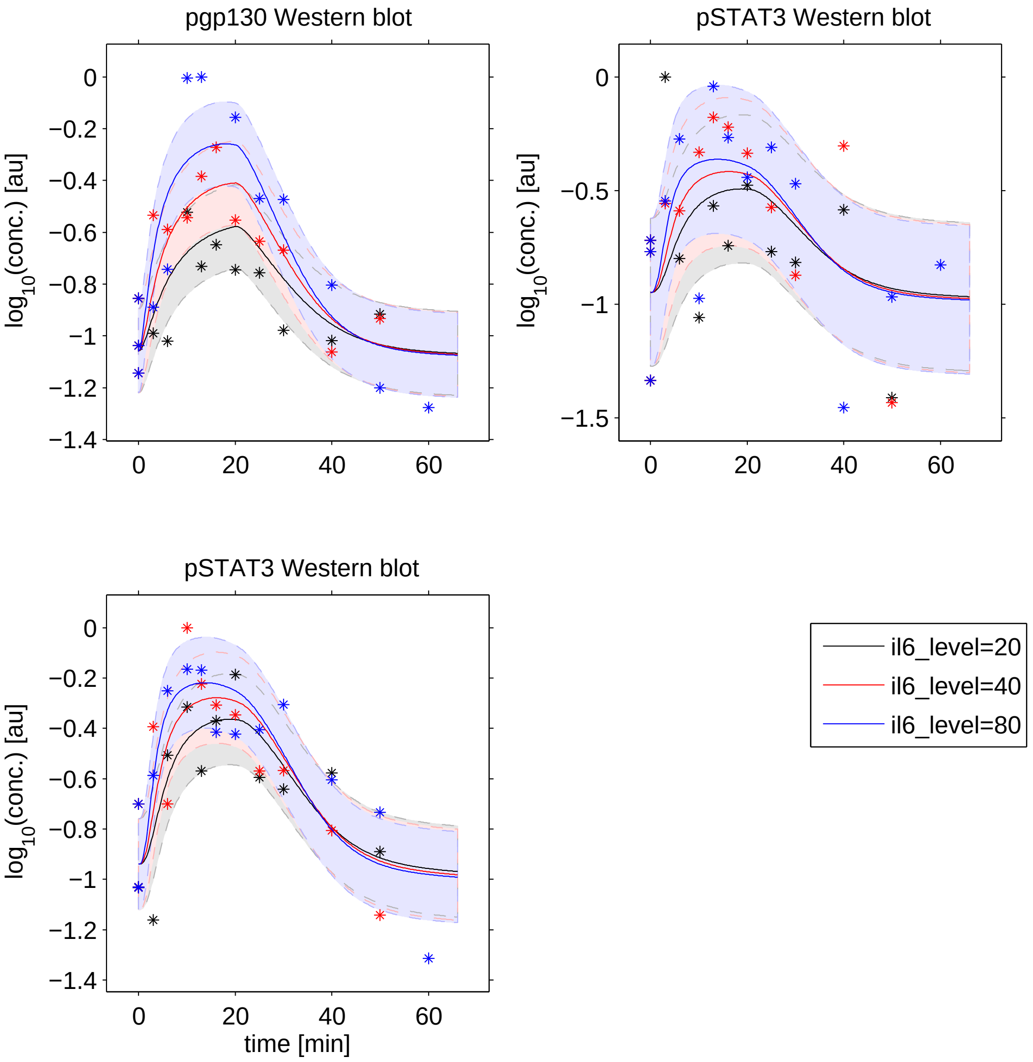

Figure S40: bohl_hep_2006_05_16_ThreeDoses observables and experimental data for the experiment.

Input	Unit	Modified equation
IL6	conc. [ng/ml]	il6_level − il6_level · heaviside(t − 20)

Table S21: Inputs modified for experiment bohl_hep_2006_05_16_ThreeDoses

4.14.3 Condition dependent parameter changes

The following model parameters were changed to simulate these experimental conditions:

Parameter	Condition values		
il6_level	20	40	80

Table S22: Model parameters modified for experiment bohl hep 2006 05 16 ThreeDoses. Different columns indicate different conditions.

4.15 Experiment: bohl hep 2006 11 15 FourPulses 120min

4.15.1 Comments

Experimenter: Sebastian Bohl
Date: 2006-11-15
Cells: primary mouse hepatocytes
Treatment: IL-6 40 ng/ml 6-12-24-120 min pulses

4.15.2 Model fit and plots

The model observables and the experimental data is shown in Figure S41. This experiment requires a custom input function which is defined in Table S23. The necessary parameter transformation is listed in Table S24.

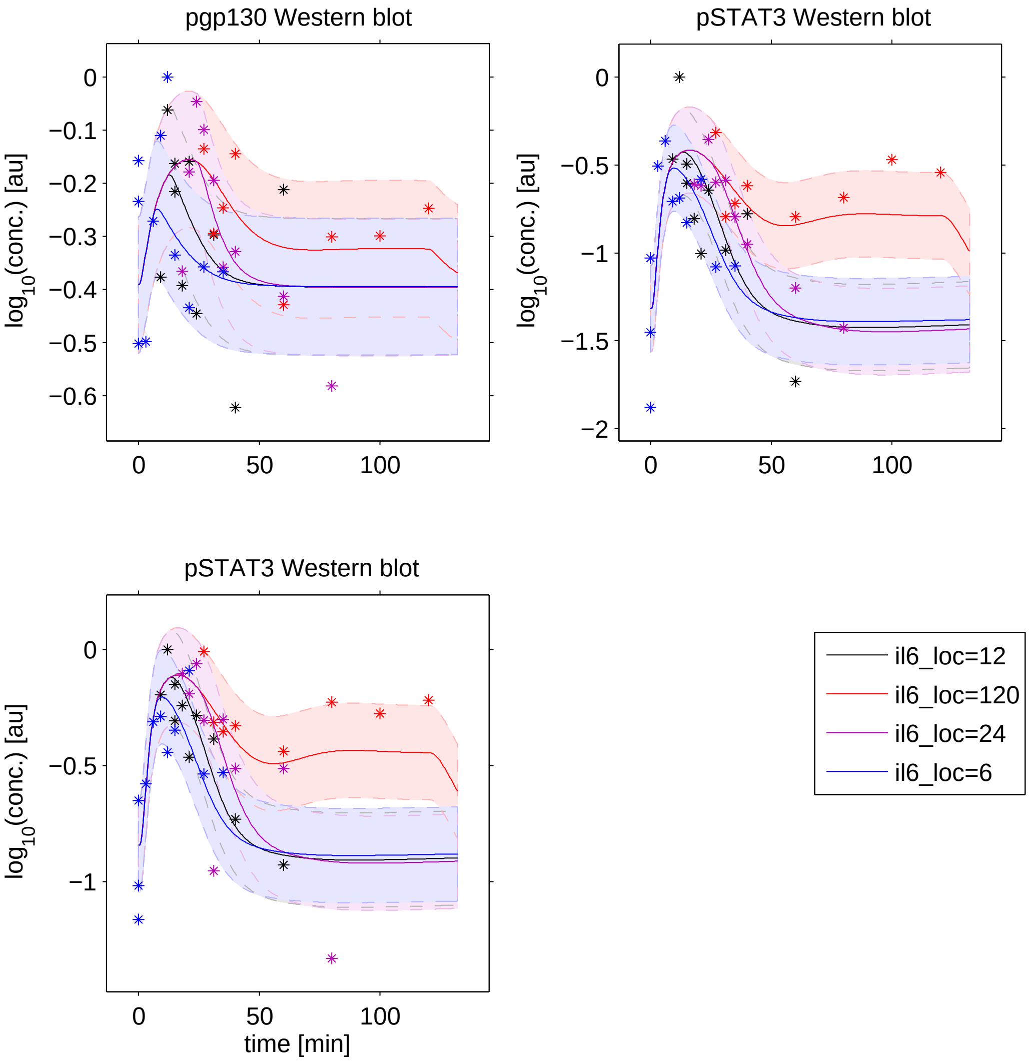

Figure S41: bohl_hep_2006_11_15_FourPulses_120min observables and experimental data for the experiment.

Input	Unit	Modified equation
IL6	conc. [ng/ml]	$40 - 40 \cdot \text{heaviside}(t - \text{il6\_loc})$

Table S23: Inputs modified for experiment bohl_hep_2006_11_15_FourPulses_120min

4.15.3 Condition dependent parameter changes

The following model parameters were changed to simulate these experimental conditions:

Parameter	Condition values			
il6_loc	12	120	24	6

Table S24: Model parameters modified for experiment bohl hep 2006 11 15 FourPulses 120min. Different columns indicate different conditions.

4.16 Experiment: bohl hep 2007 04 04 DoseResponse 3TP

4.16.1 Comments

Experimenter: Sebastian Bohl
Date: 2007-04-04
Cells: primary mouse hepatocytes
Treatment: IL-6 dose response

4.16.2 Model fit and plots

The model observables and the experimental data is shown in Figure S42. The necessary parameter transformation is listed in Table S25.

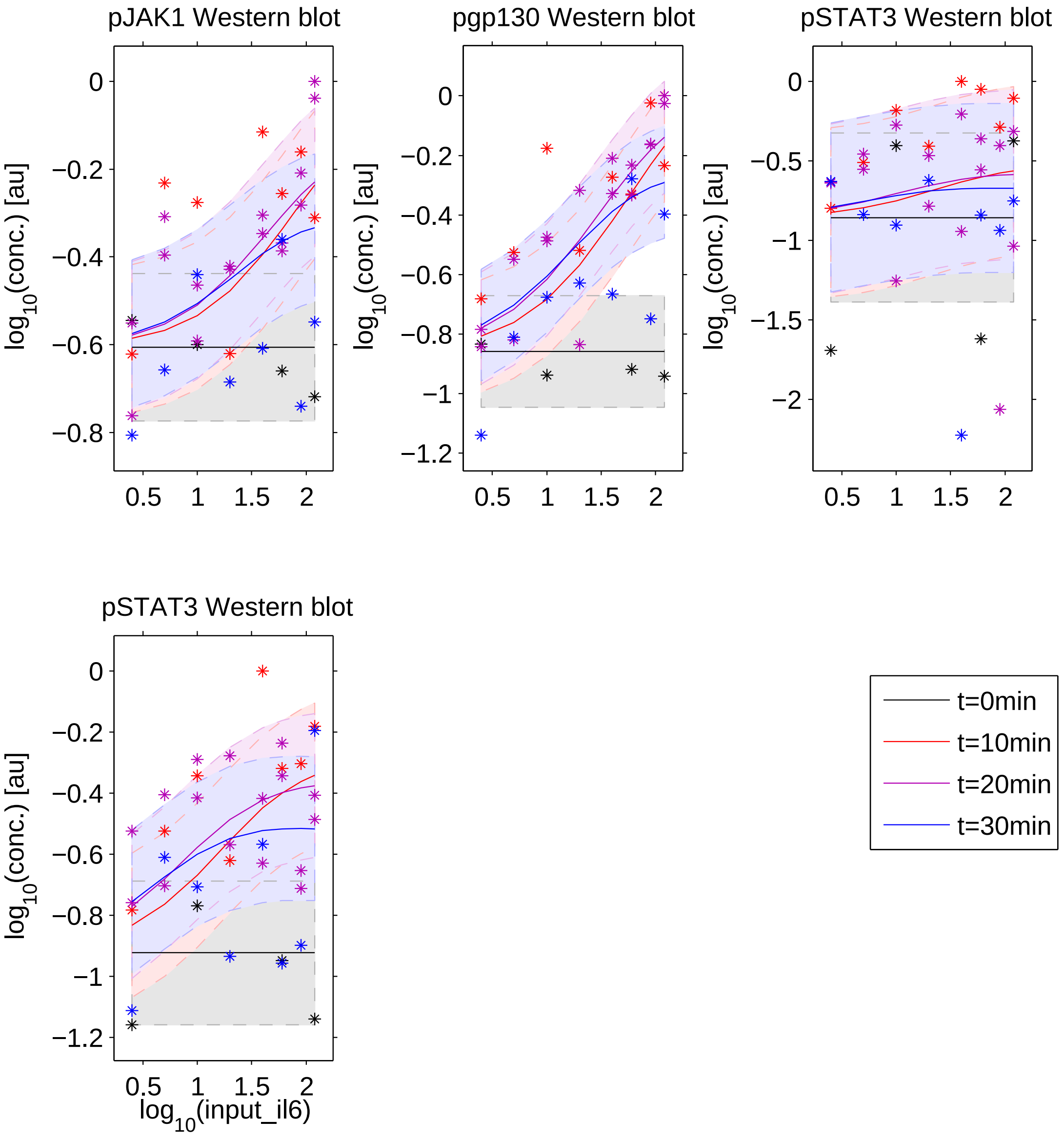

Figure S42: bohl_hep_2007_04_04_DoseResponse_3TP observables and experimental data for the experiment.

4.16.3 Condition dependent parameter changes

The following model parameters were changed to simulate these experimental conditions:

Parameter	Condition values							
input_il6	10	120	2.5	20	40	5	60	90

Table S25: Model parameters modified for experiment bohl hep 2007 04 04 DoseResponse 3TP. Different columns indicate different conditions.

4.17 Experiment: bohl hep 2007 07 17 DRTC

4.17.1 Comments

Experimenter: Sebastian Bohl
Date: 2007-07-17
Cells: primary mouse hepatocytes
Treatment: IL-6 5-10-20-40-80ng/ml constant

4.17.2 Model fit and plots

The model observables and the experimental data is shown in Figure S43. The necessary parameter transformation is listed in Table S26.

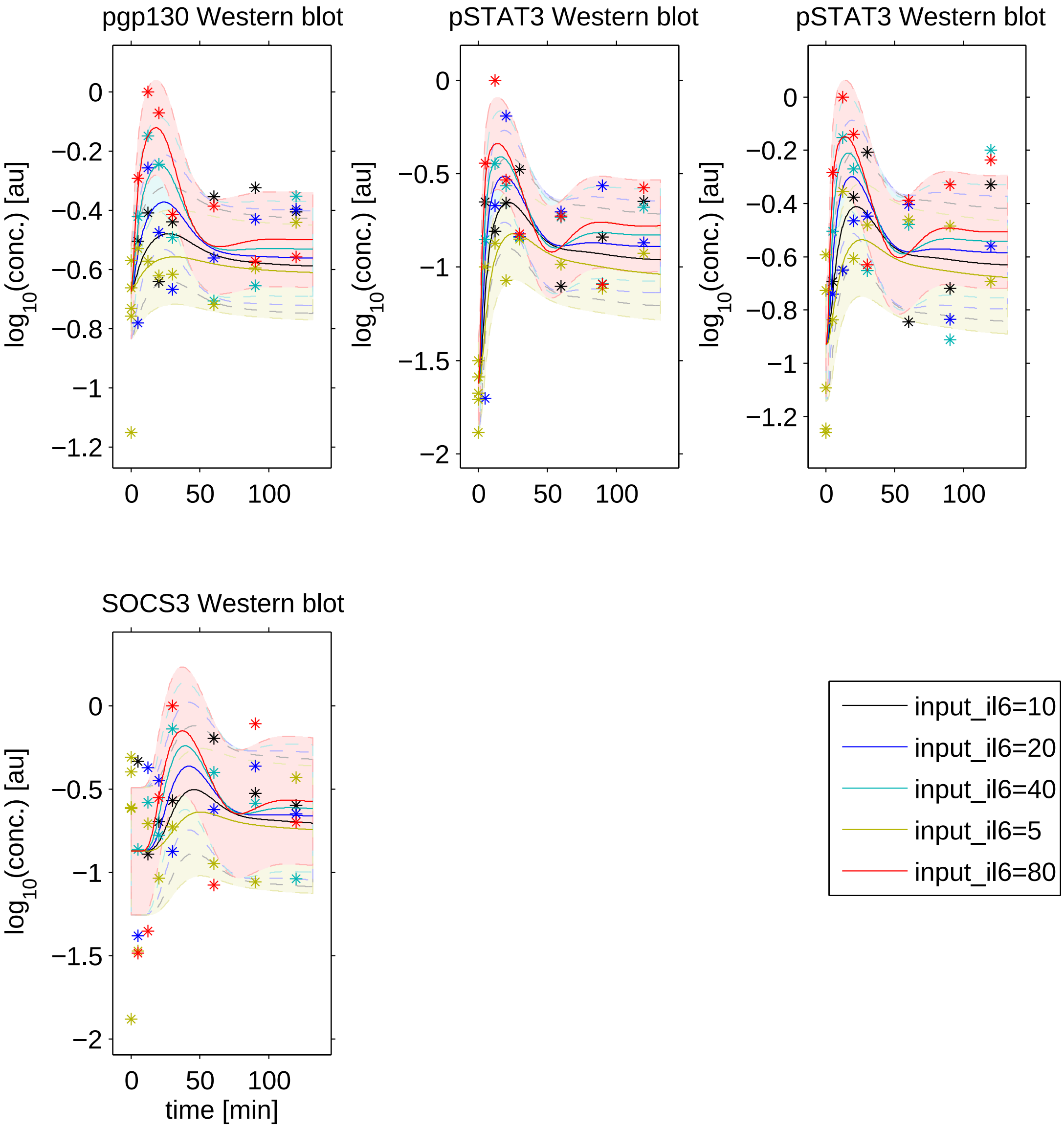

Figure S43: bohl_hep_2007_07_17_DRTC observables and experimental data for the experiment.

4.17.3 Condition dependent parameter changes

The following model parameters were changed to simulate these experimental conditions:

Parameter	Condition values				
input_il6	10	20	40	5	80

Table S26: Model parameters modified for experiment bohl hep 2007 07 17 DRTC. Different columns indicate different conditions.

4.18 Experiment: bohl hep 2009 06 23 DRTC

4.18.1 Comments

Experimenter: Sebastian Bohl
Date: 2009-06-23
Cells: primary mouse hepatocytes
Treatment: IL-6 0.25-0.5-1-2 ng/ml constant

4.18.2 Model fit and plots

The model observables and the experimental data is shown in Figure S44. The necessary parameter transformation is listed in Table S27.

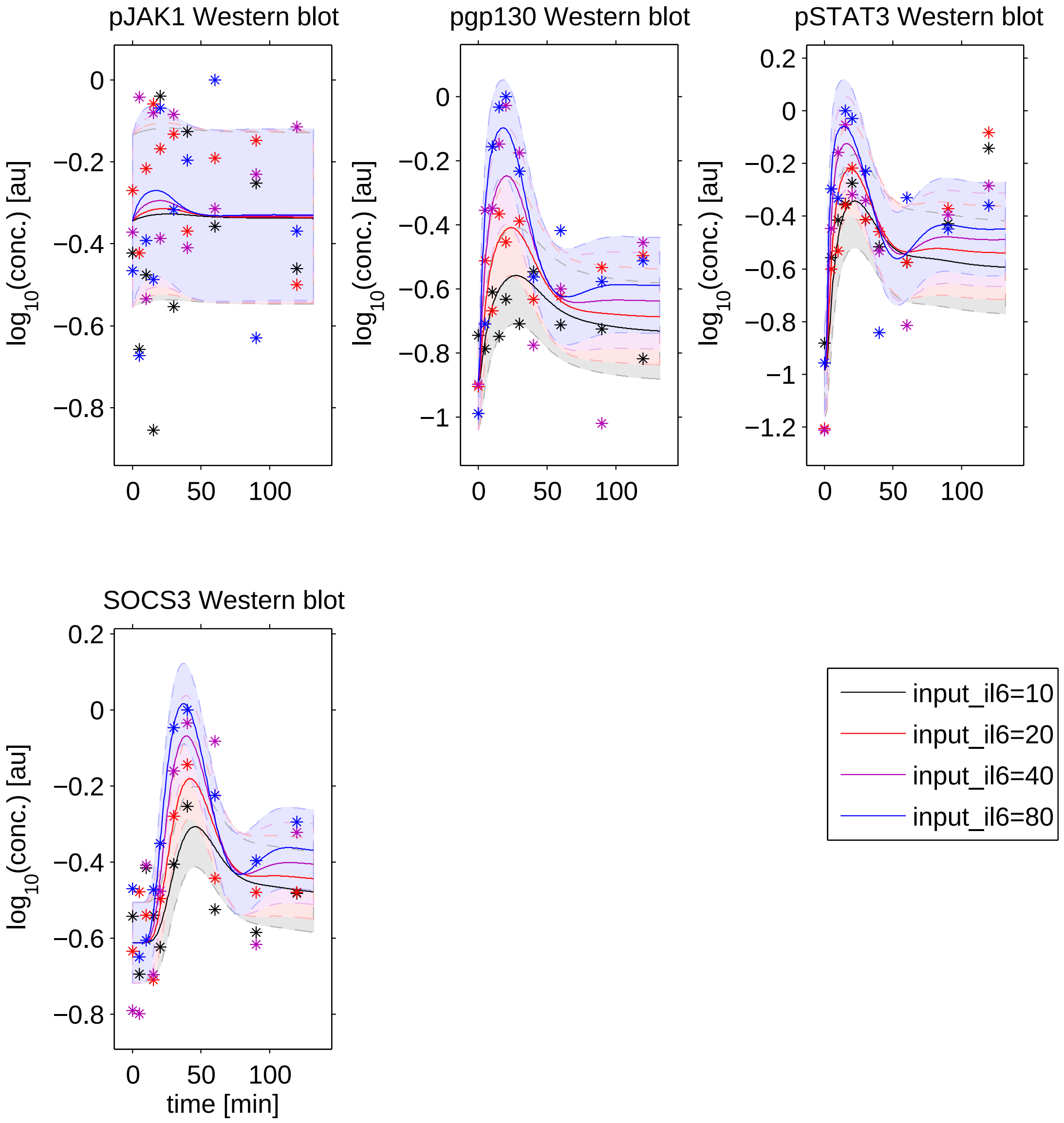

Figure S44: bohl_hep_2009_06_23_DRTC observables and experimental data for the experiment.

4.18.3 Condition dependent parameter changes

The following model parameters were changed to simulate these experimental conditions:

**Parameter**	**Condition values**			
input_il6	10	20	40	80

Table S27: Model parameters modified for experiment bohl hep 2009 06 23 DRTC. Different columns indicate different conditions.

4.19 Experiment: bohl hep 2009 07 07 DRTC

4.19.1 Comments

Experimenter: Sebastian Bohl
Date: 2009-07-07
Cells: primary mouse hepatocytes
Treatment: IL-6 0.25-0.5-1-2 ng/ml constant

4.19.2 Model fit and plots

The model observables and the experimental data is shown in Figure S45. The necessary parameter transformation is listed in Table S28.

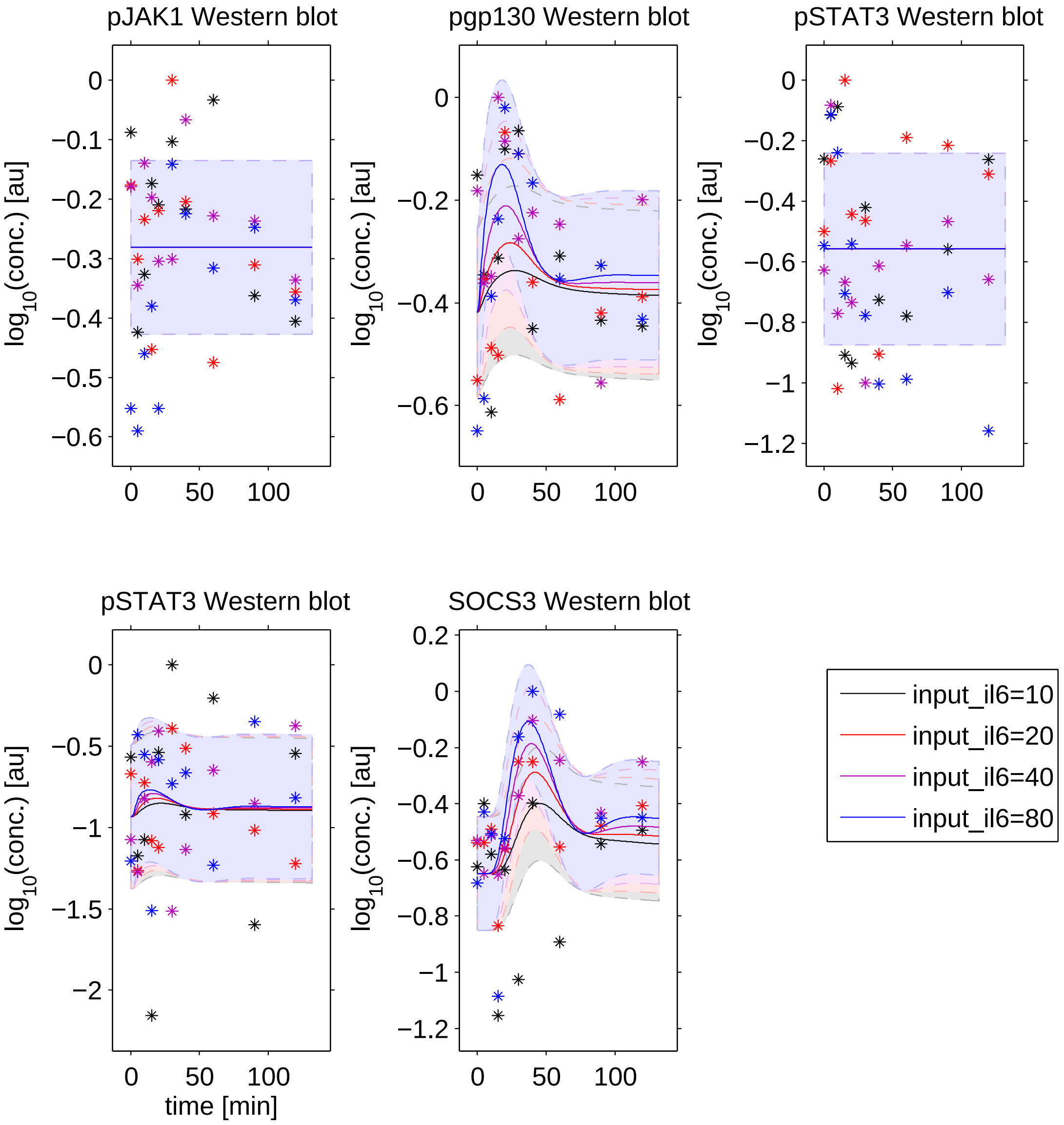

Figure S45: bohl_hep_2009_07_07_DRTC observables and experimental data for the experiment.

4.19.3 Condition dependent parameter changes

The following model parameters were changed to simulate these experimental conditions:

Parameter	Condition values			
input_il6	10	20	40	80

Table S28: Model parameters modified for experiment bohl hep 2009 07 07 DRTC. Different columns indicate different conditions.

4.20 Experiment: braun hep 2013 11 04 Stattic Ruxolitinib Inhibitor

4.20.1 Comments

Experimenter: Svantje Sobotta
Date: 2013-11-04
Cells: primary mouse hepatocytes
Treatment: IL-6 dose response 0-500 40 ng/ml = 1 nmol/l, Ruxolitinib 500 nmol/l, Stattic 1 µmol/l

4.20.2 Model fit and plots

The model observables and the experimental data is shown in Figure S46. The 3 necessary parameter transformations are listed in Table S29. Note that transformations with only one entry are the same for all experimental conditions corresponding to this experiment.

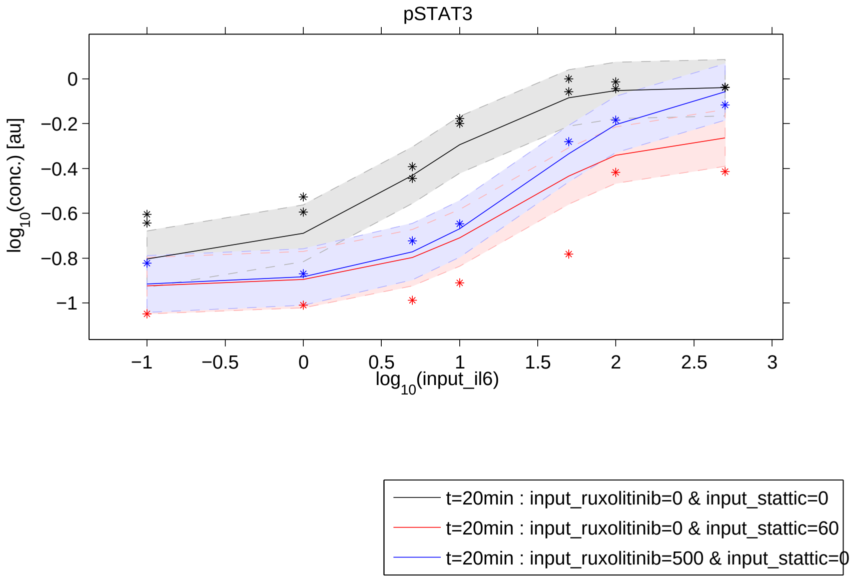

Figure S46: braun_hep_2013_11_04_Stattic_Ruxolitinib_Inhibitor observables and experimental data for the experiment.

4.20.3 Condition dependent parameter changes

The following model parameters were changed to simulate these experimental conditions:

Parameter	Condition values																				
input_il6	0.1	0.1	0.1	1	1	1	10	10	10	100	100	100	5	5	5	50	50	50	500	500	500
input_ruxolitinib	0	0	500	0	0	500	0	0	500	0	0	500	0	0	500	0	0	500	0	0	500
input_stattic	0	60	0	0	60	0	0	60	0	0	60	0	0	60	0	0	60	0	0	60	0

Table S29: Model parameters modified for experiment braun hep 2013 11 04 Stattic Ruxolitinib Inhibitor. Different columns indicate different conditions.

4.21 Experiment: braun hep 2013 11 12 Stattic Ruxolitinib Inhibitor

4.21.1 Comments

Experimenter: Svantje Sobotta
Date: 2013-11-04
Cells: primary mouse hepatocytes Treatment: IL-6 dose response 0-500 40 ng/ml = 1 nmol/l, Ruxolitinib 500 nmol/l

4.21.2 Model fit and plots

The model observables and the experimental data is shown in Figure S47. The 2 necessary parameter transformations are listed in Table S30. Note that transformations with only one entry are the same for all experimental conditions corresponding to this experiment.

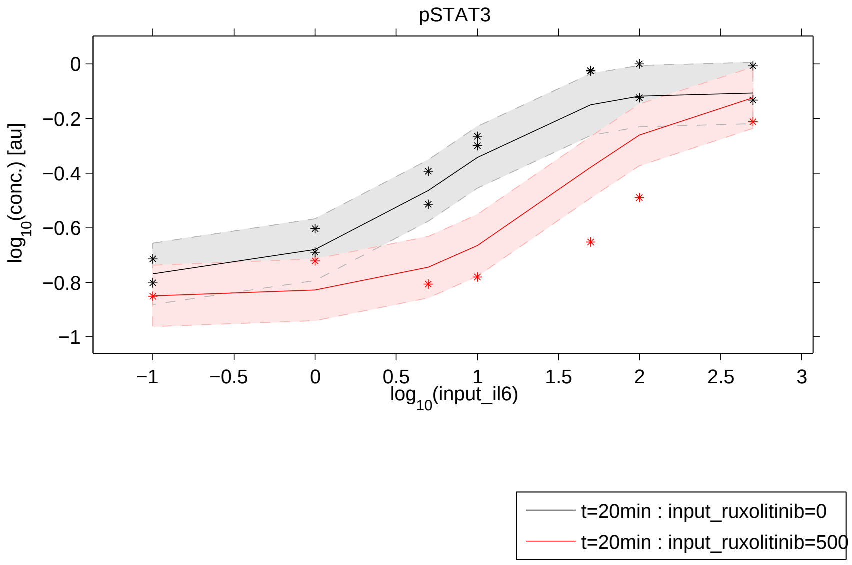

Figure S47: braun_hep_2013_11_12_Stattic_Ruxolitinib_Inhibitor observables and experimental data for the experiment.

4.21.3 Condition dependent parameter changes

The following model parameters were changed to simulate these experimental conditions:

Parameter	Condition values													
input_il6	0.1	0.1	1	1	10	10	100	100	5	5	50	50	500	500
input_ruxolitinib	0	500	0	500	0	500	0	500	0	500	0	500	0	500

Table S30: Model parameters modified for experiment braun hep 2013 11 12 Stattic Ruxolitinib Inhibitor. Different columns indicate different conditions.

4.22 Experiment: braun app hep 2014 04 28 IL6DR Rux 1h SOCS3

4.22.1 Model fit and plots

The model observables and the experimental data is shown in Figure S48. The 2 necessary parameter transformations are listed in Table S31. Note that transformations with only one entry are the same for all experimental conditions corresponding to this experiment.

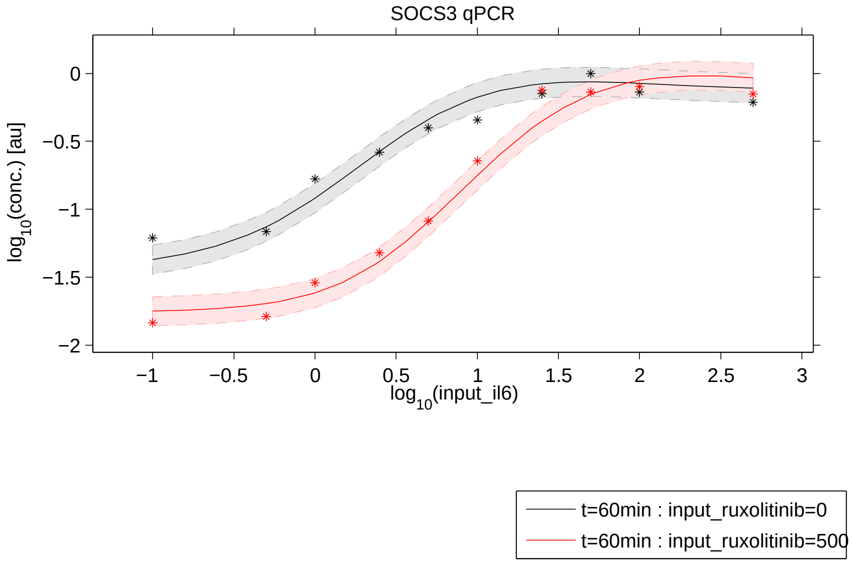

Figure S48: braun_app_hep_2014_04_28_IL6DR_Rux_1h_SOCS3 observables and experimental data for the experiment.

4.22.2 Condition dependent parameter changes

The following model parameters were changed to simulate these experimental conditions:

Parameter	Condition values																			
input_il6	0.1	0.1	0.5	0.5	1	1	10	10	100	100	2.5	2.5	25	25	5	5	50	50	500	500
input_ruxolitinib	0	500	0	500	0	500	0	500	0	500	0	500	0	500	0	500	0	500	0	500

Table S31: Model parameters modified for experiment braun app hep 2014 04 28 IL6DR Rux 1h SOCS3. Different columns indicate different conditions.

4.23 Experiment: braun app hep 2011 06 06 qPCR 140109 IL6DR 1h

4.23.1 Model fit and plots

The model observables and the experimental data is shown in Figure S49. The necessary parameter transformation is listed in Table S32.

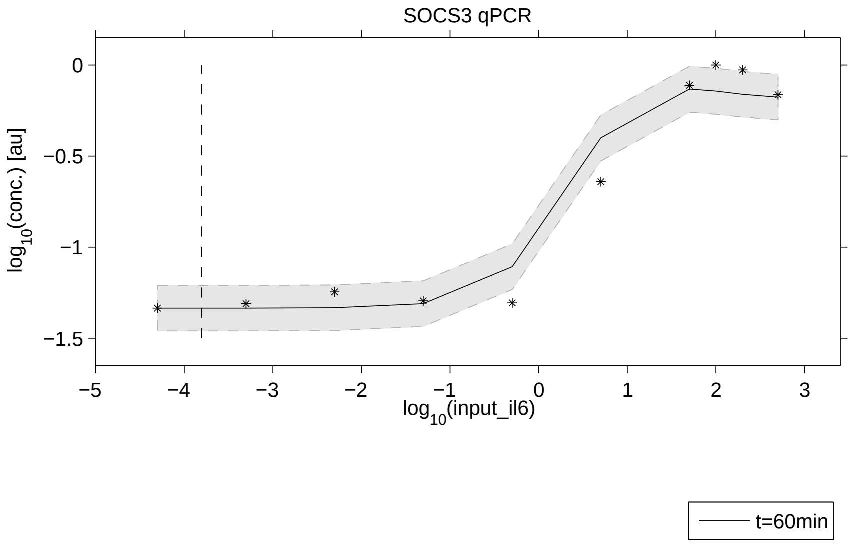

Figure S49: braun_app_hep_2011_06_06_qPCR_140109_IL6DR_1h observables and experimental data for the experiment.

4.23.2 Condition dependent parameter changes

The following model parameters were changed to simulate these experimental conditions:

Parameter	Condition values									
input_il6	0	0.0005	0.005	0.05	0.5	100	200	5	50	500

Table S32: Model parameters modified for experiment braun app hep 2011 06 06 qPCR 140109 IL6DR 1h. Different columns indicate different conditions.

4.24 Experiment: braun app hep 2013 10 14 qPCR 140109 IL6DR 1h

4.24.1 Model fit and plots

The model observables and the experimental data is shown in Figure S50. The necessary parameter transformation is listed in Table S33.

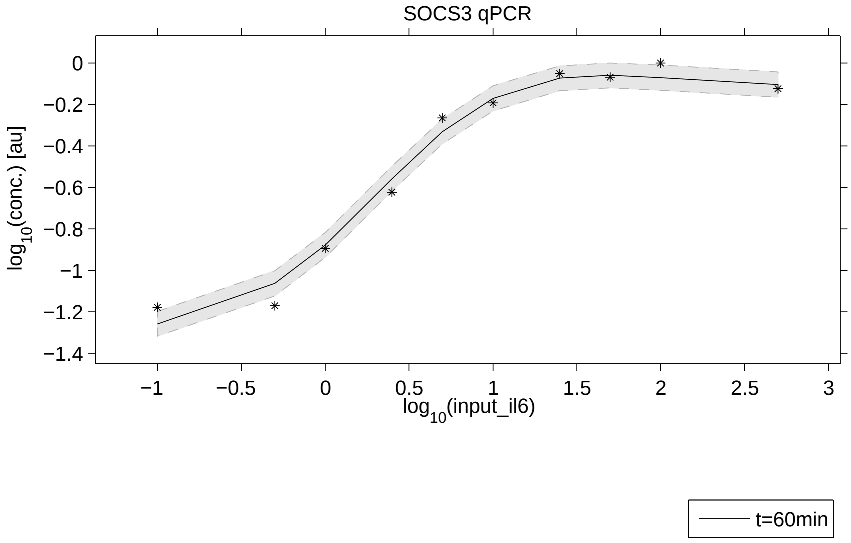

Figure S50: braun_app_hep_2013_10_14_qPCR_140109_IL6DR_1h observables and experimental data for the experiment.

4.24.2 Condition dependent parameter changes

The following model parameters were changed to simulate these experimental conditions:

Parameter	Condition values									
input_il6	0.1	0.5	1	10	100	2.5	25	5	50	500

Table S33: Model parameters modified for experiment braun app hep 2013 10 14 qPCR 140109 IL6DR 1h. Different columns indicate different conditions.

4.25 Experiment: braun app hep 2013 10 21 qPCR 140109 IL6DR 1h

4.25.1 Model fit and plots

The model observables and the experimental data is shown in Figure S51. The necessary parameter transformation is listed in Table S34.

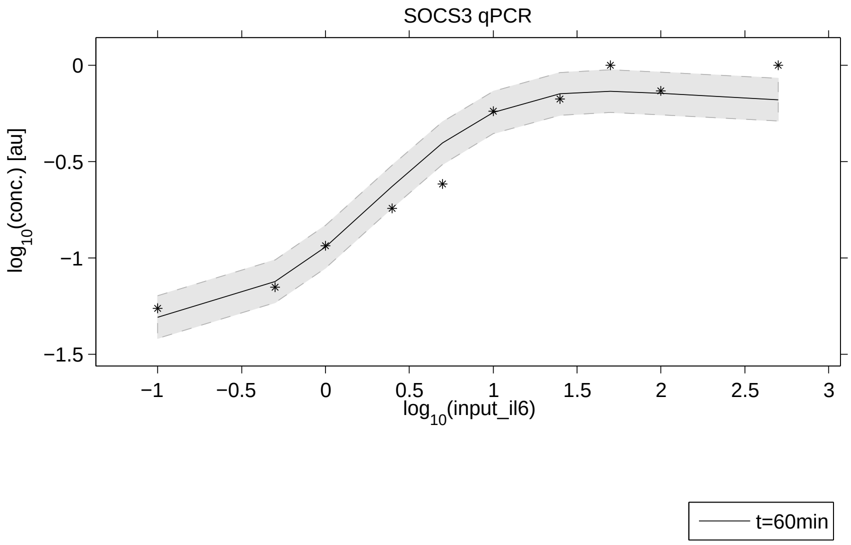

Figure S51: braun_app_hep_2013_10_21_qPCR_140109_IL6DR_1h observables and experimental data for the experiment.

4.25.2 Condition dependent parameter changes

The following model parameters were changed to simulate these experimental conditions:

Parameter	Condition values									
input_il6	0.1	0.5	1	10	100	2.5	25	5	50	500

Table S34: Model parameters modified for experiment braun app hep 2013 10 21 qPCR 140109 IL6DR 1h. Different columns indicate different conditions.

4.26 Experiment: braun app hep 2012 02 14 qPCR 140224 IL6DR Inh 1h Socs3

4.26.1 Model fit and plots

The model observables and the experimental data is shown in Figure S52. The 2 necessary parameter transformations are listed in Table S35. Note that transformations with only one entry are the same for all experimental conditions corresponding to this experiment.

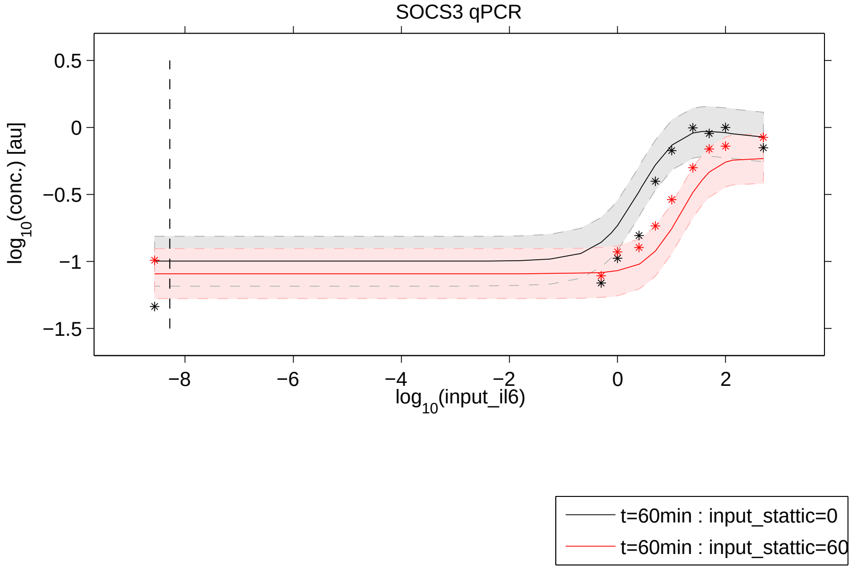

Figure S52: braun_app_hep_2012_02_14_qPCR_140224_IL6DR_Inh_1h_Socs3 observables and experimental data for the experiment.

4.26.2 Condition dependent parameter changes

The following model parameters were changed to simulate these experimental conditions:

Parameter	Condition values																			
input_il6	0	0	0.5	0.5	1	1	10	10	100	100	2.5	2.5	25	25	5	5	50	50	500	500
input_stattic	0	60	0	60	0	60	0	60	0	60	0	60	0	60	0	60	0	60	0	60

Table S35: Model parameters modified for experiment braun app hep 2012 02 14 qPCR 140224 IL6DR Inh 1h Socs3. Different columns indicate different conditions.

4.27 Experiment: braun app hep 2012 04 10 qPCR 140224 IL6DR Inh 1h Socs3

4.27.1 Model fit and plots

The model observables and the experimental data is shown in Figure S53. The 2 necessary parameter transformations are listed in Table S36. Note that transformations with only one entry are the same for all experimental conditions corresponding to this experiment.

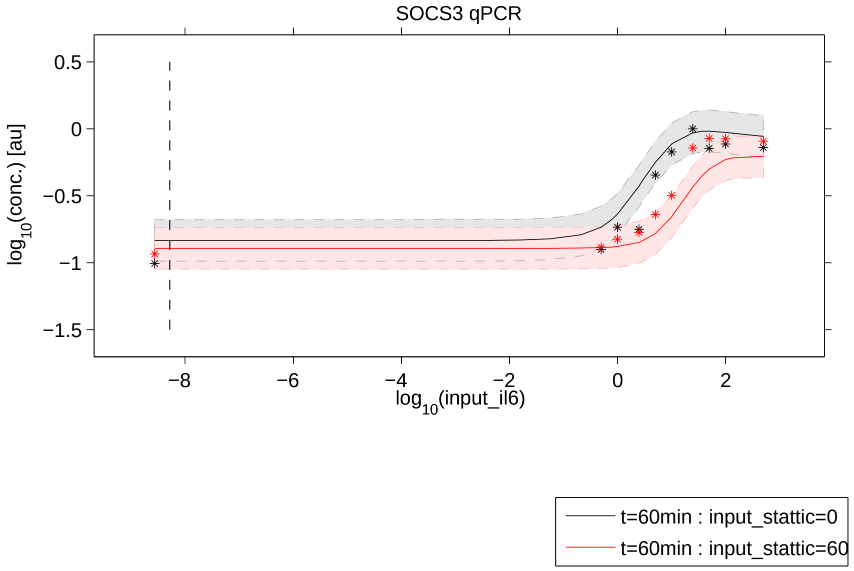

Figure S53: braun_app_hep_2012_04_10_qPCR_140224_IL6DR_Inh_1h_Socs3 observables and experimental data for the experiment.

4.27.2 Condition dependent parameter changes

The following model parameters were changed to simulate these experimental conditions:

Parameter	Condition values																			
input_il6	0	0	0.5	0.5	1	1	10	10	100	100	2.5	2.5	25	25	5	5	50	50	500	500
input_stattic	0	60	0	60	0	60	0	60	0	60	0	60	0	60	0	60	0	60	0	60

Table S36: Model parameters modified for experiment braun app hep 2012 04 10 qPCR 140224 IL6DR Inh 1h Socs3. Different columns indicate different conditions.

4.28 Experiment: braun app hep 2013 10 14 qPCR 140224 IL6DR Inh 1h Socs3

4.28.1 Model fit and plots

The model observables and the experimental data is shown in Figure S54. The 2 necessary parameter transformations are listed in Table S37. Note that transformations with only one entry are the same for all experimental conditions corresponding to this experiment.

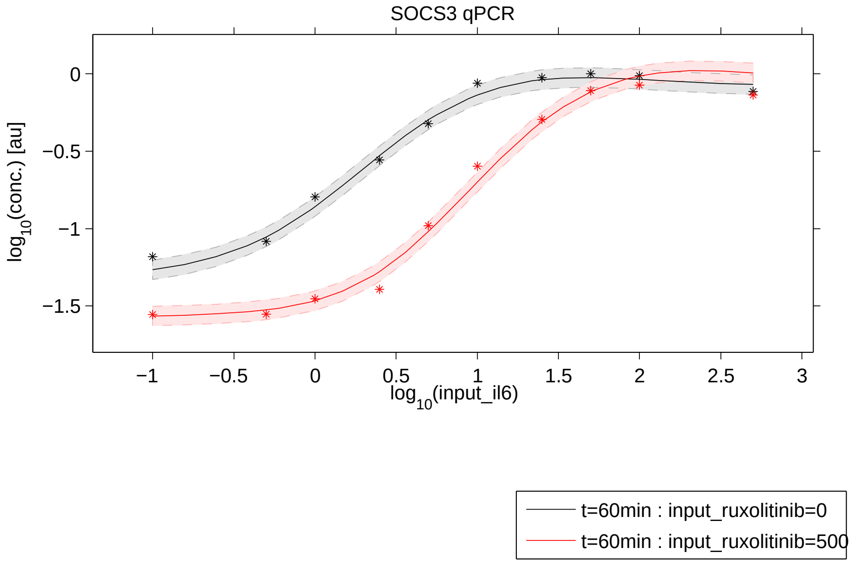

Figure S54: braun_app_hep_2013_10_14_qPCR_140224_IL6DR_Inh_1h_Socs3 observables and experimental data for the experiment.

4.28.2 Condition dependent parameter changes

The following model parameters were changed to simulate these experimental conditions:

Parameter	Condition values																			
input_il6	0.1	0.1	0.5	0.5	1	1	10	10	100	100	2.5	2.5	25	25	5	5	50	50	500	500
input_ruxolitinib	0	500	0	500	0	500	0	500	0	500	0	500	0	500	0	500	0	500	0	500

Table S37: Model parameters modified for experiment braun app hep 2013 10 14 qPCR 140224 IL6DR Inh 1h Socs3. Different columns indicate different conditions.

4.29 Experiment: braun app hep 2014 04 22 qPCR 140526 IL6DR Rux 6h SOCS3

4.29.1 Model fit and plots

The model observables and the experimental data is shown in Figure S55. The 2 necessary parameter transformations are listed in Table S38. Note that transformations with only one entry are the same for all experimental conditions corresponding to this experiment.

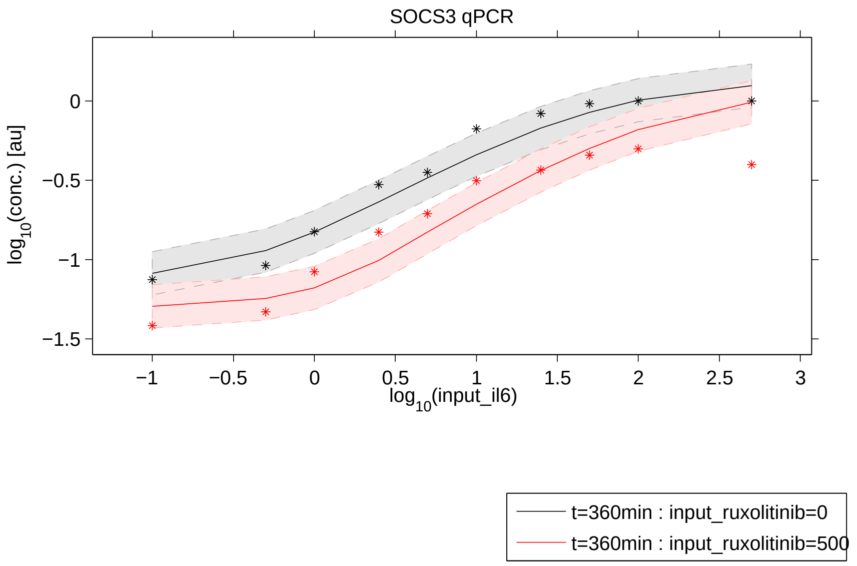

Figure S55: braun_app_hep_2014_04_22_qPCR_140526_IL6DR_Rux_6h_SOCS3 observables and experimental data for the experiment.

4.29.2 Condition dependent parameter changes

The following model parameters were changed to simulate these experimental conditions:

Parameter	Condition values																			
input_il6	0.1	0.1	0.5	0.5	1	1	10	10	100	100	2.5	2.5	25	25	5	5	50	50	500	500
input_ruxolitinib	0	500	0	500	0	500	0	500	0	500	0	500	0	500	0	500	0	500	0	500

Table S38: Model parameters modified for experiment braun app hep 2014 04 22 qPCR 140526 IL6DR Rux 6h SOCS3. Different columns indicate different conditions.

4.30 Experiment: braun app hep 2014 05 19 qPCR 140526 IL6DR Rux 6h SOCS3

4.30.1 Model fit and plots

The model observables and the experimental data is shown in Figure S56. The 2 necessary parameter transformations are listed in Table S39. Note that transformations with only one entry are the same for all experimental conditions corresponding to this experiment.

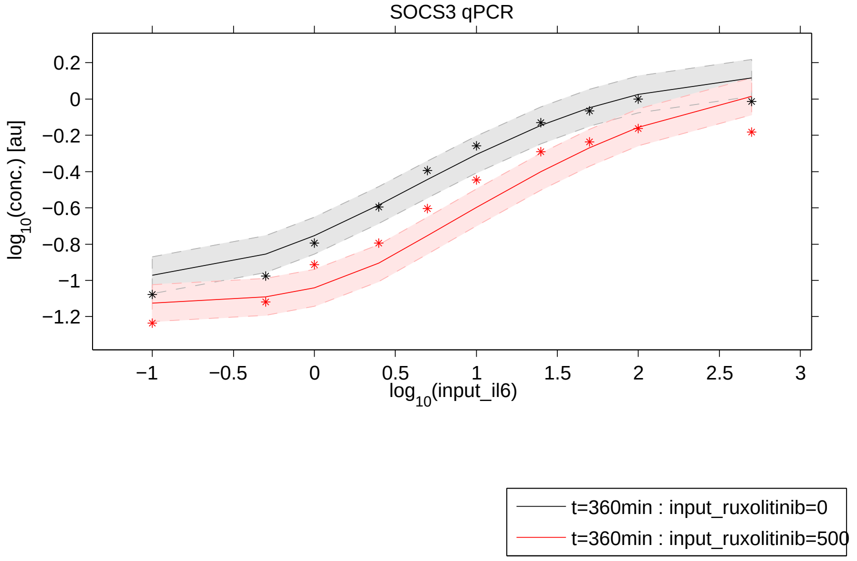

Figure S56: braun_app_hep_2014_05_19_qPCR_140526_IL6DR_Rux_6h_SOCS3 observables and experimental data for the experiment.

4.30.2 Condition dependent parameter changes

The following model parameters were changed to simulate these experimental conditions:

Parameter	Condition values																			
input_il6	0.1	0.1	0.5	0.5	1	1	10	10	100	100	2.5	2.5	25	25	5	5	50	50	500	500
input_ruxolitinib	0	500	0	500	0	500	0	500	0	500	0	500	0	500	0	500	0	500	0	500

Table S39: Model parameters modified for experiment braun app hep 2014 05 19 qPCR 140526 IL6DR Rux 6h SOCS3. Different columns indicate different conditions.

4.31 Experiment: braun app hep 2014 04 22 qPCR 140604 IL6DR Rux 24h SOCS3

4.31.1 Model fit and plots

The model observables and the experimental data is shown in Figure S57. The 2 necessary parameter transformations are listed in Table S40. Note that transformations with only one entry are the same for all experimental conditions corresponding to this experiment.

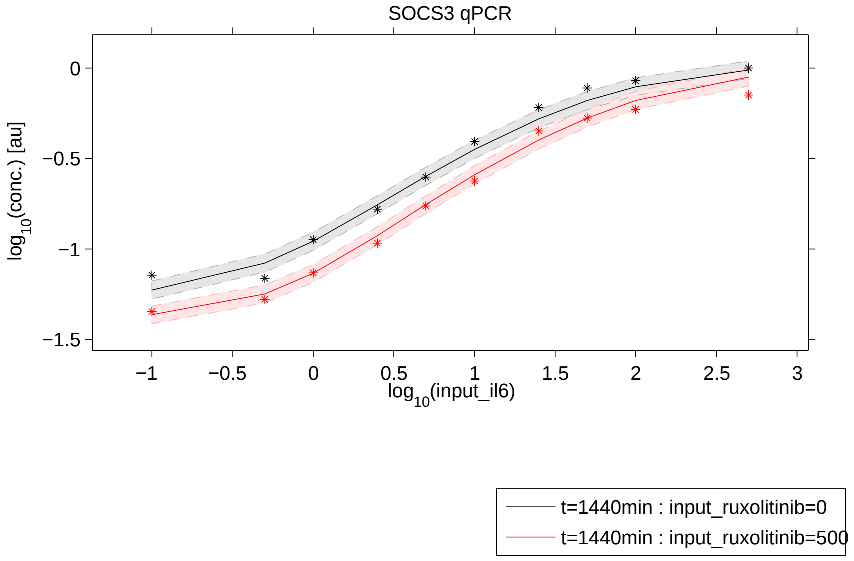

Figure S57: braun_app_hep_2014_04_22_qPCR_140604_IL6DR_Rux_24h_SOCS3 observables and experimental data for the experiment.

4.31.2 Condition dependent parameter changes

The following model parameters were changed to simulate these experimental conditions:

Parameter	Condition values																			
input_il6	0.1	0.1	0.5	0.5	1	1	10	10	100	100	2.5	2.5	25	25	5	5	50	50	500	500
input_ruxolitinib	0	500	0	500	0	500	0	500	0	500	0	500	0	500	0	500	0	500	0	500

Table S40: Model parameters modified for experiment braun app hep 2014 04 22 qPCR 140604 IL6DR Rux 24h SOCS3. Different columns indicate different conditions.

4.32 Experiment: braun app hep 2014 05 19 qPCR 140604 IL6DR Rux 24h SOCS3

4.32.1 Model fit and plots

The model observables and the experimental data is shown in Figure S58. The 2 necessary parameter transformations are listed in Table S41. Note that transformations with only one entry are the same for all experimental conditions corresponding to this experiment.

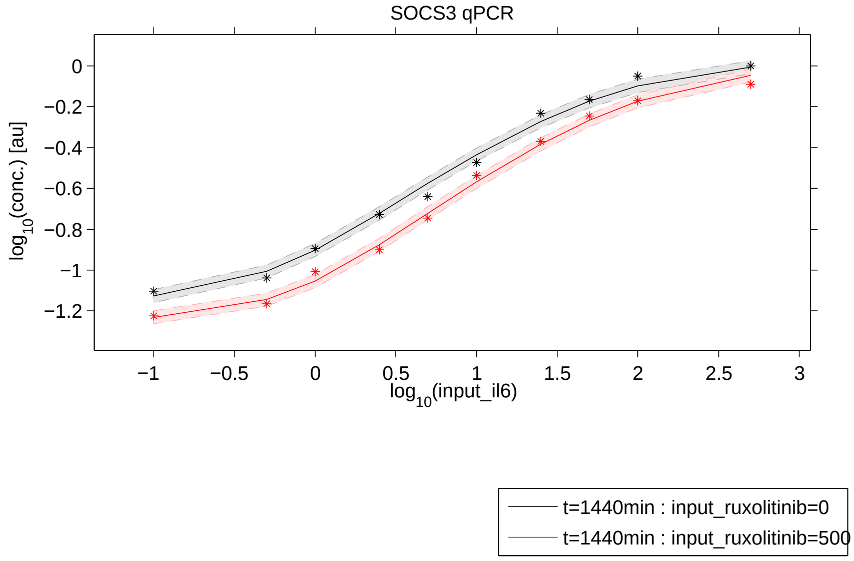

Figure S58: braun_app_hep_2014_05_19_qPCR_140604_IL6DR_Rux_24h_SOCS3 observables and experimental data for the experiment.

4.32.2 Condition dependent parameter changes

The following model parameters were changed to simulate these experimental conditions:

Parameter	Condition values																			
input_il6	0.1	0.1	0.5	0.5	1	1	10	10	100	100	2.5	2.5	25	25	5	5	50	50	500	500
input_ruxolitinib	0	500	0	500	0	500	0	500	0	500	0	500	0	500	0	500	0	500	0	500

Table S41: Model parameters modified for experiment braun app hep 2014 05 19 qPCR 140604 IL6DR Rux 24h SOCS3. Different columns indicate different conditions.

4.33 Experiment: braun hep 2011 09 06 DRTC

4.33.1 Comments

Experimenter: Svantje Sobotta
Date: 2011-09-06
Cells: primary mouse hepatocytes
Treatment: IL-6 0.5-5-50-500 ng/ml DRTC

4.33.2 Model fit and plots

The model observables and the experimental data is shown in Figure S59. The necessary parameter transformation is listed in Table S42.

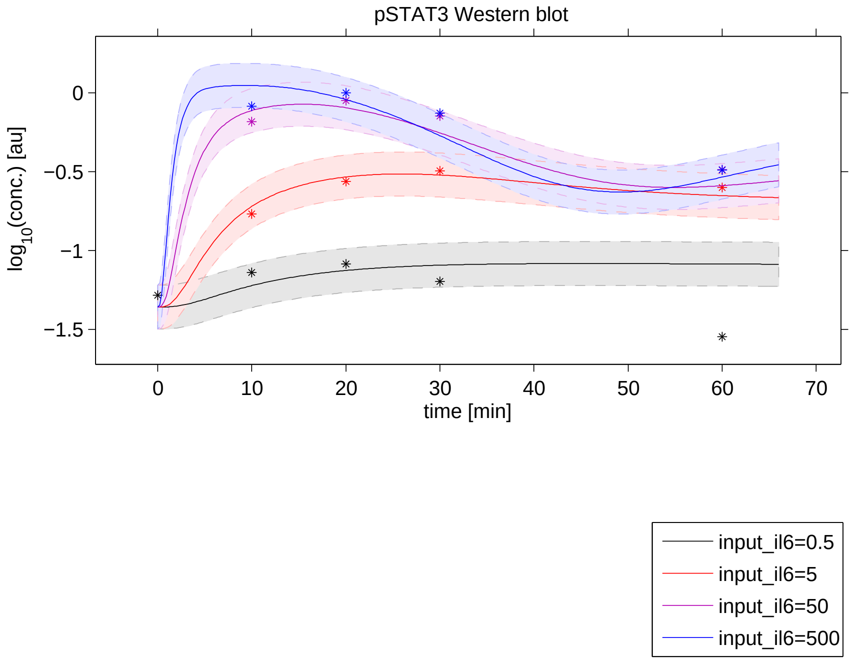

Figure S59: braun_hep_2011_09_06_DRTC observables and experimental data for the experiment.

4.33.3 Condition dependent parameter changes

The following model parameters were changed to simulate these experimental conditions:

Parameter	Condition values			
input_il6	0.5	5	50	500

Table S42: Model parameters modified for experiment braun hep 2011 09 06 DRTC. Different columns indicate different conditions.

4.34 Experiment: braun hep 2011 04 04 STAT3 pY degree replicates

4.34.1 Comments

Experimenter: Svantje Sobotta, Martin Boehm
Date: 2013-08-14
Cells: primary mouse hepatocytes
Treatment: IL-6 dose 40 ng/ml
Technique: mass spectrometry

4.34.2 Model fit and plots

The model observables and the experimental data is shown in Figure S60. The necessary parameter transformation is listed in Table S44.

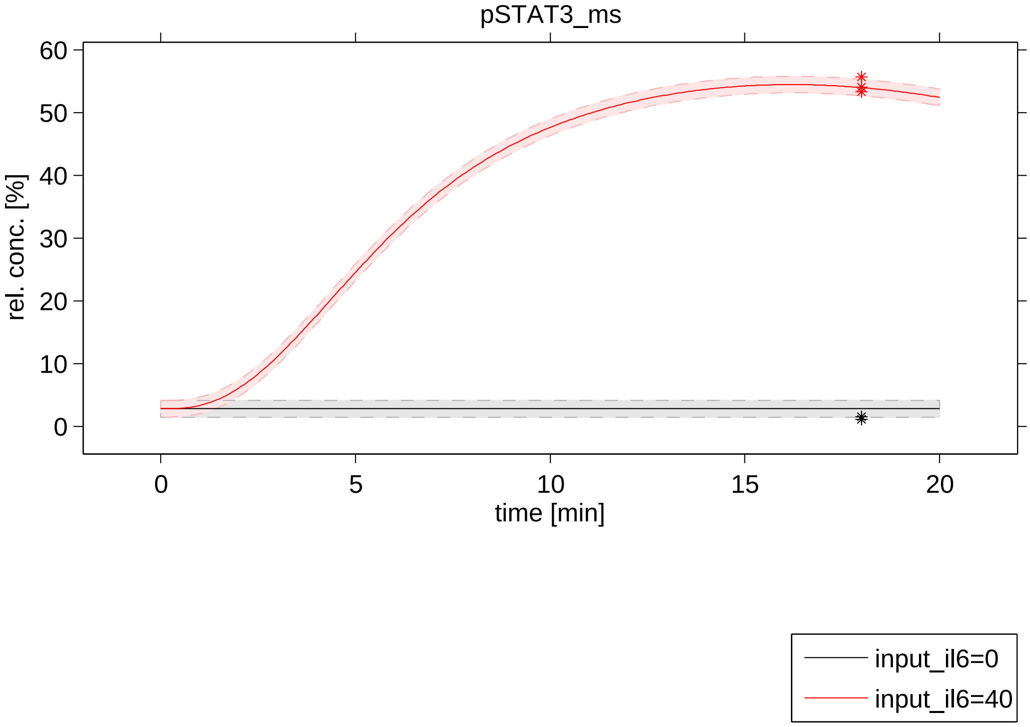

Figure S60: braun_hep_2011_04_04_STAT3_pY_degree_replicates observables and experimental data for the experiment.

4.34.3 Observables

The following observables are added in this data set:

Observable		Equations
pSTAT3_ms [%]	y	$100 \cdot [\text{pSTAT3rel}]$
	σ	sd_pSTAT3_ms

Table S43: Observables added for experiment braun_hep_2011_04_04_STAT3_pY_degree_replicates

4.34.4 Condition dependent parameter changes

The following model parameters were changed to simulate these experimental conditions:

Parameter	Condition values	
input_il6	0	40

Table S44: Model parameters modified for experiment braun hep 2011 04 04 STAT3 pY degree replicates. Different columns indicate different conditions.

4.35 Experiment: braun hep 2013 07 08 qPCR Socs3 Stat3

4.35.1 Comments

Experimenter: Svantje Sobotta
Date: 2013-07-08
Cells: primary mouse hepatocytes
Treatment: IL-6 dose response 40 ng/ml = 1 nmol/l
values normalized by HPRT

4.35.2 Model fit and plots

The model observables and the experimental data is shown in Figure S61. The necessary parameter transformation is listed in Table S45.

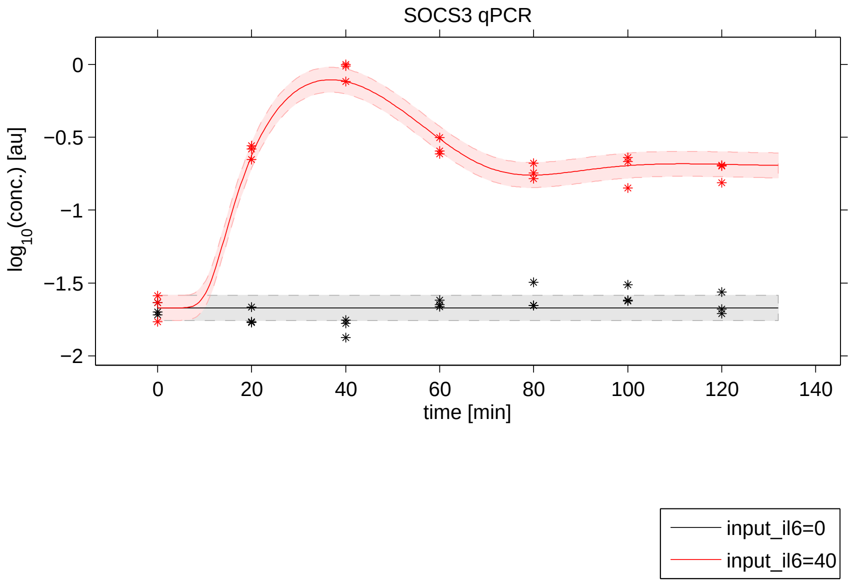

Figure S61: braun_hep_2013_07_08_qPCR_Socs3_Stat3 observables and experimental data for the experiment.

4.35.3 Condition dependent parameter changes

The following model parameters were changed to simulate these experimental conditions:

Parameter	Condition values	
input_il6	0	40

Table S45: Model parameters modified for experiment braun hep 2013 07 08 qPCR Socs3 Stat3. Different columns indicate different conditions.

4.36 Experiment: braun hep 2011 12 12 Stattic Stat3 Inhibitor DR

4.36.1 Comments

Experimenter: Svantje Sobotta
Date: 2011-12-12
Cells: primary mouse hepatocytes
Treatment: input_stattic dose response 0 to 60 µmol/l, IL-6 40 ng/ml

4.36.2 Model fit and plots

The model observables and the experimental data is shown in Figure S62. The necessary parameter transformation is listed in Table S46.

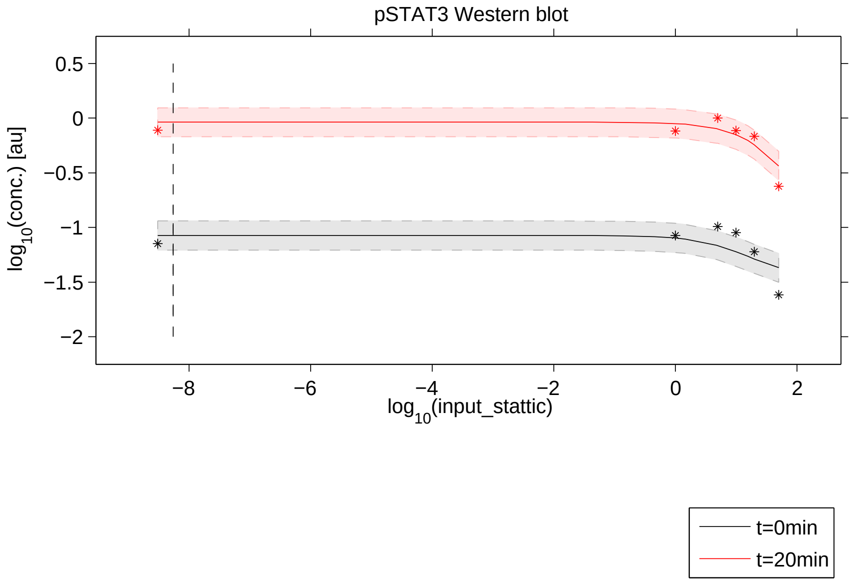

Figure S62: braun_hep_2011_12_12_Stattic_Stat3_Inhibitor_DR observables and experimental data for the experiment.

4.36.3 Condition dependent parameter changes

The following model parameters were changed to simulate these experimental conditions:

Parameter	Condition values					
input_stattic	0	1	10	20	5	50

Table S46: Model parameters modified for experiment braun hep 2011 12 12 Stattic Stat3 Inhibitor DR. Different columns indicate different conditions.

4.37 Experiment: braun hep 2012 01 10 Stattic Stat3 Inhibitor DR

4.37.1 Comments

Experimenter: Svantje Sobotta
Date: 2012-01-10
Cells: primary mouse hepatocytes
Treatment: input_stattic dose response 0 to 60 µmol/l, IL-6 40 ng/ml

4.37.2 Model fit and plots

The model observables and the experimental data is shown in Figure S63. The necessary parameter transformation is listed in Table S47.

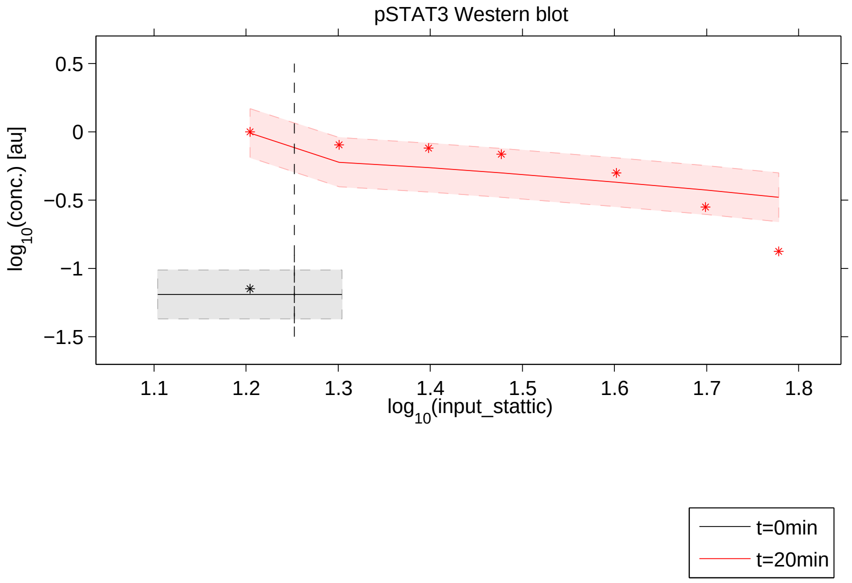

Figure S63: braun_hep_2012_01_10_Stattic_Stat3_Inhibitor_DR observables and experimental data for the experiment.

4.37.3 Condition dependent parameter changes

The following model parameters were changed to simulate these experimental conditions:

Parameter	Condition values						
input_stattic	0	20	25	30	40	50	60

Table S47: Model parameters modified for experiment braun hep 2012 01 10 Stattic Stat3 Inhibitor DR. Different columns indicate different conditions.

4.38 Experiment: braun hep 2012 01 23 Stattic Stat3 Inhibitor TC

4.38.1 Comments

Experimenter: Svantje Sobotta
Date: 2012-01-23
Cells: primary mouse hepatocytes
Treatment: IL-6 dose 50 ng/ml, Stattic 40 µmol/l

4.38.2 Model fit and plots

The model observables and the experimental data is shown in Figure S64. The 2 necessary parameter transformations are listed in Table S48. Note that transformations with only one entry are the same for all experimental conditions corresponding to this experiment.

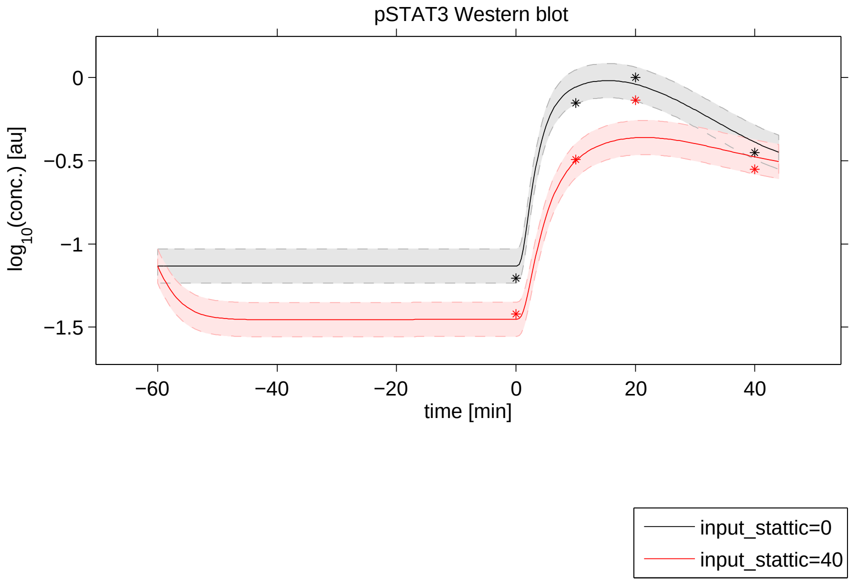

Figure S64: braun_hep_2012_01_23_Stattic_Stat3_Inhibitor_TC observables and experimental data for the experiment.

4.38.3 Condition dependent parameter changes

The following model parameters were changed to simulate these experimental conditions:

Parameter	Condition values	
input_il6	50	
input_stattic	0	40

Table S48: Model parameters modified for experiment braun hep 2012 01 23 Stattic Stat3 Inhibitor TC. Different columns indicate different conditions.

4.39 Experiment: braun hep 2012 01 23 Stattic Stat3 Inhibitor

4.39.1 Comments

Experimenter: Svantje Sobotta
Date: 2012-01-23
Cells: primary mouse hepatocytes
Treatment: IL-6 dose response 0.0005-500 40 ng/ml = 1 nmol/l, Stattic 40 µmol/l

4.39.2 Model fit and plots

The model observables and the experimental data is shown in Figure S65. The 2 necessary parameter transformations are listed in Table S49. Note that transformations with only one entry are the same for all experimental conditions corresponding to this experiment.

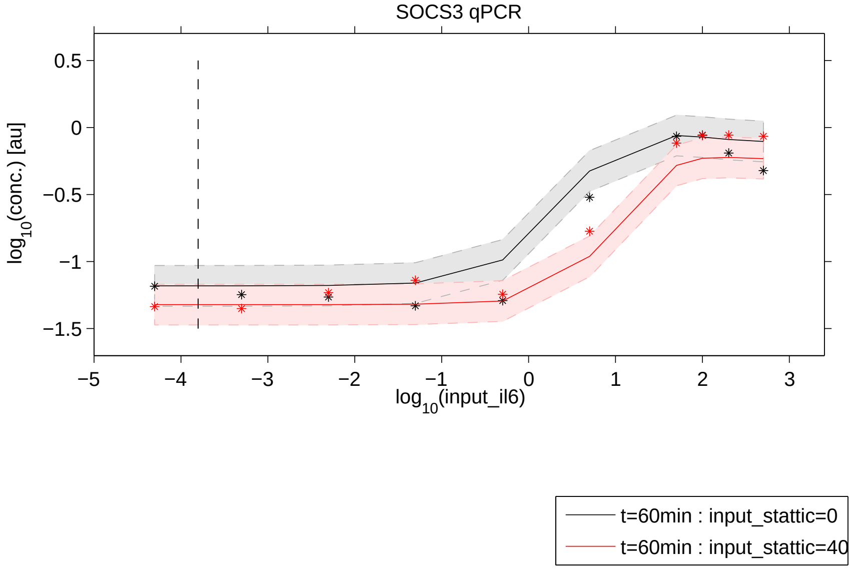

Figure S65: braun_hep_2012_01_23_Stattic_Stat3_Inhibitor observables and experimental data for the experiment.

4.39.3 Condition dependent parameter changes

The following model parameters were changed to simulate these experimental conditions:

Parameter	Condition values																			
input_il6	0	0	0.0005	0.0005	0.005	0.005	0.05	0.05	0.5	0.5	100	100	200	200	5	5	50	50	500	500
input_stattic	0	40	0	40	0	40	0	40	0	40	0	40	0	40	0	40	0	40	0	40

Table S49: Model parameters modified for experiment braun hep 2012 01 23 Stattic Stat3 Inhibitor. Different columns indicate different conditions.

4.40 Experiment: braun hep 2012 02 14 Stattic Stat3 Inhibitor TC

4.40.1 Comments

Experimenter: Svantje Sobotta
Date: 2012-02-14
Cells: primary mouse hepatocytes
Treatment: IL-6 dose 50 ng/ml, Stattic 60 µmol/l

4.40.2 Model fit and plots

The model observables and the experimental data is shown in Figure S66. The 2 necessary parameter transformations are listed in Table S50. Note that transformations with only one entry are the same for all experimental conditions corresponding to this experiment.

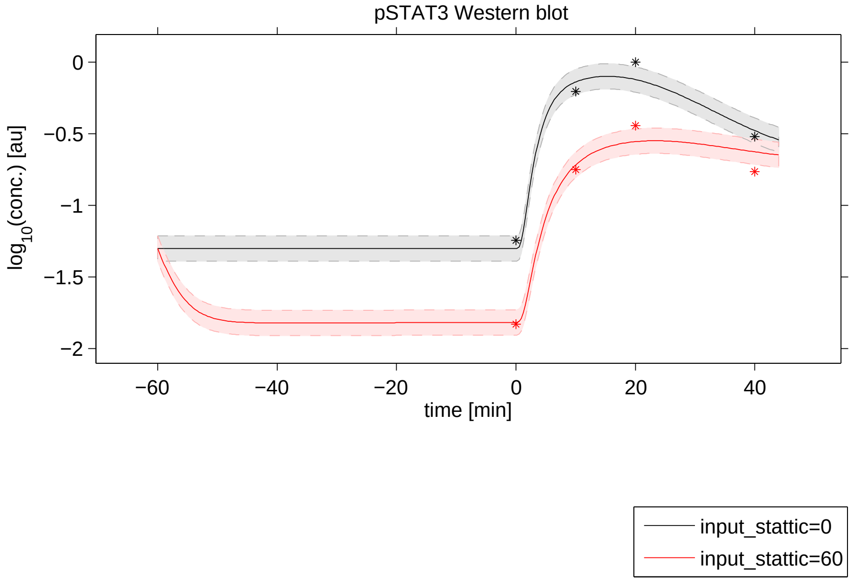

Figure S66: braun_hep_2012_02_14_Stattic_Stat3_Inhibitor_TC observables and experimental data for the experiment.

4.40.3 Condition dependent parameter changes

The following model parameters were changed to simulate these experimental conditions:

Parameter	Condition values	
input_il6	50	
input_stattic	0	60

Table S50: Model parameters modified for experiment braun hep 2012 02 14 Stattic Stat3 Inhibitor TC. Different columns indicate different conditions.

4.41 Experiment: braun hep 2012 04 10 Stattic Stat3 Inhibitor TC

4.41.1 Comments

Experimenter: Svantje Sobotta
Date: 2012-04-10
Cells: primary mouse hepatocytes
Treatment: IL-6 dose 50 ng/ml, Stattic 60 µmol/l

4.41.2 Model fit and plots

The model observables and the experimental data is shown in Figure S67. The 2 necessary parameter transformations are listed in Table S51. Note that transformations with only one entry are the same for all experimental conditions corresponding to this experiment.

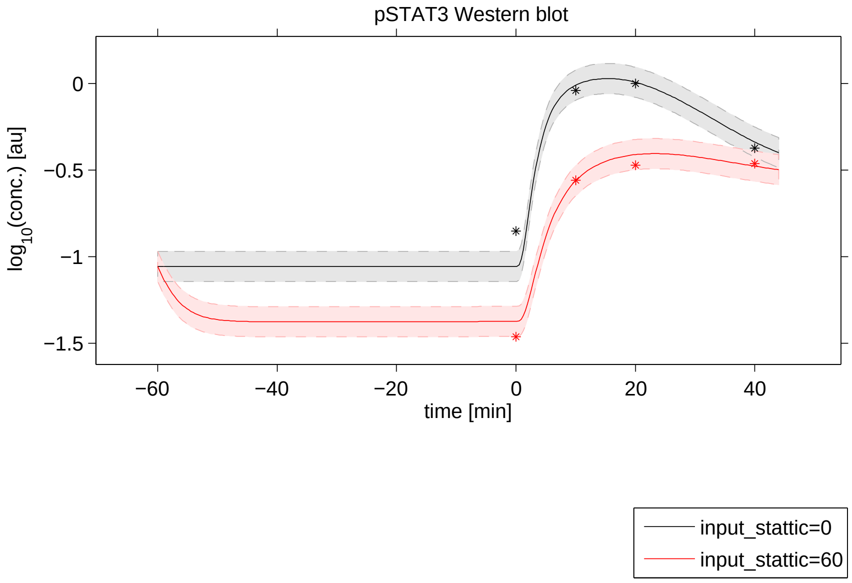

Figure S67: braun_hep_2012_04_10_Stattic_Stat3_Inhibitor_TC observables and experimental data for the experiment.

4.41.3 Condition dependent parameter changes

The following model parameters were changed to simulate these experimental conditions:

Parameter	Condition values	
input_il6	50	
input_stattic	0	60

Table S51: Model parameters modified for experiment braun hep 2012 04 10 Stattic Stat3 Inhibitor TC. Different columns indicate different conditions.

4.42 Experiment: braun hep 2012 05 02 Stattic Stat3 Inhibitor 40

4.42.1 Comments

Experimenter: Svantje Sobotta
Date: 2012-05-02
Cells: primary mouse hepatocytes
Treatment: IL-6 40 ng/ml = 1 nmol/l, Stattic 0 to 60 µmol/l

4.42.2 Model fit and plots

The model observables and the experimental data is shown in Figure S68. The necessary parameter transformation is listed in Table S52.

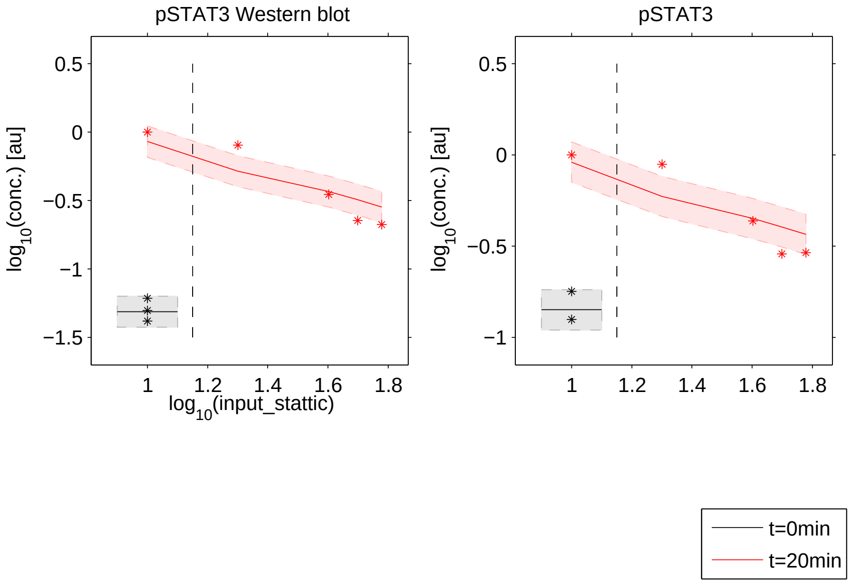

Figure S68: braun_hep_2012_05_02_Stattic_Stat3_Inhibitor_40 observables and experimental data for the experiment.

4.42.3 Condition dependent parameter changes

The following model parameters were changed to simulate these experimental conditions:

Parameter	Condition values				
input_stattic	0	20	40	50	60

Table S52: Model parameters modified for experiment braun hep 2012 05 02 Stattic Stat3 Inhibitor 40. Different columns indicate different conditions.

4.43 Experiment: braun hep 2012 05 02 Stattic Stat3 Inhibitor 60

4.43.1 Comments

Experimenter: Svantje Sobotta
Date: 2012-05-02
Cells: primary mouse hepatocytes
Treatment: IL-6 60 ng/ml, Stattic 0 to 60 µmol/l

4.43.2 Model fit and plots

The model observables and the experimental data is shown in Figure S69. The 2 necessary parameter transformations are listed in Table S53. Note that transformations with only one entry are the same for all experimental conditions corresponding to this experiment.

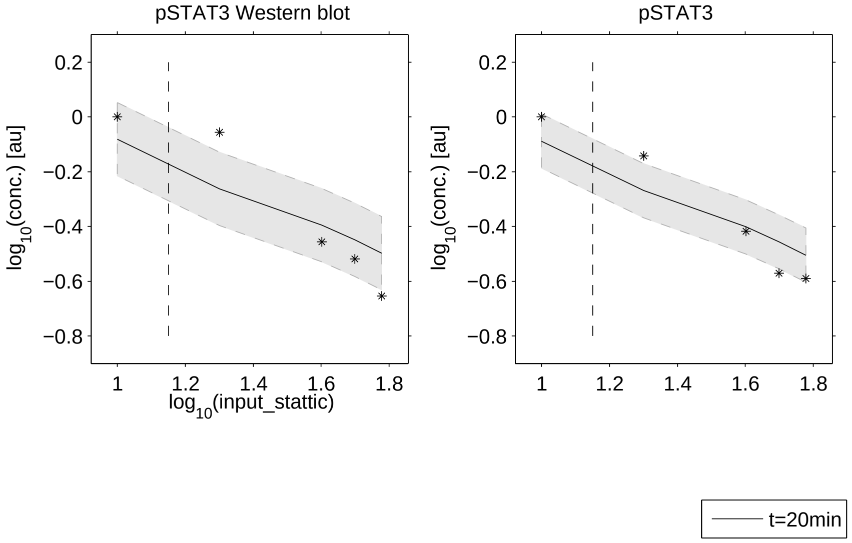

Figure S69: braun_hep_2012_05_02_Stattic_Stat3_Inhibitor_60 observables and experimental data for the experiment.

4.43.3 Condition dependent parameter changes

The following model parameters were changed to simulate these experimental conditions:

Parameter	Condition values				
input_il6			60		
input_stattic	0	20	40	50	60

Table S53: Model parameters modified for experiment braun hep 2012 05 02 Stattic Stat3 Inhibitor 60. Different columns indicate different conditions.

4.44 Experiment: braun hep 2013 06 03 Ruxolitinib

4.44.1 Comments

Experimenter: Svantje Sobotta
Date: 2013-06-03
Cells: primary mouse hepatocytes
Treatment: IL-6 dose 40 ng/ml, Ruxolitinib 0 to 5000 nmol/l

4.44.2 Model fit and plots

The model observables and the experimental data is shown in Figure S70. The 2 necessary parameter transformations are listed in Table S54. Note that transformations with only one entry are the same for all experimental conditions corresponding to this experiment.

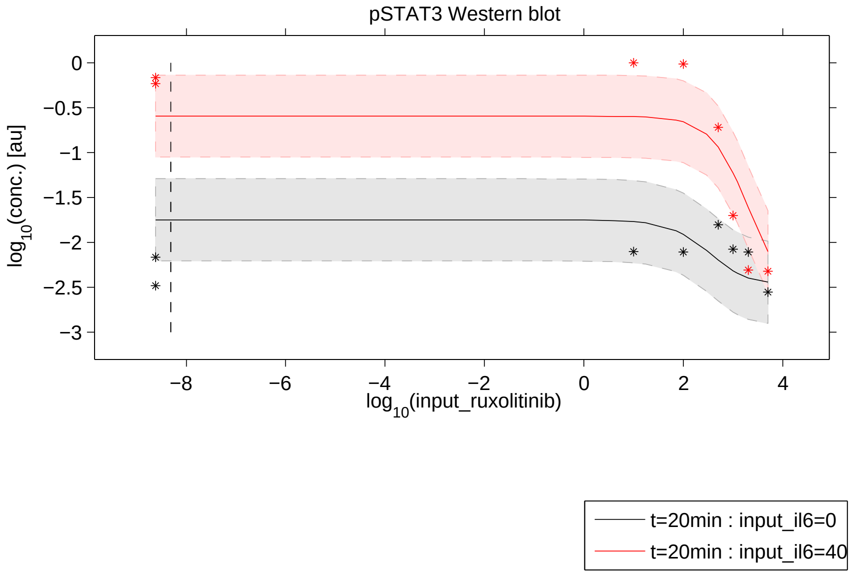

Figure S70: braun_hep_2013_06_03_Ruxolitinib observables and experimental data for the experiment.

4.44.3 Condition dependent parameter changes

The following model parameters were changed to simulate these experimental conditions:

Parameter	Condition values													
input_il6	0	0	0	0	0	0	0	40	40	40	40	40	40	40
input_ruxolitinib	0	10	100	1000	2000	500	5000	0	10	100	1000	2000	500	5000

Table S54: Model parameters modified for experiment braun hep 2013 06 03 Ruxolitinib. Different columns indicate different conditions.

4.45 Experiment: braun hep 2013 06 17 Ruxolitinib

4.45.1 Comments

Experimenter: Svantje Sobotta
Date: 2013-06-17
Cells: primary mouse hepatocytes
Treatment: IL-6 dose 40 ng/ml, Ruxolitinib 0 to 1000 nmol/l

4.45.2 Model fit and plots

The model observables and the experimental data is shown in Figure S71. The 2 necessary parameter transformations are listed in Table S55. Note that transformations with only one entry are the same for all experimental conditions corresponding to this experiment.

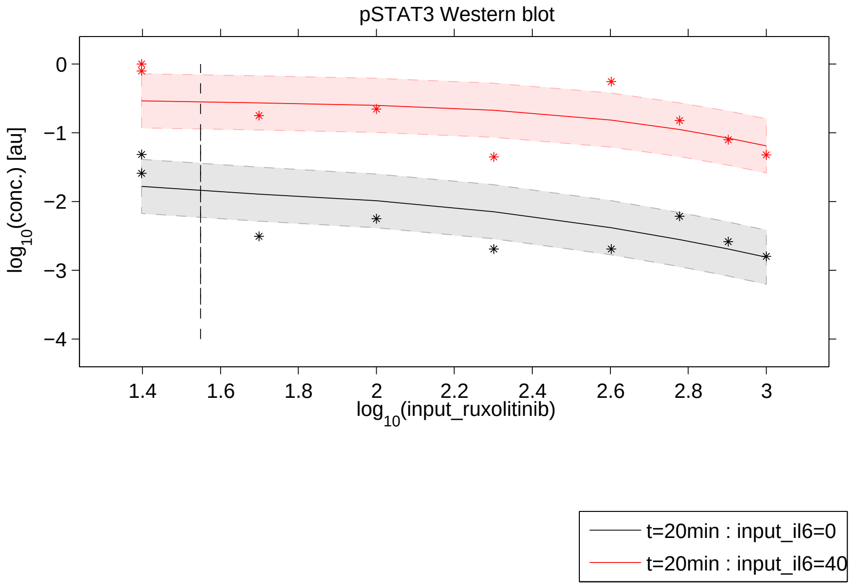

Figure S71: braun_hep_2013_06_17_Ruxolitinib observables and experimental data for the experiment.

4.45.3 Condition dependent parameter changes

The following model parameters were changed to simulate these experimental conditions:

Parameter	Condition values															
input_il6	0	0	0	0	0	0	0	0	40	40	40	40	40	40	40	40
input_ruxolitinib	0	100	1000	200	400	50	600	800	0	100	1000	200	400	50	600	800

Table S55: Model parameters modified for experiment braun hep 2013 06 17 Ruxolitinib. Different columns indicate different conditions.

4.46 Experiment: braun hep 2013 09 30 Ruxolitinib Inhibitor TC

4.46.1 Comments

Experimenter: Svantje Sobotta
Date: 2013-10-08
Cells: primary mouse hepatocytes
Treatment: IL-6 dose response 0-500 40 ng/ml, Ruxolitinib 500 nmol/l

4.46.2 Model fit and plots

The model observables and the experimental data is shown in Figure S72. The 2 necessary parameter transformations are listed in Table S56. Note that transformations with only one entry are the same for all experimental conditions corresponding to this experiment.

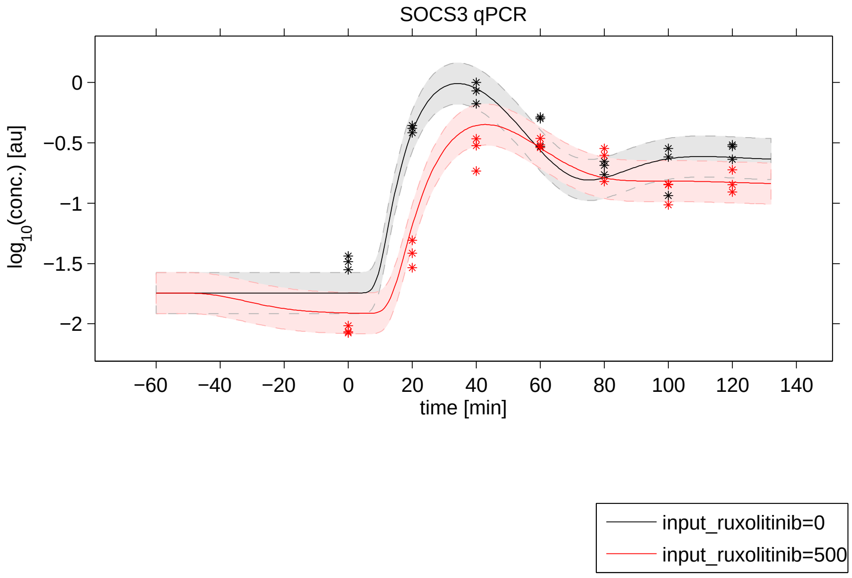

Figure S72: braun_hep_2013_09_30_Ruxolitinib_Inhibitor_TC observables and experimental data for the experiment.

4.46.3 Condition dependent parameter changes

The following model parameters were changed to simulate these experimental conditions:

Parameter	Condition values	
input_il6	100	
input_ruxolitinib	0	500

Table S56: Model parameters modified for experiment braun hep 2013 09 30 Ruxolitinib Inhibitor TC. Different columns indicate different conditions.

4.47 Experiment: xiaoyun nucSTAT3 Ratio

4.47.1 Model fit and plots

The model observables and the experimental data is shown in Figure S73. The necessary parameter transformation is listed in Table S57.

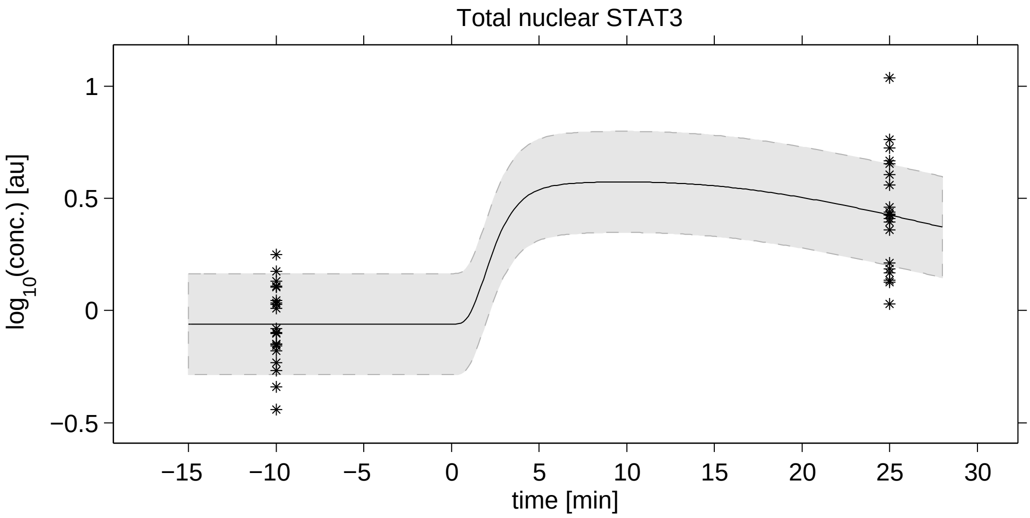

Figure S73: xiaoyun_nucSTAT3_Ratio observables and experimental data for the experiment.

4.47.2 Condition dependent parameter changes

The following model parameters were changed to simulate these experimental conditions:

Parameter	Condition values
input_il6	500

Table S57: Model parameters modified for experiment xiaoyun nucSTAT3 Ratio. Different columns indicate different conditions.

4.48 Experiment: APP calibration 1h

4.48.1 Model fit and plots

The model observables and the experimental data is shown in Figure S74. The necessary parameter transformation is listed in Table S58.

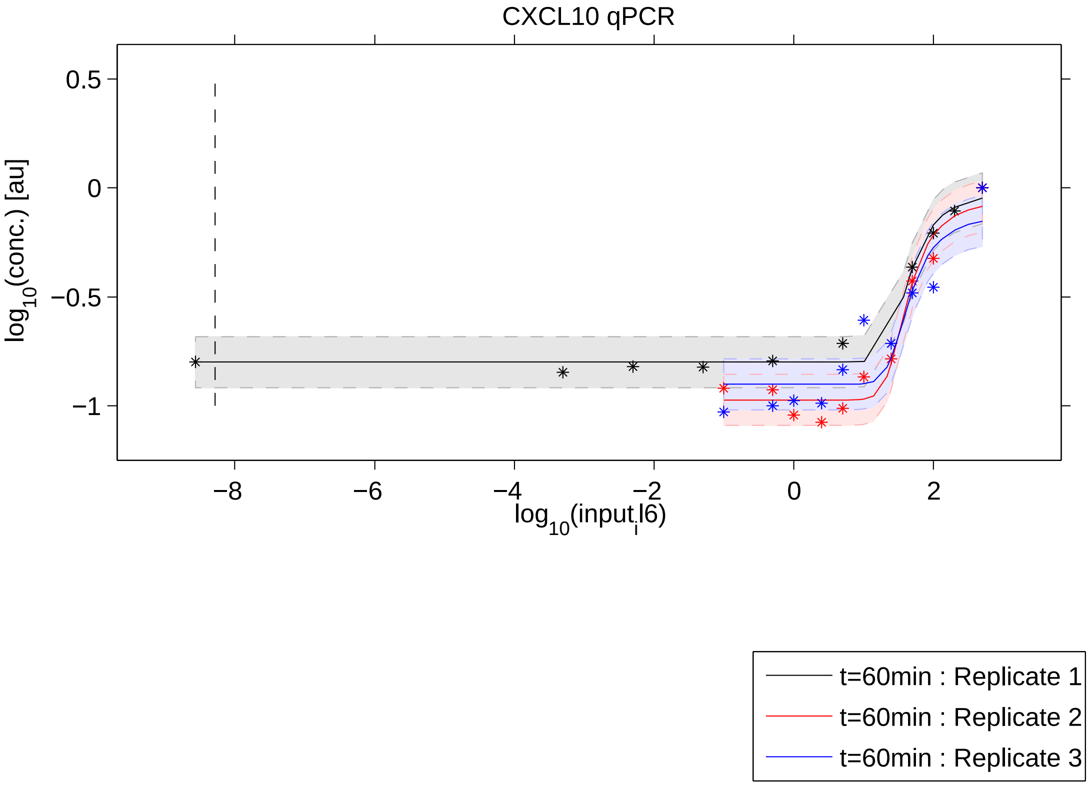

Figure S74: APP_calibration_1h observables and experimental data for the experiment.

4.48.2 Condition dependent parameter changes

The following model parameters were changed to simulate these experimental conditions:

Parameter	Condition values																													
input_il6	0	0.0005	0.005	0.05	0.5	100	200	5	50	500	0.1	0.5	1	10	100	2.5	25	5	50	500	0.1	0.5	1	10	100	2.5	25	5	50	500

Table S58: Model parameters modified for experiment APP calibration 1h. Different columns indicate different conditions.

4.49 Experiment: braun calibration hep 2014 01 13 IL6DR 6h Replicate1

4.49.1 Model fit and plots

The model observables and the experimental data is shown in Figure S75. The necessary parameter transformation is listed in Table S59.

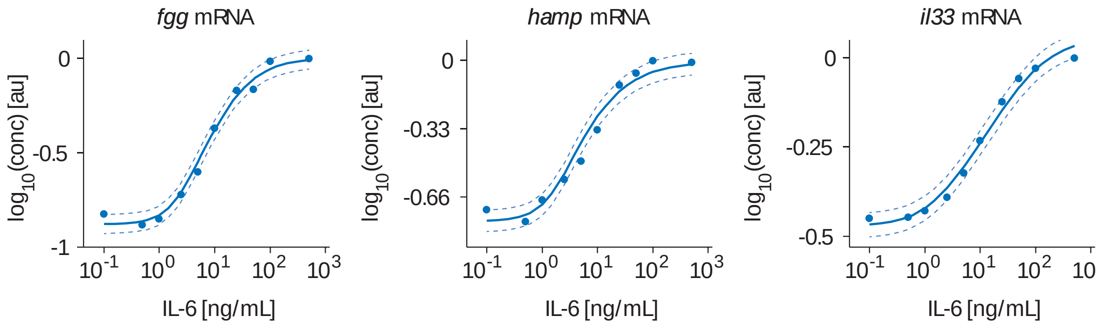

Figure S75: braun_calibration_hep_2014_01_13_IL6DR_6h_Replicate1 observables and experimental data for the experiment. Acute phase protein calibration data. Solid lines indicate model predictions, with dashed lines indicating standard deviation of the error model. Individual dots correspond to measured data points.

4.49.2 Condition dependent parameter changes

The following model parameters were changed to simulate these experimental conditions:

Parameter	Condition values									
input_il6	0.1	0.5	1	10	100	2.5	25	5	50	500

Table S59: Model parameters modified for experiment braun calibration hep 2014 01 13 IL6DR 6h - Replicate1. Different columns indicate different conditions.

4.50 Experiment: braun calibration hep 2014 01 13 IL6DR 6h Replicate2

4.50.1 Model fit and plots

The model observables and the experimental data is shown in Figure S76. The necessary parameter transformation is listed in Table S60.

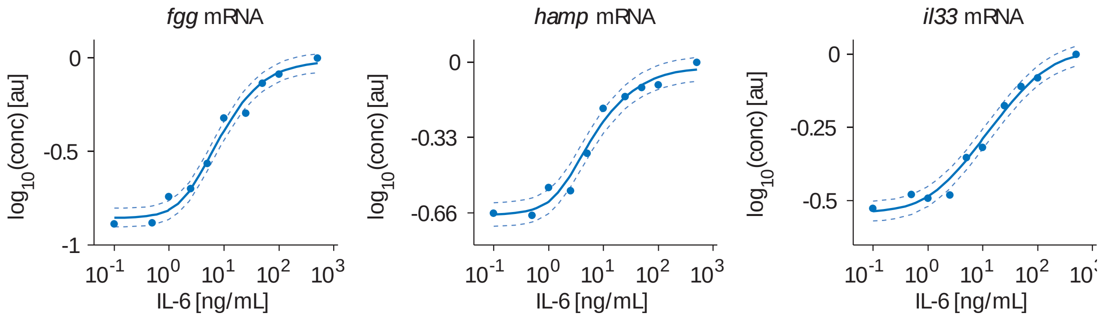

Figure S76: braun_calibration_hep_2014_01_13_IL6DR_6h_Replicate2 observables and experimental data for the experiment. Acute phase protein calibration data. Solid lines indicate model predictions, with dashed lines indicating standard deviation of the error model. Individual dots correspond to measured data points.

4.50.2 Condition dependent parameter changes

The following model parameters were changed to simulate these experimental conditions:

Parameter	Condition values									
input_il6	0.1	0.5	1	10	100	2.5	25	5	50	500

Table S60: Model parameters modified for experiment braun calibration hep 2014 01 13 IL6DR 6h - Replicate2. Different columns indicate different conditions.

4.51 Experiment: braun calibration hep 2014 01 13 IL6DR 6h Replicate3

4.51.1 Model fit and plots

The model observables and the experimental data is shown in Figure S77. The necessary parameter transformation is listed in Table S61.

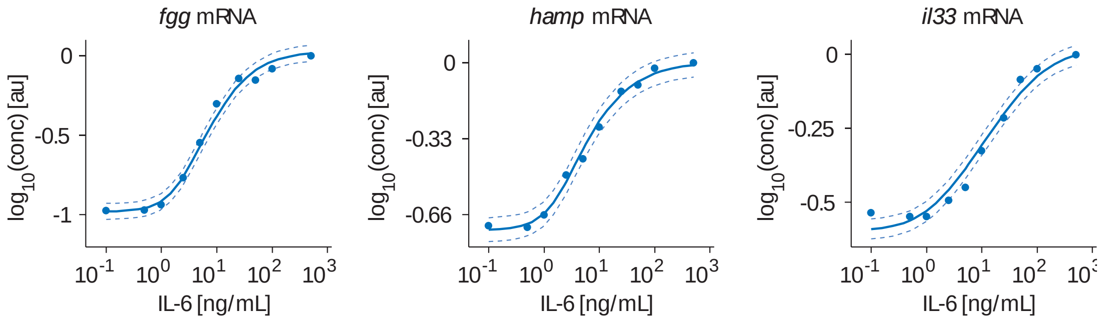

Figure S77: braun_calibration_hep_2014_01_13_IL6DR_6h_Replicate3 observables and experimental data for the experiment. Acute phase protein calibration data. Solid lines indicate model predictions, with dashed lines indicating standard deviation of the error model. Individual dots correspond to measured data points.

4.51.2 Condition dependent parameter changes

The following model parameters were changed to simulate these experimental conditions:

Parameter	Condition values									
input_il6	0.1	0.5	1	10	100	2.5	25	5	50	500

Table S61: Model parameters modified for experiment braun calibration hep 2014 01 13 IL6DR 6h - Replicate3. Different columns indicate different conditions.

4.52 Experiment: braun calibration hep 2014 01 27 IL6DR 24h Replicate1

4.52.1 Model fit and plots

The model observables and the experimental data is shown in Figure S78. The necessary parameter transformation is listed in Table S62.

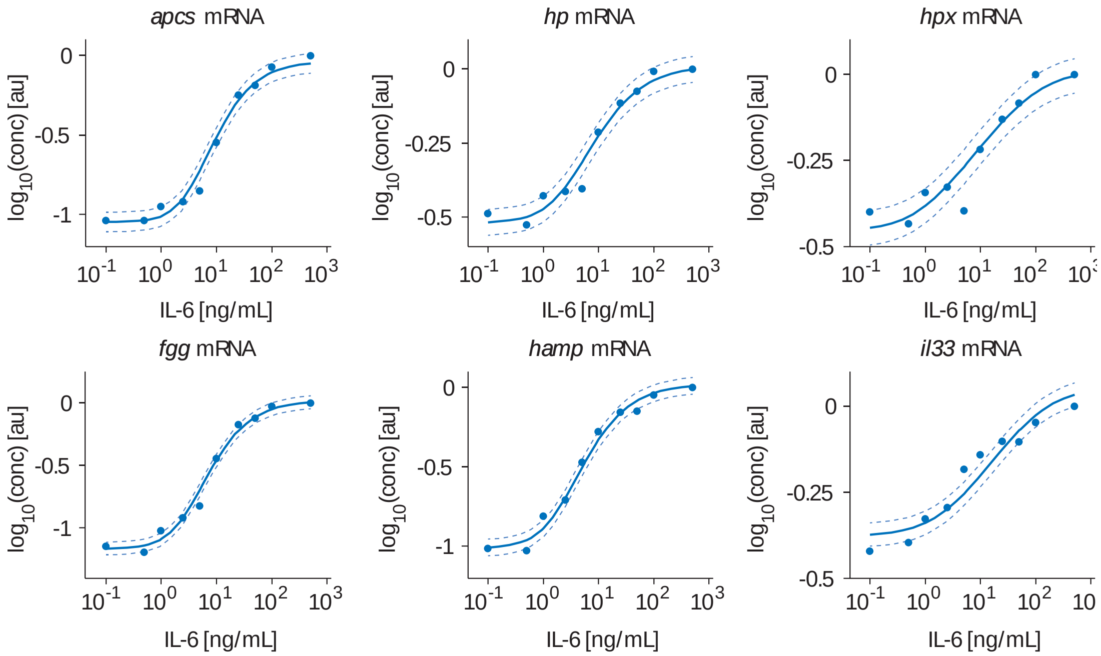

Figure S78: braun_calibration_hep_2014_01_27_IL6DR_24h_Replicate1 observables and experimental data for the experiment. Acute phase protein calibration data. Solid lines indicate model predictions, with dashed lines indicating standard deviation of the error model. Individual dots correspond to measured data points.

4.52.2 Condition dependent parameter changes

The following model parameters were changed to simulate these experimental conditions:

Parameter	Condition values									
input_il6	0.1	0.5	1	10	100	2.5	25	5	50	500

Table S62: Model parameters modified for experiment braun calibration hep 2014 01 27 IL6DR 24h - Replicate1. Different columns indicate different conditions.

4.53 Experiment: braun calibration hep 2014 01 27 IL6DR 24h Replicate2

4.53.1 Model fit and plots

The model observables and the experimental data is shown in Figure S79. The necessary parameter transformation is listed in Table S63.

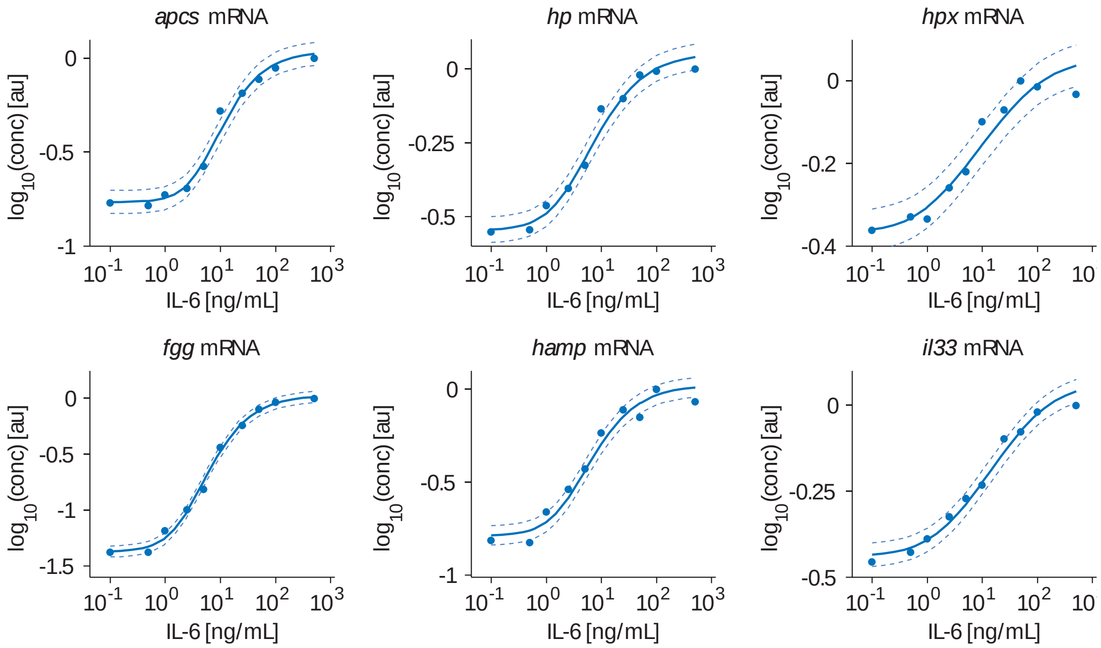

Figure S79: braun_calibration_hep_2014_01_27_IL6DR_24h_Replicate2 observables and experimental data for the experiment. Acute phase protein calibration data. Solid lines indicate model predictions, with dashed lines indicating standard deviation of the error model. Individual dots correspond to measured data points.

4.53.2 Condition dependent parameter changes

The following model parameters were changed to simulate these experimental conditions:

Parameter	Condition values									
input_il6	0.1	0.5	1	10	100	2.5	25	5	50	500

Table S63: Model parameters modified for experiment braun calibration hep 2014 01 27 IL6DR 24h - Replicate2. Different columns indicate different conditions.

4.54 Experiment: braun calibration hep 2014 01 27 IL6DR 24h Replicate3

4.54.1 Model fit and plots

The model observables and the experimental data is shown in Figure S80. The necessary parameter transformation is listed in Table S64.

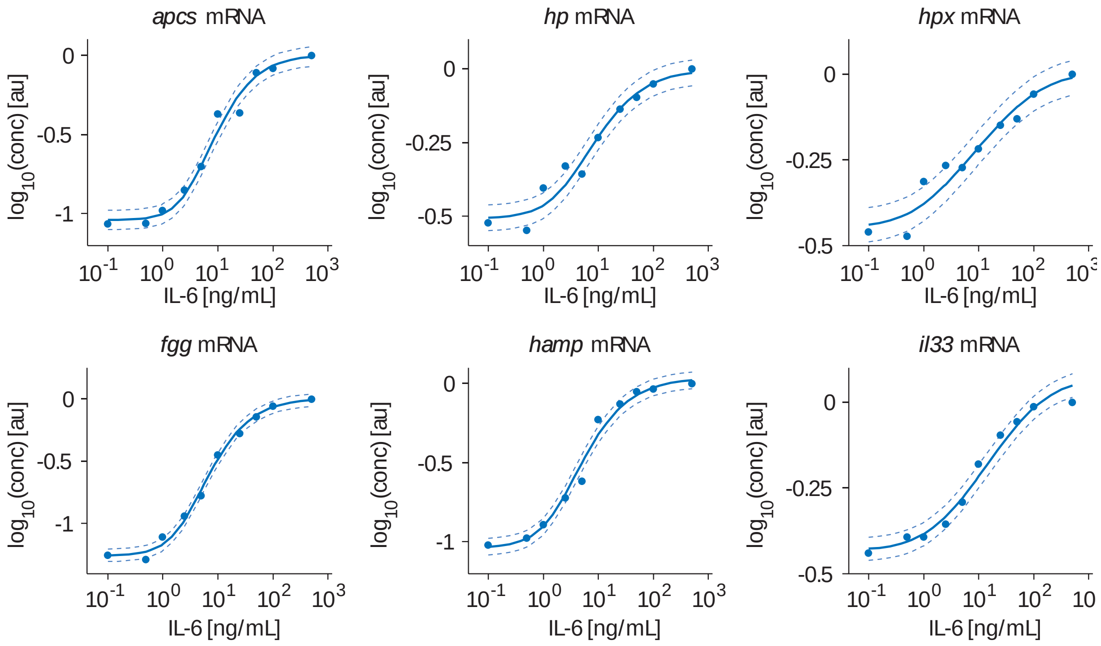

Figure S80: braun_calibration_hep_2014_01_27_IL6DR_24h_Replicate3 observables and experimental data for the experiment. Acute phase protein calibration data. Solid lines indicate model predictions, with dashed lines indicating standard deviation of the error model. Individual dots correspond to measured data points.

4.54.2 Condition dependent parameter changes

The following model parameters were changed to simulate these experimental conditions:

Parameter	Condition values									
input_il6	0.1	0.5	1	10	100	2.5	25	5	50	500

Table S64: Model parameters modified for experiment braun calibration hep 2014 01 27 IL6DR 24h - Replicate3. Different columns indicate different conditions.

4.55 Experiment: xiaoyun calibration APP tc replicate1

4.55.1 Comments

Experimenter: Xiaoyun Huang
Cells: primary mouse hepatocytes
Treatment: 0, 40 and 100 ng/ml IL-6 constant

4.55.2 Model fit and plots

The model observables and the experimental data is shown in Figure S81. This experiment requires 7 custom inputs which are defined in Table S65. The necessary parameter transformation is listed in Table S67.

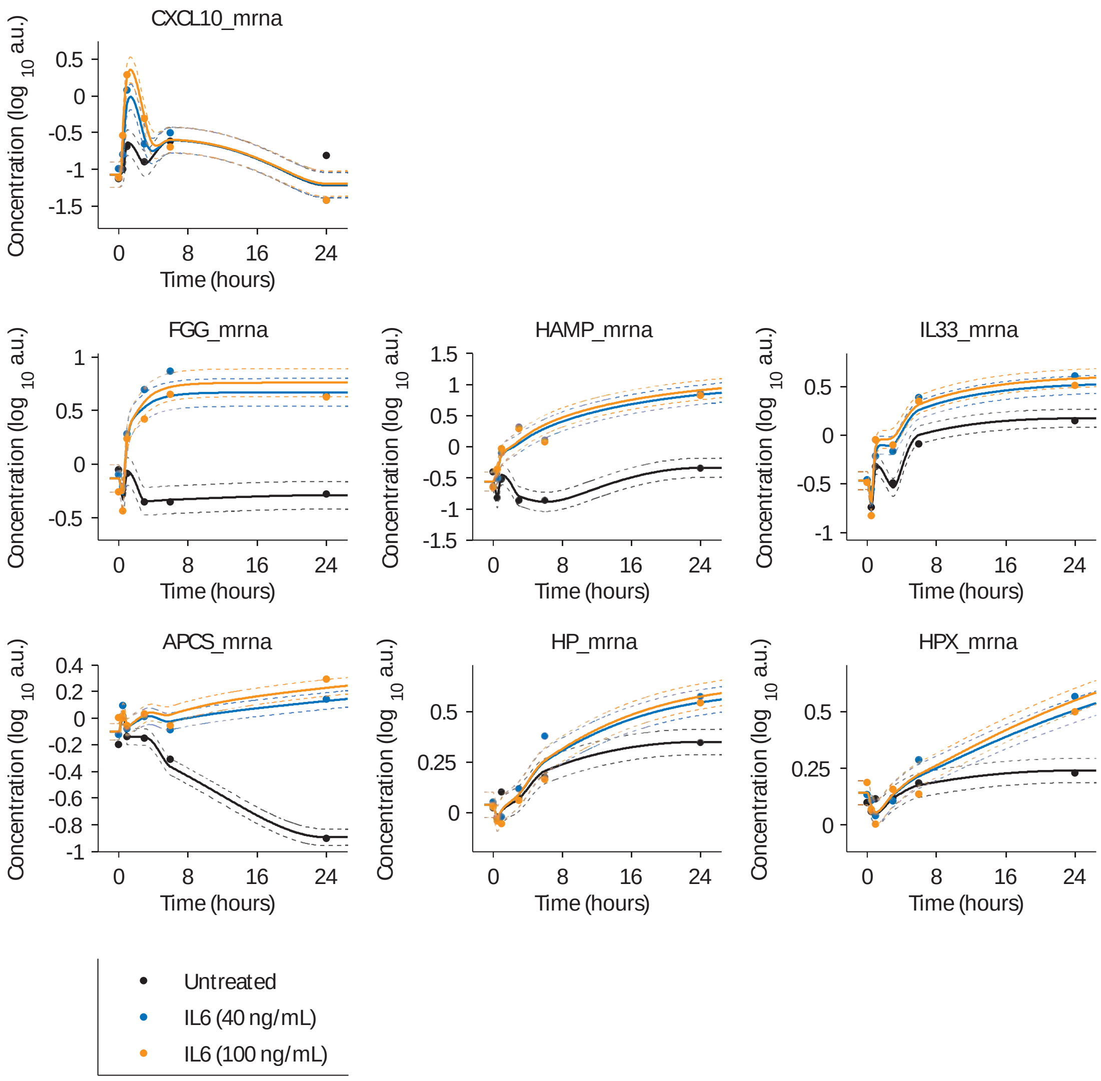

Figure S81: xiaoyun_calibration_APP_tc_replicate1 observables and experimental data for the experiment. Solid lines indicate model predictions, with dashed lines indicating standard deviation of the error model. Individual dots correspond to measured data points. The background signal of the control experiment is described by a monotonic spline and estimated from the control data.

Input	Unit	Modified equation	Description
CXCL10_background	conc. [nmol/l]	monospline10(t)	Monotonic spline describing CXCL10 background of replicate 1
APCS_background	conc. [nmol/l]	monospline10(t)	Monotonic spline describing APCS background of replicate 1
FGG_background	conc. [nmol/l]	monospline10(t)	Monotonic spline describing FGG background of replicate 1
HP_background	conc. [nmol/l]	monospline10(t)	Monotonic spline describing HP background of replicate 1
HPX_background	conc. [nmol/l]	monospline10(t)	Monotonic spline describing HPX background of replicate 1
IL33_background	conc. [nmol/l]	monospline10(t)	Monotonic spline describing IL33 background of replicate 1
HAMP_background	conc. [nmol/l]	monospline10(t)	Monotonic spline describing HAMP background of replicate 1

Table S65: Inputs modified for experiment xiaoyun_calibration_APP_tc_replicate1

4.55.3 Observables

The following observables are added in this data set:

Observable		Equations
CXCL10_mrna [au]	y	$\log_{10}$([CXCL10_background] + offset_cxcl10_qpcr_tc + [CXCL10RNA] · scale_cxcl10_qpcr_tc_1)
	σ	sd_cxcl10_qpcr_tc_1
APCS_mrna [au]	y	$\log_{10}$([APCS_background] + offset_apcs_qpcr_tc + [APCSRNA] · scale_apcs_qpcr_tc_1)
	σ	sd_apcs_qpcr_tc_1
FGG_mrna [au]	y	$\log_{10}$([FGG_background] + offset_fgg_qpcr_tc + [FGGRNA] · scale_fgg_qpcr_tc_1)
	σ	sd_fgg_qpcr_tc_1
HP_mrna [au]	y	$\log_{10}$([HP_background] + offset_hp_qpcr_tc + [HPRNA] · scale_hp_qpcr_tc_1)
	σ	sd_hp_qpcr_tc_1
HPX_mrna [au]	y	$\log_{10}$([HPX_background] + offset_hpx_qpcr_tc + [HPXRNA] · scale_hpx_qpcr_tc_1)
	σ	sd_hpx_qpcr_tc_1
IL33_mrna [au]	y	$\log_{10}$([IL33_background] + offset_il33_qpcr_tc + [IL33RNA] · scale_il33_qpcr_tc_1)
	σ	sd_il33_qpcr_tc_1
HAMP_mrna [au]	y	$\log_{10}$([HAMP_background] + offset_hamp_qpcr_tc + [HAMPRNA] · scale_hamp_qpcr_tc_1)
	σ	sd_hamp_qpcr_tc_1

Table S66: Observables added for experiment xiaoyun_calibration_APP_tc_replicate1

4.55.4 Condition dependent parameter changes

The following model parameters were changed to simulate these experimental conditions:

Parameter	Condition	values	
input_il6	0	100	40

Table S67: Model parameters modified for experiment xiaoyun calibration APP tc replicate1. Different columns indicate different conditions.

4.56 Experiment: xiaoyun calibration APP tc replicate2

4.56.1 Comments

Experimenter: Xiaoyun Huang
Cells: primary mouse hepatocytes
Treatment: 0, 40 and 100 ng/ml IL-6 constant

4.56.2 Model fit and plots

The model observables and the experimental data is shown in Figure S82. This experiment requires 7 custom inputs which are defined in Table S68. The necessary parameter transformation is listed in Table S70.

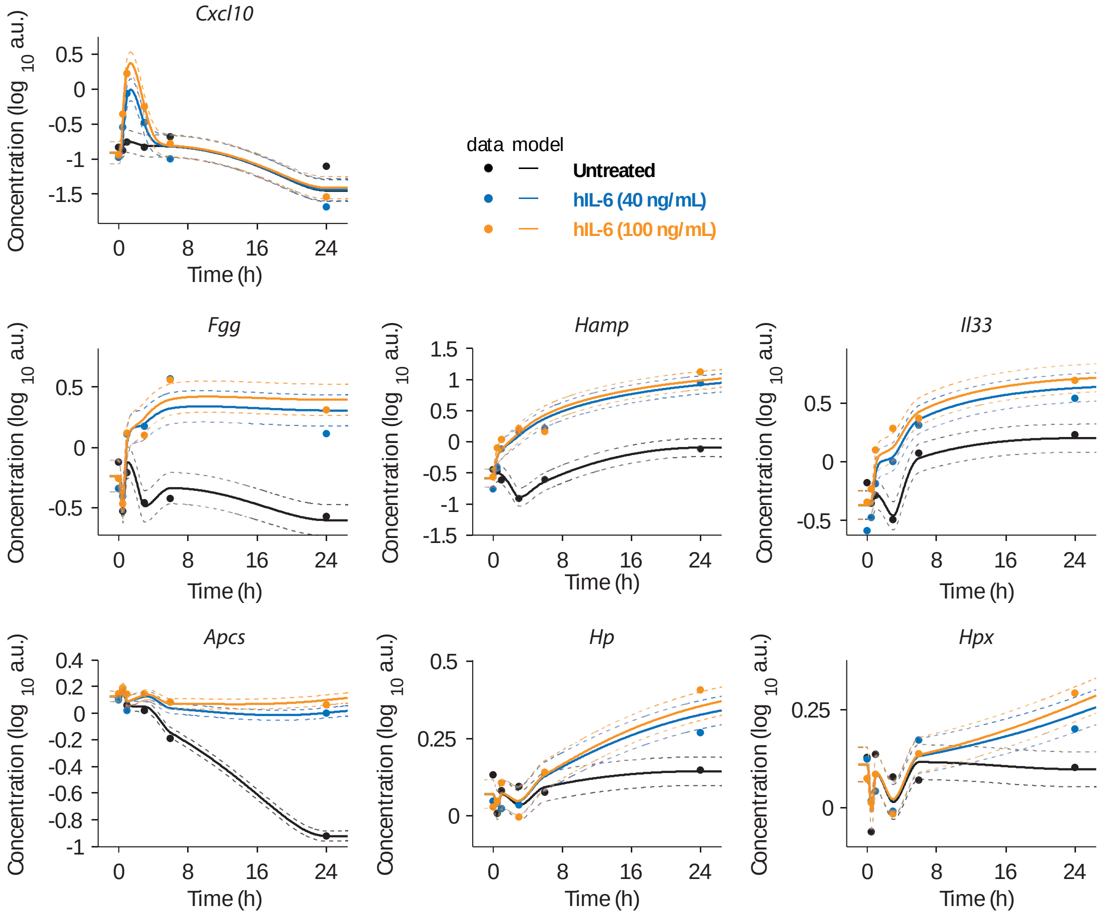

Figure S82: xiaoyun_calibration_APP_tc_replicate2 observables and experimental data for the experiment. Solid lines indicate model predictions, with dashed lines indicating standard deviation of the error model. Individual dots correspond to measured data points. The background signal of the control experiment is described by a monotonic spline and estimated from the control data.

Input	Unit	Modified equation	Description
CXCL10_background	conc. [nmol/l]	monospline10(t)	Monotonic spline describing CXCL10 background of replicate 2
APCS_background	conc. [nmol/l]	monospline10(t)	Monotonic spline describing APCS background of replicate 2
FGG_background	conc. [nmol/l]	monospline10(t)	Monotonic spline describing FGG background of replicate 2
HP_background	conc. [nmol/l]	monospline10(t)	Monotonic spline describing HP background of replicate 2
HPX_background	conc. [nmol/l]	monospline10(t)	Monotonic spline describing HPX background of replicate 2
IL33_background	conc. [nmol/l]	monospline10(t)	Monotonic spline describing IL33 background of replicate 2
HAMP_background	conc. [nmol/l]	monospline10(t)	Monotonic spline describing HAMP background of replicate 2

Table S68: Inputs modified for experiment xiaoyun_calibration_APP_tc_replicate2

4.56.3 Observables

The following observables are added in this data set:

Observable		Equations
CXCL10_mrna [au]	y	$\log_{10}$([CXCL10_background] + offset_cxcl10_qpcr_tc + [CXCL10RNA] · scale_cxcl10_qpcr_tc_2)
	σ	sd_cxcl10_qpcr_tc_2
APCS_mrna [au]	y	$\log_{10}$([APCS_background] + offset_apcs_qpcr_tc + [APCSRNA] · scale_apcs_qpcr_tc_2)
	σ	sd_apcs_qpcr_tc_2
FGG_mrna [au]	y	$\log_{10}$([FGG_background] + offset_fgg_qpcr_tc + [FGGRNA] · scale_fgg_qpcr_tc_2)
	σ	sd_fgg_qpcr_tc_2
HP_mrna [au]	y	$\log_{10}$([HP_background] + offset_hp_qpcr_tc + [HPRNA] · scale_hp_qpcr_tc_2)
	σ	sd_hp_qpcr_tc_2
HPX_mrna [au]	y	$\log_{10}$([HPX_background] + offset_hpx_qpcr_tc + [HPXRNA] · scale_hpx_qpcr_tc_2)
	σ	sd_hpx_qpcr_tc_2
IL33_mrna [au]	y	$\log_{10}$([IL33_background] + offset_il33_qpcr_tc + [IL33RNA] · scale_il33_qpcr_tc_2)
	σ	sd_il33_qpcr_tc_2
HAMP_mrna [au]	y	$\log_{10}$([HAMP_background] + offset_hamp_qpcr_tc + [HAMPRNA] · scale_hamp_qpcr_tc_2)
	σ	sd_hamp_qpcr_tc_2

Table S69: Observables added for experiment xiaoyun_calibration_APP_tc_replicate2

4.56.4 Condition dependent parameter changes

The following model parameters were changed to simulate these experimental conditions:

Parameter	Condition	values	
input_il6	0	100	40

Table S70: Model parameters modified for experiment xiaoyun calibration APP tc replicate2. Different columns indicate different conditions.

4.57 Experiment: braun validation Ruxolitinib 1h hep 2013 10 14 qPCR 140224 IL6DR Inh 1h Cxcl10

4.57.1 Model fit and plots

The model observables and the experimental data is shown in Figure S83. The 2 necessary parameter transformations are listed in Table S71. Note that transformations with only one entry are the same for all experimental conditions corresponding to this experiment.

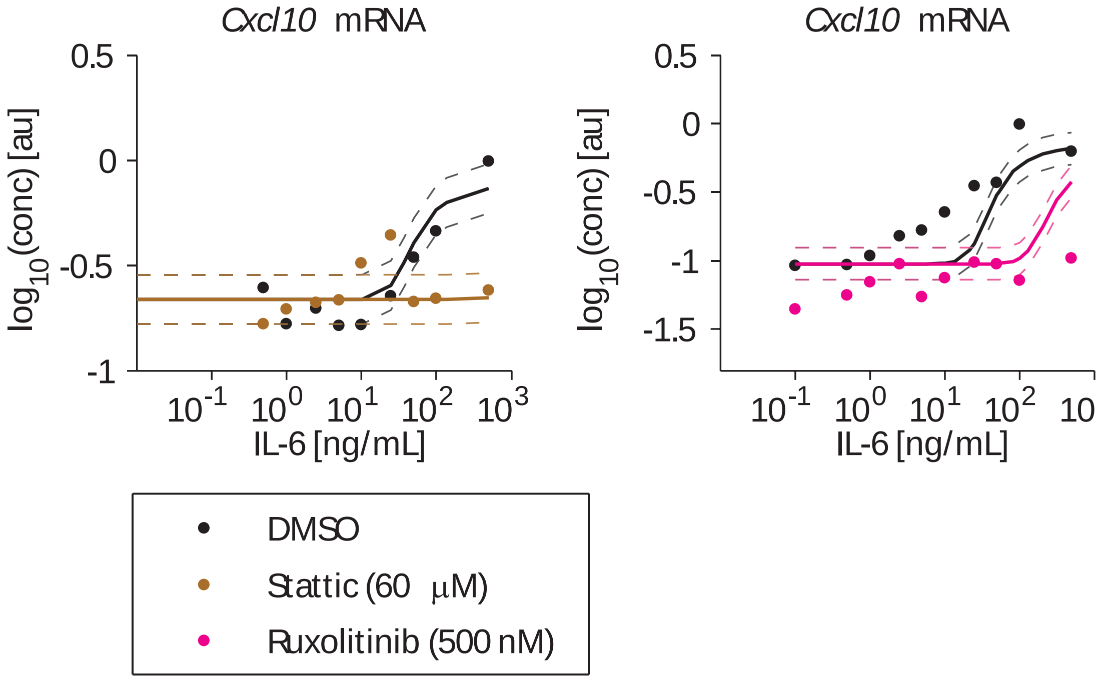

Figure S83: braun_validation_Ruxolitinib_1h_hep_2013_10_14_qPCR_140224_IL6DR_Inh_1h_Cxcl10 observables and experimental data for the experiment. Acute phase protein validation data. Solid lines indicate model predictions, with dashed lines indicating standard deviation of the error model. Individual dots correspond to measured data points. Note that all dynamic model parameters were held fixed when fitting this data.

4.57.2 Condition dependent parameter changes

The following model parameters were changed to simulate these experimental conditions:

Parameter	Condition values																			
input_il6	0.1	0.1	0.5	0.5	1	1	10	10	100	100	2.5	2.5	25	25	5	5	50	50	500	500
input_ruxolitinib	0	500	0	500	0	500	0	500	0	500	0	500	0	500	0	500	0	500	0	500

Table S71: Model parameters modified for experiment braun validation Ruxolitinib 1h hep 2013 10 14 qPCR 140224 IL6DR Inh 1h Cxcl10. Different columns indicate different conditions.

4.58 Experiment: braun validation Ruxolitinib 1h hep 2014 04 28 IL6DR Rux 1h CXCL10

4.58.1 Model fit and plots

The model observables and the experimental data is shown in Figure S84. The 2 necessary parameter transformations are listed in Table S72. Note that transformations with only one entry are the same for all experimental conditions corresponding to this experiment.

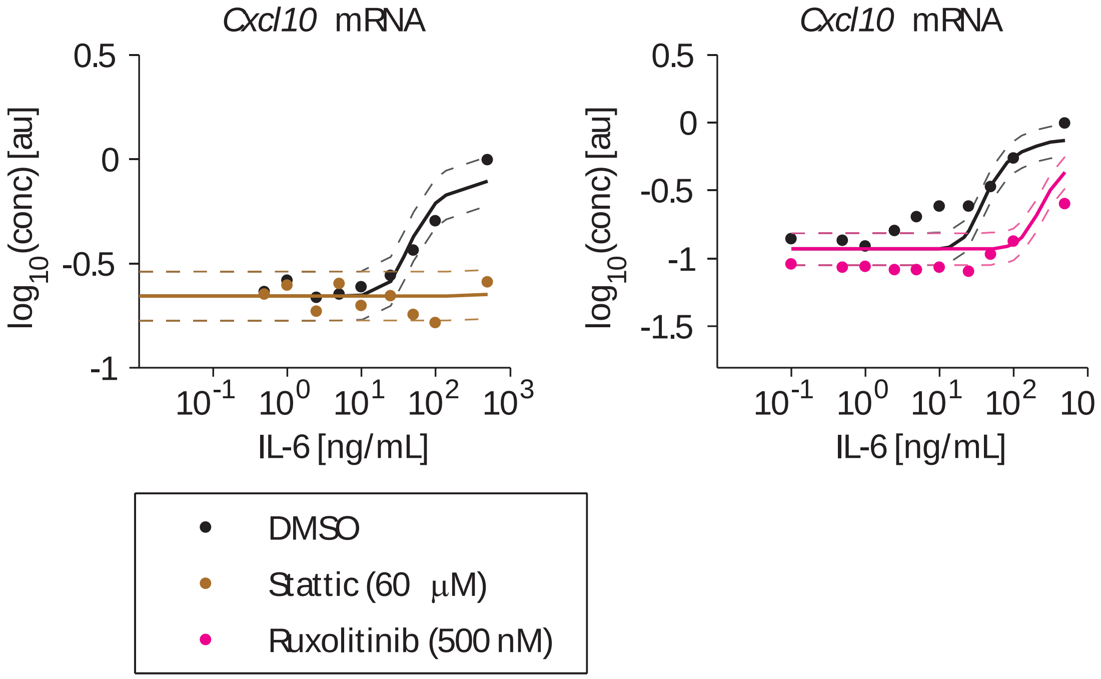

Figure S84: braun_validation_Ruxolitinib_1h_hep_2014_04_28_IL6DR_Rux_1h_CXCL10 observables and experimental data for the experiment. Acute phase protein validation data. Solid lines indicate model predictions, with dashed lines indicating standard deviation of the error model. Individual dots correspond to measured data points. Note that all dynamic model parameters were held fixed when fitting this data.

4.58.2 Condition dependent parameter changes

The following model parameters were changed to simulate these experimental conditions:

Parameter	Condition values																			
input_il6	0.1	0.1	0.5	0.5	1	1	10	10	100	100	2.5	2.5	25	25	5	5	50	50	500	500
input_ruxolitinib	0	500	0	500	0	500	0	500	0	500	0	500	0	500	0	500	0	500	0	500

Table S72: Model parameters modified for experiment braun validation Ruxolitinib 1h hep 2014 04 28 IL6DR Rux 1h CXCL10. Different columns indicate different conditions.

4.59 Experiment: braun validation Ruxolitinib 6h hep 2014 04 22 qPCR 140526 IL6DR Rux 6h APP

4.59.1 Model fit and plots

The model observables and the experimental data is shown in Figure S85. The 2 necessary parameter transformations are listed in Table S73. Note that transformations with only one entry are the same for all experimental conditions corresponding to this experiment.

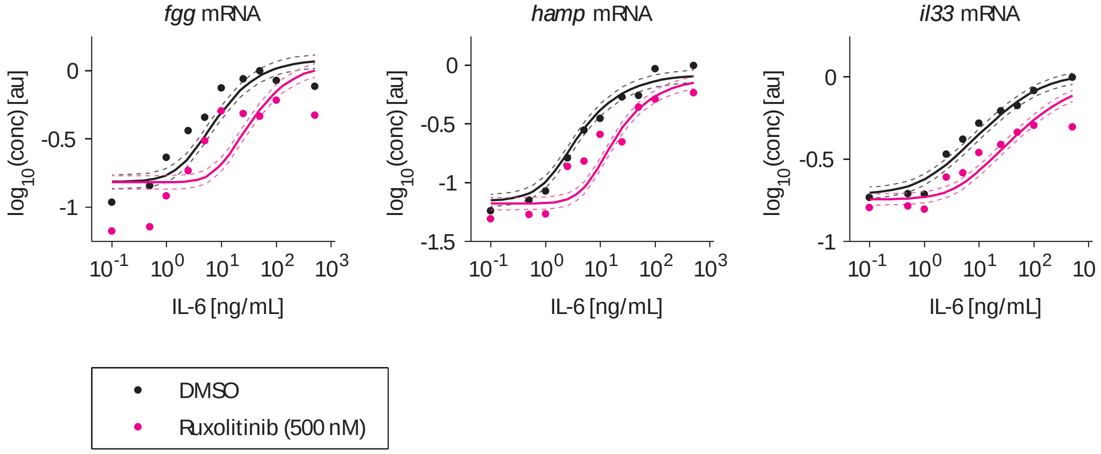

Figure S85: braun_validation_Ruxolitinib_6h_hep_2014_04_22_qPCR_140526_IL6DR_Rux_6h_APP observables and experimental data for the experiment. Acute phase protein validation data. Solid lines indicate model predictions, with dashed lines indicating standard deviation of the error model. Individual dots correspond to measured data points. Note that all dynamic model parameters were held fixed when fitting this data.

4.59.2 Condition dependent parameter changes

The following model parameters were changed to simulate these experimental conditions:

Parameter	Condition values																			
input_il6	0.1	0.1	0.5	0.5	1	1	10	10	100	100	2.5	2.5	25	25	5	5	50	50	500	500
input_ruxolitinib	0	500	0	500	0	500	0	500	0	500	0	500	0	500	0	500	0	500	0	500

Table S73: Model parameters modified for experiment braun validation Ruxolitinib 6h hep 2014 04 22 qPCR 140526 IL6DR Rux 6h APP. Different columns indicate different conditions.

4.60 Experiment: braun validation Ruxolitinib 6h hep 2014 05 19 qPCR 140526 IL6DR Rux 6h APP

4.60.1 Model fit and plots

The model observables and the experimental data is shown in Figure S86. The 2 necessary parameter transformations are listed in Table S74. Note that transformations with only one entry are the same for all experimental conditions corresponding to this experiment.

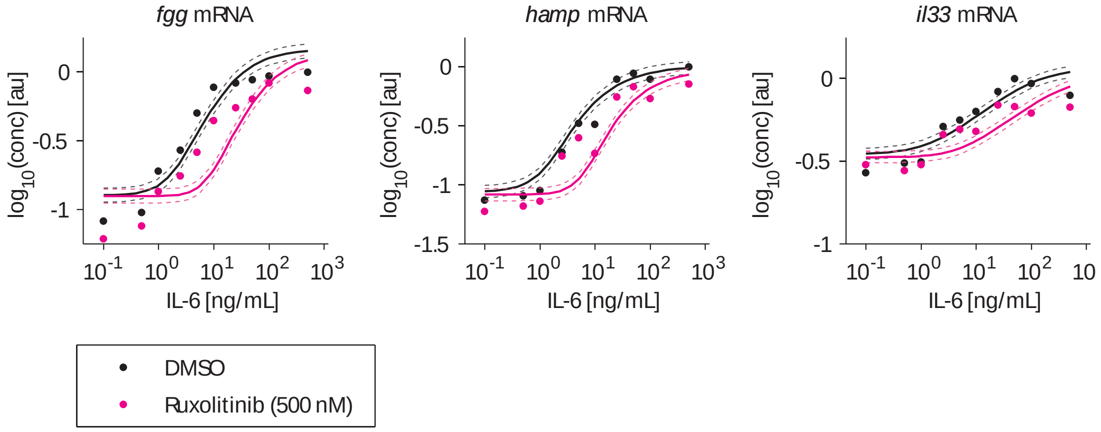

Figure S86: braun_validation_Ruxolitinib_6h_hep_2014_05_19_qPCR_140526_IL6DR_Rux_6h_APP observables and experimental data for the experiment. Acute phase protein validation data. Solid lines indicate model predictions, with dashed lines indicating standard deviation of the error model. Individual dots correspond to measured data points. Note that all dynamic model parameters were held fixed when fitting this data.

4.60.2 Condition dependent parameter changes

The following model parameters were changed to simulate these experimental conditions:

Parameter	Condition values																			
input_il6	0.1	0.1	0.5	0.5	1	1	10	10	100	100	2.5	2.5	25	25	5	5	50	50	500	500
input_ruxolitinib	0	500	0	500	0	500	0	500	0	500	0	500	0	500	0	500	0	500	0	500

Table S74: Model parameters modified for experiment braun validation Ruxolitinib 6h hep 2014 05 19 qPCR 140526 IL6DR Rux 6h APP. Different columns indicate different conditions.

4.61 Experiment: braun validation Ruxolitinib 24h hep 2014 04 22 qPCR 140604 IL6DR Rux 24h APP

4.61.1 Model fit and plots

The model observables and the experimental data is shown in Figure S87. The 2 necessary parameter transformations are listed in Table S75. Note that transformations with only one entry are the same for all experimental conditions corresponding to this experiment.

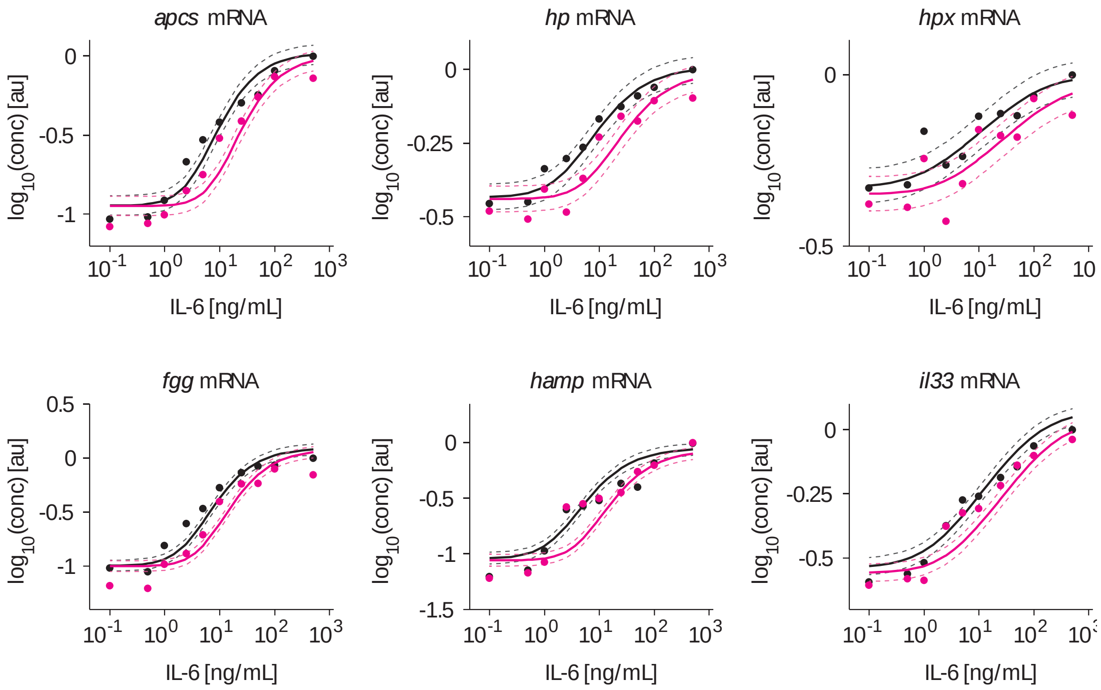

Figure S87: braun_validation_Ruxolitinib_24h_hep_2014_04_22_qPCR_140604_IL6DR_Rux_24h_APP observables and experimental data for the experiment. Acute phase protein validation data. Solid lines indicate model predictions, with dashed lines indicating standard deviation of the error model. Individual dots correspond to measured data points. Note that all dynamic model parameters were held fixed when fitting this data.

4.61.2 Condition dependent parameter changes

The following model parameters were changed to simulate these experimental conditions:

Parameter	Condition values																			
input_il6	0.1	0.1	0.5	0.5	1	1	10	10	100	100	2.5	2.5	25	25	5	5	50	50	500	500
input_ruxolitinib	0	500	0	500	0	500	0	500	0	500	0	500	0	500	0	500	0	500	0	500

Table S75: Model parameters modified for experiment braun validation Ruxolitinib 24h hep 2014 04 22 qPCR 140604 IL6DR Rux 24h APP. Different columns indicate different conditions.

4.62 Experiment: braun validation Ruxolitinib 24h hep 2014 05 19 qPCR 140604 IL6DR Rux 24h APP

4.62.1 Model fit and plots

The model observables and the experimental data is shown in Figure S88. The 2 necessary parameter transformations are listed in Table S76. Note that transformations with only one entry are the same for all experimental conditions corresponding to this experiment.

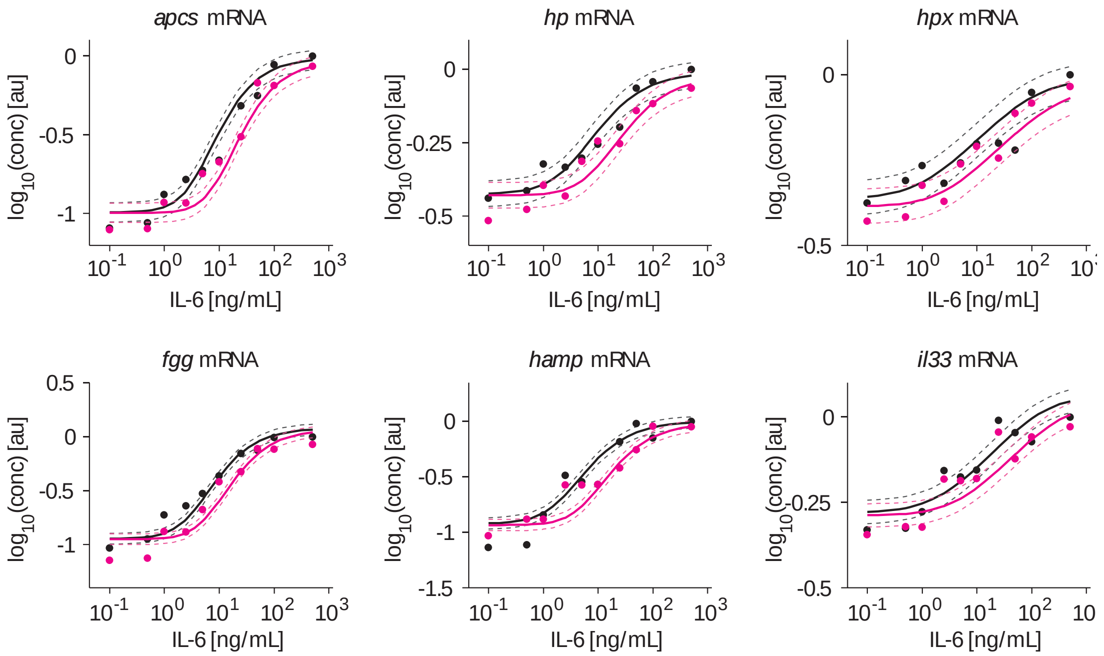

Figure S88: braun_validation_Ruxolitinib_24h_hep_2014_05_19_qPCR_140604_IL6DR_Rux_24h_APP observables and experimental data for the experiment. Acute phase protein validation data. Solid lines indicate model predictions, with dashed lines indicating standard deviation of the error model. Individual dots correspond to measured data points. Note that all dynamic model parameters were held fixed when fitting this data.

4.62.2 Condition dependent parameter changes

The following model parameters were changed to simulate these experimental conditions:

Parameter	Condition values																			
input_il6	0.1	0.1	0.5	0.5	1	1	10	10	100	100	2.5	2.5	25	25	5	5	50	50	500	500
input_ruxolitinib	0	500	0	500	0	500	0	500	0	500	0	500	0	500	0	500	0	500	0	500

Table S76: Model parameters modified for experiment braun validation Ruxolitinib 24h hep 2014 05 19 qPCR 140604 IL6DR Rux 24h APP. Different columns indicate different conditions.

4.63 Experiment: APP validation 1h

4.63.1 Model fit and plots

The model observables and the experimental data is shown in Figure S89. The 2 necessary parameter transformations are listed in Table S77. Note that transformations with only one entry are the same for all experimental conditions corresponding to this experiment.

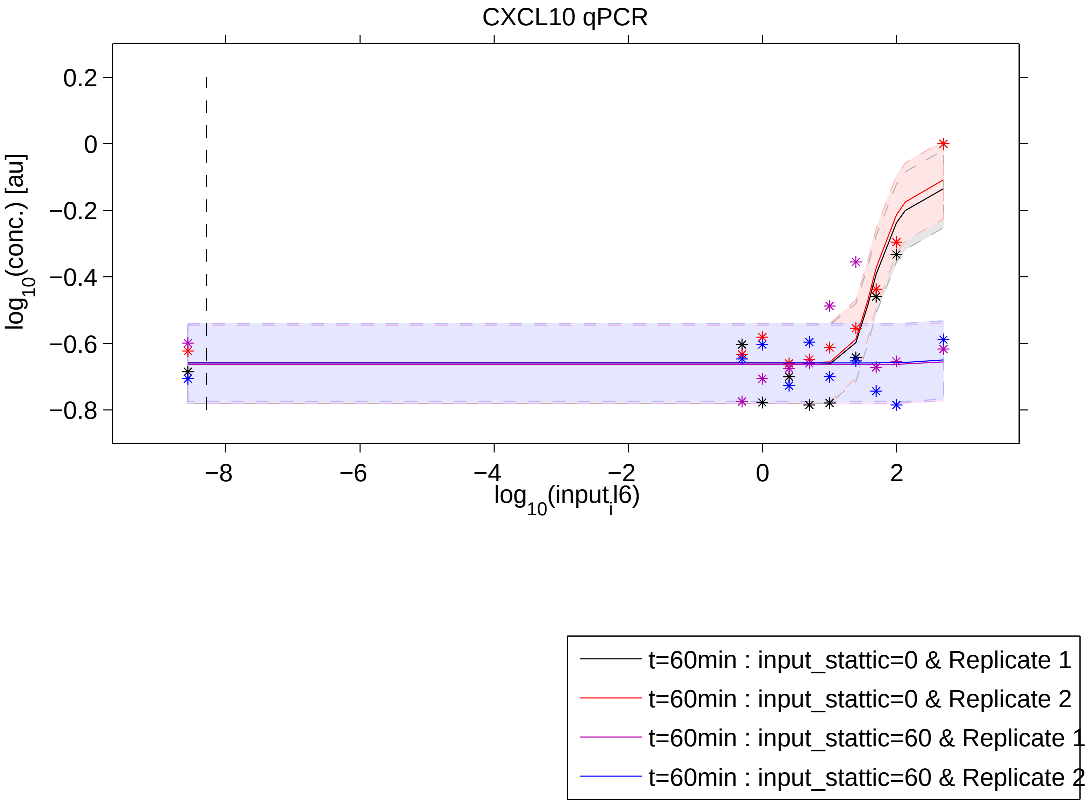

Figure S89: APP_validation_1h observables and experimental data for the experiment.

4.63.2 Condition dependent parameter changes

The following model parameters were changed to simulate these experimental conditions:

Parameter	Condition values																			
input_il6	0	0	0.5	0.5	1	1	10	10	100	100	2.5	2.5	25	25	5	5	50	50	500	500
input_stattic	0	60	0	60	0	60	0	60	0	60	0	60	0	60	0	60	0	60	0	60

Parameter	Condition values (cont.)																			
input_il6	0	0	0.5	0.5	1	1	10	10	100	100	2.5	2.5	25	25	5	5	50	50	500	500
input_stattic	0	60	0	60	0	60	0	60	0	60	0	60	0	60	0	60	0	60	0	60

Table S77: Model parameters modified for experiment APP validation 1h. Different columns indicate different conditions.

4.64 Experiment: braun validation Ruxolitinib triple 2015 04 13 triple - treatment replicate1

4.64.1 Model fit and plots

The model observables and the experimental data is shown in Figure S90. This experiment requires a custom input function which is defined in Table S78. The 4 necessary parameter transformations are listed in Table S79.

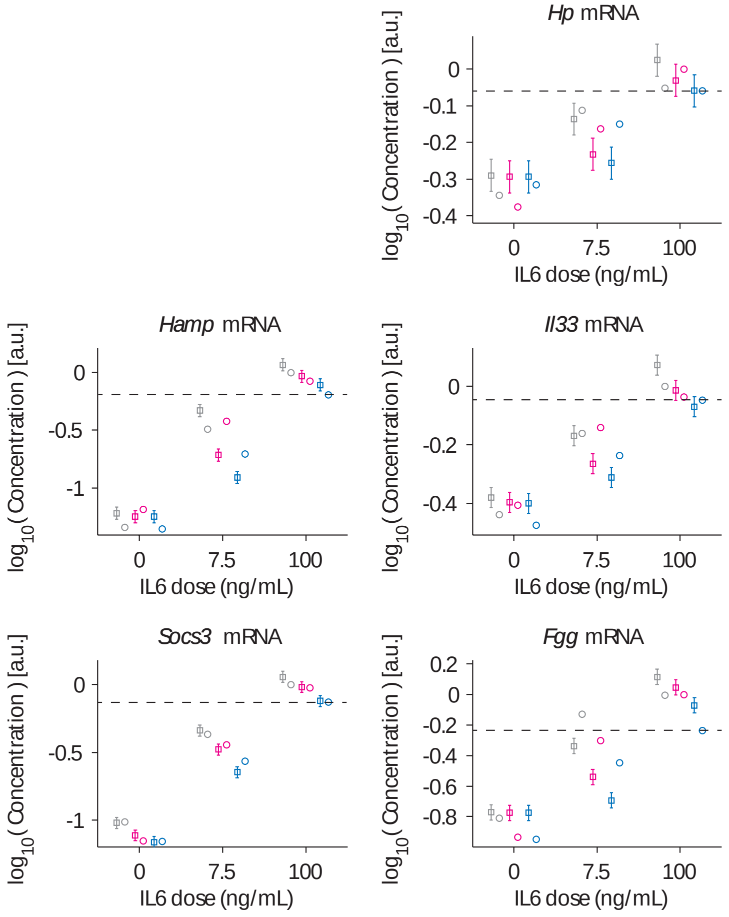

Figure S90: braun_validation_Ruxolitinib_triple_2015_04_13_triple_treatment_replicate1 observables and experimental data for the experiment. Acute phase protein validation data. Squares indicate model predictions with the error bars indicating the estimated errors. Circles indicate data points. Note that all dynamic model parameters were held fixed when fitting this data.

Input	Unit	Modified equation
Ruxolitinib	conc. [nmol/l]	$\mathrm{input\_rux1} \cdot \mathrm{heaviside}(t - \mathrm{loc1}) \cdot e^{\mathrm{rux\_degrade} \cdot (\mathrm{loc1} - t)}$ $+\mathrm{input\_rux2} \cdot \mathrm{heaviside}(t - \mathrm{loc1} - 480) \cdot e^{\mathrm{rux\_degrade} \cdot (\mathrm{loc1} - t + 480)}$ $+\mathrm{input\_rux3} \cdot \mathrm{heaviside}(t - \mathrm{loc1} - 960) \cdot e^{\mathrm{rux\_degrade} \cdot (\mathrm{loc1} - t + 960)}$

Table S78: Inputs modified for experiment braun_validation_Ruxolitinib_triple_2015_04_13_triple_treatment_replicate1

4.64.2 Condition dependent parameter changes

The following model parameters were changed to simulate these experimental conditions:

Parameter	Condition values								
input_il6	0	100	7.5	0	100	7.5	0	100	7.5
input_rux1	0	0	0	500	500	500	500	500	500
input_rux2	0	0	0	0	0	0	191	191	191
input_rux3	0	0	0	0	0	0	191	191	191
loc1	0	0	0	−60	−60	−60	0	0	0

Table S79: Model parameters modified for experiment braun validation Ruxolitinib triple 2015 04 13 triple treatment replicate1. Different columns indicate different conditions.

4.65 Experiment: braun validation Ruxolitinib triple 2015 04 13 triple - treatment replicate2

4.65.1 Model fit and plots

The model observables and the experimental data is shown in Figure S91. This experiment requires a custom input function which is defined in Table S80. The 4 necessary parameter transformations are listed in Table S81.

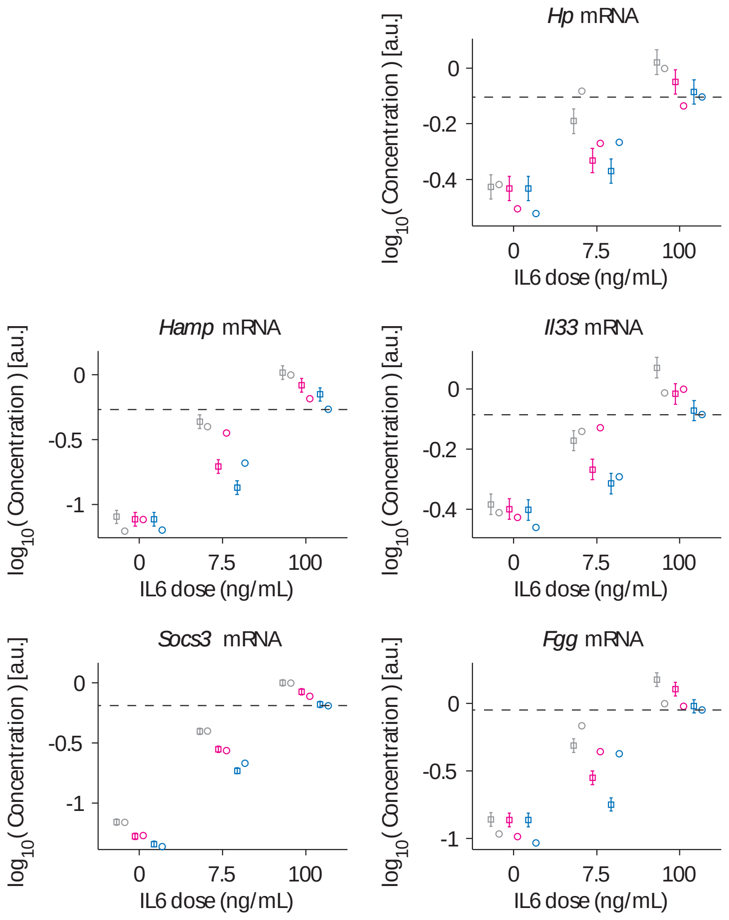

Figure S91: braun_validation_Ruxolitinib_triple_2015_04_13_triple_treatment_replicate2 observables and experimental data for the experiment. Acute phase protein validation data. Squares indicate model predictions with the error bars indicating the estimated errors. Circles indicate data points. Note that all dynamic model parameters were held fixed when fitting this data.

Input	Unit	Modified equation
Ruxolitinib	conc. [nmol/l]	$\mathrm{input\_rux1} \cdot \mathrm{heaviside}(t - \mathrm{loc1}) \cdot e^{\mathrm{rux\_degrade} \cdot (\mathrm{loc1} - t)}$ $+ \mathrm{input\_rux2} \cdot \mathrm{heaviside}(t - \mathrm{loc1} - 480) \cdot e^{\mathrm{rux\_degrade} \cdot (\mathrm{loc1} - t + 480)}$ $+ \mathrm{input\_rux3} \cdot \mathrm{heaviside}(t - \mathrm{loc1} - 960) \cdot e^{\mathrm{rux\_degrade} \cdot (\mathrm{loc1} - t + 960)}$

Table S80: Inputs modified for experiment braun_validation_Ruxolitinib_triple_2015_04_13_triple_treatment_replicate2

4.65.2 Condition dependent parameter changes

The following model parameters were changed to simulate these experimental conditions:

Parameter	Condition values								
input_il6	0	100	7.5	0	100	7.5	0	100	7.5
input_rux1	0	0	0	500	500	500	500	500	500
input_rux2	0	0	0	0	0	0	191	191	191
input_rux3	0	0	0	0	0	0	191	191	191
loc1	0	0	0	−60	−60	−60	0	0	0

Table S81: Model parameters modified for experiment braun validation Ruxolitinib triple 2015 04 13 triple treatment replicate2. Different columns indicate different conditions.

4.66 Experiment: braun validation Ruxolitinib triple 2015 04 13 triple - treatment replicate3

4.66.1 Model fit and plots

The model observables and the experimental data is shown in Figure S92. This experiment requires a custom input function which is defined in Table S82. The 4 necessary parameter transformations are listed in Table S83.

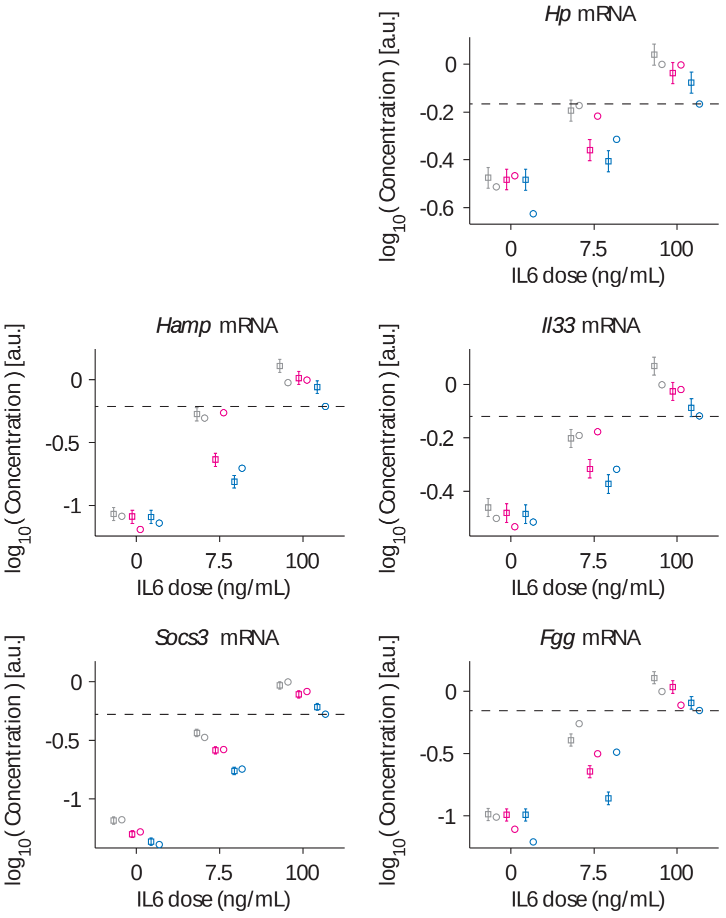

Figure S92: braun_validation_Ruxolitinib_triple_2015_04_13_triple_treatment_replicate3 observables and experimental data for the experiment. Acute phase protein validation data. Squares indicate model predictions with the error bars indicating the estimated errors. Circles indicate data points. Note that all dynamic model parameters were held fixed when fitting this data.

Input	Unit	Modified equation
Ruxolitinib	conc. [nmol/l]	$\mathrm{input\_rux1} \cdot \mathrm{heaviside}(t - \mathrm{loc1}) \cdot e^{\mathrm{rux\_degrade} \cdot (\mathrm{loc1} - t)}$ $+ \mathrm{input\_rux2} \cdot \mathrm{heaviside}(t - \mathrm{loc1} - 480) \cdot e^{\mathrm{rux\_degrade} \cdot (\mathrm{loc1} - t + 480)}$ $+ \mathrm{input\_rux3} \cdot \mathrm{heaviside}(t - \mathrm{loc1} - 960) \cdot e^{\mathrm{rux\_degrade} \cdot (\mathrm{loc1} - t + 960)}$

Table S82: Inputs modified for experiment braun_validation_Ruxolitinib_triple_2015_04_13_triple_treatment_replicate3

4.66.2 Condition dependent parameter changes

The following model parameters were changed to simulate these experimental conditions:

Parameter	Condition values								
input_il6	0	100	7.5	0	100	7.5	0	100	7.5
input_rux1	0	0	0	500	500	500	500	500	500
input_rux2	0	0	0	0	0	0	191	191	191
input_rux3	0	0	0	0	0	0	191	191	191
loc1	0	0	0	−60	−60	−60	0	0	0

Table S83: Model parameters modified for experiment braun validation Ruxolitinib triple 2015 04 13 triple treatment replicate3. Different columns indicate different conditions.

4.67 Experiment: xiaoyun validation nucSTAT3 validation 20150428 nExpID1

4.67.1 Model fit and plots

The model observables and the experimental data is shown in Figure S93. This experiment requires a custom input function which is defined in Table S84. The 5 necessary parameter transformations are listed in Table S86.

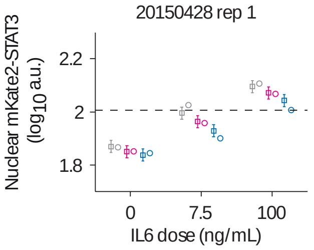

Figure S93: xiaoyun_validation_nucSTAT3_validation_20150428_nExpID1 observables and experimental data for the experiment. Squares indicate model predictions with the error bars indicating the estimated errors. Circles indicate data points. Note that all dynamic model parameters were held fixed when fitting this data.

Input	Unit	Modified equation
Ruxolitinib	conc. [nmol/l]	$\text{input\_rux1} \cdot \text{heaviside}(t - \text{loc1}) \cdot e^{\text{rux\_degrade}\cdot(\text{loc1}-t)}$
		$+\text{input\_rux2} \cdot \text{heaviside}(t - \text{loc1} - 480) \cdot e^{\text{rux\_degrade}\cdot(\text{loc1}-t+480)}$
		$+\text{input\_rux2} \cdot \text{heaviside}(t - \text{loc1} - 960) \cdot e^{\text{rux\_degrade}\cdot(\text{loc1}-t+960)}$

Table S84: Inputs modified for experiment xiaoyun_validation_nucSTAT3_validation_20150428_nExpID1

4.67.2 Observables

The following observables are added in this data set:

Observable		Equations
nucSTAT3 [au]	y	$\log_{10}(\text{offset\_ntstat3\_71} + [\text{ntSTAT3}] \cdot \text{scale\_ntstat3\_71})$
	σ	sd_ntstat3_validation_xiaoyun_validation_nucSTAT3_validation_20150428_nExpID1

Table S85: Observables added for experiment xiaoyun_validation_nucSTAT3_validation_20150428_nExpID1

4.67.3 Condition dependent parameter changes

The following model parameters were changed to simulate these experimental conditions:

Parameter	Condition values											
DMSO	1	1	0	0	1	1	0	0	1	1	0	0
input_il6	0.0	0.0	0.0	0.0	100.0	100.0	100.0	100.0	7.5	7.5	7.5	7.5
input_rux1	0	0	500	500	0	0	500	500	0	0	500	500
input_rux2	0.0	0.0	0.0	191	0.0	0.0	0.0	191	0.0	0.0	0.0	191
isTriple	0	1	0	1	0	1	0	1	0	1	0	1
loc1	−60	0	−60	0	−60	0	−60	0	−60	0	−60	0

Table S86: Model parameters modified for experiment xiaoyun validation nucSTAT3 validation 20150428 nExpID1. Different columns indicate different conditions.

4.68 Experiment: xiaoyun validation nucSTAT3 validation 20150428 nExpID2

4.68.1 Model fit and plots

The model observables and the experimental data is shown in Figure S94. This experiment requires a custom input function which is defined in Table S87. The 5 necessary parameter transformations are listed in Table S89.

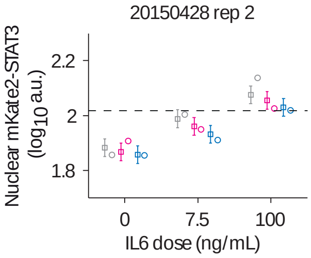

Figure S94: xiaoyun_validation_nucSTAT3_validation_20150428_nExpID2 observables and experimental data for the experiment. Squares indicate model predictions with the error bars indicating the estimated errors. Circles indicate data points. Note that all dynamic model parameters were held fixed when fitting this data.

Input	Unit	Modified equation
Ruxolitinib	conc. [nmol/l]	$\text{input\_rux1} \cdot \text{heaviside}(\text{t} - \text{loc1}) \cdot e^{\text{rux\_degrade} \cdot (\text{loc1} - \text{t})}$
		$+\text{input\_rux2} \cdot \text{heaviside}(\text{t} - \text{loc1} - 480) \cdot e^{\text{rux\_degrade} \cdot (\text{loc1} - \text{t} + 480)}$
		$+\text{input\_rux2} \cdot \text{heaviside}(\text{t} - \text{loc1} - 960) \cdot e^{\text{rux\_degrade} \cdot (\text{loc1} - \text{t} + 960)}$

Table S87: Inputs modified for experiment xiaoyun_validation_nucSTAT3_validation_20150428_nExpID2

4.68.2 Observables

The following observables are added in this data set:

Observable		Equations
nucSTAT3 [au]	y	$\log_{10}(\text{offset\_ntstat3\_72} + [\text{ntSTAT3}] \cdot \text{scale\_ntstat3\_72})$
	σ	sd_ntstat3_validation_xiaoyun_validation_nucSTAT3_validation_20150428_nExpID2

Table S88: Observables added for experiment xiaoyun_validation_nucSTAT3_validation_20150428_nExpID2

4.68.3 Condition dependent parameter changes

The following model parameters were changed to simulate these experimental conditions:

Parameter	Condition values											
DMSO	1	1	0	0	1	1	0	0	1	1	0	0
input_il6	0.0	0.0	0.0	0.0	100.0	100.0	100.0	100.0	7.5	7.5	7.5	7.5
input_rux1	0	0	500	500	0	0	500	500	0	0	500	500
input_rux2	0.0	0.0	0.0	191	0.0	0.0	0.0	191	0.0	0.0	0.0	191
isTriple	0	1	0	1	0	1	0	1	0	1	0	1
loc1	−60	0	−60	0	−60	0	−60	0	−60	0	−60	0

Table S89: Model parameters modified for experiment xiaoyun validation nucSTAT3 validation 20150428 nExpID2. Different columns indicate different conditions.

4.69 Experiment: xiaoyun validation nucSTAT3 validation 20150508 nExpID1

4.69.1 Model fit and plots

The model observables and the experimental data is shown in Figure S95. This experiment requires a custom input function which is defined in Table S90. The 5 necessary parameter transformations are listed in Table S92.

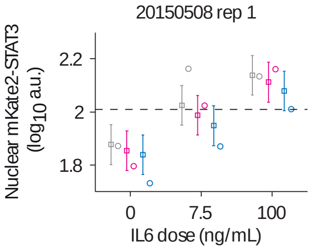

Figure S95: xiaoyun_validation_nucSTAT3_validation_20150508_nExpID1 observables and experimental data for the experiment. Squares indicate model predictions with the error bars indicating the estimated errors. Circles indicate data points. Note that all dynamic model parameters were held fixed when fitting this data.

Input	Unit	Modified equation
Ruxolitinib	conc. [nmol/l]	$\text{input\_rux1} \cdot \text{heaviside}(t - \text{loc1}) \cdot e^{\text{rux\_degrade} \cdot (\text{loc1} - t)}$
		$+\text{input\_rux2} \cdot \text{heaviside}(t - \text{loc1} - 480) \cdot e^{\text{rux\_degrade} \cdot (\text{loc1} - t + 480)}$
		$+\text{input\_rux2} \cdot \text{heaviside}(t - \text{loc1} - 960) \cdot e^{\text{rux\_degrade} \cdot (\text{loc1} - t + 960)}$

Table S90: Inputs modified for experiment xiaoyun_validation_nucSTAT3_validation_20150508_nExpID1

4.69.2 Observables

The following observables are added in this data set:

Observable		Equations
nucSTAT3 [au]	y	$\log_{10}(\text{offset\_ntstat3\_73} + [\text{ntSTAT3}] \cdot \text{scale\_ntstat3\_73})$
	σ	sd_ntstat3_validation_xiaoyun_validation_nucSTAT3_validation_20150508_nExpID1

Table S91: Observables added for experiment xiaoyun_validation_nucSTAT3_validation_20150508_nExpID1

4.69.3 Condition dependent parameter changes

The following model parameters were changed to simulate these experimental conditions:

Parameter	Condition values											
DMSO	1	1	0	0	1	1	0	0	1	1	0	0
input_il6	0.0	0.0	0.0	0.0	100.0	100.0	100.0	100.0	7.5	7.5	7.5	7.5
input_rux1	0	0	500	500	0	0	500	500	0	0	500	500
input_rux2	0.0	0.0	0.0	191	0.0	0.0	0.0	191	0.0	0.0	0.0	191
isTriple	0	1	0	1	0	1	0	1	0	1	0	1
loc1	−60	0	−60	0	−60	0	−60	0	−60	0	−60	0

Table S92: Model parameters modified for experiment xiaoyun validation nucSTAT3 validation 20150508 nExpID1. Different columns indicate different conditions.

4.70 Experiment: xiaoyun validation nucSTAT3 validation 20150508 nExpID2

4.70.1 Model fit and plots

The model observables and the experimental data is shown in Figure S96. This experiment requires a custom input function which is defined in Table S93. The 5 necessary parameter transformations are listed in Table S95.

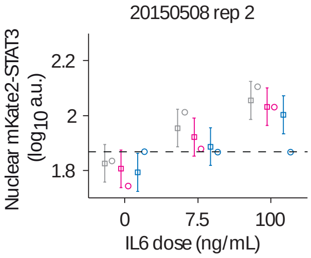

Figure S96: xiaoyun_validation_nucSTAT3_validation_20150508_nExpID2 observables and experimental data for the experiment. Squares indicate model predictions with the error bars indicating the estimated errors. Circles indicate data points. Note that all dynamic model parameters were held fixed when fitting this data.

Input	Unit	Modified equation
Ruxolitinib	conc. [nmol/l]	$\text{input\_rux1} \cdot \text{heaviside}(t - \text{loc1}) \cdot e^{\text{rux\_degrade} \cdot (\text{loc1} - t)}$
		$+\text{input\_rux2} \cdot \text{heaviside}(t - \text{loc1} - 480) \cdot e^{\text{rux\_degrade} \cdot (\text{loc1} - t + 480)}$
		$+\text{input\_rux2} \cdot \text{heaviside}(t - \text{loc1} - 960) \cdot e^{\text{rux\_degrade} \cdot (\text{loc1} - t + 960)}$

Table S93: Inputs modified for experiment xiaoyun_validation_nucSTAT3_validation_20150508_nExpID2

4.70.2 Observables

The following observables are added in this data set:

Observable		Equations
nucSTAT3 [au]	y	$\log_{10}(\text{offset\_ntstat3\_74} + [\text{ntSTAT3}] \cdot \text{scale\_ntstat3\_74})$
	σ	sd_ntstat3_validation_xiaoyun_validation_nucSTAT3_validation_20150508_nExpID2

Table S94: Observables added for experiment xiaoyun_validation_nucSTAT3_validation_20150508_nExpID2

4.70.3 Condition dependent parameter changes

The following model parameters were changed to simulate these experimental conditions:

Parameter	Condition values											
DMSO	1	1	0	0	1	1	0	0	1	1	0	0
input_il6	0.0	0.0	0.0	0.0	100.0	100.0	100.0	100.0	7.5	7.5	7.5	7.5
input_rux1	0	0	500	500	0	0	500	500	0	0	500	500
input_rux2	0.0	0.0	0.0	191	0.0	0.0	0.0	191	0.0	0.0	0.0	191
isTriple	0	1	0	1	0	1	0	1	0	1	0	1
loc1	−60	0	−60	0	−60	0	−60	0	−60	0	−60	0

Table S95: Model parameters modified for experiment xiaoyun validation nucSTAT3 validation 20150508 nExpID2. Different columns indicate different conditions.

4.71 Experiment: xiaoyun validation nucSTAT3 validation 20150528 nExpID1

4.71.1 Model fit and plots

The model observables and the experimental data is shown in Figure S97. This experiment requires a custom input function which is defined in Table S96. The 5 necessary parameter transformations are listed in Table S98.

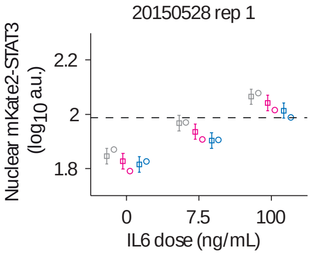

Figure S97: xiaoyun_validation_nucSTAT3_validation_20150528_nExpID1 observables and experimental data for the experiment. Squares indicate model predictions with the error bars indicating the estimated errors. Circles indicate data points. Note that all dynamic model parameters were held fixed when fitting this data.

Input	Unit	Modified equation
Ruxolitinib	conc. [nmol/l]	$\text{input\_rux1} \cdot \text{heaviside}(t - \text{loc1}) \cdot e^{\text{rux\_degrade}\cdot(\text{loc1}-t)}$ $+\text{input\_rux2} \cdot \text{heaviside}(t - \text{loc1} - 480) \cdot e^{\text{rux\_degrade}\cdot(\text{loc1}-t+480)}$ $+\text{input\_rux2} \cdot \text{heaviside}(t - \text{loc1} - 960) \cdot e^{\text{rux\_degrade}\cdot(\text{loc1}-t+960)}$

Table S96: Inputs modified for experiment xiaoyun_validation_nucSTAT3_validation_20150528_nExpID1

4.71.2 Observables

The following observables are added in this data set:

Observable		Equations
nucSTAT3 [au]	y	$\log_{10}(\text{offset\_ntstat3\_75} + [\text{ntSTAT3}] \cdot \text{scale\_ntstat3\_75})$
	σ	sd_ntstat3_validation_xiaoyun_validation_nucSTAT3_validation_20150528_nExpID1

Table S97: Observables added for experiment xiaoyun_validation_nucSTAT3_validation_20150528_nExpID1

4.71.3 Condition dependent parameter changes

The following model parameters were changed to simulate these experimental conditions:

Parameter	Condition values											
DMSO	1	1	0	0	1	1	0	0	1	1	0	0
input_il6	0.0	0.0	0.0	0.0	100.0	100.0	100.0	100.0	7.5	7.5	7.5	7.5
input_rux1	0	0	500	500	0	0	500	500	0	0	500	500
input_rux2	0.0	0.0	0.0	191	0.0	0.0	0.0	191	0.0	0.0	0.0	191
isTriple	0	1	0	1	0	1	0	1	0	1	0	1
loc1	−60	0	−60	0	−60	0	−60	0	−60	0	−60	0

Table S98: Model parameters modified for experiment xiaoyun validation nucSTAT3 validation 20150528 nExpID1. Different columns indicate different conditions.

4.72 Experiment: xiaoyun validation nucSTAT3 validation 20150528 nExpID2

4.72.1 Model fit and plots

The model observables and the experimental data is shown in Figure S98. This experiment requires a custom input function which is defined in Table S100. The 5 necessary parameter transformations are listed in Table S101.

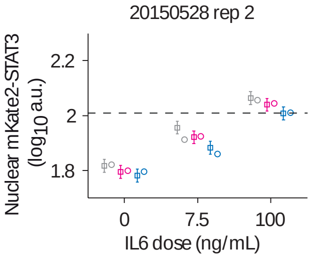

Figure S98: xiaoyun_validation_nucSTAT3_validation_20150528_nExpID2 observables and experimental data for the experiment. Squares indicate model predictions with the error bars indicating the estimated errors. Circles indicate data points. Note that all dynamic model parameters were held fixed when fitting this data.

Input	Unit	Modified equation
Ruxolitinib	conc. [nmol/l]	$\mathrm{input\_rux1} \cdot \mathrm{heaviside}(t - \mathrm{loc1}) \cdot e^{\mathrm{rux\_degrade} \cdot (\mathrm{loc1} - t)}$
		$+\mathrm{input\_rux2} \cdot \mathrm{heaviside}(t - \mathrm{loc1} - 480) \cdot e^{\mathrm{rux\_degrade} \cdot (\mathrm{loc1} - t + 480)}$
		$+\mathrm{input\_rux2} \cdot \mathrm{heaviside}(t - \mathrm{loc1} - 960) \cdot e^{\mathrm{rux\_degrade} \cdot (\mathrm{loc1} - t + 960)}$

Table S99: Inputs modified for experiment xiaoyun_validation_nucSTAT3_validation_20150528_nExpID2

4.72.2 Observables

The following observables are added in this data set:

Observable		Equations
nucSTAT3 [au]	y	$\log_{10}(\mathrm{offset\_ntstat3\_76} + [\mathrm{ntSTAT3}] \cdot \mathrm{scale\_ntstat3\_76})$
	σ	sd_ntstat3_validation_xiaoyun_validation_nucSTAT3_validation_20150528_nExpID2

Table S100: Observables added for experiment xiaoyun_validation_nucSTAT3_validation_20150528_nExpID2

4.72.3 Condition dependent parameter changes

The following model parameters were changed to simulate these experimental conditions:

Parameter	Condition values											
DMSO	1	1	0	0	1	1	0	0	1	1	0	0
input_il6	0.0	0.0	0.0	0.0	100.0	100.0	100.0	100.0	7.5	7.5	7.5	7.5
input_rux1	0	0	500	500	0	0	500	500	0	0	500	500
input_rux2	0.0	0.0	0.0	191	0.0	0.0	0.0	191	0.0	0.0	0.0	191
isTriple	0	1	0	1	0	1	0	1	0	1	0	1
loc1	−60	0	−60	0	−60	0	−60	0	−60	0	−60	0

Table S101: Model parameters modified for experiment xiaoyun validation nucSTAT3 validation 20150528 nExpID2. Different columns indicate different conditions.

5 Estimated model parameters

This section lists all the model parameters used in the model. Parameters highlighted in red color indicate parameter values close to their bounds. The parameter name prefix offset_ indicates a offset of the experimental data. The parameter name prefix scale_ indicates a scaling factor of the experimental data. The parameter name prefix sd_ indicates the magnitude of the measurement noise for a specific measurement.

	name	θ_{min}	$\hat{\theta}$	θ_{max}	log	non-log $\hat{\theta}$	fitted
1	APCS_knot10_1	-5	-2.0209	+2	1	$+9.53 \cdot 10^{-03}$	1
2	APCS_knot10_2	-5	-2.7534	+2	1	$+1.76 \cdot 10^{-03}$	1
3	APCS_knot1_1	-5	-0.1720	+2	1	$+6.73 \cdot 10^{-01}$	1
4	APCS_knot1_2	-5	+0.0842	+2	1	$+1.21 \cdot 10^{+00}$	1
5	APCS_knot2_1	-5	-0.1720	+2	1	$+6.73 \cdot 10^{-01}$	1
6	APCS_knot2_2	-5	+0.0842	+2	1	$+1.21 \cdot 10^{+00}$	1
7	APCS_knot3_1	-5	-0.0318	+2	1	$+9.29 \cdot 10^{-01}$	1
8	APCS_knot3_2	-5	+0.1338	+2	1	$+1.36 \cdot 10^{+00}$	1
9	APCS_knot4_1	-5	-0.2132	+2	1	$+6.12 \cdot 10^{-01}$	1
10	APCS_knot4_2	-5	+0.0071	+2	1	$+1.02 \cdot 10^{+00}$	1
11	APCS_knot5_1	-5	-0.2188	+2	1	$+6.04 \cdot 10^{-01}$	1
12	APCS_knot5_2	-5	-0.0000	+2	1	$+1.00 \cdot 10^{+00}$	1
13	APCS_knot6_1	-5	-0.5040	+2	1	$+3.13 \cdot 10^{-01}$	1
14	APCS_knot6_2	-5	-0.2243	+2	1	$+5.97 \cdot 10^{-01}$	1
15	APCS_knot7_1	-5	-2.0209	+2	1	$+9.53 \cdot 10^{-03}$	1
16	APCS_knot7_2	-5	-2.7534	+2	1	$+1.76 \cdot 10^{-03}$	1
17	APCS_knot8_1	-5	-2.0209	+2	1	$+9.53 \cdot 10^{-03}$	1
18	APCS_knot8_2	-5	-2.7534	+2	1	$+1.76 \cdot 10^{-03}$	1
19	APCS_knot9_1	-5	-2.0209	+2	1	$+9.53 \cdot 10^{-03}$	1
20	APCS_knot9_2	-5	-2.7534	+2	1	$+1.76 \cdot 10^{-03}$	1
21	CXCL10_knot10_1	-5	-1.3501	+2	1	$+4.47 \cdot 10^{-02}$	1
22	CXCL10_knot10_2	-5	-1.7185	+2	1	$+1.91 \cdot 10^{-02}$	1
23	CXCL10_knot1_1	-5	-1.1619	+2	1	$+6.89 \cdot 10^{-02}$	1
24	CXCL10_knot1_2	-5	-0.9743	+2	1	$+1.06 \cdot 10^{-01}$	1
25	CXCL10_knot2_1	-5	-1.1619	+2	1	$+6.89 \cdot 10^{-02}$	1
26	CXCL10_knot2_2	-5	-0.9743	+2	1	$+1.06 \cdot 10^{-01}$	1
27	CXCL10_knot3_1	-5	-1.1092	+2	1	$+7.78 \cdot 10^{-02}$	1
28	CXCL10_knot3_2	-5	-0.8627	+2	1	$+1.37 \cdot 10^{-01}$	1
29	CXCL10_knot4_1	-5	-0.6646	+2	1	$+2.16 \cdot 10^{-01}$	1
30	CXCL10_knot4_2	-5	-0.7917	+2	1	$+1.62 \cdot 10^{-01}$	1
31	CXCL10_knot5_1	-5	-0.9833	+2	1	$+1.04 \cdot 10^{-01}$	1
32	CXCL10_knot5_2	-5	-0.8556	+2	1	$+1.39 \cdot 10^{-01}$	1
33	CXCL10_knot6_1	-5	-0.6329	+2	1	$+2.33 \cdot 10^{-01}$	1
34	CXCL10_knot6_2	-5	-0.8687	+2	1	$+1.35 \cdot 10^{-01}$	1
35	CXCL10_knot7_1	-5	-1.3501	+2	1	$+4.47 \cdot 10^{-02}$	1
36	CXCL10_knot7_2	-5	-1.7185	+2	1	$+1.91 \cdot 10^{-02}$	1
37	CXCL10_knot8_1	-5	-1.3501	+2	1	$+4.47 \cdot 10^{-02}$	1
38	CXCL10_knot8_2	-5	-1.7185	+2	1	$+1.91 \cdot 10^{-02}$	1
39	CXCL10_knot9_1	-5	-1.3501	+2	1	$+4.47 \cdot 10^{-02}$	1
40	CXCL10_knot9_2	-5	-1.7185	+2	1	$+1.91 \cdot 10^{-02}$	1
41	FGG_knot10_1	-5	-0.5832	+2	1	$+2.61 \cdot 10^{-01}$	1
42	FGG_knot10_2	-5	-3.6178	+2	1	$+2.41 \cdot 10^{-04}$	1
43	FGG_knot1_1	-5	-0.3159	+2	1	$+4.83 \cdot 10^{-01}$	1
44	FGG_knot1_2	-5	-0.4823	+2	1	$+3.29 \cdot 10^{-01}$	1

Table S102: Estimated parameter values
$\hat{\theta}$ indicates the estimated value of the parameters. θ_{min} and θ_{max} indicate the upper and lower bounds for the parameters. The log-column indicates if the value of a parameter was log-transformed. If log = 1 the non-log-column indicates the non-logarithmic value of the estimate. The fitted-column indicates if the parameter value was estimated (1), was temporarily fixed (0) or if its value was fixed to a constant value (2).

	name	θ_{min}	$\hat{\theta}$	θ_{max}	log	non-log $\hat{\theta}$	fitted
45	FGG_knot2_1	-5	-0.3159	+2	1	$+4.83 \cdot 10^{-01}$	1
46	FGG_knot2_2	-5	-0.4823	+2	1	$+3.29 \cdot 10^{-01}$	1
47	FGG_knot3_1	-5	-0.6616	+2	1	$+2.18 \cdot 10^{-01}$	1
48	FGG_knot3_2	-5	-1.1557	+2	1	$+6.99 \cdot 10^{-02}$	1
49	FGG_knot4_1	-5	-0.2179	+2	1	$+6.05 \cdot 10^{-01}$	1
50	FGG_knot4_2	-5	-0.2987	+2	1	$+5.03 \cdot 10^{-01}$	1
51	FGG_knot5_1	-5	-0.6990	+2	1	$+2.00 \cdot 10^{-01}$	1
52	FGG_knot5_2	-5	-1.1218	+2	1	$+7.55 \cdot 10^{-02}$	1
53	FGG_knot6_1	-5	-0.6709	+2	1	$+2.13 \cdot 10^{-01}$	1
54	FGG_knot6_2	-5	-0.6755	+2	1	$+2.11 \cdot 10^{-01}$	1
55	FGG_knot7_1	-5	-0.5832	+2	1	$+2.61 \cdot 10^{-01}$	1
56	FGG_knot7_2	-5	-3.6179	+2	1	$+2.41 \cdot 10^{-04}$	1
57	FGG_knot8_1	-5	-0.5832	+2	1	$+2.61 \cdot 10^{-01}$	1
58	FGG_knot8_2	-5	-3.6178	+2	1	$+2.41 \cdot 10^{-04}$	1
59	FGG_knot9_1	-5	-0.5832	+2	1	$+2.61 \cdot 10^{-01}$	1
60	FGG_knot9_2	-5	-3.6178	+2	1	$+2.41 \cdot 10^{-04}$	1
61	HAMP_knot10_1	-5	-0.4448	+2	1	$+3.59 \cdot 10^{-01}$	1
62	HAMP_knot10_2	-5	-0.1635	+2	1	$+6.86 \cdot 10^{-01}$	1
63	HAMP_knot1_1	-5	-0.7518	+2	1	$+1.77 \cdot 10^{-01}$	1
64	HAMP_knot1_2	-5	-0.8343	+2	1	$+1.46 \cdot 10^{-01}$	1
65	HAMP_knot2_1	-5	-0.7514	+2	1	$+1.77 \cdot 10^{-01}$	1
66	HAMP_knot2_2	-5	-0.8338	+2	1	$+1.47 \cdot 10^{-01}$	1
67	HAMP_knot3_1	-5	-1.2741	+2	1	$+5.32 \cdot 10^{-02}$	1
68	HAMP_knot3_2	-5	-0.6863	+2	1	$+2.06 \cdot 10^{-01}$	1
69	HAMP_knot4_1	-5	-0.5952	+2	1	$+2.54 \cdot 10^{-01}$	1
70	HAMP_knot4_2	-5	-0.7507	+2	1	$+1.78 \cdot 10^{-01}$	1
71	HAMP_knot5_1	-5	-1.1967	+2	1	$+6.36 \cdot 10^{-02}$	1
72	HAMP_knot5_2	-5	-1.7991	+2	1	$+1.59 \cdot 10^{-02}$	1
73	HAMP_knot6_1	-5	-1.4703	+2	1	$+3.39 \cdot 10^{-02}$	1
74	HAMP_knot6_2	-5	-0.9315	+2	1	$+1.17 \cdot 10^{-01}$	1
75	HAMP_knot7_1	-5	-0.4446	+2	1	$+3.59 \cdot 10^{-01}$	1
76	HAMP_knot7_2	-5	-0.1634	+2	1	$+6.87 \cdot 10^{-01}$	1
77	HAMP_knot8_1	-5	-0.4448	+2	1	$+3.59 \cdot 10^{-01}$	1
78	HAMP_knot8_2	-5	-0.1635	+2	1	$+6.86 \cdot 10^{-01}$	1
79	HAMP_knot9_1	-5	-0.4448	+2	1	$+3.59 \cdot 10^{-01}$	1
80	HAMP_knot9_2	-5	-0.1635	+2	1	$+6.86 \cdot 10^{-01}$	1
81	HPX_knot10_1	-5	-0.2129	+2	1	$+6.12 \cdot 10^{-01}$	1
82	HPX_knot10_2	-5	-0.0320	+2	1	$+9.29 \cdot 10^{-01}$	1
83	HPX_knot1_1	-5	-0.5849	+2	1	$+2.60 \cdot 10^{-01}$	1
84	HPX_knot1_2	-5	-0.0160	+2	1	$+9.64 \cdot 10^{-01}$	1
85	HPX_knot2_1	-5	-0.5849	+2	1	$+2.60 \cdot 10^{-01}$	1
86	HPX_knot2_2	-5	-0.0159	+2	1	$+9.64 \cdot 10^{-01}$	1
87	HPX_knot3_1	-5	-1.1217	+2	1	$+7.56 \cdot 10^{-02}$	1
88	HPX_knot3_2	-5	-0.1840	+2	1	$+6.55 \cdot 10^{-01}$	1
89	HPX_knot4_1	-5	-2.6431	+2	1	$+2.27 \cdot 10^{-03}$	1

Table S103: Estimated parameter values
$\hat{\theta}$ indicates the estimated value of the parameters. θ_{min} and θ_{max} indicate the upper and lower bounds for the parameters. The log-column indicates if the value of a parameter was log-transformed. If log = 1 the non-log-column indicates the non-logarithmic value of the estimate. The fitted-column indicates if the parameter value was estimated (1), was temporarily fixed (0) or if its value was fixed to a constant value (2).

	name	θ_{min}	$\hat{\theta}$	θ_{max}	log	non-log $\hat{\theta}$	fitted
90	HPX_knot4_2	-5	-0.0458	+2	1	$+9.00 \cdot 10^{-01}$	1
91	HPX_knot5_1	-5	-0.7315	+2	1	$+1.86 \cdot 10^{-01}$	1
92	HPX_knot5_2	-5	-0.1486	+2	1	$+7.10 \cdot 10^{-01}$	1
93	HPX_knot6_1	-5	-0.4294	+2	1	$+3.72 \cdot 10^{-01}$	1
94	HPX_knot6_2	-5	-0.0074	+2	1	$+9.83 \cdot 10^{-01}$	1
95	HPX_knot7_1	-5	-0.2129	+2	1	$+6.12 \cdot 10^{-01}$	1
96	HPX_knot7_2	-5	-0.0319	+2	1	$+9.29 \cdot 10^{-01}$	1
97	HPX_knot8_1	-5	-0.2129	+2	1	$+6.12 \cdot 10^{-01}$	1
98	HPX_knot8_2	-5	-0.0320	+2	1	$+9.29 \cdot 10^{-01}$	1
99	HPX_knot9_1	-5	-0.2129	+2	1	$+6.12 \cdot 10^{-01}$	1
100	HPX_knot9_2	-5	-0.0320	+2	1	$+9.29 \cdot 10^{-01}$	1
101	HP_knot10_1	-5	+0.2034	+2	1	$+1.60 \cdot 10^{+00}$	1
102	HP_knot10_2	-5	-0.1200	+2	1	$+7.59 \cdot 10^{-01}$	1
103	HP_knot1_1	-5	-0.3453	+2	1	$+4.52 \cdot 10^{-01}$	1
104	HP_knot1_2	-5	-0.2668	+2	1	$+5.41 \cdot 10^{-01}$	1
105	HP_knot2_1	-5	-0.3474	+2	1	$+4.49 \cdot 10^{-01}$	1
106	HP_knot2_2	-5	-0.2656	+2	1	$+5.42 \cdot 10^{-01}$	1
107	HP_knot3_1	-5	-0.5332	+2	1	$+2.93 \cdot 10^{-01}$	1
108	HP_knot3_2	-5	-0.3499	+2	1	$+4.47 \cdot 10^{-01}$	1
109	HP_knot4_1	-5	-0.4214	+2	1	$+3.79 \cdot 10^{-01}$	1
110	HP_knot4_2	-5	-0.2664	+2	1	$+5.42 \cdot 10^{-01}$	1
111	HP_knot5_1	-5	-0.2700	+2	1	$+5.37 \cdot 10^{-01}$	1
112	HP_knot5_2	-5	-0.3468	+2	1	$+4.50 \cdot 10^{-01}$	1
113	HP_knot6_1	-5	-0.0144	+2	1	$+9.67 \cdot 10^{-01}$	1
114	HP_knot6_2	-5	-0.2181	+2	1	$+6.05 \cdot 10^{-01}$	1
115	HP_knot7_1	-5	+0.2037	+2	1	$+1.60 \cdot 10^{+00}$	1
116	HP_knot7_2	-5	-0.1190	+2	1	$+7.60 \cdot 10^{-01}$	1
117	HP_knot8_1	-5	+0.2034	+2	1	$+1.60 \cdot 10^{+00}$	1
118	HP_knot8_2	-5	-0.1200	+2	1	$+7.59 \cdot 10^{-01}$	1
119	HP_knot9_1	-5	+0.2034	+2	1	$+1.60 \cdot 10^{+00}$	1
120	HP_knot9_2	-5	-0.1200	+2	1	$+7.59 \cdot 10^{-01}$	1
121	IL33_knot10_1	-5	+0.1596	+2	1	$+1.44 \cdot 10^{+00}$	1
122	IL33_knot10_2	-5	+0.1778	+2	1	$+1.51 \cdot 10^{+00}$	1
123	IL33_knot1_1	-5	-0.5290	+2	1	$+2.96 \cdot 10^{-01}$	1
124	IL33_knot1_2	-5	-0.4637	+2	1	$+3.44 \cdot 10^{-01}$	1
125	IL33_knot2_1	-5	-0.5294	+2	1	$+2.96 \cdot 10^{-01}$	1
126	IL33_knot2_2	-5	-0.4640	+2	1	$+3.44 \cdot 10^{-01}$	1
127	IL33_knot3_1	-5	-0.8581	+2	1	$+1.39 \cdot 10^{-01}$	1
128	IL33_knot3_2	-5	-0.4700	+2	1	$+3.39 \cdot 10^{-01}$	1
129	IL33_knot4_1	-5	-0.3491	+2	1	$+4.48 \cdot 10^{-01}$	1
130	IL33_knot4_2	-5	-0.3545	+2	1	$+4.42 \cdot 10^{-01}$	1
131	IL33_knot5_1	-5	-0.6076	+2	1	$+2.47 \cdot 10^{-01}$	1
132	IL33_knot5_2	-5	-0.5810	+2	1	$+2.62 \cdot 10^{-01}$	1
133	IL33_knot6_1	-5	-0.0172	+2	1	$+9.61 \cdot 10^{-01}$	1
134	IL33_knot6_2	-5	-0.0039	+2	1	$+9.91 \cdot 10^{-01}$	1

Table S104: Estimated parameter values
$\hat{\theta}$ indicates the estimated value of the parameters. θ_{min} and θ_{max} indicate the upper and lower bounds for the parameters. The log-column indicates if the value of a parameter was log-transformed. If log = 1 the non-log-column indicates the non-logarithmic value of the estimate. The fitted-column indicates if the parameter value was estimated (1), was temporarily fixed (0) or if its value was fixed to a constant value (2).

	name	θ_{min}	$\hat{\theta}$	θ_{max}	log	non-log $\hat{\theta}$	fitted
135	IL33_knot7_1	-5	+0.1595	+2	1	$+1.44 \cdot 10^{+00}$	1
136	IL33_knot7_2	-5	+0.1777	+2	1	$+1.51 \cdot 10^{+00}$	1
137	IL33_knot8_1	-5	+0.1596	+2	1	$+1.44 \cdot 10^{+00}$	1
138	IL33_knot8_2	-5	+0.1778	+2	1	$+1.51 \cdot 10^{+00}$	1
139	IL33_knot9_1	-5	+0.1596	+2	1	$+1.44 \cdot 10^{+00}$	1
140	IL33_knot9_2	-5	+0.1778	+2	1	$+1.51 \cdot 10^{+00}$	1
141	apcsrna_des	-5	-2.9749	+5	1	$+1.06 \cdot 10^{-03}$	1
142	apcsrna_hill	-5	+0.6266	+5	1	$+4.23 \cdot 10^{+00}$	1
143	apcsrna_ka	-5	+1.6998	+5	1	$+5.01 \cdot 10^{+01}$	1
144	apcsrna_pro	-5	-2.8555	+5	1	$+1.39 \cdot 10^{-03}$	1
145	cxcl10rna_delay	-5	-1.3446	+5	1	$+4.52 \cdot 10^{-02}$	1
146	cxcl10rna_des	-5	-1.3448	+5	1	$+4.52 \cdot 10^{-02}$	1
147	cxcl10rna_hill	-5	+0.9890	+5	1	$+9.75 \cdot 10^{+00}$	1
148	cxcl10rna_ka	-5	+2.1546	+5	1	$+1.43 \cdot 10^{+02}$	1
149	cxcl10rna_pro	-5	+0.7548	+5	1	$+5.69 \cdot 10^{+00}$	1
150	f_pstat3_imp	+0	+4.9712	+100	0	$+4.97 \cdot 10^{+00}$	1
151	fggrna_delay	-5	-0.9180	+5	1	$+1.21 \cdot 10^{-01}$	1
152	fggrna_des	-5	-2.1131	+5	1	$+7.71 \cdot 10^{-03}$	1
153	fggrna_hill	-5	+0.5952	+5	1	$+3.94 \cdot 10^{+00}$	1
154	fggrna_ka	-5	+1.6916	+5	1	$+4.92 \cdot 10^{+01}$	1
155	fggrna_pro	-5	-0.6278	+5	1	$+2.36 \cdot 10^{-01}$	1
156	gp130_act_jak1	-15	-0.2700	+6	1	$+5.37 \cdot 10^{-01}$	1
157	gp130_deg	-5	-2.4193	+3	1	$+3.81 \cdot 10^{-03}$	1
158	gp130_pro	-5	-2.6189	+3	1	$+2.40 \cdot 10^{-03}$	1
159	hamprna_des	-8	-3.6729	+8	1	$+2.12 \cdot 10^{-04}$	1
160	hamprna_hill	-8	+0.5128	+8	1	$+3.26 \cdot 10^{+00}$	1
161	hamprna_ka	-8	+1.6750	+8	1	$+4.73 \cdot 10^{+01}$	1
162	hamprna_pro	-8	-2.5187	+8	1	$+3.03 \cdot 10^{-03}$	1
163	hprna_delay	-5	-2.9264	+5	1	$+1.18 \cdot 10^{-03}$	1
164	hprna_des	-5	-2.9214	+5	1	$+1.20 \cdot 10^{-03}$	1
165	hprna_hill	-5	+0.4497	+5	1	$+2.82 \cdot 10^{+00}$	1
166	hprna_ka	-5	+1.7030	+5	1	$+5.05 \cdot 10^{+01}$	1
167	hprna_pro	-5	-1.2230	+5	1	$+5.98 \cdot 10^{-02}$	1
168	hpxrna_delay	-5	-3.4287	+5	1	$+3.73 \cdot 10^{-04}$	1
169	hpxrna_des	-5	-3.4283	+5	1	$+3.73 \cdot 10^{-04}$	1
170	hpxrna_hill	-5	+0.1176	+5	1	$+1.31 \cdot 10^{+00}$	1
171	hpxrna_pro	-5	-3.6257	+5	1	$+2.37 \cdot 10^{-04}$	1
172	il33rna_delay	-8	-0.8718	+8	1	$+1.34 \cdot 10^{-01}$	1
173	il33rna_des	-8	-2.9602	+8	1	$+1.10 \cdot 10^{-03}$	1
174	il33rna_hill	-8	+0.2458	+8	1	$+1.76 \cdot 10^{+00}$	1
175	il33rna_pro	-8	-4.4645	+8	1	$+3.43 \cdot 10^{-05}$	1
176	jak1_act_basal	-15	-2.3652	+6	1	$+4.31 \cdot 10^{-03}$	1
177	jak1_act_il6	-15	-2.0421	+6	1	$+9.08 \cdot 10^{-03}$	1
178	jak1_gp130_dea	-15	-1.1070	+6	1	$+7.82 \cdot 10^{-02}$	1
179	jak1_inh_ruxolitinib	-15	-2.2202	+6	1	$+6.02 \cdot 10^{-03}$	1

Table S105: Estimated parameter values
$\hat{\theta}$ indicates the estimated value of the parameters. θ_{min} and θ_{max} indicate the upper and lower bounds for the parameters. The log-column indicates if the value of a parameter was log-transformed. If log = 1 the non-log-column indicates the non-logarithmic value of the estimate. The fitted-column indicates if the parameter value was estimated (1), was temporarily fixed (0) or if its value was fixed to a constant value (2).

	name	θ_{min}	$\hat{\theta}$	θ_{max}	log	non-log $\hat{\theta}$	fitted
180	offset_apcs_qpcr_37	-15	-1.0506	+3	1	$+8.90 \cdot 10^{-02}$	1
181	offset_apcs_qpcr_38	-15	-0.7680	+3	1	$+1.71 \cdot 10^{-01}$	1
182	offset_apcs_qpcr_39	-15	-1.0433	+3	1	$+9.05 \cdot 10^{-02}$	1
183	offset_apcs_qpcr_58	-5	-0.9487	+3	1	$+1.13 \cdot 10^{-01}$	1
184	offset_apcs_qpcr_59	-5	-0.9953	+3	1	$+1.01 \cdot 10^{-01}$	1
185	offset_apcs_qpcr_62	-5	-0.7649	+3	1	$+1.72 \cdot 10^{-01}$	1
186	offset_apcs_qpcr_63	-5	-0.6527	+3	1	$+2.23 \cdot 10^{-01}$	1
187	offset_apcs_qpcr_64	-5	-0.6600	+3	1	$+2.19 \cdot 10^{-01}$	1
188	offset_apcs_qpcr_67	-5	-1.0000	+3	1	$+1.00 \cdot 10^{-01}$	1
189	offset_apcs_qpcr_tc	-15	+0.1174	+3	0	$+1.17 \cdot 10^{-01}$	1
190	offset_cxcl10_qpcr_31	-15	-0.7989	+3	1	$+1.59 \cdot 10^{-01}$	1
191	offset_cxcl10_qpcr_32	-15	-0.9728	+3	1	$+1.06 \cdot 10^{-01}$	1
192	offset_cxcl10_qpcr_33	-15	-0.9006	+3	1	$+1.26 \cdot 10^{-01}$	1
193	offset_cxcl10_qpcr_56	-5	-0.9303	+3	1	$+1.17 \cdot 10^{-01}$	1
194	offset_cxcl10_qpcr_57	-5	-1.0234	+3	1	$+9.48 \cdot 10^{-02}$	1
195	offset_cxcl10_qpcr_62	-5	-0.2505	+3	1	$+5.62 \cdot 10^{-01}$	1
196	offset_cxcl10_qpcr_63	-5	-0.2445	+3	1	$+5.69 \cdot 10^{-01}$	1
197	offset_cxcl10_qpcr_64	-5	-0.3794	+3	1	$+4.17 \cdot 10^{-01}$	1
198	offset_cxcl10_qpcr_65	-5	-0.6629	+3	1	$+2.17 \cdot 10^{-01}$	1
199	offset_cxcl10_qpcr_66	-5	-0.6577	+3	1	$+2.20 \cdot 10^{-01}$	1
200	offset_cxcl10_qpcr_67	-5	-1.0000	+3	1	$+1.00 \cdot 10^{-01}$	1
201	offset_cxcl10_qpcr_tc	-15	+0.0163	+3	0	$+1.63 \cdot 10^{-02}$	1
202	offset_fgg_qpcr_34	-15	-0.8828	+3	1	$+1.31 \cdot 10^{-01}$	1
203	offset_fgg_qpcr_35	-15	-0.8591	+3	1	$+1.38 \cdot 10^{-01}$	1
204	offset_fgg_qpcr_36	-15	-0.9844	+3	1	$+1.04 \cdot 10^{-01}$	1
205	offset_fgg_qpcr_37	-15	-1.1722	+3	1	$+6.73 \cdot 10^{-02}$	1
206	offset_fgg_qpcr_38	-15	-1.3851	+3	1	$+4.12 \cdot 10^{-02}$	1
207	offset_fgg_qpcr_39	-15	-1.2642	+3	1	$+5.44 \cdot 10^{-02}$	1
208	offset_fgg_qpcr_58	-5	-1.0038	+3	1	$+9.91 \cdot 10^{-02}$	1
209	offset_fgg_qpcr_59	-5	-0.9542	+3	1	$+1.11 \cdot 10^{-01}$	1
210	offset_fgg_qpcr_60	-5	-0.8202	+3	1	$+1.51 \cdot 10^{-01}$	1
211	offset_fgg_qpcr_61	-5	-0.9045	+3	1	$+1.25 \cdot 10^{-01}$	1
212	offset_fgg_qpcr_62	-5	-0.7747	+3	1	$+1.68 \cdot 10^{-01}$	1
213	offset_fgg_qpcr_63	-5	-0.8641	+3	1	$+1.37 \cdot 10^{-01}$	1
214	offset_fgg_qpcr_64	-5	-0.9927	+3	1	$+1.02 \cdot 10^{-01}$	1
215	offset_fgg_qpcr_67	-5	-1.0000	+3	1	$+1.00 \cdot 10^{-01}$	1
216	offset_fgg_qpcr_tc	-15	+0.2483	+3	0	$+2.48 \cdot 10^{-01}$	1
217	offset_hamp_qpcr_34	-15	-0.8810	+3	1	$+1.32 \cdot 10^{-01}$	1
218	offset_hamp_qpcr_35	-15	-0.7402	+3	1	$+1.82 \cdot 10^{-01}$	1
219	offset_hamp_qpcr_36	-15	-0.8199	+3	1	$+1.51 \cdot 10^{-01}$	1
220	offset_hamp_qpcr_37	-15	-1.0698	+3	1	$+8.52 \cdot 10^{-02}$	1
221	offset_hamp_qpcr_38	-15	-0.8195	+3	1	$+1.52 \cdot 10^{-01}$	1
222	offset_hamp_qpcr_39	-15	-1.0996	+3	1	$+7.95 \cdot 10^{-02}$	1
223	offset_hamp_qpcr_58	-5	-1.0976	+3	1	$+7.99 \cdot 10^{-02}$	1
224	offset_hamp_qpcr_59	-5	-0.9679	+3	1	$+1.08 \cdot 10^{-01}$	1

Table S106: Estimated parameter values
$\hat{\theta}$ indicates the estimated value of the parameters. θ_{min} and θ_{max} indicate the upper and lower bounds for the parameters. The log-column indicates if the value of a parameter was log-transformed. If log = 1 the non-log-column indicates the non-logarithmic value of the estimate. The fitted-column indicates if the parameter value was estimated (1), was temporarily fixed (0) or if its value was fixed to a constant value (2).

	name	θ_{min}	$\hat{\theta}$	θ_{max}	log	non-log $\hat{\theta}$	fitted
225	offset_hamp_qpcr_60	-5	-1.4413	+3	1	$+3.62 \cdot 10^{-02}$	1
226	offset_hamp_qpcr_61	-5	-1.3330	+3	1	$+4.65 \cdot 10^{-02}$	1
227	offset_hamp_qpcr_62	-5	-1.3435	+3	1	$+4.53 \cdot 10^{-02}$	1
228	offset_hamp_qpcr_63	-5	-1.1738	+3	1	$+6.70 \cdot 10^{-02}$	1
229	offset_hamp_qpcr_64	-5	-1.1634	+3	1	$+6.86 \cdot 10^{-02}$	1
230	offset_hamp_qpcr_67	-5	-1.0000	+3	1	$+1.00 \cdot 10^{-01}$	1
231	offset_hamp_qpcr_tc	-15	-0.0089	+3	0	$-8.88 \cdot 10^{-03}$	1
232	offset_hp_qpcr_37	-15	-0.5348	+3	1	$+2.92 \cdot 10^{-01}$	1
233	offset_hp_qpcr_38	-15	-0.5654	+3	1	$+2.72 \cdot 10^{-01}$	1
234	offset_hp_qpcr_39	-15	-0.5208	+3	1	$+3.01 \cdot 10^{-01}$	1
235	offset_hp_qpcr_58	-5	-0.4455	+3	1	$+3.59 \cdot 10^{-01}$	1
236	offset_hp_qpcr_59	-5	-0.4344	+3	1	$+3.68 \cdot 10^{-01}$	1
237	offset_hp_qpcr_62	-5	-0.2970	+3	1	$+5.05 \cdot 10^{-01}$	1
238	offset_hp_qpcr_63	-5	-0.4375	+3	1	$+3.65 \cdot 10^{-01}$	1
239	offset_hp_qpcr_64	-5	-0.4904	+3	1	$+3.23 \cdot 10^{-01}$	1
240	offset_hp_qpcr_67	-5	-1.0000	+3	1	$+1.00 \cdot 10^{-01}$	1
241	offset_hp_qpcr_tc	-15	+0.6185	+3	0	$+6.19 \cdot 10^{-01}$	1
242	offset_hpx_qpcr_37	-15	-3.2034	+3	1	$+6.26 \cdot 10^{-04}$	1
243	offset_hpx_qpcr_38	-15	-1.1815	+3	1	$+6.58 \cdot 10^{-02}$	1
244	offset_hpx_qpcr_39	-15	-1.7741	+3	1	$+1.68 \cdot 10^{-02}$	1
245	offset_hpx_qpcr_58	-5	-0.7055	+3	1	$+1.97 \cdot 10^{-01}$	1
246	offset_hpx_qpcr_59	-5	-0.8111	+3	1	$+1.54 \cdot 10^{-01}$	1
247	offset_hpx_qpcr_62	-5	-0.7340	+3	1	$+1.84 \cdot 10^{-01}$	1
248	offset_hpx_qpcr_63	-5	-1.1171	+3	1	$+7.64 \cdot 10^{-02}$	1
249	offset_hpx_qpcr_64	-5	-1.2589	+3	1	$+5.51 \cdot 10^{-02}$	1
250	offset_hpx_qpcr_67	-5	-1.0000	+3	1	$+1.00 \cdot 10^{-01}$	1
251	offset_hpx_qpcr_tc	-15	-0.0587	+3	0	$-5.87 \cdot 10^{-02}$	1
252	offset_il33_qpcr_34	-15	-0.5320	+3	1	$+2.94 \cdot 10^{-01}$	1
253	offset_il33_qpcr_35	-15	-0.6069	+3	1	$+2.47 \cdot 10^{-01}$	1
254	offset_il33_qpcr_36	-15	-0.6776	+3	1	$+2.10 \cdot 10^{-01}$	1
255	offset_il33_qpcr_37	-15	-0.3959	+3	1	$+4.02 \cdot 10^{-01}$	1
256	offset_il33_qpcr_38	-15	-0.4643	+3	1	$+3.43 \cdot 10^{-01}$	1
257	offset_il33_qpcr_39	-15	-0.4570	+3	1	$+3.49 \cdot 10^{-01}$	1
258	offset_il33_qpcr_58	-5	-0.5735	+3	1	$+2.67 \cdot 10^{-01}$	1
259	offset_il33_qpcr_59	-5	-0.2948	+3	1	$+5.07 \cdot 10^{-01}$	1
260	offset_il33_qpcr_60	-5	-0.8302	+3	1	$+1.48 \cdot 10^{-01}$	1
261	offset_il33_qpcr_61	-5	-0.5177	+3	1	$+3.04 \cdot 10^{-01}$	1
262	offset_il33_qpcr_62	-5	-0.4068	+3	1	$+3.92 \cdot 10^{-01}$	1
263	offset_il33_qpcr_63	-5	-0.4104	+3	1	$+3.89 \cdot 10^{-01}$	1
264	offset_il33_qpcr_64	-5	-0.4975	+3	1	$+3.18 \cdot 10^{-01}$	1
265	offset_il33_qpcr_67	-5	-1.0000	+3	1	$+1.00 \cdot 10^{-01}$	1
266	offset_il33_qpcr_tc	-15	-0.0315	+3	0	$-3.15 \cdot 10^{-02}$	1
267	offset_npstat3_wb_8	-15	-1.3564	+6	1	$+4.40 \cdot 10^{-02}$	1
268	offset_npstat3_wb_9	-15	-1.1442	+6	1	$+7.17 \cdot 10^{-02}$	1
269	offset_npstat3_wb_10	-15	-1.0415	+6	1	$+9.09 \cdot 10^{-02}$	1

Table S107: Estimated parameter values
$\hat{\theta}$ indicates the estimated value of the parameters. θ_{min} and θ_{max} indicate the upper and lower bounds for the parameters. The log-column indicates if the value of a parameter was log-transformed. If log = 1 the non-log-column indicates the non-logarithmic value of the estimate. The fitted-column indicates if the parameter value was estimated (1), was temporarily fixed (0) or if its value was fixed to a constant value (2).

	name	θ_{min}	$\hat{\theta}$	θ_{max}	log	non-log $\hat{\theta}$	fitted
270	offset_npstat3_wb_11	-15	-0.9224	+6	1	$+1.20 \cdot 10^{-01}$	1
271	offset_npstat3_wb_12	-15	-0.9730	+6	1	$+1.06 \cdot 10^{-01}$	1
272	offset_npstat3_wb_13	-15	-0.9852	+6	1	$+1.03 \cdot 10^{-01}$	1
273	offset_npstat3_wb_14	-15	-1.0216	+6	1	$+9.51 \cdot 10^{-02}$	1
274	offset_npstat3_wb_15	-15	-1.0534	+6	1	$+8.84 \cdot 10^{-02}$	1
275	offset_npstat3_wb_17	-15	-0.9435	+6	1	$+1.14 \cdot 10^{-01}$	1
276	offset_ntstat3_71	-5	+1.1065	+3	1	$+1.28 \cdot 10^{+01}$	1
277	offset_ntstat3_72	-5	+1.3918	+3	1	$+2.47 \cdot 10^{+01}$	1
278	offset_ntstat3_73	-5	-5.0000	+3	1	$+1.00 \cdot 10^{-05}$	1
279	offset_ntstat3_74	-5	+1.0070	+3	1	$+1.02 \cdot 10^{+01}$	1
280	offset_ntstat3_75	-5	+1.1475	+3	1	$+1.40 \cdot 10^{+01}$	1
281	offset_ntstat3_76	-5	+0.6429	+3	1	$+4.39 \cdot 10^{+00}$	1
282	offset_pgp130_wb_1	-15	-1.2472	+6	1	$+5.66 \cdot 10^{-02}$	1
283	offset_pgp130_wb_2	-15	-0.8041	+6	1	$+1.57 \cdot 10^{-01}$	1
284	offset_pgp130_wb_4	-15	-1.9824	+6	1	$+1.04 \cdot 10^{-02}$	1
285	offset_pgp130_wb_5	-15	-1.3460	+6	1	$+4.51 \cdot 10^{-02}$	1
286	offset_pgp130_wb_6	-15	-0.5029	+6	1	$+3.14 \cdot 10^{-01}$	1
287	offset_pgp130_wb_8	-15	-0.5485	+6	1	$+2.83 \cdot 10^{-01}$	1
288	offset_pgp130_wb_9	-15	-0.8412	+6	1	$+1.44 \cdot 10^{-01}$	1
289	offset_pgp130_wb_10	-15	-1.0907	+6	1	$+8.12 \cdot 10^{-02}$	1
290	offset_pgp130_wb_11	-15	-0.6705	+6	1	$+2.14 \cdot 10^{-01}$	1
291	offset_pgp130_wb_12	-15	-0.3986	+6	1	$+3.99 \cdot 10^{-01}$	1
292	offset_pgp130_wb_13	-15	-0.8807	+6	1	$+1.32 \cdot 10^{-01}$	1
293	offset_pgp130_wb_14	-15	-0.3616	+6	1	$+4.35 \cdot 10^{-01}$	1
294	offset_pgp130_wb_15	-15	-0.6896	+6	1	$+2.04 \cdot 10^{-01}$	1
295	offset_pgp130_wb_16	-15	-0.9240	+6	1	$+1.19 \cdot 10^{-01}$	1
296	offset_pgp130_wb_17	-15	-0.4248	+6	1	$+3.76 \cdot 10^{-01}$	1
297	offset_pjak1_wb_1	-15	-0.5643	+6	1	$+2.73 \cdot 10^{-01}$	1
298	offset_pjak1_wb_2	-15	-0.6109	+6	1	$+2.45 \cdot 10^{-01}$	1
299	offset_pjak1_wb_4	-15	-0.8599	+6	1	$+1.38 \cdot 10^{-01}$	1
300	offset_pjak1_wb_5	-15	-0.6755	+6	1	$+2.11 \cdot 10^{-01}$	1
301	offset_pjak1_wb_6	-15	-0.6086	+6	1	$+2.46 \cdot 10^{-01}$	1
302	offset_pjak1_wb_13	-15	-0.6127	+6	1	$+2.44 \cdot 10^{-01}$	1
303	offset_pjak1_wb_16	-15	-0.3458	+6	1	$+4.51 \cdot 10^{-01}$	1
304	offset_pjak1_wb_17	-15	-0.2812	+6	1	$+5.23 \cdot 10^{-01}$	1
305	offset_pstat3_lumi_48	-15	-1.0228	+6	1	$+9.49 \cdot 10^{-02}$	1
306	offset_pstat3_lumi_49	-15	-14.0201	+6	1	$+9.55 \cdot 10^{-15}$	1
307	offset_pstat3_lumi_54	-15	-0.9483	+6	1	$+1.13 \cdot 10^{-01}$	1
308	offset_pstat3_lumi_55	-15	-0.8724	+6	1	$+1.34 \cdot 10^{-01}$	1
309	offset_pstat3_wb_1	-15	-0.6929	+6	1	$+2.03 \cdot 10^{-01}$	1
310	offset_pstat3_wb_2	-15	-0.7567	+6	1	$+1.75 \cdot 10^{-01}$	1
311	offset_pstat3_wb_3	-15	-14.1369	+6	1	$+7.30 \cdot 10^{-15}$	1
312	offset_pstat3_wb_4	-15	-1.1600	+6	1	$+6.92 \cdot 10^{-02}$	1
313	offset_pstat3_wb_5	-15	-0.9380	+6	1	$+1.15 \cdot 10^{-01}$	1
314	offset_pstat3_wb_6	-15	-14.0615	+6	1	$+8.68 \cdot 10^{-15}$	1

Table S108: Estimated parameter values
$\hat{\theta}$ indicates the estimated value of the parameters. θ_{min} and θ_{max} indicate the upper and lower bounds for the parameters. The log-column indicates if the value of a parameter was log-transformed. If log = 1 the non-log-column indicates the non-logarithmic value of the estimate. The fitted-column indicates if the parameter value was estimated (1), was temporarily fixed (0) or if its value was fixed to a constant value (2).

	name	θ_{min}	$\hat{\theta}$	θ_{max}	log	non-log $\hat{\theta}$	fitted
315	offset_pstat3_wb_7	-15	-1.5405	+6	1	$+2.88 \cdot 10^{-02}$	1
316	offset_pstat3_wb_9	-15	-1.6143	+6	1	$+2.43 \cdot 10^{-02}$	1
317	offset_pstat3_wb_10	-15	-1.0135	+6	1	$+9.69 \cdot 10^{-02}$	1
318	offset_pstat3_wb_11	-15	-1.0986	+6	1	$+7.97 \cdot 10^{-02}$	1
319	offset_pstat3_wb_12	-15	-1.5458	+6	1	$+2.85 \cdot 10^{-02}$	1
320	offset_pstat3_wb_13	-15	-0.8770	+6	1	$+1.33 \cdot 10^{-01}$	1
321	offset_pstat3_wb_14	-15	-1.5163	+6	1	$+3.05 \cdot 10^{-02}$	1
322	offset_pstat3_wb_15	-15	-2.5955	+6	1	$+2.54 \cdot 10^{-03}$	1
323	offset_pstat3_wb_16	-15	-1.1832	+6	1	$+6.56 \cdot 10^{-02}$	1
324	offset_pstat3_wb_17	-15	-0.5577	+6	1	$+2.77 \cdot 10^{-01}$	1
325	offset_pstat3_wb_41	-15	-13.8920	+6	1	$+1.28 \cdot 10^{-14}$	1
326	offset_pstat3_wb_42	-15	-1.4702	+6	1	$+3.39 \cdot 10^{-02}$	1
327	offset_pstat3_wb_43	-15	-2.0356	+6	1	$+9.21 \cdot 10^{-03}$	1
328	offset_pstat3_wb_44_TC	-15	-1.6020	+6	1	$+2.50 \cdot 10^{-02}$	1
329	offset_pstat3_wb_46	-15	-2.0429	+6	1	$+9.06 \cdot 10^{-03}$	1
330	offset_pstat3_wb_47	-15	-1.4664	+6	1	$+3.42 \cdot 10^{-02}$	1
331	offset_pstat3_wb_48	-15	-13.4426	+6	1	$+3.61 \cdot 10^{-14}$	1
332	offset_pstat3_wb_49	-15	-13.9987	+6	1	$+1.00 \cdot 10^{-14}$	1
333	offset_pstat3_wb_50	-15	-2.4600	+6	1	$+3.47 \cdot 10^{-03}$	1
334	offset_pstat3_wb_51	-15	-14.0051	+6	1	$+9.88 \cdot 10^{-15}$	1
335	offset_socs3_qpcr_18	-15	-1.3604	+6	1	$+4.36 \cdot 10^{-02}$	1
336	offset_socs3_qpcr_20	-15	-1.5272	+6	1	$+2.97 \cdot 10^{-02}$	1
337	offset_socs3_qpcr_21	-15	-1.0950	+6	1	$+8.03 \cdot 10^{-02}$	1
338	offset_socs3_qpcr_22	-15	-0.8947	+6	1	$+1.27 \cdot 10^{-01}$	1
339	offset_socs3_qpcr_23	-15	-1.5398	+6	1	$+2.89 \cdot 10^{-02}$	1
340	offset_socs3_qpcr_24	-15	-1.5969	+6	1	$+2.53 \cdot 10^{-02}$	1
341	offset_socs3_qpcr_25	-15	-1.5656	+6	1	$+2.72 \cdot 10^{-02}$	1
342	offset_socs3_qpcr_26	-15	-1.3364	+6	1	$+4.61 \cdot 10^{-02}$	1
343	offset_socs3_qpcr_27	-15	-1.5029	+6	1	$+3.14 \cdot 10^{-02}$	1
344	offset_socs3_qpcr_28	-15	-1.7975	+6	1	$+1.59 \cdot 10^{-02}$	1
345	offset_socs3_qpcr_29	-15	-1.1538	+6	1	$+7.02 \cdot 10^{-02}$	1
346	offset_socs3_qpcr_30	-15	-1.3267	+6	1	$+4.71 \cdot 10^{-02}$	1
347	offset_socs3_qpcr_44	-15	-1.3325	+6	1	$+4.65 \cdot 10^{-02}$	1
348	offset_socs3_qpcr_52	-15	-1.8408	+6	1	$+1.44 \cdot 10^{-02}$	1
349	offset_socs3_qpcr_53	-15	-1.9314	+6	1	$+1.17 \cdot 10^{-02}$	1
350	offset_socs3_qpcr_62	-5	-1.1868	+3	1	$+6.50 \cdot 10^{-02}$	1
351	offset_socs3_qpcr_63	-5	-1.3757	+3	1	$+4.21 \cdot 10^{-02}$	1
352	offset_socs3_qpcr_64	-5	-1.3969	+3	1	$+4.01 \cdot 10^{-02}$	1
353	offset_socs3_wb_14	-15	-0.9710	+6	1	$+1.07 \cdot 10^{-01}$	1
354	offset_socs3_wb_15	-15	-0.8852	+6	1	$+1.30 \cdot 10^{-01}$	1
355	offset_socs3_wb_16	-15	-0.6216	+6	1	$+2.39 \cdot 10^{-01}$	1
356	offset_socs3_wb_17	-15	-0.6569	+6	1	$+2.20 \cdot 10^{-01}$	1
357	rux_degrade	-15	-3.0129	+6	1	$+9.71 \cdot 10^{-04}$	1
358	scale_apcs_qpcr_37	-15	+2.6611	+6	1	$+4.58 \cdot 10^{+02}$	1
359	scale_apcs_qpcr_38	-15	+2.7021	+6	1	$+5.04 \cdot 10^{+02}$	1

Table S109: Estimated parameter values
$\hat{\theta}$ indicates the estimated value of the parameters. θ_{min} and θ_{max} indicate the upper and lower bounds for the parameters. The log-column indicates if the value of a parameter was log-transformed. If log = 1 the non-log-column indicates the non-logarithmic value of the estimate. The fitted-column indicates if the parameter value was estimated (1), was temporarily fixed (0) or if its value was fixed to a constant value (2).

	name	θ_{min}	$\hat{\theta}$	θ_{max}	log	non-log $\hat{\theta}$	fitted
360	scale_apcs_qpcr_39	-15	+2.7053	+6	1	$+5.07 \cdot 10^{+02}$	1
361	scale_apcs_qpcr_58	-5	+2.7121	+3	1	$+5.15 \cdot 10^{+02}$	1
362	scale_apcs_qpcr_59	-5	+2.6780	+3	1	$+4.76 \cdot 10^{+02}$	1
363	scale_apcs_qpcr_62	-5	+2.7437	+3	1	$+5.54 \cdot 10^{+02}$	1
364	scale_apcs_qpcr_63	-5	+2.8397	+3	1	$+6.91 \cdot 10^{+02}$	1
365	scale_apcs_qpcr_64	-5	+2.7980	+3	1	$+6.28 \cdot 10^{+02}$	1
366	scale_apcs_qpcr_67	-5	-1.0000	+3	1	$+1.00 \cdot 10^{-01}$	1
367	scale_apcs_qpcr_tc_1	-15	+3.0126	+6	1	$+1.03 \cdot 10^{+03}$	1
368	scale_apcs_qpcr_tc_2	-15	+2.8743	+6	1	$+7.49 \cdot 10^{+02}$	1
369	scale_cxcl10_qpcr_31	-15	+2.4397	+6	1	$+2.75 \cdot 10^{+02}$	1
370	scale_cxcl10_qpcr_32	-15	+2.4266	+6	1	$+2.67 \cdot 10^{+02}$	1
371	scale_cxcl10_qpcr_33	-15	+2.3347	+6	1	$+2.16 \cdot 10^{+02}$	1
372	scale_cxcl10_qpcr_56	-5	+2.3663	+3	1	$+2.32 \cdot 10^{+02}$	1
373	scale_cxcl10_qpcr_57	-5	+2.3249	+3	1	$+2.11 \cdot 10^{+02}$	1
374	scale_cxcl10_qpcr_62	-5	-3.1723	+3	1	$+6.72 \cdot 10^{-04}$	1
375	scale_cxcl10_qpcr_63	-5	-3.1488	+3	1	$+7.10 \cdot 10^{-04}$	1
376	scale_cxcl10_qpcr_64	-5	-3.5044	+3	1	$+3.13 \cdot 10^{-04}$	1
377	scale_cxcl10_qpcr_65	-5	+2.2835	+3	1	$+1.92 \cdot 10^{+02}$	1
378	scale_cxcl10_qpcr_66	-5	+2.3201	+3	1	$+2.09 \cdot 10^{+02}$	1
379	scale_cxcl10_qpcr_67	-5	-1.0000	+3	1	$+1.00 \cdot 10^{-01}$	1
380	scale_cxcl10_qpcr_tc_1	-15	+2.9485	+6	1	$+8.88 \cdot 10^{+02}$	1
381	scale_cxcl10_qpcr_tc_2	-15	+2.9779	+6	1	$+9.50 \cdot 10^{+02}$	1
382	scale_fgg_qpcr_34	-15	+1.3594	+6	1	$+2.29 \cdot 10^{+01}$	1
383	scale_fgg_qpcr_35	-15	+1.3306	+6	1	$+2.14 \cdot 10^{+01}$	1
384	scale_fgg_qpcr_36	-15	+1.3968	+6	1	$+2.49 \cdot 10^{+01}$	1
385	scale_fgg_qpcr_37	-15	+1.3585	+6	1	$+2.28 \cdot 10^{+01}$	1
386	scale_fgg_qpcr_38	-15	+1.3810	+6	1	$+2.40 \cdot 10^{+01}$	1
387	scale_fgg_qpcr_39	-15	+1.3499	+6	1	$+2.24 \cdot 10^{+01}$	1
388	scale_fgg_qpcr_58	-5	+1.4227	+3	1	$+2.65 \cdot 10^{+01}$	1
389	scale_fgg_qpcr_59	-5	+1.4048	+3	1	$+2.54 \cdot 10^{+01}$	1
390	scale_fgg_qpcr_60	-5	+1.4305	+3	1	$+2.69 \cdot 10^{+01}$	1
391	scale_fgg_qpcr_61	-5	+1.5382	+3	1	$+3.45 \cdot 10^{+01}$	1
392	scale_fgg_qpcr_62	-5	+1.5014	+3	1	$+3.17 \cdot 10^{+01}$	1
393	scale_fgg_qpcr_63	-5	+1.5794	+3	1	$+3.80 \cdot 10^{+01}$	1
394	scale_fgg_qpcr_64	-5	+1.5188	+3	1	$+3.30 \cdot 10^{+01}$	1
395	scale_fgg_qpcr_67	-5	-1.0000	+3	1	$+1.00 \cdot 10^{-01}$	1
396	scale_fgg_qpcr_tc_1	-15	+2.1666	+6	1	$+1.47 \cdot 10^{+02}$	1
397	scale_fgg_qpcr_tc_2	-15	+1.7927	+6	1	$+6.20 \cdot 10^{+01}$	1
398	scale_hamp_qpcr_34	-15	+0.5955	+6	1	$+3.94 \cdot 10^{+00}$	1
399	scale_hamp_qpcr_35	-15	+0.5527	+6	1	$+3.57 \cdot 10^{+00}$	1
400	scale_hamp_qpcr_36	-15	+0.5945	+6	1	$+3.93 \cdot 10^{+00}$	1
401	scale_hamp_qpcr_37	-15	+0.1045	+6	1	$+1.27 \cdot 10^{+00}$	1
402	scale_hamp_qpcr_38	-15	+0.0745	+6	1	$+1.19 \cdot 10^{+00}$	1
403	scale_hamp_qpcr_39	-15	+0.1200	+6	1	$+1.32 \cdot 10^{+00}$	1
404	scale_hamp_qpcr_58	-5	+0.0286	+3	1	$+1.07 \cdot 10^{+00}$	1

Table S110: Estimated parameter values
$\hat{\theta}$ indicates the estimated value of the parameters. θ_{min} and θ_{max} indicate the upper and lower bounds for the parameters. The log-column indicates if the value of a parameter was log-transformed. If log = 1 the non-log-column indicates the non-logarithmic value of the estimate. The fitted-column indicates if the parameter value was estimated (1), was temporarily fixed (0) or if its value was fixed to a constant value (2).

	name	θ_{min}	$\hat{\theta}$	θ_{max}	log	non-log $\hat{\theta}$	fitted
405	scale_hamp_qpcr_59	-5	+0.0724	+3	1	$+1.18 \cdot 10^{+00}$	1
406	scale_hamp_qpcr_60	-5	+0.5665	+3	1	$+3.69 \cdot 10^{+00}$	1
407	scale_hamp_qpcr_61	-5	+0.6460	+3	1	$+4.43 \cdot 10^{+00}$	1
408	scale_hamp_qpcr_62	-5	+0.2301	+3	1	$+1.70 \cdot 10^{+00}$	1
409	scale_hamp_qpcr_63	-5	+0.1679	+3	1	$+1.47 \cdot 10^{+00}$	1
410	scale_hamp_qpcr_64	-5	+0.2699	+3	1	$+1.86 \cdot 10^{+00}$	1
411	scale_hamp_qpcr_67	-5	-1.0000	+3	1	$+1.00 \cdot 10^{-01}$	1
412	scale_hamp_qpcr_tc_1	-15	+1.0698	+6	1	$+1.17 \cdot 10^{+01}$	1
413	scale_hamp_qpcr_tc_2	-15	+1.1330	+6	1	$+1.36 \cdot 10^{+01}$	1
414	scale_hp_qpcr_37	-15	+1.2459	+6	1	$+1.76 \cdot 10^{+01}$	1
415	scale_hp_qpcr_38	-15	+1.3137	+6	1	$+2.06 \cdot 10^{+01}$	1
416	scale_hp_qpcr_39	-15	+1.2219	+6	1	$+1.67 \cdot 10^{+01}$	1
417	scale_hp_qpcr_58	-5	+1.1988	+3	1	$+1.58 \cdot 10^{+01}$	1
418	scale_hp_qpcr_59	-5	+1.1636	+3	1	$+1.46 \cdot 10^{+01}$	1
419	scale_hp_qpcr_62	-5	+1.1920	+3	1	$+1.56 \cdot 10^{+01}$	1
420	scale_hp_qpcr_63	-5	+1.2852	+3	1	$+1.93 \cdot 10^{+01}$	1
421	scale_hp_qpcr_64	-5	+1.3386	+3	1	$+2.18 \cdot 10^{+01}$	1
422	scale_hp_qpcr_67	-5	-1.0000	+3	1	$+1.00 \cdot 10^{-01}$	1
423	scale_hp_qpcr_tc_1	-15	+1.6371	+6	1	$+4.34 \cdot 10^{+01}$	1
424	scale_hp_qpcr_tc_2	-15	+1.3967	+6	1	$+2.49 \cdot 10^{+01}$	1
425	scale_hpx_qpcr_37	-15	+0.0982	+6	1	$+1.25 \cdot 10^{+00}$	1
426	scale_hpx_qpcr_38	-15	+0.1131	+6	1	$+1.30 \cdot 10^{+00}$	1
427	scale_hpx_qpcr_39	-15	+0.0844	+6	1	$+1.21 \cdot 10^{+00}$	1
428	scale_hpx_qpcr_58	-5	-0.0115	+3	1	$+9.74 \cdot 10^{-01}$	1
429	scale_hpx_qpcr_59	-5	-0.0021	+3	1	$+9.95 \cdot 10^{-01}$	1
430	scale_hpx_qpcr_62	-5	+0.1191	+3	1	$+1.32 \cdot 10^{+00}$	1
431	scale_hpx_qpcr_63	-5	+0.1350	+3	1	$+1.36 \cdot 10^{+00}$	1
432	scale_hpx_qpcr_64	-5	+0.1548	+3	1	$+1.43 \cdot 10^{+00}$	1
433	scale_hpx_qpcr_67	-5	-1.0000	+3	1	$+1.00 \cdot 10^{-01}$	1
434	scale_hpx_qpcr_tc_1	-15	+0.6280	+6	1	$+4.25 \cdot 10^{+00}$	1
435	scale_hpx_qpcr_tc_2	-15	+0.1365	+6	1	$+1.37 \cdot 10^{+00}$	1
436	scale_il33_qpcr_34	-15	+0.1663	+6	1	$+1.47 \cdot 10^{+00}$	1
437	scale_il33_qpcr_35	-15	+0.1401	+6	1	$+1.38 \cdot 10^{+00}$	1
438	scale_il33_qpcr_36	-15	+0.1670	+6	1	$+1.47 \cdot 10^{+00}$	1
439	scale_il33_qpcr_37	-15	-0.2299	+6	1	$+5.89 \cdot 10^{-01}$	1
440	scale_il33_qpcr_38	-15	-0.1820	+6	1	$+6.58 \cdot 10^{-01}$	1
441	scale_il33_qpcr_39	-15	-0.1737	+6	1	$+6.70 \cdot 10^{-01}$	1
442	scale_il33_qpcr_58	-5	-0.1304	+3	1	$+7.41 \cdot 10^{-01}$	1
443	scale_il33_qpcr_59	-5	-0.2787	+3	1	$+5.26 \cdot 10^{-01}$	1
444	scale_il33_qpcr_60	-5	+0.1916	+3	1	$+1.55 \cdot 10^{+00}$	1
445	scale_il33_qpcr_61	-5	+0.1708	+3	1	$+1.48 \cdot 10^{+00}$	1
446	scale_il33_qpcr_62	-5	-0.0628	+3	1	$+8.65 \cdot 10^{-01}$	1
447	scale_il33_qpcr_63	-5	-0.0640	+3	1	$+8.63 \cdot 10^{-01}$	1
448	scale_il33_qpcr_64	-5	-0.0290	+3	1	$+9.35 \cdot 10^{-01}$	1
449	scale_il33_qpcr_67	-5	-1.0000	+3	1	$+1.00 \cdot 10^{-01}$	1

Table S111: Estimated parameter values
$\hat{\theta}$ indicates the estimated value of the parameters. θ_{min} and θ_{max} indicate the upper and lower bounds for the parameters. The log-column indicates if the value of a parameter was log-transformed. If log = 1 the non-log-column indicates the non-logarithmic value of the estimate. The fitted-column indicates if the parameter value was estimated (1), was temporarily fixed (0) or if its value was fixed to a constant value (2).

	name	θ_{min}	$\hat{\theta}$	θ_{max}	log	non-log $\hat{\theta}$	fitted
450	scale_il33_qpcr_tc_1	-15	+0.4213	+6	1	$+2.64 \cdot 10^{+00}$	1
451	scale_il33_qpcr_tc_2	-15	+0.5969	+6	1	$+3.95 \cdot 10^{+00}$	1
452	scale_npstat3_wb_8	-15	+0.3923	+6	1	$+2.47 \cdot 10^{+00}$	1
453	scale_npstat3_wb_9	-15	+0.4680	+6	1	$+2.94 \cdot 10^{+00}$	1
454	scale_npstat3_wb_10	-15	+0.3999	+6	1	$+2.51 \cdot 10^{+00}$	1
455	scale_npstat3_wb_11	-15	+0.3546	+6	1	$+2.26 \cdot 10^{+00}$	1
456	scale_npstat3_wb_12	-15	+0.5876	+6	1	$+3.87 \cdot 10^{+00}$	1
457	scale_npstat3_wb_13	-15	+0.2148	+6	1	$+1.64 \cdot 10^{+00}$	1
458	scale_npstat3_wb_14	-15	+0.4309	+6	1	$+2.70 \cdot 10^{+00}$	1
459	scale_npstat3_wb_15	-15	+0.4826	+6	1	$+3.04 \cdot 10^{+00}$	1
460	scale_npstat3_wb_17	-15	-0.5625	+6	1	$+2.74 \cdot 10^{-01}$	1
461	scale_ntstat3_71	-5	+0.3737	+3	1	$+2.36 \cdot 10^{+00}$	1
462	scale_ntstat3_72	-5	+0.3000	+3	1	$+2.00 \cdot 10^{+00}$	1
463	scale_ntstat3_73	-5	+0.4637	+3	1	$+2.91 \cdot 10^{+00}$	1
464	scale_ntstat3_74	-5	+0.3406	+3	1	$+2.19 \cdot 10^{+00}$	1
465	scale_ntstat3_75	-5	+0.3343	+3	1	$+2.16 \cdot 10^{+00}$	1
466	scale_ntstat3_76	-5	+0.3731	+3	1	$+2.36 \cdot 10^{+00}$	1
467	scale_pgp130_wb_1	-15	+0.4813	+6	1	$+3.03 \cdot 10^{+00}$	1
468	scale_pgp130_wb_2	-15	+0.5947	+6	1	$+3.93 \cdot 10^{+00}$	1
469	scale_pgp130_wb_4	-15	+0.6486	+6	1	$+4.45 \cdot 10^{+00}$	1
470	scale_pgp130_wb_5	-15	+0.6135	+6	1	$+4.11 \cdot 10^{+00}$	1
471	scale_pgp130_wb_6	-5	+0.8987	+5	1	$+7.92 \cdot 10^{+00}$	1
472	scale_pgp130_wb_8	-15	-0.0845	+6	1	$+8.23 \cdot 10^{-01}$	1
473	scale_pgp130_wb_9	-15	+0.3993	+6	1	$+2.51 \cdot 10^{+00}$	1
474	scale_pgp130_wb_10	-15	+0.3044	+6	1	$+2.02 \cdot 10^{+00}$	1
475	scale_pgp130_wb_11	-15	+0.0346	+6	1	$+1.08 \cdot 10^{+00}$	1
476	scale_pgp130_wb_12	-15	+0.2922	+6	1	$+1.96 \cdot 10^{+00}$	1
477	scale_pgp130_wb_13	-15	+0.3352	+6	1	$+2.16 \cdot 10^{+00}$	1
478	scale_pgp130_wb_14	-15	-0.0298	+6	1	$+9.34 \cdot 10^{-01}$	1
479	scale_pgp130_wb_15	-15	+0.3746	+6	1	$+2.37 \cdot 10^{+00}$	1
480	scale_pgp130_wb_16	-15	+0.4654	+6	1	$+2.92 \cdot 10^{+00}$	1
481	scale_pgp130_wb_17	-15	+0.1933	+6	1	$+1.56 \cdot 10^{+00}$	1
482	scale_pjak1_wb_1	-15	+0.2752	+6	1	$+1.88 \cdot 10^{+00}$	1
483	scale_pjak1_wb_2	-15	+0.3467	+6	1	$+2.22 \cdot 10^{+00}$	1
484	scale_pjak1_wb_4	-15	+0.4807	+6	1	$+3.02 \cdot 10^{+00}$	1
485	scale_pjak1_wb_5	-15	+0.3099	+6	1	$+2.04 \cdot 10^{+00}$	1
486	scale_pjak1_wb_6	-15	+0.3083	+6	1	$+2.03 \cdot 10^{+00}$	1
487	scale_pjak1_wb_13	-15	+0.0338	+6	1	$+1.08 \cdot 10^{+00}$	1
488	scale_pjak1_wb_16	-15	-0.5027	+6	1	$+3.14 \cdot 10^{-01}$	1
489	scale_pjak1_wb_17	-15	-13.9023	+6	1	$+1.25 \cdot 10^{-14}$	1
490	scale_pstat3_lumi_48	-15	+1.6421	+6	1	$+4.39 \cdot 10^{+01}$	1
491	scale_pstat3_lumi_49	-15	+1.6082	+6	1	$+4.06 \cdot 10^{+01}$	1
492	scale_pstat3_lumi_54	-15	+1.5605	+6	1	$+3.63 \cdot 10^{+01}$	1
493	scale_pstat3_lumi_55	-15	+1.4694	+6	1	$+2.95 \cdot 10^{+01}$	1
494	scale_pstat3_wb_1	-15	+1.2008	+6	1	$+1.59 \cdot 10^{+01}$	1

Table S112: Estimated parameter values
$\hat{\theta}$ indicates the estimated value of the parameters. θ_{min} and θ_{max} indicate the upper and lower bounds for the parameters. The log-column indicates if the value of a parameter was log-transformed. If log = 1 the non-log-column indicates the non-logarithmic value of the estimate. The fitted-column indicates if the parameter value was estimated (1), was temporarily fixed (0) or if its value was fixed to a constant value (2).

	name	θ_{min}	$\hat{\theta}$	θ_{max}	log	non-log $\hat{\theta}$	fitted
495	scale_pstat3_wb_2	-15	+1.4978	+6	1	$+3.15 \cdot 10^{+01}$	1
496	scale_pstat3_wb_3	-15	+1.5998	+6	1	$+3.98 \cdot 10^{+01}$	1
497	scale_pstat3_wb_4	-15	+1.5971	+6	1	$+3.95 \cdot 10^{+01}$	1
498	scale_pstat3_wb_5	-15	+1.4540	+6	1	$+2.84 \cdot 10^{+01}$	1
499	scale_pstat3_wb_6	-15	+1.8446	+6	1	$+6.99 \cdot 10^{+01}$	1
500	scale_pstat3_wb_7	-15	+1.5362	+6	1	$+3.44 \cdot 10^{+01}$	1
501	scale_pstat3_wb_9	-15	+1.4453	+6	1	$+2.79 \cdot 10^{+01}$	1
502	scale_pstat3_wb_10	-15	+1.1703	+6	1	$+1.48 \cdot 10^{+01}$	1
503	scale_pstat3_wb_11	-15	+0.9118	+6	1	$+8.16 \cdot 10^{+00}$	1
504	scale_pstat3_wb_12	-15	+1.2642	+6	1	$+1.84 \cdot 10^{+01}$	1
505	scale_pstat3_wb_13	-15	+0.7677	+6	1	$+5.86 \cdot 10^{+00}$	1
506	scale_pstat3_wb_14	-15	+1.2210	+6	1	$+1.66 \cdot 10^{+01}$	1
507	scale_pstat3_wb_15	-15	+1.3015	+6	1	$+2.00 \cdot 10^{+01}$	1
508	scale_pstat3_wb_16	-15	+1.5498	+6	1	$+3.55 \cdot 10^{+01}$	1
509	scale_pstat3_wb_17	-15	-13.9008	+6	1	$+1.26 \cdot 10^{-14}$	1
510	scale_pstat3_wb_41	-15	+1.6141	+6	1	$+4.11 \cdot 10^{+01}$	1
511	scale_pstat3_wb_42	-15	+1.6757	+6	1	$+4.74 \cdot 10^{+01}$	1
512	scale_pstat3_wb_43	-15	+1.7163	+6	1	$+5.20 \cdot 10^{+01}$	1
513	scale_pstat3_wb_44_TC	-15	+1.6577	+6	1	$+4.55 \cdot 10^{+01}$	1
514	scale_pstat3_wb_46	-15	+1.5827	+6	1	$+3.83 \cdot 10^{+01}$	1
515	scale_pstat3_wb_47	-15	+1.7030	+6	1	$+5.05 \cdot 10^{+01}$	1
516	scale_pstat3_wb_48	-15	+1.6593	+6	1	$+4.56 \cdot 10^{+01}$	1
517	scale_pstat3_wb_49	-15	+1.6153	+6	1	$+4.12 \cdot 10^{+01}$	1
518	scale_pstat3_wb_50	-15	+1.1288	+6	1	$+1.35 \cdot 10^{+01}$	1
519	scale_pstat3_wb_51	-15	+1.1913	+6	1	$+1.55 \cdot 10^{+01}$	1
520	scale_socs3_qpcr_18	-15	+1.7114	+6	1	$+5.15 \cdot 10^{+01}$	1
521	scale_socs3_qpcr_20	-15	+2.1732	+6	1	$+1.49 \cdot 10^{+02}$	1
522	scale_socs3_qpcr_21	-15	+2.2551	+6	1	$+1.80 \cdot 10^{+02}$	1
523	scale_socs3_qpcr_22	-15	+2.2455	+6	1	$+1.76 \cdot 10^{+02}$	1
524	scale_socs3_qpcr_23	-15	+2.2496	+6	1	$+1.78 \cdot 10^{+02}$	1
525	scale_socs3_qpcr_24	-15	+2.2880	+6	1	$+1.94 \cdot 10^{+02}$	1
526	scale_socs3_qpcr_25	-15	+2.1728	+6	1	$+1.49 \cdot 10^{+02}$	1
527	scale_socs3_qpcr_26	-15	+2.3923	+6	1	$+2.47 \cdot 10^{+02}$	1
528	scale_socs3_qpcr_27	-15	+2.2882	+6	1	$+1.94 \cdot 10^{+02}$	1
529	scale_socs3_qpcr_28	-15	+2.2542	+6	1	$+1.80 \cdot 10^{+02}$	1
530	scale_socs3_qpcr_29	-15	+2.4045	+6	1	$+2.54 \cdot 10^{+02}$	1
531	scale_socs3_qpcr_30	-15	+2.2837	+6	1	$+1.92 \cdot 10^{+02}$	1
532	scale_socs3_qpcr_44	-15	+2.2393	+6	1	$+1.74 \cdot 10^{+02}$	1
533	scale_socs3_qpcr_52	-15	+1.7928	+6	1	$+6.21 \cdot 10^{+01}$	1
534	scale_socs3_qpcr_53	-15	+1.7529	+6	1	$+5.66 \cdot 10^{+01}$	1
535	scale_socs3_qpcr_62	-5	+2.4375	+3	1	$+2.74 \cdot 10^{+02}$	1
536	scale_socs3_qpcr_63	-5	+2.3894	+3	1	$+2.45 \cdot 10^{+02}$	1
537	scale_socs3_qpcr_64	-5	+2.3540	+3	1	$+2.26 \cdot 10^{+02}$	1
538	scale_socs3_wb_14	-15	-0.9938	+6	1	$+1.01 \cdot 10^{-01}$	1
539	scale_socs3_wb_15	-15	-0.8020	+6	1	$+1.58 \cdot 10^{-01}$	1

Table S113: Estimated parameter values
$\hat{\theta}$ indicates the estimated value of the parameters. θ_{min} and θ_{max} indicate the upper and lower bounds for the parameters. The log-column indicates if the value of a parameter was log-transformed. If log = 1 the non-log-column indicates the non-logarithmic value of the estimate. The fitted-column indicates if the parameter value was estimated (1), was temporarily fixed (0) or if its value was fixed to a constant value (2).

	name	θ_{min}	$\hat{\theta}$	θ_{max}	log	non-log $\hat{\theta}$	fitted
540	scale_socs3_wb_16	-15	-0.6626	+6	1	$+2.17 \cdot 10^{-01}$	1
541	scale_socs3_wb_17	-15	-0.8172	+6	1	$+1.52 \cdot 10^{-01}$	1
542	sd_JAK1_gp130_abs	-5	-0.6736	+3	1	$+2.12 \cdot 10^{-01}$	1
543	sd_SOCS3_abs	-5	-0.8518	+3	1	$+1.41 \cdot 10^{-01}$	1
544	sd_apcs_qpcr	-5	-1.2124	+3	1	$+6.13 \cdot 10^{-02}$	1
545	sd_apcs_qpcr_tc_1	-5	-1.2081	+3	1	$+6.19 \cdot 10^{-02}$	1
546	sd_apcs_qpcr_tc_2	-5	-1.4171	+3	1	$+3.83 \cdot 10^{-02}$	1
547	sd_ctSTAT3_abs	-5	-1.1513	+3	1	$+7.06 \cdot 10^{-02}$	1
548	sd_cxcl10_qpcr	-5	-0.9320	+3	1	$+1.17 \cdot 10^{-01}$	1
549	sd_cxcl10_qpcr_tc_1	-5	-0.7594	+3	1	$+1.74 \cdot 10^{-01}$	1
550	sd_cxcl10_qpcr_tc_2	-5	-0.8036	+3	1	$+1.57 \cdot 10^{-01}$	1
551	sd_fgg_qpcr	-5	-1.2999	+3	1	$+5.01 \cdot 10^{-02}$	1
552	sd_fgg_qpcr_tc_1	-5	-0.8887	+3	1	$+1.29 \cdot 10^{-01}$	1
553	sd_fgg_qpcr_tc_2	-5	-0.8884	+3	1	$+1.29 \cdot 10^{-01}$	1
554	sd_hamp_qpcr	-5	-1.2810	+3	1	$+5.24 \cdot 10^{-02}$	1
555	sd_hamp_qpcr_tc_1	-5	-0.8114	+3	1	$+1.54 \cdot 10^{-01}$	1
556	sd_hamp_qpcr_tc_2	-5	-0.8393	+3	1	$+1.45 \cdot 10^{-01}$	1
557	sd_hp_qpcr	-5	-1.3592	+3	1	$+4.37 \cdot 10^{-02}$	1
558	sd_hp_qpcr_tc_1	-5	-1.2012	+3	1	$+6.29 \cdot 10^{-02}$	1
559	sd_hp_qpcr_tc_2	-5	-1.3354	+3	1	$+4.62 \cdot 10^{-02}$	1
560	sd_hpx_qpcr	-5	-1.2999	+3	1	$+5.01 \cdot 10^{-02}$	1
561	sd_hpx_qpcr_tc_1	-5	-1.2726	+3	1	$+5.34 \cdot 10^{-02}$	1
562	sd_hpx_qpcr_tc_2	-5	-1.3512	+3	1	$+4.45 \cdot 10^{-02}$	1
563	sd_il33_qpcr	-5	-1.4685	+3	1	$+3.40 \cdot 10^{-02}$	1
564	sd_il33_qpcr_tc_1	-5	-1.0351	+3	1	$+9.22 \cdot 10^{-02}$	1
565	sd_il33_qpcr_tc_2	-5	-0.9202	+3	1	$+1.20 \cdot 10^{-01}$	1
566	sd_npstat3_wb_8	-15	-0.6555	+6	1	$+2.21 \cdot 10^{-01}$	1
567	sd_npstat3_wb_9	-15	-0.6311	+6	1	$+2.34 \cdot 10^{-01}$	1
568	sd_npstat3_wb_10	-15	-0.7425	+6	1	$+1.81 \cdot 10^{-01}$	1
569	sd_npstat3_wb_11	-15	-0.5563	+6	1	$+2.78 \cdot 10^{-01}$	1
570	sd_npstat3_wb_12	-15	-0.6920	+6	1	$+2.03 \cdot 10^{-01}$	1
571	sd_npstat3_wb_13	-15	-0.6270	+6	1	$+2.36 \cdot 10^{-01}$	1
572	sd_npstat3_wb_14	-15	-0.7174	+6	1	$+1.92 \cdot 10^{-01}$	1
573	sd_npstat3_wb_15	-15	-0.6738	+6	1	$+2.12 \cdot 10^{-01}$	1
574	sd_npstat3_wb_17	-15	-0.3530	+6	1	$+4.44 \cdot 10^{-01}$	1
575	sd_ntstat3_ratio_70	-15	-0.6474	+6	1	$+2.25 \cdot 10^{-01}$	1
576	sd_ntstat3_validation_71	-5	-1.6425	+3	1	$+2.28 \cdot 10^{-02}$	1
577	sd_ntstat3_validation_72	-5	-1.4888	+3	1	$+3.24 \cdot 10^{-02}$	1
578	sd_ntstat3_validation_73	-5	-1.1258	+3	1	$+7.49 \cdot 10^{-02}$	1
579	sd_ntstat3_validation_74	-5	-1.1610	+3	1	$+6.90 \cdot 10^{-02}$	1
580	sd_ntstat3_validation_75	-5	-1.5449	+3	1	$+2.85 \cdot 10^{-02}$	1
581	sd_ntstat3_validation_76	-5	-1.6306	+3	1	$+2.34 \cdot 10^{-02}$	1
582	sd_pSTAT3_ms	-5	+0.1201	+0.7	1	$+1.32 \cdot 10^{+00}$	1
583	sd_pgp130_wb_1	-15	-0.7214	+6	1	$+1.90 \cdot 10^{-01}$	1
584	sd_pgp130_wb_2	-15	-1.0099	+6	1	$+9.77 \cdot 10^{-02}$	1

Table S114: Estimated parameter values
$\hat{\theta}$ indicates the estimated value of the parameters. θ_{min} and θ_{max} indicate the upper and lower bounds for the parameters. The log-column indicates if the value of a parameter was log-transformed. If log = 1 the non-log-column indicates the non-logarithmic value of the estimate. The fitted-column indicates if the parameter value was estimated (1), was temporarily fixed (0) or if its value was fixed to a constant value (2).

	name	θ_{min}	$\hat{\theta}$	θ_{max}	log	non-log $\hat{\theta}$	fitted
585	sd_pgp130_wb_4	-15	-0.6637	+6	1	$+2.17 \cdot 10^{-01}$	1
586	sd_pgp130_wb_5	-15	-0.7806	+6	1	$+1.66 \cdot 10^{-01}$	1
587	sd_pgp130_wb_6	-15	-1.0643	+6	1	$+8.62 \cdot 10^{-02}$	1
588	sd_pgp130_wb_8	-15	-0.6408	+6	1	$+2.29 \cdot 10^{-01}$	1
589	sd_pgp130_wb_9	-15	-0.7673	+6	1	$+1.71 \cdot 10^{-01}$	1
590	sd_pgp130_wb_10	-15	-0.7900	+6	1	$+1.62 \cdot 10^{-01}$	1
591	sd_pgp130_wb_11	-15	-0.8019	+6	1	$+1.58 \cdot 10^{-01}$	1
592	sd_pgp130_wb_12	-15	-0.8909	+6	1	$+1.29 \cdot 10^{-01}$	1
593	sd_pgp130_wb_13	-15	-0.7265	+6	1	$+1.88 \cdot 10^{-01}$	1
594	sd_pgp130_wb_14	-15	-0.8918	+6	1	$+1.28 \cdot 10^{-01}$	1
595	sd_pgp130_wb_15	-15	-0.7957	+6	1	$+1.60 \cdot 10^{-01}$	1
596	sd_pgp130_wb_16	-15	-0.8239	+6	1	$+1.50 \cdot 10^{-01}$	1
597	sd_pgp130_wb_17	-15	-0.7844	+6	1	$+1.64 \cdot 10^{-01}$	1
598	sd_pjak1_wb_1	-15	-0.8105	+6	1	$+1.55 \cdot 10^{-01}$	1
599	sd_pjak1_wb_2	-15	-0.9297	+6	1	$+1.18 \cdot 10^{-01}$	1
600	sd_pjak1_wb_4	-15	-0.8786	+6	1	$+1.32 \cdot 10^{-01}$	1
601	sd_pjak1_wb_5	-15	-0.7254	+6	1	$+1.88 \cdot 10^{-01}$	1
602	sd_pjak1_wb_6	-15	-0.6502	+6	1	$+2.24 \cdot 10^{-01}$	1
603	sd_pjak1_wb_13	-15	-0.7746	+6	1	$+1.68 \cdot 10^{-01}$	1
604	sd_pjak1_wb_16	-15	-0.6790	+6	1	$+2.09 \cdot 10^{-01}$	1
605	sd_pjak1_wb_17	-15	-0.8349	+6	1	$+1.46 \cdot 10^{-01}$	1
606	sd_pstat3_lumi_48	-15	-0.9592	+6	1	$+1.10 \cdot 10^{-01}$	1
607	sd_pstat3_lumi_49	-15	-1.0062	+6	1	$+9.86 \cdot 10^{-02}$	1
608	sd_pstat3_lumi_54	-15	-0.9000	+6	1	$+1.26 \cdot 10^{-01}$	1
609	sd_pstat3_lumi_55	-15	-0.9483	+6	1	$+1.13 \cdot 10^{-01}$	1
610	sd_pstat3_wb_1	-15	-0.6809	+6	1	$+2.08 \cdot 10^{-01}$	1
611	sd_pstat3_wb_2	-15	-0.9624	+6	1	$+1.09 \cdot 10^{-01}$	1
612	sd_pstat3_wb_3	-15	-1.0443	+6	1	$+9.03 \cdot 10^{-02}$	1
613	sd_pstat3_wb_4	-15	-0.7881	+6	1	$+1.63 \cdot 10^{-01}$	1
614	sd_pstat3_wb_5	-15	-0.8872	+6	1	$+1.30 \cdot 10^{-01}$	1
615	sd_pstat3_wb_6	-15	-0.6171	+6	1	$+2.41 \cdot 10^{-01}$	1
616	sd_pstat3_wb_7	-15	-1.0225	+6	1	$+9.49 \cdot 10^{-02}$	1
617	sd_pstat3_wb_9	-15	-0.5684	+6	1	$+2.70 \cdot 10^{-01}$	1
618	sd_pstat3_wb_10	-15	-0.4869	+6	1	$+3.26 \cdot 10^{-01}$	1
619	sd_pstat3_wb_11	-15	-0.4873	+6	1	$+3.26 \cdot 10^{-01}$	1
620	sd_pstat3_wb_12	-15	-0.6106	+6	1	$+2.45 \cdot 10^{-01}$	1
621	sd_pstat3_wb_13	-15	-0.2747	+6	1	$+5.31 \cdot 10^{-01}$	1
622	sd_pstat3_wb_14	-15	-0.6269	+6	1	$+2.36 \cdot 10^{-01}$	1
623	sd_pstat3_wb_15	-15	-0.6094	+6	1	$+2.46 \cdot 10^{-01}$	1
624	sd_pstat3_wb_16	-15	-0.7527	+6	1	$+1.77 \cdot 10^{-01}$	1
625	sd_pstat3_wb_17	-15	-0.4990	+6	1	$+3.17 \cdot 10^{-01}$	1
626	sd_pstat3_wb_41	-15	-0.8561	+6	1	$+1.39 \cdot 10^{-01}$	1
627	sd_pstat3_wb_42	-15	-0.8768	+6	1	$+1.33 \cdot 10^{-01}$	1
628	sd_pstat3_wb_43	-15	-0.7482	+6	1	$+1.79 \cdot 10^{-01}$	1
629	sd_pstat3_wb_44_TC	-15	-0.9886	+6	1	$+1.03 \cdot 10^{-01}$	1

Table S115: Estimated parameter values
$\hat{\theta}$ indicates the estimated value of the parameters. θ_{min} and θ_{max} indicate the upper and lower bounds for the parameters. The log-column indicates if the value of a parameter was log-transformed. If log = 1 the non-log-column indicates the non-logarithmic value of the estimate. The fitted-column indicates if the parameter value was estimated (1), was temporarily fixed (0) or if its value was fixed to a constant value (2).

	name	θ_{min}	$\hat{\theta}$	θ_{max}	log	non-log $\hat{\theta}$	fitted
630	sd_pstat3_wb_46	-15	-1.0553	+6	1	$+8.80 \cdot 10^{-02}$	1
631	sd_pstat3_wb_47	-15	-1.0610	+6	1	$+8.69 \cdot 10^{-02}$	1
632	sd_pstat3_wb_48	-15	-0.9455	+6	1	$+1.13 \cdot 10^{-01}$	1
633	sd_pstat3_wb_49	-15	-0.8734	+6	1	$+1.34 \cdot 10^{-01}$	1
634	sd_pstat3_wb_50	-15	-0.3404	+6	1	$+4.57 \cdot 10^{-01}$	1
635	sd_pstat3_wb_51	-15	-0.4066	+6	1	$+3.92 \cdot 10^{-01}$	1
636	sd_socs3_qpcr_18	-15	-0.6907	+6	1	$+2.04 \cdot 10^{-01}$	1
637	sd_socs3_qpcr_20	-15	-0.9017	+6	1	$+1.25 \cdot 10^{-01}$	1
638	sd_socs3_qpcr_21	-15	-0.7316	+6	1	$+1.86 \cdot 10^{-01}$	1
639	sd_socs3_qpcr_22	-15	-0.8084	+6	1	$+1.55 \cdot 10^{-01}$	1
640	sd_socs3_qpcr_23	-15	-1.2201	+6	1	$+6.02 \cdot 10^{-02}$	1
641	sd_socs3_qpcr_24	-15	-1.2028	+6	1	$+6.27 \cdot 10^{-02}$	1
642	sd_socs3_qpcr_25	-15	-0.9561	+6	1	$+1.11 \cdot 10^{-01}$	1
643	sd_socs3_qpcr_26	-15	-0.8671	+6	1	$+1.36 \cdot 10^{-01}$	1
644	sd_socs3_qpcr_27	-15	-1.3056	+6	1	$+4.95 \cdot 10^{-02}$	1
645	sd_socs3_qpcr_28	-15	-0.9718	+6	1	$+1.07 \cdot 10^{-01}$	1
646	sd_socs3_qpcr_29	-15	-0.9923	+6	1	$+1.02 \cdot 10^{-01}$	1
647	sd_socs3_qpcr_30	-15	-1.4964	+6	1	$+3.19 \cdot 10^{-02}$	1
648	sd_socs3_qpcr_44	-15	-0.8194	+6	1	$+1.52 \cdot 10^{-01}$	1
649	sd_socs3_qpcr_52	-15	-1.0683	+6	1	$+8.54 \cdot 10^{-02}$	1
650	sd_socs3_qpcr_53	-15	-0.7697	+6	1	$+1.70 \cdot 10^{-01}$	1
651	sd_socs3_qpcr_62	-5	-1.3987	+3	1	$+3.99 \cdot 10^{-02}$	1
652	sd_socs3_qpcr_63	-5	-1.5849	+3	1	$+2.60 \cdot 10^{-02}$	1
653	sd_socs3_qpcr_64	-5	-1.5096	+3	1	$+3.09 \cdot 10^{-02}$	1
654	sd_socs3_wb_14	-15	-0.4012	+6	1	$+3.97 \cdot 10^{-01}$	1
655	sd_socs3_wb_15	-15	-0.4168	+6	1	$+3.83 \cdot 10^{-01}$	1
656	sd_socs3_wb_16	-15	-0.9758	+6	1	$+1.06 \cdot 10^{-01}$	1
657	sd_socs3_wb_17	-15	-0.6930	+6	1	$+2.03 \cdot 10^{-01}$	1
658	socs3_assoc	-5	-1.7135	+3	1	$+1.93 \cdot 10^{-02}$	1
659	socs3_des	-15	-0.3481	+6	1	$+4.49 \cdot 10^{-01}$	1
660	socs3_pro	-15	+0.2299	+6	1	$+1.70 \cdot 10^{+00}$	1
661	socs3rna_delay	-15	-0.5172	+6	1	$+3.04 \cdot 10^{-01}$	1
662	socs3rna_des	-15	-0.5173	+6	1	$+3.04 \cdot 10^{-01}$	1
663	socs3rna_hill	-10	+0.2338	+4	1	$+1.71 \cdot 10^{+00}$	1
664	socs3rna_pro	-15	-2.7695	+10	1	$+1.70 \cdot 10^{-03}$	1
665	stat3_act_gp130	-15	-0.3856	+6	1	$+4.12 \cdot 10^{-01}$	1
666	stat3_exp	-15	+0.2946	+6	1	$+1.97 \cdot 10^{+00}$	1
667	stat3_imp	-15	-1.2265	+6	1	$+5.94 \cdot 10^{-02}$	1
668	stat3_inh_socs3	-5	-0.2084	+3	1	$+6.19 \cdot 10^{-01}$	1
669	stat3_inh_stattic	-15	-0.9984	+6	1	$+1.00 \cdot 10^{-01}$	1
670	total_STAT3	-15	+2.8921	+6	1	$+7.80 \cdot 10^{+02}$	1

Table S116: Estimated parameter values
$\hat{\theta}$ indicates the estimated value of the parameters. θ_{min} and θ_{max} indicate the upper and lower bounds for the parameters. The log-column indicates if the value of a parameter was log-transformed. If log = 1 the non-log-column indicates the non-logarithmic value of the estimate. The fitted-column indicates if the parameter value was estimated (1), was temporarily fixed (0) or if its value was fixed to a constant value (2).

Supplementary References

[1] S. Mueller, J. Huard, K. Waldow, X. Huang, L. A. D'Alessandro, S. Bohl, K. Borner, D. Grimm, S. Klamt, U. Klingmuller, and M. Schilling. T160-phosphorylated cdk2 defines threshold for hgf dependent proliferation in primary hepatocytes. *Mol Syst Biol*, 11(3):795, 2015.

[2] J. Vandesompele, K. De Preter, F. Pattyn, B. Poppe, N. Van Roy, A. De Paepe, and F. Speleman. Accurate normalization of real-time quantitative rt-pcr data by geometric averaging of multiple internal control genes. *Genome Biol*, 3(7):RESEARCH0034, 2002.

[3] A Raue, B Steiert, M Schelker, C Kreutz, T Maiwald, H Hass, J Vanlier, C Tönsing, L Adlung, R Engesser, W Mader, T Heinemann, J Hasenauer, M Schilling, T Höfer, E Klipp, F Theis, U Klingmüller, B Schöberl, and J Timmer. Data2Dynamics: a modeling environment tailored to parameter estimation in dynamical systems. *Bioinformatics*, page btv405, 2015.

[4] Uri Alon. *An introduction to systems biology: design principles of biological circuits.* CRC press, 2006.

[5] Alan C Hindmarsh, Peter N Brown, Keith E Grant, Steven L Lee, Radu Serban, Dan E Shumaker, and Carol S Woodward. SUNDIALS: Suite of nonlinear and differential/algebraic equation solvers. *ACM Transactions on Mathematical Software*, 31(3):363–396, sep 2005.

[6] L Liu, KM. McBridge, and NC Reich. Stat3 nuclear import is independent of tyrosine phosphorylation and mediated by importin-α 3. *Proceedings of the National Academy of Sciences*, 102(23):8150–8155, 2005.

[7] NC Reich and L Liu. Tracking stat nuclear traffic. *Nature Reviews Immunology*, 6(8):602–612, 2006.
